# Direct Synthesis of Highly Substituted Pyrroles and Dihydropyrroles Using Linear Selective Hydroacylation Reactions

**DOI:** 10.1002/chem.201600311

**Published:** 2016-04-23

**Authors:** Manjeet K. Majhail, Paul M. Ylioja, Michael C. Willis

**Affiliations:** ^1^ Department of ChemistryUniversity of OxfordMansfield RoadOxfordOX1 3TAUK

**Keywords:** cascade process, heterocycles, hydroacylation, pyrrole, rhodium

## Abstract

Rhodium(I) catalysts incorporating small bite‐angle diphosphine ligands, such as (Cy_2_P)_2_NMe or bis(diphenylphosphino)methane (dppm), are effective at catalysing the union of aldehydes and propargylic amines to deliver the linear hydroacylation adducts in good yields and with high selectivities. In situ treatment of the hydroacylation adducts with *p‐*TSA triggers a dehydrative cyclisation to provide the corresponding pyrroles. The use of allylic amines, in place of the propargylic substrates, delivers functionalised dihydropyrroles. The hydroacylation reactions can also be combined in a cascade process with a Rh^I^‐catalysed Suzuki‐type coupling employing aryl boronic acids, providing a three‐component assembly of highly substituted pyrroles.

## Introduction

**Scheme 1:**
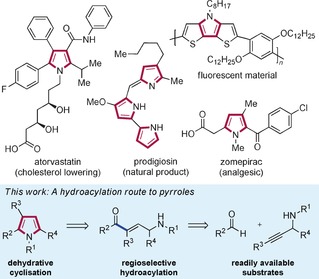
Selected examples of pyrroles in bioactive molecules and materials, and our proposed alkyne hydroacylation route to their synthesis.

The synthesis of aza‐heterocycles has been the subject of much investigation over recent years due to their abundance in nature and their wide application. Of particular significance is the pyrrole motif, as it is integral to many bioactive natural products, successful pharmaceuticals, and has a growing presence in materials science (Scheme 1).^1^ Many classical methods for the preparation of pyrroles employ 1,4‐dicarbonyl compounds,^2^ which are often limited by the availability of starting materials, especially when structural complexity is required. The surge in catalytic protocols for the construction of this ubiquitous heterocycle indicates that there is a requirement to deliver functionalised ring systems using mild reaction conditions, from simple substrates, in an experimentally straightforward fashion.^3^, ^4^ One of the driving forces for the development of these new methods is the desire to employ non‐traditional starting materials, potentially allowing access to new areas of chemical space.^5^, 1

Carbonyl‐containing compounds are archetypal intermediates for heterocycle synthesis, and consequently catalytic reactions that deliver these molecules, preferably from alternative feedstocks, are ideal candidates to be employed in these ventures, and to address many of the shortfalls associated with classical heterocycle syntheses. Transition‐metal‐catalysed intermolecular hydroacylation reactions,^6^ which combine aldehydes with alkenes and alkynes, are one such class of reaction. These reactions are atom‐economical C−H activation processes that deliver a variety of carbonyl‐containing molecules through the formation of new C−C bonds. Such processes are emerging as useful tools in synthesis, and recent years have seen the first applications of these reactions to heterocycle construction. Our laboratory has reported the synthesis of highly substituted furans through intermolecular alkyne hydroacylation,^7^ while the Dong group has reported the efficient preparation of benzofuran rings using rhodium‐catalysed coupling of aldehydes and vinylphenols.^8^ In addition, both ourselves^9^ and the Stanley group^10^ have described intramolecular conjugate addition‐based processes to access dihydroquinolones and chromanones, respectively, from hydroacylation adducts.

We wished to exploit the benefits of alkyne hydroacylation in a route to complex pyrroles; our proposed retrosynthesis is shown in Scheme 1, and involves the hydroacylative union of aldehydes and propargylic amines to deliver γ‐amino enone products.^11^ Dehydrative cyclisation of these enones would deliver the targeted pyrroles. Employing appropriately substituted aldehydes and alkynes would allow the direct synthesis of highly substituted ring systems. In this article we demonstrate how recent advances in catalyst design have allowed us to develop efficient syntheses of both pyrroles and dihydropyrroles using highly selective intermolecular alkyne and alkene hydroacylation reactions.

## Results and Discussion

**Scheme 2:**
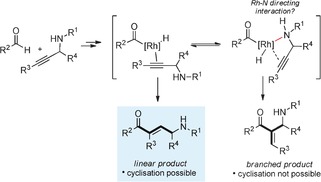
Linear versus branched selectivity in alkyne hydroacylation.

One of the main limitations of intermolecular hydroacylation reactions employing Rh^I^‐catalysts is a competing reductive decarbonylation pathway.^6^ The majority of approaches to overcome this issue invoke some form of substrate chelation, be it from the aldehyde,^12^, ^13^, ^14^, ^15^, ^16^ or alkene or alkyne.^17^ Although there are now a number of successful non‐chelation intermolecular hydroacylation methods,^18^, ^19^ the most general and selective processes invariably rely on chelation control and have allowed the development of reactions that operate under mild conditions, employ low loadings of catalyst and achieve high levels of selectivity.^20^ Accordingly, we selected chelating aldehydes as our preferred substrates. The presence of coordinating functionality on the alkene or alkyne coupling partner has also been shown to have a significant impact on the course of several hydroacylation reactions, and has been employed to facilitate both reactivity and selectivity.^17^ For example, both Dong and Suemune employed alkenes bearing coordinating functionality to achieve branched‐selective reactions,^21^ required to develop enantioselective processes. We were therefore conscious that our proposed alkyne substrates—propargylic amines—may well be involved in coordination to the metal catalyst and that this could effect the regioselectivity of the proposed reactions. Poor regiocontrol would have significant consequences for our designed pyrrole synthesis, as only the linear hydroacylation adducts are capable of undergoing a pyrrole‐forming cyclisation (Scheme 2).2

We began our investigation by studying the coupling of *o*‐SMe‐benzaldehyde **1 a** and *N*‐Boc propargylic amine **2 a** (Table 1). Our concerns over regioselectivity were soon proved correct, with reactions employing a catalyst incorporating the ligand DPEphos14c delivering the hydroacylation adducts as a 2:1 mixture of linear and branched enones, respectively (entry 1). We have recently shown that small‐bite‐angle, methylene‐bridged diphosphines generate efficient and selective catalysts for a variety of hydroacylation reactions.^22^ Unfortunately, in the present study, both bis(diphenylphosphino)methane (dppm) and bis(dicyclohexylphosphinomethane) (dcpm) ligands offered only modest improvement (entries 2–4). However, it was the electronic parameters of the PNP ligand system, specifically the ligand (Cy_2_)P_2_NMe, that offered a significant increase in the level of regiocontrol (entry 5).^23^ To achieve higher conversions we turned our attention to evaluating the effect of concentration and temperature on the reactions. Increasing the concentration to 1.0 m with respect to the aldehyde, allowed complete conversion for both the PCP and PNP systems (entry 4 and 6). When assessing the effect of temperature, the general trend indicated that decreased temperatures accomplished greater linear selectivity, without any loss in conversion under ambient conditions (entry 8). Reaction at 0 °C provided a more selective transformation but required a slightly longer reaction time (entry 9). We also evaluated Cbz‐ and Ts‐protected propargylic amines in order to determine whether the nature of the amine‐protecting group played a significant role in the selectivity of the reactions. In both cases the PNP‐derived catalyst outperformed the corresponding dcpm system, and for the Cbz substrate was able to provide the desired linear adduct with reasonable, but diminished with respect to Boc‐derivative, selectivity (entries 10 and 11). The *N*‐Ts‐substrate was poorly selective with either catalyst (entries 12 and 13). For convenience we decided to undertake subsequent reactions using the room temperature conditions shown in entry 8, and to employ *N*‐Boc‐protected amines.1

**Table 1:** Ligand evaluation for the hydroacylation reaction between aldehyde **1 a** and propargylic amine **2 a**.^[a]^

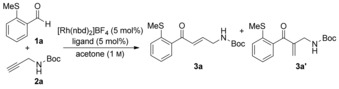						
Entry	PG	Ligand	Time	*T* [°C]	Conv. [%]^[b]^	**3 a**/**3 a′** ^[b]^
1^[c]^	Boc	DPEphos	16 h	55	41 (32)	2:1
2^[c]^	Boc	dppm	16 h	55	76 (69)	5:1
3^[c]^	Boc	dcpm	16 h	55	85	3:1
4	Boc	dcpm	1 h	55	100 (87)	5:1
5^[c]^	Boc	PNP(Cy)	16 h	55	78	9:1
6	Boc	PNP(Cy)	10 min	55	100	10:1
7	Boc	PNP(Cy)	10 min	80^[d]^	100	7:1
8^[e]^	Boc	PNP(Cy)	30 min	22	100^[f]^ (90)	17:1
9	Boc	PNP(Cy)	2 h	0	100 (94)	20:1
10	Cbz	dcpm	16 h	22	100 (79)	4:1
11	Cbz	PNP(Cy)	30 min	22	100 (82)	12:1
12	Ts	dcpm	16 h	22	100	1:1
13	Ts	PNP(Cy)	10 min	22	100	4:1

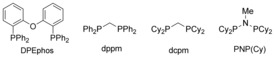						

[a] Reaction conditions: **1 a** (1.0 equiv, 0.15 mmol), **2 a** (1.3 equiv), [Rh(nbd)_2_]BF_4_ (5 mol %), ligand (5 mol %), acetone, 1 m with respect to aldehyde. [b] Determined by ^1^H NMR analysis of the crude reaction mixture. Value in parenthesis is the combined isolated yield of the two regioisomers. [c] 0.3 m with respect to aldehyde. [d] Performed in 1,2‐dichloroethane. [e] [Rh(nbd)_2_]BF_4_ (2 mol %), PNP(Cy) (2 mol %). [f] 86 % isolated yield of linear isomer. PG=protecting group.

We next investigated whether this intermolecular hydroacylation could maintain high levels of selectivity when varying the propargylic amine substrates (Table 2). Pleasingly, introducing substituents α to the nitrogen atom displayed enhanced selectivity for the linear product (**3 b**–**d**), demonstrating that further improvements in regiocontrol could be achieved by substrate design. A range of aldehydes, including substituted aryl (**3 f**), alkyl (**3 g**), alkenyl (**3 h**), heteroaromatic variants (**3 i**), and the use of a nitrogen‐chelating atom (**3 j**), were also introduced without incident. We then expanded the process to include internal alkyne substrates, which following cyclisation would allow the direct formation of tetra‐substituted pyrroles. The hydroacylation of an internal alkyne proceeded efficiently, although an alternative ligand was required (**3 e**); the PNP(Cy)‐based catalyst did not deliver the necessary regiocontrol (2:1, linear: branched), however, when using dppm, a PCP‐based ligand system, acceptable levels of linear‐selectivity were achieved (8:1). This brief study also established that it was possible to employ a catalyst loading of only 1 mol % (**3 d**), although for pragmatic reasons the majority of reactions were performed using a 5 mol % loading.2

**Table 2:** Substrate scope for linear selective preparation of γ‐amino enones.^[a]^

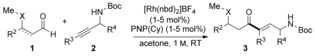
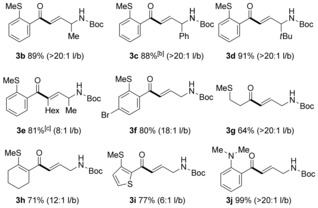

[a] Reaction conditions: aldehyde (1.0 equiv, 0.15 mmol), alkyne (1.3 equiv), [Rh(nbd)_2_]BF_4_ (5 mol %), PNP(Cy) (5 mol %), acetone (1 m with respect to aldehyde), RT. Yields are of isolated linear products; l/b ratios were determined by ^1^H NMR analysis of the crude reaction mixture. [b] Aldehyde (3.0 mmol, 1.0 equiv), [Rh(nbd)_2_]BF_4_ (1 mol %), PNP(Cy) (1 mol %). [c] dppm (5 mol %) used in place of PNP(Cy).

With a selective hydroacylation reaction in hand, we next studied the cyclisation to generate the desired pyrroles. γ‐Amino enones are known to undergo Brønsted‐acid‐catalysed cyclisation,^11^ and pleasingly, addition of *p*‐toluene sulfonic acid and a small quantity of acetonitrile directly to the reaction mixtures upon completion of the hydroacylation process allowed rapid and clean conversions to the substituted pyrroles. A series of *N*‐Boc functionalised pyrroles was then prepared using this one‐pot cascade process (Table 3). Varied substituents at the propargylic position could be readily installed, with alkyl (**4 b**–**c** and **4 u**), functionalised aryl and heteroaryl (**4 k**, **4 n** and **4 v**) groups all tolerated. In particular, it is noteworthy that the aryl substituents could possess steric bulk at the hindered *ortho*‐position (**4 r**) and contain useful functionality such as halides (**4 m**), esters (**4 s**) and nitrile (**4 f**). In addition, owing to the excellent functional group tolerance at the 2‐ and 5‐position of the pyrrole ring, we were able to directly synthesise several interesting linked heterocycles in a concise manner (**4 k**, **n**, **u** and **4 v**). Encouragingly, a larger scale preparation of pyrrole **4 d** was also possible, achieving an 88 % yield when using only 1 mol % of the catalyst, affording over 3

**Table 3:** Substrate scope of the cascade pyrrole synthesis through γ‐amino enones.^[a]^

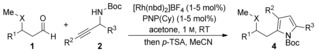
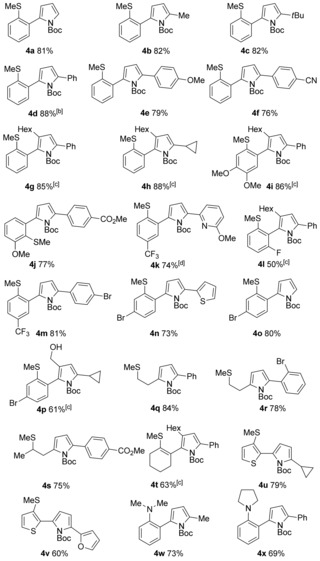

[a] Reaction conditions: aldehyde (1.0 equiv, 0.15 mmol), alkyne (1.3 equiv), [Rh(nbd)_2_]BF_4_ (5 mol %), PNP(Cy) (5 mol %), acetone (1 m with respect to aldehyde), RT. Yields are of isolated products. [b] Aldehyde (1.0 equiv, 3.2 mmol), [Rh(nbd)_2_]BF_4_ (1 mol %), PNP(Cy) (1 mol %), 1.04 g of isolated product. [c] dppm (5 mol %) used in place of PNP(Cy). [d] [Rh(nbd)_2_]BF_4_ (7 mol %), PNP(Cy) (7 mol %).

Substitution on every position of the aromatic‐ring of aryl aldehydes was achieved in good‐to‐excellent yields, including the sterically hindered *ortho*‐position (**4 l**), and halide substituents, which have potential for further derivatisation (**4 l**, **4 n**–**p**). Heteroaryl groups, in the form of thiophene‐derived aldehydes, also proved to be good substrates (**4 u**, **v**). Alkyl (**4 q**–**s**) and alkenyl (**4 t**) aldehydes displayed good reactivity, although lower regioselectivity in the hydroacylation step was observed. We were also successful in exchanging the chelating atom to nitrogen (**4 w**, **x**).^9^, 15c In addition, we were able to prepare a range of tri‐substituted *N*‐Boc‐pyrroles using internal propargylic amines in conjunction with a dppm derived catalyst; alkyl chains (**4 g**–**I**, **4 l** and **4 t**) as well as a hydroxymethyl group (**4 p**) were successfully installed.

**Scheme 3:**
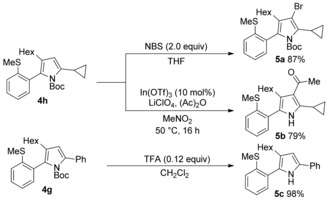
C‐3 functionalisation and *N*‐deprotection.

A limitation of the presented synthesis is the use of an alkyne as one of the reaction components, as this necessarily means that the C‐3 position of the pyrrole products is unsubstituted. Accordingly, we chose to demonstrate the preparation of fully substituted pyrroles from the functionalisation of representative tetra‐substituted examples (Scheme 3). Treatment of pyrrole **4 h** with NBS delivered the 3‐Br‐derivative (**5 a**) in excellent yield. An acyl group could be installed employing In(OTf)_3_‐catalysed acylation (**5 b**).^24^ We also established that *N*‐deprotection was straightforward, with TFA treatment of pyrrole **4 g** delivering the parent NH‐pyrrole (**5 c**).3

As the examples in Table 3 demonstrate, the use of chelating aldehydes as substrates allowed the preparation of a broad range of pyrroles, featuring diverse substituents, in good to excellent yields. However, the caveat to employing chelating aldehydes as substrates is that the pyrrole products also feature the chelating substituent. In the majority of examples, this chelating substituent is a MeS group. We have recently shown that for aryl methyl sulfides, the methyl sulfide group can function as an activating group for a variety of rhodium‐catalysed processes.^25^ When translated to the present study, we were able to show that we can obtain “traceless” pyrrole products by combining the key hydroacylation reactions with MeS‐Suzuki‐type couplings using aryl boronic acids (Table 4). In order to achieve efficient hydroacylation and Suzuki‐type reactions it was necessary to employ a mixed ligand system, in which both PNP(Cy) and dcpm were used in combination with a common Rh^I^ salt. This approach allowed the preparation of pyrroles **6 a**–**c**, resulting from the union of an aryl aldehyde, a propargylic amine and an aryl boronic acid, and involving the formation of two C−C and one C−N bonds.4

**Table 4:** Three‐component couplings leading to pyrroles **6 a**–**c**.^[a]^

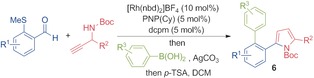
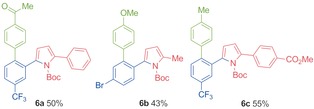

[a] Reaction conditions: aldehyde (1.0 equiv, 0.15 mmol), alkyne (1.3 equiv), [Rh(nbd)_2_]BF_4_ (10 mol %), PNP(Cy) (5 mol %), dcpm (5 mol %), acetone (1 m with respect to aldehyde), 55 °C, 10 min; then Ag_2_CO_3_ (1.0 equiv), aryl boronic acid (1.5 equiv), acetone (0.3 m with respect to aldehyde), 55 °C, 4 h; then filter through silica plug and add *p*‐TSA (1.0 equiv). Yields are of isolated products.

To extend the general process to the synthesis of dihydropyrroles required the hydroacylative coupling of aldehydes with allylic amines (Table 5). Pleasingly, use of the PNP(Cy)‐derived catalyst allowed efficient reactions of allyl amines with S‐chelating aldehydes. A variety of alkyl (**7 b**, **d**), aryl (**7 a**, **g**, **h**) and heteroaryl (**7 c**) substituents were tolerated at the allylic position of the alkenes. Substituted aryl (**7 c**–**e**), as well as heteroaryl (**7 f**, **7 g**) aldehydes could also be employed without incident.5 Unfortunately, aldehydes featuring a coordinating amino‐substituent proved to be unreactive in this system,^9^ as did disubstituted alkenes. As in the pyrrole syntheses, the hydroacylation adducts were treated directly with *p*‐TSA to induce cyclisation. In the event, acid treatment also induced cleavage of the Boc‐group, resulting in the formation of dihydropyrroles (**7 a**–**h**) in good‐to‐excellent yields.

**Table 5:** Dihydropyrrole synthesis from the combination of S‐chelating aldehydes and allylic amines.^[a]^

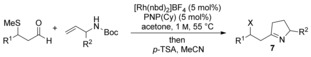
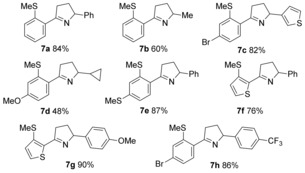

[a] Reaction conditions: aldehyde (1.0 equiv, 0.15 mmol), alkyne (1.5 equiv), [Rh(nbd)_2_]BF_4_ (5 mol %), ligand (5 mol %), acetone (1 m with respect to aldehyde), 55 °C. Yields are of isolated products.

Dihydropyrroles are useful substrates to access the corresponding saturated heterocycles. For example, reduction of dihydropyrroles to form the pyrrolidines through the *syn*‐addition of hydride was achieved by the use of diisobutylaluminium hydride (DIBAL‐H) (Table 6). Employing this bulky reducing agent allowed the formation of a range of *cis*‐pyrrolidines as the only observed diastereoisomer.^26^ However, formation of the *trans‐*pyrrolidines was not as straightforward, with the use of sodium borohydride resulting in a mixture of diastereoisomers in favour of the *trans*‐isomer.^27^ For example, pure *trans*‐pyrrolidine **8 g** was obtained in 68 % yield from a crude 3:1 mixture of diastereomers. Finally, an allyl Grignard reagent could also be employed as the nucleophile, delivering pyrrolidine **8 h**, featuring a quaternary centre, in excellent yield and diastereoselectivity.^28^, 6

**Table 6:** Reduction of dihydropyrroles to pyrrolidines.^[a]^

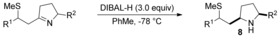
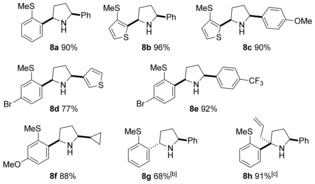

[a] Reaction conditions: dihydropyrrole (1.0 equiv), DIBAL‐H (3.0 equiv). Yields are of isolated products, with >20:1 d.r. as determined by ^1^H NMR analysis. [b] NaBH_4_ (2.5 equiv), AcOH, RT, in place of DIBAL‐H in PhMe. Crude product obtained as a 3:1, *trans*/*cis* mixture, as determined by ^1^H NMR analysis. [c] Allyl magnesium bromide (1.5 equiv), used in place of DIBAL‐H.

## Conclusions

We have developed a rhodium‐based catalyst system that allows the linear selective hydroacylation of propargylic amines. We have further demonstrated the applicability of the intermediates obtained from this novel hydroacylation in the direct synthesis of tri‐ and tetra‐substituted pyrroles, with the ability to install a variety of functional groups in a one‐pot cascade. We have shown that access to fully substituted pyrroles can be obtained by classical brominations and acylations. Furthermore, we have demonstrated that the methyl sulfide group, utilised as a chelating motif for the hydroacylation reaction, can act as a pseudo‐halide and be exploited in telescoped cross‐coupling reactions using the same catalytic species required for the initial C−H activation. These cascade Suzuki‐type reactions allow access to considerable structural diversity from simple starting materials in a three‐component assembly, using an experimentally straightforward method. In addition, we have demonstrated that the methodology developed for the coupling of alkynes and aldehydes is equally applicable to alkenes, allowing extension of the methodology to the synthesis of dihydropyrroles, and by derivatisation, to pyrrolidines.

## Experimental Section

### General procedure for the synthesis of *N*‐Boc pyrroles as exemplified by the synthesis of *tert*‐butyl 2‐[2‐(methylthio)phenyl]‐1*H*‐pyrrole‐1‐carboxylate (4 a)

An oven‐dried microwave vial was charged with [Rh(nbd)_2_BF_4_] (2.8 mg, 5 mol %) [nbd=norbornadiene] and PNP(Cy) (3.2 mg, 5 mol %). Once under an inert atmosphere, they were dissolved in acetone (1 mL). Hydrogen gas was bubbled through the solution at room temperature for 1–2 min in order to generate the active catalyst species. The hydrogen was purged using nitrogen gas, and this was bubbled through the catalyst to dryness. The dry catalyst was dissolved in acetone (75 μL) and this was transferred to a nitrogen‐filled microwave vial containing 2‐(methylthio)benzaldehyde (**1 a**; 19 μL, 0.15 mmol, 1.0 equiv) and *N*‐Boc‐propargylamine (**2 a**; 30.2 mg, 0.195 mmol, 1.3 equiv). The reaction mixture, once homogenous (on occasion, sonication was required), was then stirred at room temperature and monitored by TLC until complete. After 20 min, the reaction vessel was opened to air followed by the addition of acetonitrile (1.5 mL) and *p*‐TSA (42.8 mg, 0.230 mmol, 1.5 equiv). The reaction mixture was further stirred until complete. After 3 h the solution was diluted with acetonitrile (5 mL) and neutralised by the addition of sat. NaHCO_3(aq.)_ (10 mL) in a separatory funnel. The aqueous mixture was extracted with EtOAc (3×5 mL) and the combined organic extracts were washed with brine (10 mL) and dried over MgSO_4_. The solvent was removed in vacuo to obtain the crude product, and this was then purified by flash column chromatography (5–10 % Et_2_O in petrol) to afford title pyrrole **4 a** as a colourless oil (34.8 mg, 81 %).

## Supporting information

As a service to our authors and readers, this journal provides supporting information supplied by the authors. Such materials are peer reviewed and may be re‐organized for online delivery, but are not copy‐edited or typeset. Technical support issues arising from supporting information (other than missing files) should be addressed to the authors.

Supplementary
