# Supplementary material for: Direct Synthesis of Highly Substituted Pyrroles and Dihydropyrroles Using Linear Selective Hydroacylation Reactions
Source: Chemistry. 2016 Apr 23;22(23):7879–84. doi: 10.1002/chem.201600311 (PMC5074311; doi:10.1002/chem.201600311)

CHEMISTRY
A European Journal

Supporting Information

Direct Synthesis of Highly Substituted Pyrroles and Dihydropyrroles Using Linear Selective Hydroacylation Reactions

Manjeet K. Majhail, Paul M. Ylioja, and Michael C. Willis*[a]

chem_201600311_sm_miscellaneous_information.pdf

Direct Synthesis of Highly Substituted Pyrroles and Dihydropyrroles Using Linear Selective Hydroacylation Reactions

Manjeet K. Majhail, Paul M. Ylioja and Michael C. Willis

Department of Chemistry, Chemistry Research Laboratory, University of Oxford, Mansfield Road, Oxford, OX1 3TA, UK

michael.willis@chem.ox.ac.uk

Supporting Information

Table of Contents

1. General experimental methods S2
2. Synthesis of novel aldehydes for hydroacylation S3
3. Synthesis of novel α-amido sulfones (intermediates for the formation of propargylic and allylic amines) S7
4. Synthesis of novel propargylic amines S10
5. Synthesis of novel allylic amines S19
6. Hydroacylation products from aldehyde and propargylic amine coupling S22
7. One-pot formation of functionalised pyrrole rings S32
8. Functionalising existing pyrroles S48
9. Synthesis of pyrroles *via* a telescoped hydroacylation-Suzuki-cyclisation S51
10. One-pot synthesis of dihydropyrroles S54
11. Reduction of dihydropyrrole products S59
12. References S66
13. NMR Spectra of novel compounds S67

1. General experimental methods

Reactions were conducted with continuous magnetic stirring under an inert atmosphere of nitrogen and using dry solvents unless otherwise stated. Nitrogen gas was passed through a Drierite® and silica filled drying tube before use. Glassware was oven-dried (>200 °C), and allowed to cool to room temperature under vacuum once assembled. Reagents were purchased from Sigma-Aldrich Chemical Co. Ltd., Acros Organics Ltd, Alfa Aesar or Fluorochem Ltd. and used as supplied. PNP(Cy) was synthesised according to a reported procedure[1] and was weighed out in air and stored in a Schlenk tube under nitrogen after use. Solvents were collected fresh from an in-house solvent purification system, which involves passing the solvent through anhydrous alumina columns using an Innovative Technology Inc. PS-400-7 solvent purification system. Acetone, for use in rhodium reactions, was purchased from Sigma-Aldrich (HPLC grade), distilled over Drierite® and degassed prior to use.

Reactions were monitored by thin layer chromatography (TLC) using pre-coated aluminium backed silica plates (Merck Kieselgel 60 F254). Plates were visualised under ultraviolet light (254 nm) followed by staining with potassium permanganate, vanillin or Seebach's stain (Magic). Flash column chromatography was carried out using matrix 60 silica.

^{1}H NMR spectra were obtained on a Bruker AVIII400 (400 MHz) spectrometer using the residual solvent as an internal standard. ^{13}C NMR spectra were obtained on a Bruker AVIII400 (101 MHz) spectrometer using the residual solvent as an internal standard. Chemical shifts were reported in parts per million (ppm) with the multiplicities of the spectra reported as following: s, singlet; d, doublet; t, triplet; q, quartet; m, multiplet; br, broad; app, apparent. Low-resolution ESI mass spectra were recorded on a Waters LCT Premier spectrometer. High-resolution ESI mass spectrometry measurements were recorded on a Brucker Daltronics microTOF (ESI^{+}) spectrometer or Micromass LCT under the conditions of field iniosation (FI) by the internal service at the Department of Organic Chemistry, University of Oxford. Values quoted are a ratio of mass to charge in Daltons and relative intensities of peaks observed are quoted as a percentage. Infra-red spectra were recorded as thin films on a Bruker Tensor 27 FT-IR spectrometer with internal calibration in the range 4000-600 cm^{-1}. Melting points were determined using a Stuart Scientific Melting Point Apparatus SMP1.

2. Synthesis of novel aldehydes for hydroacylation

The following aldehydes are commercially available and were distilled prior to use:

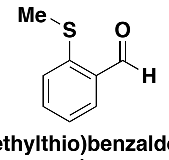

2-(Methylthio)benzaldehyde
1a

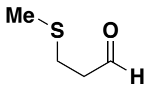

3-(Methylthio)propanal
1b

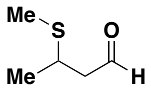

3-(Methylthio)butanal
1c

The synthetic procedures and characterisation have previously been reported for the following aldehydes:

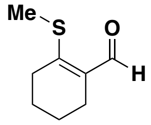

2-(Methylthio)cyclohex-1-ene-1-carbaldehyde[2]
1d

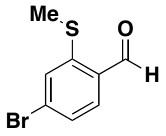

4-Bromo-2-(methylthio) benzaldehyde[3]
1e

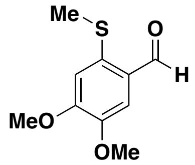

4,5-Dimethoxy-2-(methylthio) benzaldehyde[4]
1f

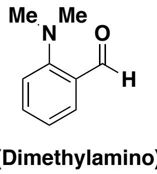

2-(Dimethylamino) benzaldehyde[5]
1g

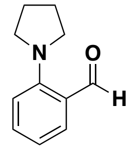

2-(Pyrrolidin-1-yl) benzaldehyde[5]
1h

General procedure A for the synthesis of *ß*-sulfur tethered aldehydes, exemplified by the preparation of 3-methoxy-2-(methylthio)benzaldehyde, 1i

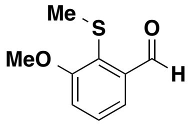

Methanethiol sodium salt solution (1.28 mL, 3.0 M in water, 3.84 mmol, 1.2 equiv.) was added dropwise to 2-fluoro-3-methoxybenzaldehyde (0.50 g, 3.20 mmol, 1.0 equiv.) in DMF (4 mL) at 0 °C. The stirring solution was warmed to room temperature over 16 hrs, after which it was diluted with water (15 mL). The aqueous mixture was extracted with Et_2O (3 × 10 mL) and the combined organic extracts were washed with sat. $LiCl_{(aq)}$ (3 × 10 mL), dried over $MgSO_4$ and concentrated under reduced pressure. Purification *via* flash column chromatography (20% Et_2O in petrol) afforded title *aldehyde* **1i** as a white solid (0.27 g, 46%).

^{1}H NMR (400 MHz, $CDCl_3$): δ 10.80 (1H, s, C*H*O), 7.53 (1H, dd, *J* 8.0, 1.5, Ar-*H*), 7.41 (1H, app t, *J* 8.0, Ar-*H*), 7.14 (1H, dd, *J* 8.0, 1.5, Ar-*H*), 3.98 (3H, s, OC*H₃*), 2.43 (3H, s, SC*H₃*); ^{13}C NMR (101 MHz, $CDCl_3$): δ 192.6, 160.4, 138.5, 129.4, 128.7, 120.2, 115.8, 56.3, 18.9; IR: ν_{max} (neat)/cm^{-1} 2750, 1691, 1571, 1464, 1264, 1237, 1064, 906, 729; LRMS (ESI$^+$): *m/z* 183 ([M+H]$^+$, 25%), 205 ([M+Na]$^+$, 100%); HRMS (ESI$^+$) found *m/z* 183.04719 [M+H]$^+$, $C_9H_{11}O_2S^+$ requires 183.04743; mp: 59-60 °C (EtOAc/hexane).

2-Fluoro-6-(methylthio)benzaldehyde, 1j

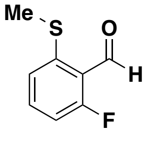

Prepared according to general procedure **A** using methanethiol sodium salt solution (1.52 mL, 3.0 M in water, 15.5 mmol, 1.1 equiv.) and 2, 6-difluorobenzaldehyde (1.52 mL, 14.1 mmol, 1.0 equiv.) in DMF (20 mL). The crude oil was purified by flash column chromatography (20-50% DCM in toluene) to obtain *aldehyde* **1j** as a pale yellow solid (0.95 g, 40%).

^{1}H NMR (400 MHz, $CDCl_3$): δ 10.51 (1H, s, C*H*O), 7.51 (1H, dd, *J* 8.0, 5.0, Ar-*H*), 7.08 (1H, d, *J* 8.0, Ar-*H*), 6.91 (1H, app t, *J* 8.0, Ar-*H*), 2.48 (3H, s, SCH_3); ^{13}C NMR (101 MHz, $CDCl_3$): δ 187.2, 166.7 (d, $^1J_{CF}$ 260.0), 146.1, 134.9 (d, $^2J_{CF}$ 22.0), 120.6 (d, $^3J_{CF}$ 4.0), 119.80 (d, $^3J_{CF}$ 3.5), 110.8 (d, $^2J_{CF}$ 21.5), 15.4; ^{19}F NMR (377 MHz, $CDCl_3$): δ -120.3; IR: ν_{max} (neat)/cm^{-1} 2885, 1684, 1560, 1557, 1454, 1323, 1237, 1204, 908, 832, 777, 662; LRMS (ESI$^+$): *m/z* 171 ([M+H]$^+$, 10%), 193 ([M+Na]$^+$, 100%); HRMS (ESI$^+$) found 193.00933 [M+Na]$^+$, $C_8H_7OFSNa^+$ requires 193.00939; mp: 51-53 °C (EtOAc/hexane).

4-Methoxy-2-(methylthio)benzaldehyde, 1k

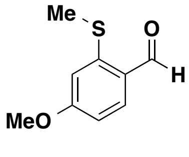

Prepared according to general procedure **A** using methanethiol sodium salt solution (5.60 mL, 3.0 M in water, 16.9 mmol, 1.3 equiv.) and 2-fluoro-4-methoxybenzaldehyde (2.00 g, 13.0 mmol, 1.0 equiv.) in DMF (18 mL). The crude was purified by flash column chromatography (5-10% EtOAc in petrol) to obtain *aldehyde* **1k** as an off-white solid (2.03 g, 86%).

^{1}H NMR (400 MHz, $CDCl_3$): δ 10.11 (1H, s, C*H*O), 7.78 (1H, d, *J* 8.5, Ar-*H*), 6.82-6.78 (2H, m, Ar-*H*), 3.92 (3H, s, OCH_3), 2.49 (3H, s, SCH_3); ^{13}C NMR (101 MHz, $CDCl_3$): δ 189.7, 164.0, 145.7, 136.2, 126.5, 111.0, 109.1, 55.6, 15.1; IR: ν_{max}

(neat)/cm^{-1} 2839, 1671, 1589, 1551, 1278, 1234, 1064, 1035, 891, 805, 611; LRMS (ESI$^+$): *m/z* 205 ([M+Na]$^+$, 100%); HRMS (ESI$^+$) found 205.02926 [M+Na]$^+$, $C_9H_{10}O_2SNa^+$ requires 205.02937; mp: 84-85 °C (EtOAc/petrol).

2,4-Bis(methylthio)benzaldehyde, 1l

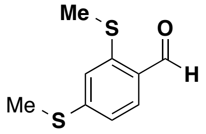

Prepared according to general procedure **A** using methanethiol sodium salt solution (10.4 mL, 3.0 M in water, 31.1 mmol, 2.1 equiv.) and 2-fluoro-4-bromobenzaldehyde (3.00 g, 14.8 mmol, 1.0 equiv.) in DMF (18 mL). The crude was purified by flash column chromatography (10-30% Et_2O in petrol) to obtain *aldehyde* **1l** as off-white needles (0.96 g, 33%).

^{1}H NMR (400 MHz, $CDCl_3$): δ 10.15 (1H, s, C*H*O), 7.70 (1H, dd, *J* 8.0, 1.0, Ar-*H*), 7.34-7.22 (1H, m, Ar-*H*), 7.07 (1H, d, *J* 8.0, Ar-*H*), 2.56 (3H, s, *ortho*-SC*H$_3$*), 2.50 (3H, s, *para*-SC*H$_3$*); ^{13}C NMR (101 MHz, $CDCl_3$): δ 190.2, 147.7, 143.8, 133.5, 129.5, 121.3, 120.5, 15.4, 14.7; IR: ν_{max} (neat)/cm^{-1} 2920, 2835, 1675, 1574, 1534, 1286, 1108, 955, 862; LRMS (ESI$^+$): *m/z* 199 ([M+H]$^+$, 10%), 221 ([M+Na]$^+$, 100%); HRMS (ESI$^+$) found 199.02465 [M+H]$^+$, $C_9H_{11}OS_2^+$ requires 199.02458; mp: 64-66 °C (DCM/petrol).

3-(Methylthio)thiophene-2-carbaldehyde, 1m

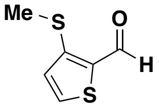

Prepared according to general procedure **A** using methanethiol sodium salt solution (5.80 mL, 3.0 M in water, 17.3 mmol, 1.1 equiv.) and 3-bromothiophene-2-carboxaldehyde (1.70 mL, 15.7 mmol, 1.0 equiv.) in DMF (19 mL). The crude was purified by flash column chromatography (10% Et_2O in petrol) to obtain a brown solid, which was recrystalised (DCM/hexane) to afford *aldehyde* **1m** as white needles (0.91 g, 37%).

^{1}H NMR (400 MHz, $CDCl_3$): δ 10.06 (1H, s, C*H*O), 7.74 (1H, dd, *J* 5.0, 1.0, Ar-*H*), 7.12 (1H, d, *J* 5.0, Ar-*H*), 2.61 (3H, s, SC*H$_3$*); ^{13}C NMR (101 MHz, $CDCl_3$): 181.5, 146.0, 135.2, 134.6, 128.1, 17.1; IR: ν_{max} (neat)/cm^{-1} 3101, 2839, 1648, 1478, 1365, 1223, 1777, 907, 665; LRMS (ESI$^+$): *m/z* 159 ([M+H]$^+$, 50%), 181 ([M+Na]$^+$, 100%); HRMS (ESI$^+$) found 180.97536 [M+Na]$^+$, $C_6H_6OS_2Na^+$ requires 180.97523; mp: 34-35 °C (DCM/hexnae).

2-(Methylthio)-5-(trifluoromethyl)benzaldehyde, 1n

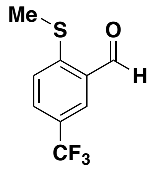

Prepared according to general procedure **A** using methanethiol sodium salt solution (1.90 mL, 3.0 M in water, 5.75 mmol, 1.1 equiv.) and 2-fluoro-5-(trifluoromethyl)benzaldehyde (0.74 mL, 5.20 mmol, 1.0 equiv.) in DMF (10 mL). The crude was purified by flash column chromatography (30% Et_2O in petrol) to afford *aldehyde* **1n** as a white solid (0.85 g, 74%).

^{1}H NMR (400 MHz, $CDCl_3$): δ 10.28 (1H, s, C*H*O), 8.07 (1H, d, *J* 1.5, Ar-*H*), 7.77 (1H, dd, *J* 8.5, 1.5, Ar-*H*), 7.46 (1H, d, *J* 8.5, Ar-*H*), 2.57 (3H, s, SC*H*$_3$); ^{13}C NMR (101 MHz, $CDCl_3$): 190.0, 148.4, 130.2 (q, $^3J_{CF}$ 3.5), 129.9 (q, $^3J_{CF}$ 3.5), 127.0, 126.6 (q, $^2J_{CF}$ 30.5), 125.1, 123.6 (q, $^1J_{CF}$ 250.0), 15.2; ^{19}F NMR (377 MHz, $CDCl_3$): -62.5; IR: ν_{max} (neat)/cm^{-1} 3041, 1686, 1611, 1556, 1350, 1334, 1310, 1262, 1114; LRMS (ESI$^+$): *m/z* 221 ([M+H]$^+$, 100%); HRMS (ESI$^+$) found 221.02435 [M+H]$^+$, $C_9H_8OF_3S^+$ requires 221.02425; mp: 102-104 °C (DCM/petrol).

3. Synthesis of novel α-amido sulfones (intermediates for the formation of propargylic and allylic amines)

The synthetic procedures and characterisation have previously been reported for the following α-amido sulfones:

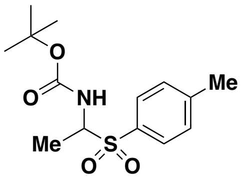

tert-Butyl (1-tosylethyl)carbamate[6]
9a

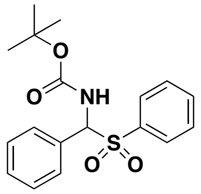

tert-Butyl [phenyl(phenylsulfonyl) methyl]carbamate[7]
9b

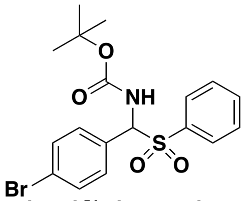

tert-butyl [(4-bromophenyl) (phenylsulfonyl)methyl]carbamate[8]
9c

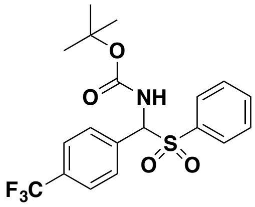

tert-Butyl [(phenylsulfonyl)[4-(trifluoromethyl)phenyl]methy]carbamate[8]
9d

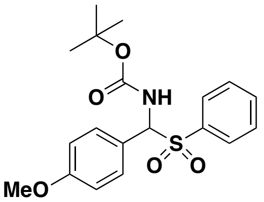

tert-Butyl [(4-methoxyphenyl) (phenylsulfonyl)methyl]carbamate[9]
9e

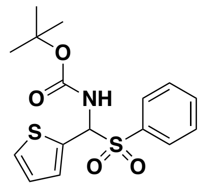

tert-butyl [(phenylsulfonyl)(thiophen-2-yl)-methyl]carbamate[9]
9f

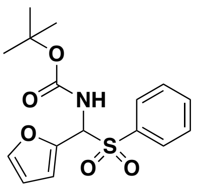

tert-Butyl [furan-2-yl(phenylsulfonyl) methy]carbamate[9]
9g

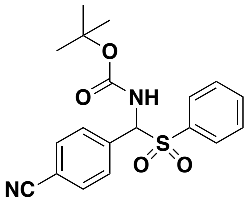

tert-Butyl [(4-cyanophenyl)(phenylsulfonyl)-methyl]carbamate[9]
9h

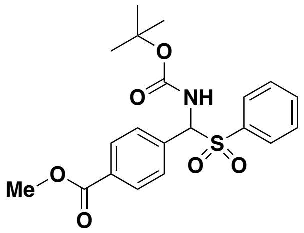

Methyl 4-{[(*tert*-butoxycarbonyl)amino] (phenylsulfonyl)methyl}benzoate[10]
9i

General procedure B for the synthesis of α-amido sulfones, exemplified by the preparation of *tert*-butyl[[6-methoxypyridin-2-yl)-(phenylsulfonyl)-methyl]carbamate, 9j

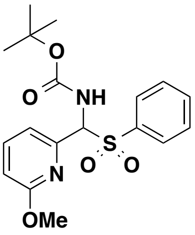

Prepared following an adapted literature procedure.[7] To a stirring solution of *tert*-butyl-carbamate (1.60 g, 13.3 mmol, 1.0 equiv.) and sodium phenylsulfinate (4.80 g, 26.6 mmol, 2.0 equiv.) in H_2O/MeOH (2:1, v/v) (34.2 mL) was added 6-methoxypyridine-2-carbaldehyde (1.93 mL, 16.0 mmol, 1.2 equiv.) and formic acid (1.51 mL, 39.9 mmol, 1.5 equiv.) at room temperature. The mixture was

stirred for 60 hrs. The resulting white precipitate was filtered with 10% DCM in petrol and triturated (10% DCM in petrol) for 2 hrs. After filtration the white solid was dried under vacuum to afford pure *α-amido sulfone* **9j** as a white solid (3.74 g, 74%).

^1H NMR (400 MHz, $CDCl_3$): δ 7.87 (2H, d, *J* 7.5, Ar-*H*), 7.64 (2H, t, *J* 7.5, Ar-*H*), 7.53 (2H, t, *J* 7.5, Ar-*H*), 7.17 (1H, d, *J* 7.0, Ar-*H*), 6.79 (1H, d, *J* 8.0, Ar-*H*), 6.47 (1H, br d, *J* 9.5, N*H*), 5.98 (1H, d, *J* 9.5, NHC*H*), 3.86 (3H, s, OCH_3), 1.34 (9H, s, $CO_2C(CH_3)_3$); ^{13}C NMR (101 MHz, $CDCl_3$): δ 163.8, 153.7, 145.4, 139.1, 136.8, 133.8, 129.7, 128.8, 118.4, 112.0, 81.0, 74.6, 53.5, 28.1; IR: ν_{max} (neat)/cm^{-1} 2979, 1719, 1603, 1576, 1447, 1416, 1368, 1344, 1143, 989; HRMS (FI$^+$) found 378.1389 [M]$^+$, $C_{18}H_{22}N_2O_5S^+$ requires 378.1249; mp: 136-137 °C (EtOAc/hexane).

***tert*-Butyl [(2-bromophenyl)(phenylsulfonyl)methyl]carbamate, 9k**

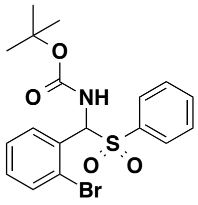

Prepared according to general procedure **B** using *tert*-butyl-carbamate (1.50 g, 12.8 mmol, 1.0 equiv.), sodium phenylsulfinate (5.70 g, 32.0 mmol, 2.5 equiv.) in H_2O/THF (2:1, v/v) (38.4 mL), 2-bromobenzaldehyde (1.80 mL, 15.4 mmol, 1.2 equiv.) and formic acid (0.72 mL, 19.2 mmol, 2.0 equiv.). The mixture was stirred for 60 hrs. Trituration afforded *α-amido sulfone* **9k** as a white powder (3.02 g, 55%).

^1H NMR (400 MHz, CD_2Cl_2): δ 7.97 (2H, d, *J* 8.0, Ar-*H*), 7.69 (1H, t, *J* 7.5, Ar-*H*), 7.65-7.63 (1H, m, Ar-*H*), 7.61-7.56 (3H, m, Ar-*H*), 7.45 (1H, app td, *J* 7.5, 1.0, Ar-*H*), 7.33-7.30 (1H, m, Ar-*H*), 6.66 (1H, d, *J* 10.5, N*H*), 5.88 (1H, d, *J* 10.5, NHC*H*), 1.31 (9H, s, $CO_2C(CH_3)_3$); ^{13}C NMR (101 MHz, CD_2Cl_2): δ 153.3, 137.2, 134.2, 133.9, 133.2, 131.2, 131.0, 129.4, 129.2, 128.0, 125.8, 81.2, 72.4, 27.7; IR: ν_{max} (neat)/cm^{-1} 2975, 1986, 1704, 1511, 1447, 1276, 1144, 1027, 727; HRMS (FI$^+$) found 427.0262 [M]$^+$, $C_{18}H_{20}{}^{81}BrNO_4S^+$ requires 427.0277; mp: 163-164 °C (EtOAc/hexane).

tert-Butyl [2,2-dimethyl-1-(phenylsulfonyl)propyl]carbamate, 9l

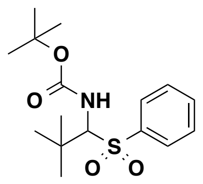

Prepared according to general procedure **B** using *tert*-butyl-carbamate (1.0 g, 8.5 mmol, 1.0 equiv.), sodium phenylsulfinate (2.8 g, 17 mmol, 2.0 equiv.) in H_2O/THF (2:1, v/v) (11.9 mL), trimethylacetaldehyde (1.8 mL, 17 mmol, 2.0 equiv.) and formic acid (3.2 mL, 8.5 mmol, 1.0 equiv.). The mixture was stirred for 72 hrs. Trituration afforded pure *α-amido sulfone* **9l** (2.05 g, 74%). Due to rapid decomposition HRMS data was not retrieved and the compound was used directly in propargylic amine synthesis.

^{1}H NMR (400 MHz, $CDCl_3$): δ 7.88 (2H, dd, *J* 7.5, 2.0, Ar-*H*), 6.95-6.92 (3H, m, Ar-*H*), 5.20 (1H, d, *J* 11.5, N*H*), 4.84 (1H, d, *J* 11.5, NHC*H*), 1.15 (9H, s, $CO_2C(CH_3)_3$), 1.13 (9H, s, $CHC(CH_3)_3$); ^{13}C NMR (101 MHz, $CDCl_3$): δ 153.9, 139.3, 133.4, 129.1, 128.8, 80.6, 36.7, 28.3, 27.4, 25.3; IR: ν_{max} (neat)/cm^{-1} 3294, 2982, 2030, 1692, 1552, 1516, 1366, 1177, 1048, 997; mp: 86-88 °C (EtOAc/hexane).

tert-Butyl [cyclopropyl(tosyl)methyl]carbamate, 9m

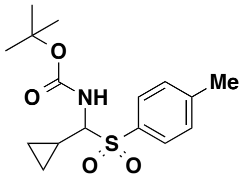

Prepared according to general procedure **B** using *tert*-butyl-carbamate (0.7 g, 6.0 mmol, 1.0 equiv.), sodium *p*-toluenesulfinate (1.18 g, 6.60 mmol, 1.1 equiv.) in H_2O/MeOH (2:1, v/v) (9.5 mL), cyclopropanecarboxaldehyde (0.5 mL, 3.97 mmol, 1.5 equiv.) and formic acid (0.89 mL, 23.5 mmol, 6.5 equiv.). The mixture was stirred for 16 hrs. Trituration afforded pure *α-amido sulfone* **9m** (0.69 g, 35%).

^{1}H NMR (400 MHz, $CDCl_3$): δ 7.82 (2H, d, *J* 8.0, Ar-*H*), 7.35 (2H, d, *J* 8.0, Ar-*H*), 5.13 (1H, d, *J* 10.0, N*H*), 4.37 (1H, app t, *J* 10.0, NHC*H*), 2.44 (3H, s, Ar-CH_3), 1.39-1.32 (1H, m, NHCHC*H*), 1.28 (9H, s, $CO_2C(CH_3)_3$), 0.86-0.79 (1H, m, NHCHCHCH_aH$_b$CH$_a$H$_b$), 0.73-0.66 (1H, m, NHCHCHCH$_a$$H_bCH_aH_b$), 0.63-0.59 (1H, m, NHCHCHCH$_a$H$_b$CH_aH$_b$), 0.47-0.41 (1H, m, NHCHCHCH$_a$H$_b$CH$_a$$H_b$); ^{13}C NMR (101 MHz, CD_2Cl_2): δ 153.8, 145.0, 134.5, 129.6, 129.2, 80.5, 74.3, 27.7, 21.3, 9.0, 4.2, 2.0; IR: ν_{max} (neat)/cm^{-1} 2978, 1716, 1597, 1516, 1368, 1163, 1140, 950; HRMS (FI$^+$) found 325.1274 [M]$^+$, $C_{16}H_{23}NO_4S^+$ requires 325.1348; mp: 126-127 °C (EtOAc/hexane).

4. Synthesis of novel propargylic amines

The following propargylic amine is commercially available and was used as supplied from Sigma-Aldrich Chemical Co. Ltd.:

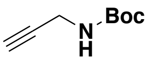

***N*-Boc-propargyl amine**
2a

The synthetic procedures and characterisation have previously been reported for the following propargylic amines:

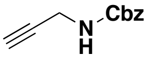

Benzyl prop-2-yn-1-ylcarbamate[11]
2s

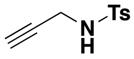

4-Methyl-*N*-(prop-2-yn-1-yl) benzenesulfonamide[12]
2t

***tert*-Butyl but-3-yn-2-ylcarbamate, 2b**

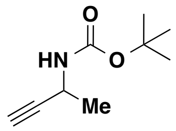

A solution of *n*-butyllithium (1.94 mL, 1.8 M in hexanes, 3.50 mmol, 2.1 equiv.) was added dropwise to a stirring solution of trimethylsilylacetylene (0.48 mL, 3.40 mmol, 2.0 equiv.) in THF (15 mL) at -15 °C. The reaction mixture was stirred at -15 °C for 30 mins. The temperature was lowered to -78 °C and a solution of α-amido sulfone **9a**[6] (0.50 g, 1.70 mmol, 1.0 equiv.) in THF (5 mL) was added. The mixture was warmed to room temperature over 1 hr. Quick addition of sat. $NH_4Cl_{(aq)}$ (5 mL) at -78 °C quenched the reaction and the mixture was allowed to warm to room temperature. The product was extracted with DCM (3 × 10 mL) and washed with brine (10 mL). The organic layer was dried over $MgSO_4$, filtered and concentrated under reduced pressure. With no further purification the crude propargyl amine (0.37 g, 1.52 mmol, 1.0 equiv.) was dissolved in MeOH (29 mL). Potassium carbonate (0.21 g, 1.52 mmol, 1.0 equiv.) was added to the solution at room temperature and stirred for 4 hrs. The reaction mixture was diluted with water (15 mL) and extracted with Et_2O (3 × 10 mL). The combined organic extracts were washed with brine (15 mL), dried over $MgSO_4$, filtered and concentrated *in vacuo*. The crude mixture was purified by flash column chromatography (20% EtOAc in petrol) to give title *propargylic amine* **2b** as a white solid (0.23 g, 80%).

^1H NMR (400 MHz, $CDCl_3$): δ 4.73 (1H, br s, N*H*), 4.51 (1H, app br s, NHC*H*), 2.28 (1H, d, *J* 2.5, C*H*CCHNH), 1.48 (9H, s, $CO_2C(CH_3)_3$), 1.43 (3H, d, *J* 7.0, $CHCCH(CH_3)$); ^{13}C NMR (101 MHz, $CDCl_3$): δ 154.6, 84.6, 79.9, 70.1, 38.2, 28.4, 22.5; IR: ν_{max} (neat)/cm^{-1} 2864, 1691, 1562, 1312, 1293, 1252, 989, 899, 722; LRMS (ESI+):

m/z 192 ([M+Na]$^+$, 100%); HRMS (ESI$^+$) found 192.09963 [M+Na]$^+$, $C_9H_{15}NO_2Na^+$ requires 192.09950; mp: 69-70 °C (EtOAc/petrol).

***tert*-Butyl (4,4-dimethylpent-1-yn-3-yl)carbamate, 2c**

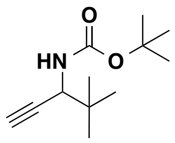

A solution of *n*-butyllithium (3.51 mL, 1.8 M in hexanes, 6.41 mmol, 2.1 equiv.) was added dropwise to a solution trimethylsilylacetylene (0.86 mL, 6.10 mmol, 2.0 equiv.) in THF (22 mL) at -15 °C. The reaction mixture was stirred at -15 °C for 30 mins. The temperature was lowered to -78 °C and a solution of α-amido sulfone **9l** (1.00 g, 3.05 mmol, 1.0 equiv.) in THF (10 mL) was added to the mixture. The mixture was stirred at -78 °C for 1 hr. Sat. $NH_4Cl_{(aq)}$ (10 mL) was added at -78 °C and the mixture was warmed to room temperature. The product was extracted with DCM (3 × 10 mL) and washed with brine (10 mL). The organic layer was dried over $MgSO_4$, filtered and concentrated under reduced pressure. With no further purification, the crude propargylic amine (0.80 g, 2.80 mmol, 1.0 equiv.) was dissolved in in MeOH (53 mL) and potassium carbonate (0.39 g, 2.80 mmol, 1.0 equiv.) was added, the reaction was stirred for 16 hrs at room temperature. Water (15 mL) was added to the reaction mixture and the product was extracted with Et_2O (3 × 10 mL) and washed with brine (15 mL). The organic layer was dried over $MgSO_4$, filtered and concentrated *in vacuo*. The crude mixture was purified by flash column chromatography (5-10% EtOAc in petrol) to give the title *propargylic amine* **2c** as a pale yellow solid (0.36 g, 61%).

^{1}H NMR (400 MHz, $CDCl_3$): δ 4.72 (1H, d, *J* 8.0, N*H*), 4.27 (1H, d, *J* 8.0, NHC*H*), 2.28 (1H, d, *J* 2.5, C*H*CCHNH), 1.48 (9H, s, $CO_2C(CH_3)_3$), 1.01 (9H, s, $NHCHC(CH_3)_3$); ^{13}C NMR (101 MHz, $CDCl_3$): δ 155.3, 82.5, 79.8, 71.7, 52.2, 35.4, 28.4, 25.8; IR: ν_{max} (neat)/cm^{-1} 3296, 2968, 1692, 1515, 1367, 1249, 1171, 1049, 998, 906, 731, 649; LRMS (ESI$^+$): *m/z* 234 ([M+Na]$^+$, 100%); HRMS (ESI$^+$) found 234.14666 [M+Na]$^+$, $C_{12}H_{21}NO_2Na^+$ requires 234.14645; mp: 64-65 °C (EtOAc/petrol). ^{1}H and ^{13}C NMR data is consistent with literature.[13]

General procedure C for the synthesis of terminal propargylic amines as exemplified by the preparation of *tert*-butyl (1-phenylprop-2-yn-1-yl)carbamate, 2d

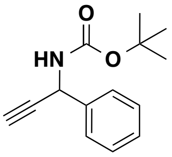

Following a procedure adapted from Shin *et al.*[13] A solution of ethynylmagnesium bromide (20.2 mL, 0.5 M in THF, 9.50 mmol, 2.2 equiv.) was added dropwise to a solution of *α*-amido sulfone **9b**[7] (1.5 g, 4.32 mmol, 1.0 equiv.) in THF (35 mL) at -78 °C. The reaction mixture was warmed to room temperature over 3 hrs. Upon completion the reaction was quenched with sat. $NH_4Cl_{(aq)}$ (mL) at -78 °C and was allowed to warm to room temperature. The product was extracted with DCM (3 × 10 mL) and washed with brine (10 mL). The combined organic layers were dried over $MgSO_4$, filtered and concentrated under reduced pressure. The crude product was purified by flash column chromatography (5-10% EtOAc in petrol) to give the title *propargylic amine* **2d** as a white solid (0.89 g, 89%).

^1H NMR (400 MHz, $CDCl_3$): δ 7.53 (2H, d, *J* 7.5, Ar-*H*), 7.40-7.33 (3H, m, Ar-*H*), 5.71 (1H, d, *J* 7.0, NHC*H*), 5.24 (1H, br s, N*H*), 2.52 (1H, d, *J* 2.5, C*H*CCHNH), 1.49 (9H, s, $CO_2C(CH_3)_3$); ^{13}C NMR (101 MHz, $CDCl_3$): δ 154.8, 138.8, 128.7, 128.1, 126.9, 82.2, 80.3, 73.0, 46.1, 28.4; IR: ν_{max} (neat)/cm^{-1} 3305, 2979, 1696, 1495, 1393, 1368, 1246, 1166, 1020, 911, 876, 698; LRMS (ESI$^+$): *m/z* 254 ([M+Na]$^+$, 100%); HRMS (ESI$^+$) found 254.11523 [M+Na]$^+$, $C_{14}H_{17}NO_2Na^+$ requires 254.11515; mp: 93-95 °C (EtOAc/petrol). ^1H and ^{13}C NMR data is consistent with literature.[13]

***tert*-Butyl [1-(4-methoxyphenyl)prop-2-yn-1-yl]carbamate, 2e**

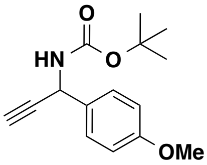

Prepared according to general procedure **C** using ethynylmagnesium bromide (23.4 mL, 0.5 M in THF, 11.7 mmol, 2.2 equiv.) and *α*-amido sulfone **9e**[9] (2.00g, 5.30 mmol, 1.0 equiv.) in THF (37 mL) for 1 hr. The crude product was purified by flash column chromatography (5-10% EtOAc in petrol) to give *propargylic amine* **2e** as an off-white solid (1.22 g, 88%).

^1H NMR (400 MHz, $CDCl_3$): δ 7.45 (2H, d, *J* 8.5, Ar-*H*), 6.91 (2H, d, *J* 8.5, Ar-*H*),

5.63 (1H, d, *J* 6.0, NHC*H*), 5.05 (1H, br s, N*H*), 3.83 (3H, s, OCH_3), 2.51 (1H, d, *J* 2.5, C*H*CCHNH), 1.48 (9H, s, $CO_2C(CH_3)_3$); ^{13}C NMR (101 MHz, $CDCl_3$): δ 159.4, 154.7, 130.9, 128.1, 114.0, 82.4, 80.2, 72.7, 55.3, 45.7, 28.4; IR: ν_{max} (neat)/cm^{-1} 2943, 2253, 1703, 1612, 1511, 1247, 1165, 1035, 909, 732; LRMS (ESI$^+$): *m/z* 284 ([M+Na]$^+$, 100%), 545 ([2M+Na]$^+$, 80%); HRMS (ESI$^+$) found 284.12571 [M+Na]$^+$, $C_{15}H_{19}O_3NNa^+$ requires 284.12571; mp: 94-96 °C (EtOAc/petrol). ^{1}H and ^{13}C NMR data is consistent with literature.[13]

***tert*-Butyl [1-(furan-2-yl)prop-2-yn-1-yl]carbamate, 2f**

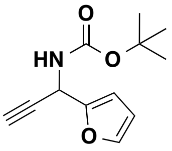

Prepared according to general procedure **C** using ethynylmagnesium bromide (6.94 mL, 0.5 M in THF, 3.26 mmol, 2.2 equiv.) and *α*-amido sulfone **9g**[9] (0.50 g, 1.48 mmol, 1.0 equiv.) in THF (6 mL) for 45 mins. The crude product was purified by flash column chromatography (5-15% EtOAc in petrol) to give *propargylic amine* **2f** as an off-white solid (0.14 g, 43%).

^{1}H NMR (400 MHz, $CDCl_3$): δ 7.39 (1H, dd, *J* 2.0, 1.0, Ar-*H*), 6.39 (1H, dd, *J* 3.0, 1.0, Ar-*H*), 6.34 (1H, dd, *J* 3.0, 2.0, Ar-*H*), 5.71 (1H, d, *J* 6.0, NHC*H*), 5.19 (1H, br s, N*H*), 2.44 (1H, d, *J* 2.5, C*H*CCHNH), 1.47 (9H, s, $CO_2C(CH_3)_3$); ^{13}C NMR (101 MHz, $CDCl_3$): δ 154.5, 150.8, 142.8, 110.4, 107.4, 80.5, 80.0, 71.9, 40.5, 28.3; IR: ν_{max} (neat)/cm^{-1} 3299, 2980, 1698, 1503, 1392, 1246, 1166, 1012, 862, 740, 656; LRMS (ESI$^+$): *m/z* 244 ([M+Na]$^+$, 100%); HRMS (ESI$^+$) found 244.09413 [M+Na]$^+$, $C_{12}H_{15}O_3NNa^+$ requires 244.09441; mp: 71-72 °C (EtOAc/petrol). ^{1}H and ^{13}C NMR data is consistent with literature.[13]

***tert*-Butyl [1-(4-cyanophenyl)prop-2-yn-1-yl]carbamate, 2g**

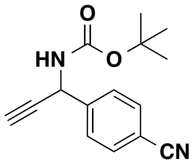

Prepared according to general procedure **C** using ethynylmagnesium bromide (12.6 mL, 0.5 M in THF, 5.90 mmol, 2.2 equiv.) and *α*-amido sulfone **9h**[9] (1.00 g, 2.68 mmol, 1.0 equiv.) in THF (21 mL) for 2 hrs. The crude product was purified by flash column chromatography (5% EtOAc in petrol) to give *propargylic amine* **2g** as a white solid (0.466 g, 68%).

^{1}H NMR (400 MHz, $CDCl_3$): δ 7.69-7.66 (4H, m, Ar-*H*), 5.73 (1H, d, *J* 6.0, NHC*H*), 5.15 (1H, br s, N*H*), 2.58 (1H, d, *J* 2.5, C*H*CCHNH), 1.48 (9H, s, $CO_2C(CH_3)_3$); ^{13}C

NMR (101 MHz, $CDCl_3$): δ 154.7, 144.1, 132.5, 127.6, 118.5, 112.0, 80.9, 80.7, 74.2, 45.9, 28.3; IR: ν_{max} (neat)/cm^{-1} 3301, 2980, 1694, 1609, 1504, 1392, 1164, 1020, 733; LRMS (ESI$^+$): *m/z* 257 ([M+H]$^+$, 10%), 279 ([M+Na]$^+$, 100%); HRMS (ESI$^+$) found 279.11003 [M+Na]$^+$, $C_{15}H_{16}O_2N_2Na^+$ requires 279.11040; mp: 106-108 °C (EtOAc/petrol). ^{1}H and ^{13}C NMR data is consistent with literature.[13]

***tert*-Butyl {1-[4-(trifluoromethyl)phenyl]prop-2-yn-1-yl}carbamate, 2h**

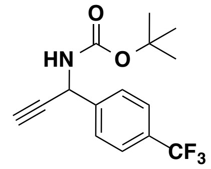

Prepared according to general procedure **C** using ethynylmagnesium bromide (11.3 mL, 0.5 M in THF, 5.30 mmol, 2.2 equiv.) and *α*-amido sulfone **9d**[8] (1.00 g, 2.41 mmol, 1.0 equiv.) in THF (19 mL) for 2 hrs. The crude product was purified by flash column chromatography (5-15% EtOAc in petrol) to give *propargylic amine* **2h** as a white solid (0.426 g, 59%).

^{1}H NMR (400 MHz, $CDCl_3$): δ 7.65-7.60 (4H, m, Ar-*H*), 5.75 (1H, app br s, NHC*H*), 5.44 (1H, br s, N*H*), 2.55 (1H, d, *J* 2.5, C*H*CCHNH), 1.46 (9H, s, $CO_2C(CH_3)_3$); ^{13}C NMR (101 MHz, $CDCl_3$): δ 154.8, 142.9, 130.3 (q, $^2J_{CF}$ 32.4), 127.2, 125.6 (q, $^3J_{CF}$ 3.5), 124.0 (q, $^1J_{CF}$ 272.3), 81.3, 80.6, 73.7, 45.8, 28.2; ^{19}F NMR (377 MHz, $CDCl_3$): -62.6; IR: ν_{max} (neat)/cm^{-1} 3310, 3100, 1689, 1620, 1496, 1386, 1124, 1018, 909, 732, 661; LRMS (ESI$^+$): *m/z* 322 ([M+Na]$^+$, 100%); HRMS (ESI$^+$) found 322.10200 [M+Na]$^+$, $C_{15}H_{16}O_2NF_3Na^+$ requires 322.10253; mp: 92-93 °C (EtOAc/petrol).

***tert*-Butyl [1-(thiophen-2-yl)prop-2-yn-1-yl]carbamate, 2i**

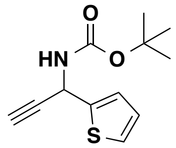

Prepared according to general procedure **C** using ethynylmagnesium bromide (6.60 mL, 0.5 M in THF, 3.10 mmol, 2.2 equiv.) and *α*-amido sulfone **9f**[9] (0.50 g, 1.41 mmol, 1.0 equiv.) in THF (11 mL) for 2 hrs. The crude product was purified by flash column chromatography (10% EtOAc in petrol) to give *propargylic amine* **2i** as a pale yellow solid (0.154 g, 46%).

^{1}H NMR (400 MHz, $CDCl_3$): δ 7.27 (1H, dd, *J* 5.0, 1.0, Ar-*H*), 7.19 (1H, dd, *J* = 3.5, 1.0, Ar-*H*), 6.97 (1H, dd, *J* 5.0, 3.5, Ar-*H*), 5.90 (1H, app br s, NHC*H*), 5.26 (1H, br s, N*H*), 2.53 (1H, d, *J* 2.5, C*H*CCHNH), 1.49 (9H, s, $CO_2C(CH_3)_3$); ^{13}C NMR (101 MHz, $CDCl_3$): δ 154.4, 142.6, 126.7, 125.7, 125.6, 81.5, 80.5, 72.5, 42.1, 28.3; IR: ν_{max}

(neat)/cm^{-1} 3304, 2979, 1695, 1488, 1367, 1161, 1018, 909, 864, 731; LRMS (ESI$^+$): *m/z* 260 ([M+Na]$^+$, 100%); HRMS (ESI$^+$) found 260.07123 [M+Na]$^+$, $C_{12}H_{15}O_2NSNa^+$ requires 260.07157; mp: 58-59 °C (EtOAc/petrol). ^{1}H and ^{13}C NMR data is consistent with literature.[13]

***tert*-Butyl [1-(2-bromophenyl)prop-2-yn-1-yl]carbamate, 2j**

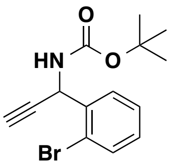

Prepared according to general procedure **C** using ethynylmagnesium bromide (12.1 mL, 0.5 M in THF, 5.70 mmol, 2.2 equiv.) and *α*-amido sulfone **9k** (1.1 g, 2.59 mmol, 1.0 equiv.) in THF (21 mL) for 2 hrs. The crude product was purified by flash column chromatography (5-20% EtOAc in petrol) to give *propargylic amine* **2j** as a white solid (0.578 g, 72%).

^{1}H NMR (400 MHz, $CDCl_3$): δ 7.64 (1H, dd, *J* 8.0, 1.5, Ar-*H*), 7.55 (1H, dd, *J* 8.0, 1.0, Ar-*H*), 7.32 (1H, app td, *J* 8.0, 1.0, Ar-*H*), 7.16 (1H, app td, *J* 8.0, 1.5, Ar-*H*), 5.86 (1H, app br s, NHC*H*), 5.37 (1H, br s, N*H*), 2.48 (1H, d, *J* 2.5, C*H*CCHNH), 1.43 (9H, s, $CO_2C(CH_3)_3$); ^{13}C NMR (101 MHz, $CDCl_3$): δ 154.3, 137.8, 133.3, 129.7, 128.7, 127.8, 123.1, 81.4, 80.3, 73.2, 46.6, 28.3; IR: ν_{max} (neat)/cm^{-1} 3299, 2978, 1696, 1489, 1366, 1159, 1024, 753, 641; LRMS (ESI$^+$): *m/z* 332 ([^{79}Br] [M+Na]$^+$, 95%), 334 ([^{81}Br] [M+Na]$^+$, 100%); HRMS (ESI$^+$) found 332.02503 [[^{79}Br] M+Na]$^+$, 334.02289 [[^{81}Br] M+Na]$^+$, $C_{14}H_{16}O_2N^{79}BrNa^+$ requires 332.02566, $C_{14}H_{16}O_2N^{81}BrNa^+$ requires 334.02362; mp: 96-97 °C (EtOAc/petrol).

***tert*-Butyl [1-(4-bromophenyl)prop-2-yn-1-yl]carbamate, 2k**

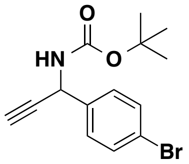

Prepared according to general procedure **C** using ethynylmagnesium bromide (8.80 mL, 0.5 M in THF, 4.14 mmol, 2.2 equiv.) and *α*-amido sulfone **9c**[8] (0.80 g, 1.88 mmol, 1.0 equiv.) in THF (15 mL) for 2 hrs. The crude product was purified by flash column chromatography (5-10% EtOAc in petrol) to give *propargylic amine* **2k** as a white solid (0.29 g, 50%).

^{1}H NMR (400 MHz, $CDCl_3$): δ 7.52-7.49 (2H, m, Ar-*H*), 7.43-7.38 (2H, m, Ar-*H*), 5.64 (1H, app br s, NHC*H*), 5.13 (1H, br s, N*H*), 2.53 (1H, d, *J* 2.5, C*H*CCHNH), 1.48 (9H, s, $CO_2C(CH_3)_3$); ^{13}C NMR (101 MHz, $CDCl_3$): δ 154.7, 138.0, 131.8, 128.6,

122.1, 81.5, 80.5, 73.4, 45.7, 28.3; IR: ν_{max} (neat)/cm^{-1} 3300, 2978, 1690, 1487, 1367, 1161, 1011, 876, 646; LRMS (ESI$^+$): *m/z* 332 ([^{79}Br] [M+Na]$^+$, 100%), 334 ([^{81}Br] [M+Na]$^+$, 95%); HRMS (ESI$^+$) found 332.02490 [[^{79}Br] M+Na]$^+$, 334.02277 [[^{81}Br] M+Na]$^+$, $C_{14}H_{16}O_2N^{79}BrNa^+$ requires 332.02566, $C_{14}H_{16}O_2N^{81}BrNa$ requires 334.02362; mp: 99-100 °C (EtOAc/petrol).

Methyl 4-{1-[(*tert*-butoxycarbonyl)amino]prop-2-yn-1-yl}benzoate, 2l

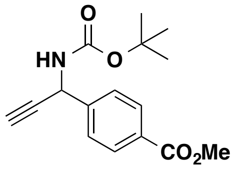

Prepared according to general procedure **C** using ethynylmagnesium bromide (13.9 mL, 0.5 M in THF, 6.51 mmol, 2.2 equiv.) and *α*-amido sulfone **9i**[10] (1.20 g, 2.96 mmol, 1.0 equiv.) in THF (24 mL) for 2 hrs. The crude product was purified by flash column chromatography (5% EtOAc in petrol) to give *propargylic amine* **2l** as a white solid (0.624 g, 73%).

^{1}H NMR (400 MHz, $CDCl_3$): δ 8.05-8.02 (2H, m, Ar-*H*), 7.58 (2H, d, *J* 8.0, Ar-*H*), 5.73 (1H, app br s, NHC*H*), 5.25 (1H, br s, N*H*), 3.92 (3H, s, CO_2CH_3), 2.54 (1H, d, *J* 2.5, C*H*CCHNH), 1.47 (9H, s, $CO_2C(CH_3)_3$); ^{13}C NMR (101 MHz, $CDCl_3$): 166.7, 154.7, 143.8, 129.9, 129.8, 126.8, 81.4, 80.6, 73.6, 52.2, 45.9, 28.3; IR: ν_{max} (neat)/cm^{-1} 3304, 2984, 1704, 1612, 1509, 1280, 1162, 1019, 910, 732; LRMS (ESI$^+$): *m/z* 312 ([M+Na]$^+$, 100%); HRMS (ESI$^+$) found 312.11994 [M+Na]$^+$, $C_{16}H_{19}O_4NNa^+$ requires 312.12063; mp: 110-111 °C (EtOAc/petrol). ^{1}H and ^{13}C NMR data is consistent with literature.[13]

***tert*-Butyl [1-(6-methoxypyridin-2-yl)prop-2-yn-1-yl]carbamate, 2m**

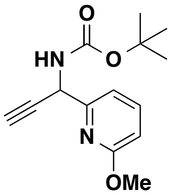

Prepared according to general procedure **C** using ethynylmagnesium bromide (17.8 mL, 0.5 M in THF, 8.71 mmol, 2.2 equiv.) and *α*-amido sulfone **9j** (1.50 g, 3.96 mmol, 1.0 equiv.) in THF (32 mL) for 2 hrs. The crude product was purified by flash column chromatography (5-10% EtOAc in petrol) to give *propargylic amine* **2m** as a light brown solid (0.522 g, 50%).

^{1}H NMR (400 MHz, $CDCl_3$): δ 7.60 (1H, dd, *J* 8.0, 7.5, Ar-*H*), 7.02 (1H, d, *J* 7.5, Ar-*H*), 6.71 (1H, d, *J* 8.0, Ar-*H*), 5.67 (1H, app br s, NHC*H*), 5.58 (1H, br s, N*H*), 3.98

(3H, s, OCH_3), 2.42 (1H, d, *J* 2.5, C*H*CCHNH), 1.50 (9H, s, $CO_2C(CH_3)_3$); ^{13}C NMR (101 MHz, $CDCl_3$): 163.9, 154.9, 154.0, 139.5, 113.9, 110.3, 82.3, 80.2, 72.0, 53.5, 47.0, 28.4; IR: ν_{max} (neat)/cm^{-1} 3299, 2931, 1701, 1493, 1167, 908, 731; LRMS (ESI$^+$): *m/z* 263 ([M+H]$^+$, 100%), 285 ([M + Na]$^+$, 35%); HRMS (ESI$^+$) found 285.12029 [M+Na]$^+$, $C_{14}H_{18}O_3N_2Na^+$ requires 285.12096; mp: 42-43 °C (EtOAc/petrol).

tert-Butyl (1-cyclopropylprop-2-yn-1-yl)carbamate, 2n

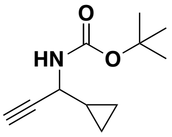

Prepared according to general procedure **C** using ethynylmagnesium bromide (5.05 mL, 0.49 M in THF, 2.02 mmol, 2.2 equiv.) and *α*-amido sulfone **9m** (0.30 g, 0.92 mmol, 1.0 equiv.) in THF (7.5 mL) for 2 hrs. The crude product was purified by flash column chromatography (10-20% Et_2O in toluene) to give *propargylic amine* as a white solid (154 mg, 86%).

^{1}H NMR (400 MHz, $CDCl_3$): δ 4.93 (1H, br s, N*H*), 4.39 (1H, app br s, NHC*H*), 2.21 (1H, d, *J* 2.5, C*H*CCHNH), 1.44 (9H, s, $CO_2C(CH_3)_3$), 1.15-1.07 (1H, m, NHCHC*H*), 0.47-0.43 (4H, m, NHCHCH(CH_2)$_2$); ^{13}C NMR (101 MHz, $CDCl_3$): 154.8, 81.2, 71.1, 45.5, 28.3, 15.1, 2.7, 1.6; IR: ν_{max} (neat)/cm^{-1} 3008, 2978, 1691, 1492, 1366, 1243, 1163, 1045, 1021; LRMS (ESI$^+$): *m/z* 218 ([M+Na]$^+$, 100%); HRMS (ESI$^+$) found 218.11496 [M+Na]$^+$, $C_{11}H_{17}O_2NNa^+$ requires 218.11515; mp: 64-65 °C (DCM/petrol).

General procedure D for the synthesis of internal propargylic amines as exemplified by the synthesis of _tert_-butyl dec-3-yn-2-ylcarbamate, 2o

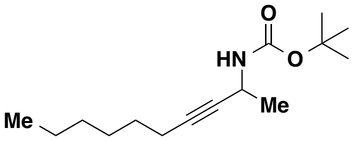

A procedure adapted from Shin *et al.*[13] A solution of *n*-butyllithium (1.37 mL, 2.5 M in hexanes, 3.43 mmol, 2.05 equiv.) was added dropwise to a solution of 1-octyne (0.49 mL, 3.34 mmol, 2.0 equiv.) in THF (15 mL) at -15 °C. The reaction mixture was stirred at -15 °C for 30 mins. The temperature was lowered to -78 °C and a solution of *α*-amido sulfone **9a**[6] (0.50 g, 1.67 mmol, 1.0 equiv.) in THF (5 mL) was added dropwise. The mixture was allowed to warm to room temperature for 2 hrs. Upon completion, the reaction was quenched by quick addition of sat. $NH_4Cl_{(aq)}$ at -78 °C and then warmed to room temperature. The product was extracted with DCM (3 × 10 mL) and washed with brine (10 mL).

The organic layer was dried over $MgSO_4$, filtered and concentrated under reduced pressure. The crude product was purified by flash column chromatography (10-20% EtOAc in petrol) to afford title *propargylic amine* **2o** as a colourless oil (297 mg, 70%).

^1H NMR (400 MHz, $CDCl_3$): δ 4.69 (1H, br s, N*H*), 4.47 (1H, app br s, NHC*H*), 2.17 (2H, td, *J* 7.0, 2.0, $CH_3(CH_2)_4CH_2$), 1.53-1.49 (2H, m, $CH_3(CH_2)_3CH_2$), 1.47 (9H, s, $CO_2C(CH_3)_3$), 1.39-1.27 (9H, m, $CH_3(CH_2)_3$ and $NHCHCH_3$), 0.91 (3H, t, *J* 7.0, $CH_3(CH_2)_5$); ^{13}C NMR (101 MHz, $CDCl_3$): 154.7, 82.6, 80.7, 31.3, 28.9, 28.6, 28.5, 28.4, 28.0, 23.3, 22.5, 18.6, 14.0; IR: ν_{max} (neat)/cm^{-1} 2932, 2860, 1704, 1495, 1367, 1163, 1053, 908; LRMS (ESI$^+$): *m/z* 276 ([M + Na]$^+$, 100%); HRMS (ESI$^+$) found 276.1934 [M+Na]$^+$, $C_{15}H_{27}O_2NNa^+$ requires 276.1940.

***tert*-Butyl (1-phenylnon-2-yn-1-yl)carbamate, 2p**

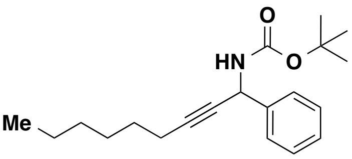

Prepared according to general procedure **D** using *n*-butyllithium (4.70 mL, 2.5 M in hexanes, 11.8 mmol, 2.05 equiv.), 1-octyne (1.7 mL, 11.5 mmol, 2.0 equiv.) in THF (25 mL) and *a*-amido sulfone **9b**[7] (1.5 g, 5.74 mmol, 1.0 equiv.) in THF (50 mL). The mixture was stirred for 30 mins. The crude product was purified by flash column chromatography (10-30% Et_2O in petrol) to give *propargylic amine* **2p** as a colourless oil (1.44 g, 78%).

^1H NMR (400 MHz, $CDCl_3$): δ 7.52 (2H, d, *J* 7.5, Ar-*H*), 7.43-7.35 (2H, m, Ar-*H*), 7.34-7.29 (1H, m, Ar-*H*), 5.67 (1H, app br s, NHC*H*), 5.03 (1H, br s, N*H*), 2.27 (2H, td, *J* 7.0, 2.0, $CH_3(CH_2)_4CH_2$), 1.59-1.52 (2H, m, $CH_3(CH_2)_3CH_2$), 1.50 (9H, s, $CO_2C(CH_3)_3$), 1.46-1.39 (2H, m, $CH_3(CH_2)_2CH_2$), 1.37-1.27 (4H, m, $CH_3(CH_2)_2$), 0.91 (3H, t, *J* 10.0, $CH_3(CH_2)_5$); ^{13}C NMR (101 MHz, $CDCl_3$): δ 154.8, 140.3, 128.5, 127.7, 126.8, 85.5, 79.9, 78.3, 46.5, 31.3, 29.4, 28.6, 28.4, 22.5, 18.8, 14.0; IR: ν_{max} (neat)/cm^{-1} 2931, 1703, 1493, 1367, 1243, 1167, 1019, 907, 730; LRMS (ESI$^+$): *m/z* 338 ([M+Na]$^+$, 100%), 653 ([2M+Na]$^+$, 50%); HRMS (ESI$^+$) found 338.20918 [M+Na]$^+$, $C_{20}H_{29}NO_2Na^+$ requires 338.20905.

***tert*-Butyl (1-cyclopropyldec-2-yn-1-yl)carbamate, 2q**

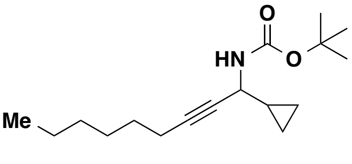

Prepared according to general procedure **D** using ethylmagnesium bromide (2.2

mL, 1.0 M in THF, 2.2 mmol, 2.05 equiv.), 1-octyne (0.32 mL, 2.16 mmol, 2.0 equiv.) in THF (3.8 mL), *α*-amido sulfone **9m** (0.35 g, 1.08 mmol, 1.0 equiv.) in THF (7.5 mL). The mixture was stirred for 4 hrs. The crude product was purified by flash column chromatography (5-10% EtOAc in petrol) to give *propargylic amine* **2q** as a colourless oil (0.22 g, 73%).

^{1}H NMR (400 MHz, $CDCl_3$): δ 4.77 (1H, app br s, NHC*H*), 4.48 (1H, br s, N*H*), 2.16 (2H, td, *J* 7.0, 2.0, $CH_3(CH_2)_4C\mathit{H_2}$), 1.64-1.56 (2H, m, $CH_3(CH_2)_3C\mathit{H_2}$), 1.48 (9H, s, $CO_2C(C\mathit{H_3})_3$), 1.39-1.28 (6H, m, $CH_3(C\mathit{H_2})_3$), 1.17-1.09 (1H, m, NHCHC*H*), 0.91 (3H, t, *J* 7.0, $C\mathit{H_3}(CH_2)_5$), 0.49-0.42 (4H, m, $NHCHCH(C\mathit{H_2})_2$); ^{13}C NMR (101 MHz, $CDCl_3$): 154.9, 83.7, 79.6, 69.8, 31.3, 28.7, 28.4, 22.5, 18.6, 15.4, 14.0, 10.4, 2.6, 1.2; IR: ν_{max} (neat)/cm^{-1} 2928, 2861, 1710, 1494, 1320, 1241, 1017, 912, 732; LRMS (ESI$^+$): *m/z* 302 ([M+Na]$^+$, 100%); HRMS (ESI$^+$) found 302.20866 [M+Na]$^+$, $C_{17}H_{29}O_2NNa^+$ requires 302.20905.

tert-Butyl [4-(benzyloxy)-1-cyclopropylbut-2-yn-1-yl]carbamate, 2r

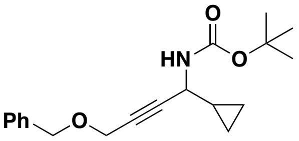

Prepared according to general procedure **D** using *n*-butyllithium (0.56 mL, 2.25 M in hexanes, 1.25 mmol, 2.05 equiv.), [(prop-2-yn-1-yloxy)methyl]benzene (0.18 mg, 1.22 mmol, 2.0 equiv.) in THF (3.0 mL) and *α*-amido sulfone **9m** (0.20 g, 0.61 mmol, 1.0 equiv.) in THF (4.0 mL). The mixture was stirred for 30 mins. The crude product was purified by flash column chromatography (5-10% EtOAc in petrol) to give *propargylic amine*, **2r** as a colourless oil (0.134 g, 73%).

^{1}H NMR (400 MHz, $CDCl_3$): δ 7.39-7.36 (4H, m, Ar-*H*), 7.35-7.33 (1H, m, Ar-*H*), 4.84 (1H, br s, N*H*), 4.60 (2H, s, $ArC\mathit{H_2}O$), 4.50 (1H, app br s, NHC*H*), 4.19 (2H, d, *J* 2.0, $OC\mathit{H_2}CCCHNH$), 1.49 (9H, s, $CO_2C(C\mathit{H_3})_3$), 1.19-1.14 (1H, m, NHCHC*H*), 0.54-0.47 (4H, m, $NHCHCH(C\mathit{H_2})_2$); ^{13}C NMR (101 MHz, $CDCl_3$): δ 154.8, 137.4, 128.4, 128.2, 127.9, 84.0, 78.8, 71.5, 57.3, 28.4, 15.4, 2.8, 1.7; IR: ν_{max} (neat)/cm^{-1} 3338, 2979, 1701, 1495, 1366, 1159, 1069, 909, 732; LRMS (ESI$^+$): *m/z* 338 ([M+Na]$^+$, 100%); HRMS (ESI$^+$) found 338.17132 [M+Na]$^+$, $C_{19}H_{25}O_3NNa^+$ requires 338.17266.

5. Synthesis of novel allylic amines

The synthetic procedures and characterisation have previously been reported for the following allylic amine:

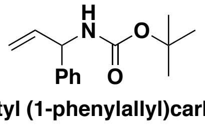

tert-Butyl (1-phenylallyl)carbamate
10a[7]

General procedure E for the synthesis of terminal allylic amines as exemplified by the preparation of *tert*-butyl but-3-en-2-ylcarbamate, 10b:

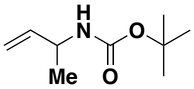

Following a procedure adapted from Petrini *et al.*[7] A solution of vinylmagnesium bromide (6.16 mL, 1.0 M in THF, 6.16 mmol, 2.2 equiv.) was added dropwise to a solution of *α*-amido sulfone **9a**[6] (0.8 g, 2.8 mmol, 1.0 equiv.) in THF (26.5 mL) at -20 °C. The reaction mixture was stirred at -20 °C for 30 min and then warmed to room temperature over 30 min. Upon completion the reaction was quenched with sat. $NH_4Cl_{(aq)}$ (mL) at -20 °C and was allowed to warm to room temperature. The product was extracted with Et_2O (3 × 10 mL) and washed with brine (10 mL). The combined organic layers were dried over $MgSO_4$, filtered and concentrated under reduced pressure. The crude product was purified by flash column chromatography (10% Et_2O in petrol) to give title *allylic amine* **10b** as a colourless oil (80 mg, 17%).

^{1}H NMR (400 MHz, $CDCl_3$): δ 5.84 (1H, ddd, *J* 17.0, 10.5, 5.0, CH_2C*H*CHNH), 5.19-5.06 (2H, m, C*H*$_2$CHCHNH), 4.48 (1H, br s, N*H*), 4.24 (1H, app br s, NHC*H*), 1.47 (9H, s, CO_2C(C*H*$_3$)$_3$), 1.23 (3H, d, *J* 7.0, NHCHC*H*$_3$); ^{13}C NMR (101 MHz, $CDCl_3$): δ 140.2, 116.4, 113.6, 79.5, 48.2, 28.4, 20.7; IR: ν_{max} (neat)/cm^{-1} 2979, 1701, 1497, 1392, 1170, 910; HRMS (FI$^+$) found 171.1258 [M]$^+$, $C_9H_{17}NO_2^+$ requires 171.1259.

tert-Butyl [1-(thiophen-2-yl)allyl]carbamate, 10c

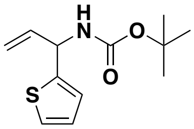

Prepared according to general procedure **E** using vinylmagnesium bromide (2.5 mL, 1.0 M in THF, 2.5 mmol, 2.2 equiv.), *α*-amido sulfone **9f**[9] (0.40 g, 1.13 mmol, 1.0 equiv.) in THF (11 mL). The crude product was purified by flash column chromatography (5-10% Et_2O in petrol) to give *allylic amine* **10c** as a white solid (175 mg, 65%).

^{1}H NMR (400 MHz, $CDCl_3$): δ 7.28 (1H, ddd, *J* 5.0, 3.0, 2.5, Ar-*H*), 7.13-7.11 (1H, m, Ar-*H*), 7.02-7.00 (1H, m, Ar-*H*), 6.02 (1H, ddd, *J* 17.0, 10.5, 6.0, CH_2C*H*CHNH), 5.36 (1H, app br s, NHC*H*), 5.27-5.20 (2H, m, C*H*$_2$CHCHNH), 5.00 (1H, br s, N*H*), 1.46 (9H, s, CO_2C(C*H*$_3$)$_3$); ^{13}C NMR (101 MHz, $CDCl_3$): δ 155.0, 142.2, 137.6, 126.7, 126.2, 121.6, 115.3, 79.7, 52.5, 28.4; IR: ν_{max} (neat)/cm^{-1} 2971, 1712, 1520, 1416, 1521, 1244, 1077, 794; LRMS (ESI$^+$): *m/z* 262 ([M+Na]$^+$, 100%); HRMS (ESI$^+$) found 262.08729 [M+Na]$^+$, $C_{12}H_{17}O_2NSNa^+$ requires 262.08777; mp: 64 °C

DCM/hexane).

tert-Butyl [1-(4-methoxyphenyl)allyl]carbamate, 10d

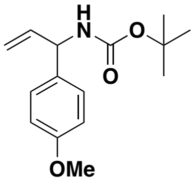

Prepared according to general procedure **E** using vinylmagnesium bromide (1.12 mL, 1.0 M in THF, 1.12 mmol, 2.2 equiv.), *α*-amido sulfone **9e**[9] (0.20 g, 0.53 mmol, 1.0 equiv.) in THF (5 mL). The crude product was purified by flash column chromatography (15-30% Et_2O in petrol) to give *allylic amine* **10d** as a white solid (112 mg, 80%).

^1H NMR (400 MHz, $CDCl_3$): δ 7.24-7.22 (2H, m, Ar-*H*), 6.90-6.87 (2H, m, Ar-*H*), 6.00 (1H, ddd, *J* 17.0, 10.0, 5.5, $CH_2C\mathit{H}CHNH$), 5.28-5.21 (3H, m, $C\mathit{H_2}CHCHNH$ and $NHC\mathit{H}$), 4.94 (1H, br s, N*H*), 3.80 (3H, s, $OC\mathit{H_3}$), 1.46 (9H, s, $CO_2C(C\mathit{H_3})_3$); ^{13}C NMR (101 MHz, $CDCl_3$): δ 158.9, 155.0, 138.2, 133.2, 128.2, 115.1, 114.0, 79.5, 56.0, 55.2, 28.4; IR: ν_{max} (neat)/cm^{-1} 2979, 1715, 1612, 1366, 1245, 1108, 1036; LRMS (ESI^+): *m/z* 286 ($[M+Na]^+$, 100%); HRMS (ESI^+) found 286.12130 $[M+Na]^+$, $C_{15}H_{21}O_3NNa^+$ requires 286.14136; mp: 74-75 °C (DCM/petrol). ^1H and ^{13}C data consistent with literature.[14]

tert-Butyl {1-[4-(trifluoromethyl)phenyl]allyl}carbamate, 10e

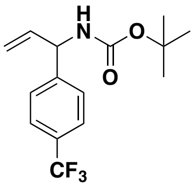

Prepared according to general procedure **E** using vinylmagnesium bromide (1.32 mL, 1.0 M in THF, 1.32 mmol, 2.2 equiv.), *α*-amido sulfone **9d**[8] (0.25 g, 0.60 mmol, 1.0 equiv.) in THF (5.7 mL). The crude product was purified by flash column chromatography (15-20% Et_2O in petrol) to give *allylic amine* **10e** as a white solid (163 mg, 90%).

^1H NMR (400 MHz, $CDCl_3$): δ 7.61 (2H, d, *J* 8.0, Ar-*H*), 7.43 (2H, d, *J* 8.0, Ar-*H*), 5.98 (1H, ddd, *J* 17.0, 10.5, 6.0, $CH_2C\mathit{H}CHNH$), 5.36 (1H, br s, N*H*), 5.27 (1H, d, *J* 10.5, $C\mathit{H_a}H_bCHCHNH$), 5.22 (1H, d, *J* 17.0, $CH_a\mathit{H_b}CHCHNH$), 5.10 (1H, app br d, *J* 7.0, $NHC\mathit{H}$), 1.45 (9H, s, $CO_2C(C\mathit{H_3})_3$); ^{13}C NMR (101 MHz, $CDCl_3$): δ 155.0, 145.2, 137.1, 129.6 (q, $^2J_{CF}$ 32.5), 127.3, 125.5 (q, $^3J_{CF}$ 3.5), 124.1 (q, $^1J_{CF}$ 275.0) 116.6, 80.0, 56.4, 28.3; ^{19}F NMR (377 MHz, $CDCl_3$): δ -62.5; IR: ν_{max} (neat)/cm^{-1} 2982,

1700, 1619, 1492, 1367, 1297, 1107, 1067, 990; LRMS (ESI$^+$): *m/z* 324 ([M+Na]$^+$, 100%); HRMS (ESI$^+$) found 324.1180 [M+Na]$^+$, $C_{15}H_{18}O_2NF_3Na^+$ requires 324.11818; mp: 80-81 °C (DCM/petrol). ^{1}H and ^{13}C data consistent with literature.[14]

***tert*-Butyl (1-cyclopropylallyl)carbamate, 10f**

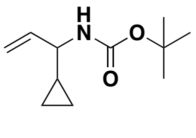

Prepared according to general procedure **E** using vinylmagnesium bromide (3.50 mL, 1.0 M in THF, 3.52 mmol, 2.2 equiv.), *α*-amido sulfone **9m** (0.50 g, 1.60 mmol, 1.0 equiv.) in THF (15 mL). The crude product was purified by flash column chromatography (10% Et_2O in petrol) to give *allylic amine* **10f** as a colourless oil (160 mg, 51%).

^{1}H NMR (400 MHz, $CDCl_3$): δ 5.80 (1H, ddd, *J* 17.0, 10.5, 5.5, CH_2C*H*CHNH), 5.21-5.06 (2H, m, C*H*$_2$CHCHNH), 4.70 (1H, app br s, NHC*H*), 3.58 (1H, br s, N*H*), 1.43 (9H, s, CO_2C(C*H*$_3$)$_3$), 0.89-0.81 (1H, m, NHCHC*H*(CH_2)$_2$), 0.53-0.45 (2H, m, NHCHCH(C*H*$_2$$CH_2$)), 0.37 (1H, dt, *J* 9.0, 4.5, NHCHCH(CH_2C*H*$_a$H$_b$)), 0.27-0.22 (1H, m, NHCHCH(CH_2CH_a*H*$_b$)); ^{13}C NMR (101 MHz, $CDCl_3$): δ 155.3, 137.9, 114.4, 79.2, 56.3, 28.4, 15.6, 2.7; IR: ν_{max} (neat)/cm^{-1} 2979, 1688, 1497, 1390, 1243, 1164, 1018, 989; LRMS (ESI$^+$): *m/z* 220 ([M+Na]$^+$, 100%); HRMS (ESI$^+$) found 220.13142 [M+Na]$^+$, $C_{11}H_{19}O_2NNa^+$ requires 220.13080;

6. Hydroacylation products from aldehyde and propargylic amine coupling

General procedure F for the synthesis of linear *γ*-amino enones *via* rhodium-catalysed hydroacylation of propargylic amines as exemplified by the synthesis of *tert*-butyl (*E*)-[4-(2-(methylthio)phenyl)-4-oxobut-2-en-1-yl]carbamate, 3a-Boc

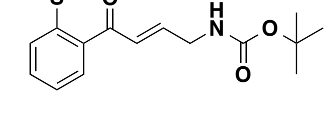

An oven-dried microwave vial was charged with [Rh(nbd)$_2$BF$_4$] (1.1 mg, 2 mol%) and PNP(Cy) (1.3 mg, 2 mol%), once under an inert atmosphere, they were dissolved in acetone (1.0 mL). Hydrogen gas was bubbled through the solution at room temperature for 1-2 mins in order to generate the active catalyst species (colour change from red to pale yellow). The hydrogen gas was purged using

nitrogen gas, and this was bubbled through the catalyst to dryness. The dry catalyst was dissolved in acetone (75 μL, 1.0 M with respect to aldehyde minus starting materials) and this was transferred to a nitrogen-filled microwave vial containing 2-(methylthio)benzaldehyde **1a** (19 μL, 0.15 mmol, 1.0 equiv.) and *N*-Boc-propargylamine **2a** (30.2 mg, 0.195 mmol, 1.3 equiv.). The reaction mixture, once homogenous (on occasion, sonication was required), was then stirred at room temperature. The reaction was monitored by TLC until complete. After 20 mins, the reaction mixture was filtered through a plug of silica (acetone, 10 mL) and the solvent removed *in vacuo* to obtain the crude product. The crude product was analysed by ^{1}H NMR spectroscopy to measure the ratio of the linear to branched products (17:1, linear **3a-Boc**: branched**3a'-Boc**). The regioisomers were then separated by flash column chromatography (5% EtOAc in petrol) to afford title linear *γ-amino enone* **3a-Boc** as a pale yellow oil (39.7 mg, 86%).

^{1}H NMR (400 MHz, $CDCl_3$): δ 7.63 (1H, d, *J* 7.5, Ar-*H*), 7.47 (1H, app t, *J* 7.5, Ar-*H*), 7.37 (1H, d, *J* 7.5, Ar-*H*), 7.21 (1H, app t, *J* 7.5, Ar-*H*), 6.86-6.76 (2H, m, COC*H*C*H*), 4.82 (1H, br s, N*H*), 4.02 (2H, app br s, NHC*H*$_2$), 2.46 (3H, s, SC*H*$_3$), 1.46 (9H, s, $CO_2C(CH_3)_3$); ^{13}C NMR (101 MHz, $CDCl_3$): δ 182.1, 155.6, 145.6, 140.8, 136.3, 131.6, 129.7, 127.9, 126.1, 124.0, 79.9, 41.8, 28.4, 16.4; IR: ν_{max} (neat)/cm^{-1} 3370, 2977, 1710, 1514, 1434, 1366, 1250, 1167, 1026; LRMS (ESI$^+$): *m/z* 308 ([M+H]$^+$, 50%), 330 ([M+Na]$^+$, 100%); HRMS (ESI$^+$) found 330.1132 [M+Na]$^+$, $C_{16}H_{21}NO_3SNa^+$ requires 330.1134.

***tert*-Butyl {2-[2-(methylthio)benzoyl]allyl}carbamate, 3a'-Boc**

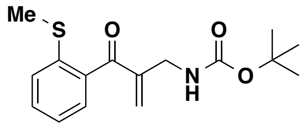

Prepared following a procedure adapted from Willis *et. al.*,[2] An oven-dried microwave vial was charged with [Rh(nbd)$_2$BF$_4$] (11.2 mg, 10 mol%) and dppe(*orhto*-^{i}Pr) (17.0 mg, 10 mol%), once under an inert atmosphere, they were dissolved in acetone (1.0 mL). Hydrogen gas was bubbled through the solution at room temperature for 1-2 minutes in order to generate the active catalyst species. The hydrogen gas was purged using nitrogen gas, and this was bubbled through the catalyst to dryness. The dry catalyst was dissolved in acetone (0.5 mL) and this was transferred to a nitrogen-filled microwave vial containing 2-(methylthio)benzaldehyde **1a** (39 μL, 0.30 mmol, 1.0 equiv.) and *N*-Boc-propargylamine **2a** (93.1 mg, 0.60 mmol, 2.0 equiv.). The reaction was monitored by TLC until complete. After 6 hrs the vial was opened to air and filtered through a plug of silica (acetone, 15 mL) and the solvent removed *in vacuo*. The crude product was purified by flash column chromatography (5-10%

EtOAc in petrol) to afford title branched *γ-amino enone* **3a'-Boc** as a pale yellow oil (78.2 mg, 85%).

^{1}H NMR (400 MHz, $CDCl_3$): δ 7.45 (1H, app td, *J* 7.5, 1.5, Ar-*H*), 7.40-7.38 (2H, m, Ar-*H*), 7.22 (1H, app td, *J* 7.5, 1.5, Ar-*H*), 6.13 (1H, app s, $NHCH_2CCH_aH_bCO$), 5.69 (1H, app s, $NHCH_2CCH_aH_bCO$), 5.10 (1H, br s, N*H*), 4.14 (2H, d, *J* 6.0, $NHCH_2$), 2.45 (3H, s, SCH_3), 1.45 (9H, s, $CO_2C(CH_3)_3$); ^{13}C NMR (101 MHz, $CDCl_3$): δ 198.0, 155.6, 145.4, 138.4, 137.9, 131.0, 129.3, 129.2, 127.5, 124.6, 79.4, 41.5, 28.4, 17.0; IR: ν_{max} (neat)/cm^{-1} 3361, 2978, 1695, 1655, 1585, 1513, 1434, 1392, 1367, 1310, 1281, 1251, 1169, 1029; LRMS (ESI$^+$): *m/z* 308 ([M+H]$^+$, 60%), 330 ([M+Na]$^+$, 100%); HRMS (ESI$^+$) found 330.1135 [M+Na]$^+$, $C_{16}H_{21}NO_3SNa^+$ requires 330.1134.

Benzyl (*E*)-{4-[2-(methylthio)phenyl]-4-oxobut-2-en-1-yl}carbamate, 3a-Cbz

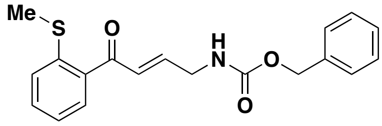

Prepared according to general procedure **F** using 2-(methylthio)benzaldehyde **1a** (19 *μ*L, 0.15 mmol, 1.0 equiv.), propargylic amine (benzyl prop-2-yn-1-ylcarbamate **2s**) (42.6 mg, 0.225 mmol, 1.3 equiv.), [Rh(nbd$_2$)]BF_4 (2.8 mg, 5 mol%) and PNP(Cy) (3.2 mg, 5 mol%) in acetone (88 *μ*L, 1 M). After stirring for 30 min at room temperature the reaction gave a crude mixture of regioisomers (12:1, linear: branched). The compounds were separated by flash column chromatography (5-20% EtOAc in petrol) to yield linear *γ-amino enone* **3a-Cbz** as a yellow oil (38.8 mg, 76%).

^{1}H NMR (400 MHz, $CDCl_3$): δ 7.61 (1H, d, *J* 7.5, Ar-*H*), 7.49-7.45 (1H, m, Ar-*H*), 7.38-7.35 (6H, m, Ar-*H*), 7.20 (1H, app t, *J* 7.5, Ar-*H*), 6.87-6.78 (2H, m, COC*H*C*H*), 5.16 (3H, br s, N*H* and CO_2CH_2), 4.09 (2H, dd, *J* 5.5, 3.5, $NHCH_2$), 2.45 (3H, s, SCH_3); ^{13}C NMR (101 MHz, $CDCl_3$): δ 192.3, 156.2, 144.9, 141.0, 136.3, 136.1, 131.7, 129.8, 128.6, 128.3, 128.2, 127.9, 126.0, 124.0, 67.1, 42.2, 16.3; IR: ν_{max} (neat)/cm^{-1} 2919, 2777, 1704, 1667, 1619, 1528, 1249; LRMS (ESI$^+$): *m/z* 342 ([M+H]$^+$, 100%), 364 ([M+Na]$^+$, 95%); HRMS (ESI$^+$) found 364.0979 [M+Na]$^+$, $C_{19}H_{19}NO_3SNa^+$ requires 364.0978.

Benzyl {2-[2-(methylthio)benzoyl]allyl}carbamate, 3a'-Cbz

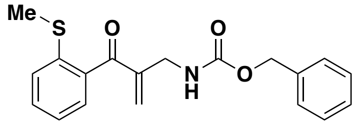

Prepared according to general procedure **F** using 2-(methylthio)benzaldehyde

1a (19 μL, 0.15 mmol, 1.0 equiv.), propargylic amine (benzyl prop-2-yn-1-ylcarbamate **2s**) (42.6 mg, 0.225 mmol, 1.3 equiv.), [Rh(nbd$_2$)]BF$_4$ (2.8 mg, 5 mol%) and dcpm (3.1 mg, 5 mol%) in acetone (88 μL, 1 M). After stirring for 16 hrs at room temperature the reaction gave a crude mixture of regioisomers (4:1, linear: branched). The compounds were separated by flash column chromatography (5-20% EtOAc in petrol) to yield branched *γ-amino enone* **3a'-Cbz** as a cream oil (9.3 mg, 18%).

^{1}H NMR (400 MHz, CDCl$_3$): δ 7.45 (1H, app td, *J* 7.5, 1.5, Ar-*H*), 7.40-7.35 (7H, m, Ar-*H*), 7.22 (1H, app t, *J* 7.5, Ar-*H*), 6.16 (1H, app s, NHCH$_2$CC*H$_a$*H$_b$CO), 5.71 (1H, app s, NHCH$_2$CCH$_a$*H$_b$*CO), 5.37 (1H, t, *J* 5.0, N*H*), 5.15 (2H, s, CO$_2$C*H$_2$*), 4.22 (2H, d, *J* 6.5, NHC*H$_2$*), 2.43 (3H, s, SC*H$_3$*); ^{13}C NMR (101 MHz, CDCl$_3$): δ 156.4, 145.0, 138.2, 137.8, 136.5, 131.0, 129.92, 129.88, 129.1, 128.5, 128.1, 128.0, 127.7, 124.6, 66.8, 41.9, 17.1; IR: ν_{max} (neat)/cm^{-1} 2970, 2931, 1702, 1467, 1378, 1160; LRMS (ESI$^+$): *m/z* 364 ([M+Na]$^+$, 100%); HRMS (ESI$^+$) found 364.09781 [M+Na]$^+$, C$_{19}$H$_{19}$NO$_3$SNa$^+$ requires 364.09779.

(*E*)-4-Methyl-*N*-{4-[2-(methylthio)phenyl]-4-oxobut-2-en-1-yl}benzenesulfonamide, 3a-Ts

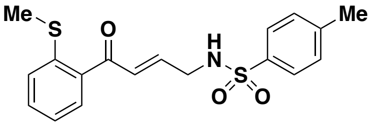

Prepared according to general procedure **F** using 2-(methylthio)benzaldehyde **1a** (19 μL, 0.15 mmol, 1.0 equiv.), propargylic amine (4-methyl-*N*-(prop-2-yn-1-yl)benzenesulfonamide **2t**) (47.1 mg, 0.225 mmol, 1.3 equiv.), [Rh(nbd$_2$)]BF$_4$ (2.8 mg, 5 mol%) and PNP(Cy) (3.2 mg, 5 mol%) in acetone (84 μL, 1 M). After stirring for 10 min at room temperature the reaction gave a crude mixture of regioisomers (4:1, linear: branched). The compounds were separated by flash column chromatography (5-20% EtOAc in petrol) to yield linear *γ-amino enone* **3a-Ts** as a yellow solid (17.4 mg, 32%).

^{1}H NMR (500 MHz, CDCl$_3$): δ 7.79 (2H, d, *J* 8.0, Ar-*H*), 7.56 (1H, d, *J* 7.5, Ar-*H*), 7.48 (1H, app t, *J* 7.5, Ar-*H*), 7.35 (3H, dd, *J* 12.5, 8.0, Ar-*H*), 7.21 (1H, t, *J* 7.5, Ar-*H*), 6.84 (1H, d, *J* 15.5, COC*H*), 6.74-6.69 (1H, m, COCHC*H*), 4.68 (1H, t, *J* 6.0, N*H*), 3.89 (2H, t, *J* 5.0, NHC*H$_2$*), 2.46 (3H, s, SC*H$_3$*), 2.44 (3H, s, Ar-C*H$_3$*); ^{13}C NMR (125 MHz, CDCl$_3$): δ 191.4, 143.9, 142.4, 141.4, 136.8, 135.6, 132.0, 129.91, 129.72, 128.7, 127.1, 125.9, 123.9, 44.3, 21.6, 16.3; IR: ν_{max} (neat)/cm^{-1} 2941, 2831, 1716, 1554, 1433, 1328, 1159; LRMS (ESI$^+$): *m/z* 362 ([M+H]$^+$, 100%), 384 ([M+Na]$^+$, 55%); HRMS (ESI$^+$) found 384.0700 [M+Na]$^+$, C$_{18}$H$_{19}$NO$_3$S$_2$Na$^+$ requires 384.0699; mp: 107-109 °C (DCM/hexane).

4-Methyl-*N*-{2-[2-(methylthio)benzoyl]allyl}benzenesulfonamide, 3a'-Ts

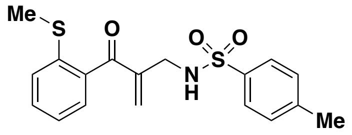

Prepared according to general procedure **F** using 2-(methylthio)benzaldehyde **1a** (19 μL, 0.15 mmol, 1.0 equiv.), propargylic amine (4-methyl-*N*-(prop-2-yn-1-yl)benzenesulfonamide **2t**) (47.1 mg, 0.225 mmol, 1.3 equiv.), [Rh(nbd$_2$)]BF$_4$ (2.8 mg, 5 mol%) and dcpm (3.1 mg, 5 mol%) in acetone (84 μL, 1 M). After stirring for 16 hrs at room temperature the reaction gave a crude mixture of regioisomers (1:1, linear: branched). The compounds were separated by flash column chromatography (5-20% EtOAc in petrol) to yield branched *γ-amino enone* **3a'-Ts** as a cream oil (21.9 mg, 40%).

^{1}H NMR (400 MHz, CDCl$_3$): δ 7.80-7.78 (2H, m, Ar-*H*), 7.44 (1H, ddd, *J* 8.0, 7.0, 1.5, Ar-*H*), 7.37 (1H, d, *J* 7.5, Ar-*H*), 7.33 (2H, dd, *J* 8.5, 0.5, Ar-*H*), 7.19-7.15 (1H, m, Ar-*H*), 7.15-7.11 (1H, m, Ar-*H*), 6.15 (1H, app s, NHCH$_2$CC*H$_a$*H$_b$CO), 5.68 (1H, app s, NHCH$_2$CCH$_a$*H$_b$*CO), 5.18 (1H, t, *J* 6.5, N*H*), 4.00 (2H, d, *J* 6.5, NHC*H$_2$*), 2.46 (3H, s, SC*H$_3$*), 2.43 (3H, s, Ar-C*H$_3$*); ^{13}C NMR (101 MHz, CDCl$_3$): δ 197.6, 143.5, 139.4, 138.5, 137.2, 137.1, 131.21, 131.17, 129.8, 129.1, 127.5, 127.2, 124.4, 44.2, 21.6, 16.9; IR: ν_{max} (neat)/cm^{-1} 2923, 2852, 1652, 1598, 1434, 1329, 1184, 1093; LRMS (ESI$^+$): *m/z* 384 ([M+Na]$^+$, 100%); HRMS (ESI$^+$) found 384.06992 [M+Na]$^+$, $C_{18}H_{19}NO_3S_2Na^+$ requires 384.06989.

***tert*-Butyl (*E*)-{5-[2-(methylthio)phenyl]-5-oxopent-3-en-2-yl}carbamate, 3b**

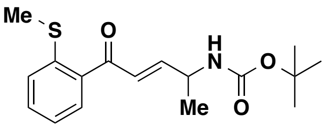

Prepared according to general procedure **F** using 2-(methylthio)benzaldehyde **1a** (19 μL, 0.15 mmol, 1.0 equiv.), propargylic amine (*tert*-butyl but-3-yn-2-ylcarbamate **2b**) (38.0 mg, 0.225 mmol, 1.5 equiv.), [Rh(nbd$_2$)]BF$_4$ (2.8 mg, 5 mol%) and PNP(Cy) (3.2 mg, 5 mol%) in acetone (93 μL, 1 M). After stirring for 1 hr at room temperature the reaction gave a crude mixture of regioisomers (25:1, linear: branched). The compounds were separated by flash column chromatography (5-10% EtOAc in petrol) to yield linear *γ-amino enone* **3b** as a yellow oil (42.8 mg, 89%).

^{1}H NMR (400 MHz, CDCl$_3$): δ 7.64 (1H, dd, *J* 7.5, 1.5, Ar-*H*), 7.46 (1H, app td, *J* 7.5, 1.5, Ar-*H*), 7.36 (1H, d, *J* 7.5, Ar-*H*), 7.20 (1H, app t, *J* 7.5, Ar-*H*), 6.81-6.73 (2H, m, COC*H*C*H*), 4.65 (1H, br s, N*H*), 4.49-4.47 (1H, m, NHC*H*(CH$_3$)), 2.45 (3H, s, SC*H$_3$*),

1.47 (9H, s, $CO_2C(CH_3)_3$), 1.32 (3H, d, *J* 7.0, NHCH(CH_3)); ^{13}C NMR (101 MHz, $CDCl_3$): δ 192.7, 155.0, 149.9, 141.0, 136.3, 131.7, 129.8, 126.6, 126.0, 123.9, 47.5, 28.4, 27.3, 20.4, 16.4; IR: ν_{max} (neat)/cm^{-1} 3343, 2977, 1694, 1619, 1586, 1511, 1434, 1366, 1247, 1209, 1165, 1049, 1009; LRMS (ESI$^+$): *m/z* 322 ([M+H]$^+$, 10%), 344 ([M+Na]$^+$, 100%); HRMS (ESI$^+$) found 344.1292 [M+Na]$^+$, $C_{17}H_{23}NO_3SNa^+$ requires 344.1291.

***tert*-Butyl (*E*)-{4-[2-(methylthio)phenyl]-4-oxo-1-phenylbut-2-en-1-yl}carbamate, 3c**

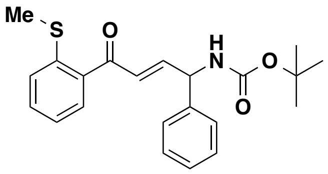

Prepared according to general procedure **F** using 2-(methylthio)benzaldehyde **1a** (0.39 mL, 3.0 mmol, 1.0 equiv.), propargylic amine (*tert*-butyl (1-phenylprop-2-yn-1-yl)carbamate **2d**) (0.90 g, 3.9 mmol, 1.3 equiv.), [Rh(nbd$_2$)]BF_4 (11.2 mg, 1 mol%) and PNP(Cy) (14.2 mg, 1 mol%) in acetone (1.7 mL, 1 M). After stirring for 15 mins at room temperature the reaction gave the linear *γ*-amino enone, as the only regioisomer detectable by ^{1}H NMR. The crude reaction mixture was purified by flash column chromatography (10-40% Et_2O in petrol) to yield *γ-amino enone* **3c** as a yellow solid (1.01 g, 88%).

^{1}H NMR (400 MHz, $CDCl_3$): δ 7.67 (1H, dd, *J* 7.5, 1.5, Ar-*H*), 7.49-7.45 (2H, m, Ar-*H*), 7.41-7.29 (5H, m, Ar-*H*), 7.21 (1H, app td, *J* 7.5, 1.5, Ar-*H*), 7.00 (1H, dd, *J* 15.5, 5.0, COCHC*H*), 6.87 (1H, dd, *J* 15.5, 1.5, COC*H*CH), 5.55 (1H, br s, N*H*), 5.02 (1H, d, *J* 5.0, NHC*H*), 2.46 (3H, s, SCH_3), 1.48 (9H, s, $CO_2C(CH_3)_3$); ^{13}C NMR (101 MHz, $CDCl_3$): δ 192.4, 154.8, 147.4, 141.1, 139.3, 136.2, 131.7, 129.9, 129.0, 128.2, 127.3, 126.1, 124.0, 80.2, 55.9, 30.3, 28.4, 16.4; IR: ν_{max} (neat)/cm^{-1} 3343, 2939, 1692, 1619, 1495, 1434, 1366, 1249, 1167, 1025; LRMS (ESI$^+$): *m/z* 384 ([M+H]$^+$, 100%), 406 ([M+Na]$^+$, 25%); HRMS (ESI$^+$) found 406.1444 [M+Na]$^+$, $C_{22}H_{25}NO_3SNa^+$ requires 406.1447; mp: 144-145 °C (DCM/hexane).

***tert*-Butyl (*E*)-{2,2-dimethyl-6-[2-(methylthio)phenyl]-6-oxohex-4-en-3-yl}carbamate, 3d**

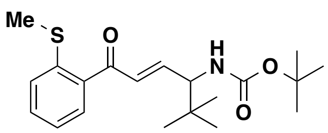

Prepared according to general procedure **F** using 2-(methylthio)benzaldehyde **1a** (19 *μ*L, 0.15 mmol, 1.0 equiv.), propargylic amine (*tert*-butyl (4,4-dimethylpent-1-yn-3-yl)carbamate **2c**) (47.5 mg, 0.225 mmol, 1.5 equiv.), [Rh(nbd$_2$)]BF_4 (2.8 mg, 5 mol%) and PNP(Cy) (3.2 mg, 5 mol%) in acetone (79

μL, 1 M). After stirring for 3 hrs at room temperature the reaction gave the linear γ-amino enone, as the only regioisomer detectable by ^{1}H NMR. The crude reaction mixture was purified by flash column chromatography (25% EtOAc in petrol) to yield *γ-amino enone* **3d** as a yellow solid (49.6 mg, 91%).

^{1}H NMR (400 MHz, $CDCl_3$): δ 7.68 (1H, dd, *J* 7.5, 1.5, Ar-*H*), 7.48 (1H, app td, *J* 7.5, 1.5, Ar-*H*), 7.38 (1H, d, *J* 7.5, Ar-*H*), 7.23 (1H, app td, *J* 7.5, 1.5, Ar-*H*), 6.91 (1H, dd, *J* 15.5, 6.0, COCHC*H*), 6.83 (1H, d, *J* 15.5, COC*H*CH), 4.68 (1H, d, *J* 9.0, N*H*), 4.20 (1H, dd, *J* 9.0, 6.0, NHC*H*), 2.47 (3H, s, SCH_3), 1.48 (9H, s, $CO_2C(CH_3)_3$), 0.99 (9H, s, NHCHC(CH_3)$_3$); ^{13}C NMR (101 MHz, $CDCl_3$): δ 192.3, 155.4, 146.5, 141.1, 131.7, 129.8, 128.6, 126.0, 123.9, 79.7, 60.5, 35.0, 28.4, 26.5, 25.4, 16.4; IR: ν_{max} (neat)/cm^{-1} 2968, 1698, 1618, 1501, 1366, 1247, 1169, 1050, 1011; LRMS (ESI$^+$): *m/z* 364 ([M+H]$^+$, 45%), 386 ([M+Na]$^+$, 100%); HRMS (ESI$^+$) found 386.1755 [M+Na]$^+$, $C_{20}H_{29}NO_3SNa^+$ requires 386.1760; mp: 130-131 °C (EtOAc/petrol).

***tert*-Butyl (*E*)-{4-[2-(methylthio)benzoyl]dec-3-en-2-yl}carbamate, 3e**

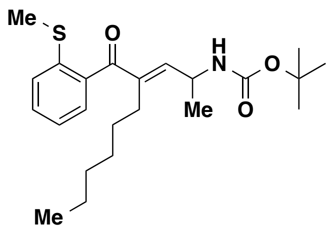

An oven-dried microwave vial was charged with [Rh(nbd)$_2$BF$_4$] (2.8 mg, 5 mol%) and dppm (2.9 mg, 5 mol%). Once under an inert atomosphere, they were dissolved in acetone (1.0 mL). Hydrogen gas was bubbled through the solution at room temperature for 1-2 mins in order to generate the active catalyst species. The hydrogen gas was purged using nitrogen gas, and this was bubbled through the catalyst to dryness. The dry catalyst was dissolved in acetone (74 μL, 1.0 M with respect to aldehyde minus starting materials) and this was transferred to a nitrogen-filled microwave vial containing 2-(methylthio)benzaldehyde **1a** (19 μL, 0.15 mmol, 1.0 equiv.) and propargylic amine (*tert*-butyl dec-3-yn-2-ylcarbamate **2o**) (57.0 mg, 0.225 mmol, 1.5 equiv.). The reaction mixture, once homogenous (on occasion, sonication was required), was then stirred at room temperature. The reaction was monitored by TLC until complete. After 16 hrs, the reaction mixture was filtered through a plug of silica (acetone, 10 mL) and the solvent removed *in vacuo* to obtain the crude product. The crude product was analysed by ^{1}H NMR spectroscopy to measure the ratio of the linear to branched products (8:1, linear: branched). The regioisomers were then separated by flash column chromatography (5% EtOAc in petrol) to afford title linear *γ-amino enone* **3e** as a pale yellow oil (49.3 mg, 81%).

^1H NMR (400 MHz, $CDCl_3$): δ 7.42 (1H, ddd, *J* 8.0, 7.0, 1.5, Ar-*H*), 7.36 (1H, d, *J* 7.0, Ar-*H*), 7.32 (1H, d, *J* 7.0, Ar-*H*), 7.19 (1H, app td, *J* 7.5, 1.5, Ar-*H*), 5.86 (1H, d, *J* 9.0, COCC*H*), 4.61 (1H, br s, N*H*), 4.46 (1H, app br s, NHC*H*), 2.53 (2H, dd, *J* 9.0, 6.0, COCCH_2), 2.44 (3H, s, SCH_3), 1.61-1.48 (2H, m, COCCH_2CH_2), 1.46 (9H, s, $CO_2C(CH_3)_3$), 1.41-1.32 (6H, m, $CH_3(CH_2)_3$), 1.24 (3H, d, *J* 7.0, NHCH(CH_3)), 0.91 (3H, t, *J* 7.0, CH_3(CH_2)$_5$); ^{13}C NMR (101 MHz, $CDCl_3$): δ 198.8, 167.8, 146.7, 141.9, 138.8, 130.4, 129.3, 127.2, 124.5, 100.0, 68.6, 45.1, 31.7, 29.6, 29.2, 28.4, 26.6, 22.6, 21.0, 16.9, 14.1; IR: ν_{max} (neat)/cm^{-1} 2961, 2926, 2855, 1710, 1644, 1492, 1366, 1247, 1166, 1052, 908; LRMS (ESI$^+$): *m/z* 428 ([M+Na]$^+$, 100%); HRMS (ESI$^+$) found 406.24124 [M+H]$^+$, $C_{23}H_{36}O_3NS^+$ requires 406.24104.

tert-Butyl (*E*)-{4-[4-bromo-2-(methylthio)phenyl]-4-oxobut-2-en-1-yl}carbamate, 3f

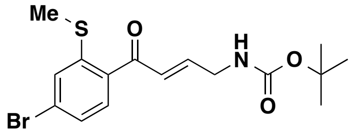

Prepared according to general procedure **F** using 4-bromo-2-(methylthio)benzaldehyde **1e**[3] (34.6 mg, 0.15 mmol, 1.0 equiv.), *N*-Boc-propargylamine **2a** (35.7 mg, 0.225 mmol, 1.5 equiv.), [Rh(nbd$_2$)]BF_4 (2.8 mg, 5 mol%) and PNP(Cy) (3.2 mg, 5 mol%) in acetone (79 μL, 1 M). After stirring for 1 hr at room temperature the reaction gave a crude mixture of regioisomers (18:1, linear: branched). The compounds were separated by flash column chromatography (5% EtOAc in petrol) to yield linear *γ-amino enone* **3f** as a yellow solid (46.2 mg, 80%).

^1H NMR (400 MHz, $CDCl_3$): δ 7.52 (1H, d, *J* 8.0, Ar-*H*), 7.44 (1H, d, *J* 1.5, Ar-*H*), 7.33 (1H, dd, *J* 8.0, 1.5, Ar-*H*), 6.85 (1H, dt, *J* 15.5, 4.0, COCHC*H*), 6.76 (1H, d, *J* 15.5, COC*H*CH), 4.84 (1H, br s, N*H*), 4.02 (2H, app br s, NHCH_2), 2.46 (3H, s, SCH_3), 1.48 (9H, s, $CO_2C(CH_3)_3$); ^{13}C NMR (101 MHz, $CDCl_3$): δ 191.3, 155.6, 146.0, 143.8, 134.4, 131.1, 128.2, 127.3, 126.9, 80.0, 41.8, 28.4, 27.4, 16.3; IR: ν_{max} (neat)/cm^{-1} 2832, 1707, 1573, 1539, 1367, 1281, 1166; LRMS (ESI$^+$): *m/z* 408 ([^{79}Br] [M+Na]$^+$, 100%), 410 ([^{81}Br] [M+Na]$^+$, 95%); HRMS (ESI$^+$) found 408.0235 [[^{79}Br] M+Na]$^+$, 410.0218 [[^{81}Br] M+Na]$^+$, $C_{16}H_{20}O_3N^{79}BrSNa^+$ requires 408.0239, $C_{16}H_{20}O_3N^{81}BrSNa^+$ requires 410.0219; mp: 90-92 °C (DCM/hexane).

tert-Butyl (_E_)-[6-(methylthio)-4-oxohex-2-en-1-yl]carbamate, 3g

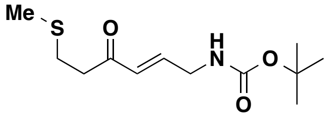

Prepared according to general procedure **F** using 3-(methylthio)propionaldehyde **1b** (15 μL, 0.15 mmol, 1.0 equiv.), *N*-Boc-propargylamine **2a** (35.7 mg, 0.225 mmol, 1.5 equiv.), [Rh(nbd$_2$)]BF_4 (2.8 mg, 5 mol%) and PNP(Cy) (3.2 mg, 5 mol%) in acetone (99 μL, 1 M). After stirring for 3 hrs at room temperature the reaction gave a crude mixture of regioisomers (25:1, linear: branched). The compounds were separated by flash column chromatography (5% EtOAc in petrol) to yield linear *γ-amino enone* **3g** as a yellow oil (24.8 mg, 64%).

^{1}H NMR (400 MHz, $CDCl_3$): δ 6.81 (1H, dt, *J* 16.0, 5.5, COCHC*H*), 6.22 (1H, d, *J* 16.0, COC*H*CH), 4.84 (1H, br s, N*H*), 3.96 (2H, app br s, NHC*H*$_2$), 2.90-2.76 (4H, m, SC*H*$_2$C*H*$_2$), 2.13 (3H, s, SC*H*$_3$), 1.46 (9H, s, $CO_2C(CH_3)_3$); ^{13}C NMR (101 MHz, $CDCl_3$): δ 198.1, 155.6, 143.4, 129.2, 80.1, 41.5, 40.1, 28.4, 28.2, 15.9; IR: ν_{max} (neat)/cm^{-1} 3352, 2978, 1693, 1634, 1513, 1392, 1366, 1248, 1164; LRMS (ESI$^+$): *m/z* 282 ([M+Na]$^+$, 100%); HRMS (ESI$^+$) found 282.1132 [M+Na]$^+$, $C_{12}H_{21}NO_3SNa^+$ requires 282.1134.

tert-Butyl (_E_)-{4-[2-(methylthio)cyclohex-1-en-1-yl]-4-oxobut-2-en-1-yl}carbamate, 3h

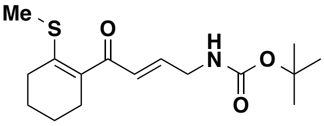

Prepared according to general procedure **F** using 2-(methylthio)cyclohex-1-ene-1-carbaldehyde **2d**[2] (23.4 mg, 0.15 mmol, 1.0 equiv.), *N*-Boc-propargylamine **2a** (35.7 mg, 0.225 mmol, 1.5 equiv.), [Rh(nbd$_2$)]BF_4 (2.8 mg, 5 mol%) and PNP(Cy) (3.2 mg, 5 mol%) in acetone (91 μL, 1 M). After stirring for 15 mins at 0 ºC the reaction gave a crude mixture of regioisomers (12:1, linear: branched). The compounds were separated by flash column chromatography (5% EtOAc in petrol) to yield linear *γ-amino enone* **3h** as a pale yellow solid (33.2 mg, 71%).

^{1}H NMR (400 MHz, $CDCl_3$): δ 6.80 (1H, dt, *J* 15.5, 5.0, COCHC*H*), 6.54 (1H, d, *J* 15.5, COC*H*CH), 4.72 (1H, br s, N*H*), 3.97 (2H, app s, NHC*H*$_2$), 2.48 (2H, t, *J* 6.0, SCC*H*$_2$), 2.41 (2H, t, *J* 6.0, COCC*H*$_2$), 2.26 (3H, s, SC*H*$_3$), 1.76-1.69 (4H, m, $SCCH_2(CH_2)_2$), 1.48 (9H, s, $CO_2C(CH_3)_3$); ^{13}C NMR (101 MHz, $CDCl_3$): δ 192.8, 155.6, 145.1, 142.8, 132.4, 127.8, 79.8, 41.8, 29.9, 28.4, 28.3, 23.0, 21.9, 15.0; IR: ν_{max} (neat)/cm^{-1} 2930, 1703, 1609, 1510, 1366, 1250, 1169; LRMS (ESI$^+$): *m/z* 312 ([M+H]$^+$,

100%), 334 ([M+Na]$^+$, 90%); HRMS (ESI$^+$) found 334.1456 [M+Na]$^+$, $C_{16}H_{25}NO_3SNa^+$ requires 334.1447; mp: 99-102 °C (DCM/hexane).

***tert*-Butyl (*E*)-{4-[3-(methylthio)thiophen-2-yl]-4-oxobut-2-en-1-yl}carbamate, 3i**

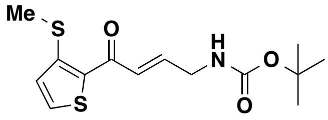

Prepared according to general procedure **F** using 3-(methylthio)thiophene-2-carbaldehyde **1m** (23.7 mg, 0.15 mmol, 1.0 equiv.), *N*-Boc-propargylamine **2a** (35.7 mg, 0.225 mmol, 1.5 equiv.), [Rh(nbd$_2$)]BF_4 (2.8 mg, 5 mol%) and PNP(Cy) (3.2 mg, 5 mol%) in acetone (90 μL, 1 M). After stirring for 16 hrs at room temperature the reaction gave a crude mixture of regioisomers (6:1, linear: branched). The compounds were separated by flash column chromatography (5% EtOAc in petrol) to yield linear *γ-amino enone* **3i** as a yellow oil (36.2 mg, 77%).

^{1}H NMR (400 MHz, $CDCl_3$): δ 7.60 (1H, d, *J* 5.0, Ar-*H*), 7.08 (1H, d, *J* 5.0, Ar-*H*), 7.03 (1H, dt, *J* 15.0, 5.0, COCHC*H*), 6.79 (1H, dt, *J* 15.0, 2.0, COC*H*CH), 4.84 (1H, br s, N*H*), 4.02 (2H, app br s, NHC*H$_2$*), 2.56 (3H, s, SC*H$_3$*), 1.49 (9H, s, $CO_2C(CH_3)_3$); ^{13}C NMR (101 MHz, $CDCl_3$): δ 180.9, 155.6, 143.7, 131.3, 128.6, 126.4, 125.0, 110.5, 83.5, 28.4, 23.9, 16.7; IR: ν_{max} (neat)/cm^{-1} 2978, 2928, 1687, 1655, 1585, 1495, 1402, 1242, 1163, 908; LRMS (ESI$^+$): *m/z* 314 ([M+H]$^+$, 50%), 336 ([M+Na]$^+$, 100%); HRMS (ESI$^+$) found 314.08762 [M+H]$^+$, $C_{14}H_{20}O_3NS_2^+$ requires 314.08791.

***tert*-Butyl (*E*)-{4-[2-(dimethylamino)phenyl]-4-oxobut-2-en-1-yl}carbamate, 3j**

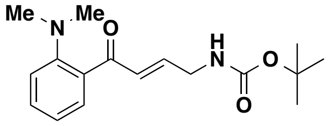

Prepared according to general procedure **F** using 2-(dimethylamino)benzaldehyde **1g**[5] (22.4 mg, 0.15 mmol, 1.0 equiv.), *N*-Boc-propargylamine **2a** (35.7 mg, 0.225 mmol, 1.5 equiv.), [Rh(nbd$_2$)]BF_4 (2.8 mg, 5 mol%) and PNP(Cy) (3.2 mg, 5 mol%) in acetone (92 μL, 1 M). After stirring for 6 hrs at room temperature the reaction gave the linear γ-amino enone, as the only regioisomer detectable by ^{1}H NMR. The crude reaction mixture was purified by flash column chromatography (25% EtOAc in petrol) to yield *γ-amino enone* **3j** as a yellow solid (45.3 mg, 99%).

^{1}H NMR (400 MHz, $CDCl_3$): δ 7.46 (1H, dd, *J* 7.5, 2.0, Ar-*H*), 7.38 (1H, ddd, *J* 8.0, 7.5, 2.0, Ar-*H*), 6.99 (1H, dd, *J* 8.0, 1.0, Ar-*H*), 6.94 (1H, dd, *J* 7.5, 1.0, Ar-*H*), 6.88 (1H, d, *J* 12.0, COC*H*CH), 6.82 (1H, dt, *J* 12.0, 2.0, COCHC*H*), 4.81 (1H, br s, N*H*), 3.98 (2H, app br s, NHCH_2), 2.80 (6H, s, N(CH_3)$_2$), 1.47 (9H, s, $CO_2C(CH_3)_3$); ^{13}C NMR (101 MHz, $CDCl_3$): δ 194.7, 152.1, 142.7, 132.0, 130.5, 129.0, 119.8, 116.7, 79.7, 44.2, 41.6, 28.4, 21.1, 14.2; IR: ν_{max} (neat)/cm^{-1} 3352, 2978, 1698, 1668, 1619, 1595, 1497, 1433, 1366, 1280, 1250, 1165; LRMS (ESI$^+$): *m/z* 305 ([M+H]$^+$, 100%); HRMS (ESI$^+$) found 327.1681 [M+Na]$^+$, $C_{17}H_{24}N_2O_3Na^+$ requires 327.1679; mp: 89-91 °C (DCM/hexane).

7. One-pot formation of functionalised pyrrole rings

General procedure G for the synthesis of *N*-Boc pyrroles as exemplified by the synthesis of *tert*-butyl 2-[2-(methylthio)phenyl]-1*H*-pyrrole-1-carboxylate, 4a

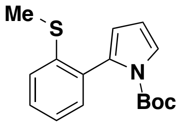

An oven-dried microwave vial was charged with [Rh(nbd)$_2$BF$_4$] (2.8 mg, 5 mol%) and PNP(Cy) (3.2 mg, 5 mol%) (for use with terminal alkynes) or dppm (for use with internal alkynes). Once under an inert atmosphere, they were dissolved in acetone (1 mL). Hydrogen gas was bubbled through the solution at room temperature for 1-2 mins in order to generate the active catalyst species. The hydrogen gas was purged using nitrogen gas, and this was bubbled through the catalyst to dryness. The dry catalyst was dissolved in acetone (75 μL, 1.0 M with respect to aldehyde minus starting materials) and this was transferred to a nitrogen-filled microwave vial containing 2-(methylthio)benzaldehyde **1a** (19 μL, 0.15 mmol, 1.0 equiv.) and *N*-Boc-propargylamine **2a** (30.2 mg, 0.195 mmol, 1.3 equiv.). The reaction mixture, once homogenous (on occasion, sonication was required), was then stirred at room temperature and monitored by TLC until complete. After 20 mins, the reaction vessel was opened to air followed by the addition of acetonitrile (1.5 mL) and *p*-TSA (42.8 mg, 0.230 mmol, 1.5 equiv.). The reaction mixture was further stirred until complete, after 3 hrs, the solution was diluted with acetonitrile (5 mL) and neutralised by the addition of sat. $NaHCO_3$ $_{(aq)}$ (10 mL) in a separatory funnel. The aqueous mixture was extracted with EtOAc (3 × 5 mL) and the combined organic extracts were washed with brine (10 mL) and dried over $MgSO_4$. The solvent was removed *in vacuo* to obtain the crude product and purified by flash column chromatography (5-10% Et_2O in petrol) to afford title *pyrrole* **4a** as a colourless oil (34.8 mg, 81%).

^1H NMR (400 MHz, $CDCl_3$): δ 7.44 (1H, dd, *J* 3.5, 2.0, NC*H*), 7.35 (1H, ddd, *J* 8.0, 7.5, 1.5, Ar-*H*), 7.25 (2H, app qd, *J* 3.5, 1.5, Ar-*H*), 7.18 (1H, app td, *J* 7.5, 1.5, Ar-*H*), 6.31 (1H, app t, *J* 3.5, NCHC*H*), 6.18 (1H, dd, *J* 3.5, 2.0, NCC*H*), 2.38 (3H, s, SC*H₃*), 1.28 (9H, s, $CO_2C(CH_3)_3$); ^{13}C NMR (101 MHz, $CDCl_3$): δ 149.1, 139.9, 134.2, 132.0, 130.3, 128.3, 124.8, 124.3, 121.7, 114.4, 110.5, 83.1, 27.4, 15.5; IR: ν_{max} (neat)/cm^{-1} 2981, 1738, 1438, 1396, 1370, 1340, 1313, 1152, 1085; LRMS (ESI^+): *m/z* 290 ($[M+H]^+$, 100%), 312 ($[M+Na]^+$, 50%); HRMS (ESI^+) found 312.1033 $[M+Na]^+$, $C_{16}H_{19}NO_2SNa^+$ requires 312.1029.

tert-Butyl 2-methyl-5-[2-(methylthio)phenyl]-1*H*-pyrrole-1-carboxylate, 4b

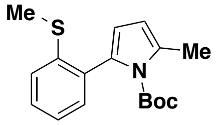

Prepared according to general procedure **G** using 2-(methylthio)benzaldehyde **1a** (19 *μ*L, 0.15 mmol, 1.0 equiv), propargylic amine (*tert*-butyl but-3-yn-2-ylcarbamate **2b**) (33.0 mg, 0.195 mmol, 1.3 equiv.), $[Rh(nbd_2)]BF_4$ (2.8 mg, 5 mol%) and PNP(Cy) (3.2 mg, 5 mol%) in acetone (98 *μ*L, 1 M). After stirring for 4 hrs at room temperature, acetonitrile (1.5 mL) and *p*-TSA (42.8 mg, 0.230 mmol, 1.5 equiv.) were added and stirred at room temperature for a further 3 hrs. The crude product was purified by flash column chromatography (5-10% Et_2O in petrol) to afford *pyrrole* **4b** as a colourless oil (37.2 mg, 82%).

^1H NMR (400 MHz, $CDCl_3$): δ 7.33 (1H, ddd, *J* 8.0, 7.5, 2.0, Ar-*H*), 7.24-7.19 (2H, m, Ar-*H*), 7.16 (1H, app td, *J* 7.5, 1.5, Ar-*H*), 6.06 (1H, d, *J* 3.0, NC(Ar)C*H*), 6.03 (1H, dq, *J* 3.0, 1.0, $NC(CH_3)CH$), 2.52 (3H, d, *J* 1.0, $NC(CH_3)$), 2.37 (3H, s, SCH_3), 1.21 (9H, s, $CO_2C(CH_3)_3$); ^{13}C NMR (101 MHz, $CDCl_3$): δ 149.9, 139.4, 135.0, 132.9, 131.8, 130.2, 128.0, 124.3, 124.2, 112.7, 110.5, 82.8, 27.4, 15.8, 15.3; IR: ν_{max} (neat)/cm^{-1} 2942, 2832, 1742, 1367, 1313, 1149, 1024; LRMS (ESI^+): *m/z* 304 ($[M+H]^+$, 100%), 326 ($[M+Na]^+$, 95%); HRMS (ESI^+) found 326.1184 $[M+Na]^+$, $C_{17}H_{21}NO_2SNa^+$ requires 326.1185.

tert-Butyl 2-(_tert_-butyl)-5-[2-(methylthio)phenyl]-1*H*-pyrrole-1-carboxylate, 4c

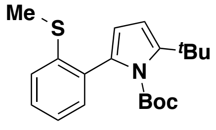

Prepared according to general procedure **G** using 2-(methylthio)benzaldehyde **1a** (19 *μ*L, 0.15 mmol, 1.0 equiv.), propargylic amine (*tert*-butyl (4,4-dimethylpent-1-yn-3-yl)carbamate **2c**) (41.2 mg, 0.195 mmol, 1.3 equiv.),

[Rh(nbd$_2$)]BF$_4$ (2.8 mg, 5 mol%) and PNP(Cy) (3.2 mg, 5 mol%) in acetone (90 μL, 1 M). After stirring for 30 mins at room temperature, acetonitrile (1.5 mL) and *p*-TSA (72.5 mg, 0.600 mmol, 4.0 equiv.) were added and stirred at room temperature for a further 3.5 hrs. The crude product was purified by flash column chromatography (5-10% Et$_2$O in petrol) to afford *pyrrole* **4c** as colourless oil (42.6 mg, 82%).

^{1}H NMR (400 MHz, CDCl$_3$): δ 7.33 (1H, ddd, *J* 8.0, 7.5, 1.5, Ar-*H*), 7.24 (1H, dd, *J* 7.5, 1.0, Ar-*H*), 7.20 (1H, dd, *J* 8.0, 1.0, Ar-*H*), 7.15 (1H, app td, *J* 7.5, 1.0, Ar-*H*), 6.11 (1H, d, *J* 3.5, NCC*H*), 6.09 (1H, d, *J* 3.5, NCC*H*), 2.36 (3H, s, SC*H$_3$*), 1.48 (9H, s, CO$_2$C(C*H$_3$*)$_3$), 1.15 (9H, s, NCC(C*H$_3$*)$_3$); ^{13}C NMR (101 MHz, CDCl$_3$): δ 151.3, 147.0, 144.9, 139.6, 134.2, 130.6, 128.0, 124.2, 124.1, 111.0, 107.4, 83.1, 33.1, 30.4, 27.0, 15.2; IR: ν_{max} (neat)/cm^{-1} 2966, 1748, 1457, 1393, 1368, 1326, 1299, 1257, 1159, 1144, 1084, 1003; LRMS (ESI$^+$): *m/z* 346 ([M+H]$^+$, 100%), 368 ([M+Na]$^+$, 20%); HRMS (ESI$^+$) found 368.1650 [M+Na]$^+$, $C_{20}H_{27}NO_2SNa^+$ requires 368.1655.

***tert*-Butyl 2-[2-(methylthio)phenyl]-5-phenyl-1*H*-pyrrole-1-carboxylate, 4d**

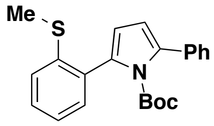

Prepared according to general procedure **G** using 2-(methylthio)benzaldehyde **1a** (0.41 mL, 3.20 mmol, 1.0 equiv.), propargylic amine (*tert*-butyl (1-phenylprop-2-yn-1-yl)carbamate **2d**) (0.96 g, 4.16 mmol, 1.3 equiv.), [Rh(nbd$_2$)]BF$_4$ (23.9 mg, 2 mol%) and PNP(Cy) (30.3 mg, 2 mol%) in acetone (1.8 mL, 1 M). After stirring for 30 mins at room temperature, acetonitrile (10 mL) and *p*-TSA (0.61 g, 3.20 mmol, 1.0 equiv.) were added and stirred at room temperature for a further 4 hrs. The crude product was purified by flash column chromatography (5-10% Et$_2$O in petrol) to afford *pyrrole* **4d** as a white solid (1.04 g, 88%).

^{1}H NMR (400 MHz, CDCl$_3$): δ 7.48-7.45 (2H, m, Ar-*H*), 7.43-7.39 (3H, m, Ar-*H*), 7.38-7.34 (2H, m, Ar-*H*), 7.28 (1H, dd, *J* 8.0, 1.0, Ar-*H*), 7.23 (1H, td, *J* 7.5, 1.0, Ar-*H*), 6.33 (1H, d, *J* 3.5, NCC*H*), 6.25 (1H, d, *J* 3.5, NCC*H*), 2.42 (3H, s, SC*H$_3$*), 1.16 (9H, s, CO$_2$C(C*H$_3$*)$_3$); ^{13}C NMR (101 MHz, CDCl$_3$): δ 149.2, 139.8, 136.0, 134.5, 133.9, 133.6, 130.6, 128.8, 128.5, 127.7, 127.0, 124.6, 124.3, 112.9, 112.6, 83.2, 27.1, 15.4; IR: ν_{max} (neat)/cm^{-1} 2980, 1748, 1748, 1482, 1368, 1304, 1147; LRMS (ESI$^+$): *m/z* 366 ([M+H]$^+$, 100%), 388 ([M+Na]$^+$, 25%); HRMS (ESI$^+$) found 388.1338 [M+Na]$^+$, $C_{22}H_{23}NO_2SNa^+$ requires 388.1342; mp: 94-95 °C (DCM/hexane).

tert-Butyl 2-(4-methoxyphenyl)-5-[2-(methylthio)phenyl]-1H-pyrrole-1-carboxylate, 4e

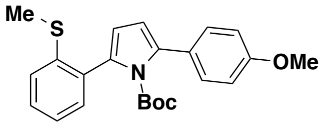

Prepared according to general procedure **G** using 2-(methylthio)benzaldehyde **1a** (19 μL, 0.15 mmol, 1.0 equiv.), propargylic amine (*tert*-butyl [1-(4-methoxyphenyl)prop-2-yn-1-yl]carbamate **2e**) (51.0 mg, 0.195 mmol, 1.3 equiv.), $[Rh(nbd_2)]BF_4$ (2.8 mg, 5 mol%) and PNP(Cy) (3.2 mg, 5 mol%) in acetone (70 μL, 1 M). After stirring for 2 hrs at room temperature, acetonitrile (1.5 mL) and *p*-TSA (42.8 mg, 0.230 mmol, 1.5 equiv.) were added and stirred at room temperature for a further 3 hrs. The crude product was purified by flash column chromatography (5-10% Et_2O in petrol) to afford *pyrrole* **4e** as colourless oil (46.8 mg, 79%).

^{1}H NMR (400 MHz, $CDCl_3$): δ 7.39-7.35 (3H, m, Ar-*H*), 7.32 (1H, dd, *J* 7.5, 1.5, Ar-*H*), 7.25 (1H, d, *J* 7.5, Ar-*H*), 7.21 (1H, app t, *J* 7.5, Ar-*H*), 6.94 (2H, d, *J* 8.5, Ar-*H*), 6.25 (1H, d, *J* 3.5, NCC*H*), 6.22 (1H, d, *J* 3.5, NCC*H*), 3.87 (3H, s, OC*H*$_3$), 2.40 (3H, s, SC*H*$_3$), 1.15 (9H, s $CO_2C(CH_3)_3$); ^{13}C NMR (101 MHz, $CDCl_3$): δ 158.8, 149.3, 139.8, 135.9, 134.1, 133.2, 130.6, 130.0, 128.3, 127.0, 124.5, 124.3, 113.2, 112.9, 112.1, 83.1, 55.3, 27.1, 15.4; IR: ν_{max} (neat)/cm^{-1} 2979, 1744, 1613, 1493, 1367, 1302, 1246, 1176, 730; LRMS (ESI$^+$): *m/z* 396 ([M+H]$^+$, 90%), 418 ([M+Na]$^+$, 100%); HRMS (ESI$^+$) found 396.16249 [M+H]$^+$, $C_{23}H_{26}O_3NS^+$ requires 396.16279.

tert-Butyl 2-(4-cyanophenyl)-5-[2-(methylthio)phenyl]-1H-pyrrole-1-carboxylate, 4f

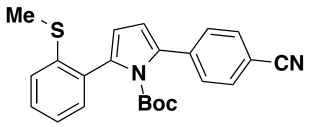

Prepared according to general procedure **G** using 2-(methylthio)benzaldehyde **1a** (19 μL, 0.15 mmol, 1.0 equiv.), propargylic amine (*tert*-butyl [1-(4-cyanophenyl)prop-2-yn-1-yl]carbamate **2g**) (50.0 mg, 0.195 mmol, 1.3 equiv.), $[Rh(nbd_2)]BF_4$ (2.8 mg, 5 mol%) and PNP(Cy) (3.2 mg, 5 mol%) in acetone (81 μL, 1 M). After stirring for 2 hrs at room temperature, acetonitrile (1.5 mL) and *p*-TSA (29.0 mg, 0.150 mmol, 1.0 equiv.) were added and stirred at room temperature for a further 16 hrs. The crude product was purified by flash column chromatography (5-10% Et_2O in petrol) to afford *pyrrole* **4f** as colourless oil (44.6 mg, 76%).

^{1}H NMR (400 MHz, $CDCl_3$): δ 7.69-7.66 (2H, m, Ar-*H*), 7.54-7.51 (2H, m, Ar-*H*),

7.40 (1H, ddd, *J* 8.0, 7.5, 2.0, Ar-*H*), 7.31-7.29 (1H, m, Ar-*H*), 7.26 (1H, d, *J* 7.0, Ar-*H*), 7.22 (1H, app td, *J* 7.5, 2.0, Ar-*H*), 6.40 (1H, d, *J* 3.5, NCC*H*), 6.25 (1H, d, *J* 3.5, NCC*H*), 2.41 (3H, s, SCH_3), 1.15 (9H, s, $CO_2C(CH_3)_3$); ^{13}C NMR (101 MHz, $CDCl_3$): δ 148.9, 139.8, 138.8, 135.2, 134.1, 133.4, 131.6, 130.5, 129.0, 128.8, 124.5, 124.4, 119.1, 114.5, 113.4, 110.3, 84.0, 27.1, 15.3; IR: ν_{max} (neat)/cm^{-1} 2980, 2226, 1745, 1607, 1454, 1301, 1255, 1146, 909; LRMS (ESI^+): *m/z* 391 ($[M+H]^+$, 20%), 413 ($[M+Na]^+$, 100%); HRMS (ESI^+) found 413.12903 $[M+Na]^+$, $C_{23}H_{22}O_2N_2SNa^+$ requires 413.12942.

tert-Butyl 3-hexyl-2-[2-(methylthio)phenyl]-5-phenyl-1*H*-pyrrole-1-carboxylate, 4g

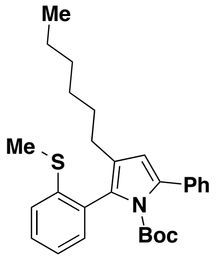

Prepared according to general procedure **G** using 2-(methylthio)benzaldehyde **1a** (19 *µ*L, 0.15 mmol, 1.0 equiv.), propargylic amine (*tert*-butyl (1-phenylnon-2-yn-1-yl)carbamate **2p**) (61.5 mg, 0.195 mmol, 1.3 equiv.), $[Rh(nbd_2)]BF_4$ (2.8 mg, 5 mol%) and dppm (2.9 mg, 5 mol%) in acetone (70 *µ*L, 1 M). After stirring for 4 hrs at room temperature, acetonitrile (1.5 mL) and *p*-TSA (28.5 mg, 0.150 mmol, 1.0 equiv.) were added and stirred at room temperature for a further 4 hrs. The crude product was purified by flash column chromatography (5-10% Et_2O in petrol) to afford *pyrrole* **4g** as colourless oil (57 mg, 85%).

^1H NMR (400 MHz, $CDCl_3$): δ 7.44 (2H, dd, *J* 8.5, 1.5, Ar-*H*), 7.41-7.37 (3H, m, Ar-*H*), 7.34-7.31 (1H, m, Ar-*H*), 7.29-7.20 (3H, m, Ar-*H*), 6.26 (1H, s, NCC*H*), 2.31-2.16 (2H, m, $CH_3(CH_2)_4CH_2$), 1.55 (2H, dd, *J* 7.0, 3.5, $CH_3(CH_2)_3CH_2$), 1.31-1.24 (6H, m, $CH_3(CH_2)_3$), 1.13 (9H, s, $CO_2C(CH_3)_3$), 0.88 (3H, t, *J* 7.0, $CH_3(CH_2)_5$); ^{13}C NMR (101 MHz, $CDCl_3$): δ 149.4, 140.8, 135.3, 134.6, 132.9, 131.3, 129.5, 128.5, 128.4, 127.7, 126.8, 126.2, 124.12, 124.08, 113.9, 82.7, 31.7, 30.2, 29.1, 27.1, 25.7, 22.6, 15.1, 14.1; IR: ν_{max} (neat)/cm^{-1} 3061, 2968, 2855, 1744, 1606, 1484, 1361, 1254, 1074, 991; LRMS (ESI^+): *m/z* 450 ($[M+H]^+$, 100%), 472 ($[M+Na]^+$, 30%); HRMS (ESI^+) found 472.22689 $[M+Na]^+$, $C_{28}H_{35}O_2NSNa^+$ requires 472.22807.

tert-Butyl 5-cyclopropyl-3-hexyl-2-[2-(methylthio)phenyl]-1_H_-pyrrole-1-carboxylate, 4h

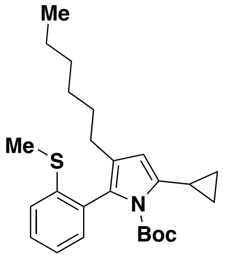

Prepared according to general procedure **G** using 2-(methylthio)benzaldehyde **1a** (63 μL, 0.49 mmol, 1.0 equiv.), propargylic amine (*tert*-butyl (1-cyclopropyldec-2-yn-1-yl)carbamate **2q**) (178 mg, 0.640 mmol, 1.3 equiv.), [Rh(nbd$_2$)]BF_4 (9.35 mg, 5 mol%) and dppm (9.61 mg, 5 mol%) in acetone (0.3 mL, 1 M). After stirring for 16 hrs at room temperature, acetonitrile (1.5 mL) and *p*-TSA (93.0 mg, 0.49 mmol, 1.0 equiv.) were added and stirred at room temperature for a further 2 hrs. The crude product was purified by flash column chromatography (10% Et_2O in petrol) to afford *pyrrole* **4h** as colourless oil (178.3 mg, 88%).

^{1}H NMR (400 MHz, $CDCl_3$): δ 7.32 (1H, ddd, *J* 8.0, 5.0, 3.5, Ar-*H*), 7.19 (1H, d, *J* 8.0, Ar-*H*), 7.17-7.15 (2H, m, Ar-*H*), 5.85 (1H, s, NC(CH(CH_2)$_2$)C*H*), 2.35 (3H, s, SCH_3), 2.33-2.29 (1H, m, NC(C*H*(CH_2)$_2$)CH), 2.18-2.07 (2H, m, CH_3(CH_2)$_4$CH_2), 1.46-1.25 (6H, m, CH_3CH_2(CH_2)$_3$$CH_2$), 1.20 (9H, s, CO_2C(CH_3)$_3$), 0.94-0.83 (6H, m, CH_3CH_2(CH_2)$_4$ and NCCH(C$H_a$$H_bCH_a$$H_b$), 0.75-0.72 (1H, m, NCCH(C$H_a$$H_bCH_a$$H_b$), 0.64-0.58 (1H, m, NCCH(C$H_a$$H_bCH_a$$H_b$), 0.49-0.44 (1H, m, NCCH(C$H_a$$H_bCH_a$$H_b$); ^{13}C NMR (101 MHz, $CDCl_3$): δ 149.8, 145.1, 140.4, 138.4, 133.9, 131.0, 127.8, 127.6, 125.5, 124.0, 109.0, 82.2, 31.6, 30.2, 29.0, 27.4, 25.7, 22.6, 15.0, 14.1, 9.8, 7.2, 6.9; IR: ν_{max} (neat)/cm^{-1} 3085, 2926, 1736, 1590, 1477, 1335, 1158, 1047, 830; LRMS (ESI$^+$): *m/z* 414 ([M+H]$^+$, 100%), 436 ([M+Na]$^+$, 75%); HRMS (ESI$^+$) found 436.22792 [M+H]$^+$, $C_{25}H_{35}O_2NSNa^+$ requires 436.22830.

***tert*-Butyl 2-[4,5-dimethoxy-2-(methylthio)phenyl]-3-hexyl-5-phenyl-1*H*-pyrrole-1-carboxylate, 4i**

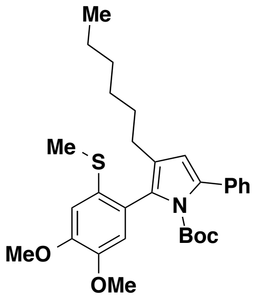

Prepared according to general procedure **G** using 4,5-dimethoxy-2-(methylthio)benzaldehyde[4] **1f** (31.9 mg, 0.15 mmol, 1.0 equiv.), propargylic amine (*tert*-butyl (1-phenylnon-2-yn-1-yl)carbamate **2p**) (61.5 mg, 0.195 mmol, 1.3 equiv.), [Rh(nbd$_2$)]BF$_4$ (2.8 mg, 5 mol%) and dppm (2.9 mg, 5 mol%) in acetone (80 μL, 0.9 M). After stirring for 16 hrs at room temperature, acetonitrile (1.5 mL) and *p*-TSA (29.0 mg, 0.15 mmol, 1.0 equiv.) were added and stirred at room temperature for a further 2 hrs. The crude product was purified by flash column chromatography (5-10% EtOAc in petrol) to afford *pyrrole* **4i** as colourless oil (65.8 mg, 86%).

^{1}H NMR (400 MHz, CDCl$_3$): δ 7.43-7.36 (4H, m, Ar-*H*), 7.32-7.28 (1H, m, Ar-*H*), 6.92 (1H, s, Ar-*H*), 6.84 (1H, s, Ar-*H*), 6.23 (1H, s, NCC*H*), 3.97 (3H, s, OC*H$_3$*), 3.89 (3H, s, OC*H$_3$*), 2.35 (3H, s, SC*H$_3$*), 2.31-2.14 (2H, m, CH$_3$(CH$_2$)$_4$C*H$_2$*), 1.56-1.48 (2H, m, CH$_3$(CH$_2$)$_3$C*H$_2$*), 1.31-1.24 (6H, m, CH$_3$(C*H$_2$*)$_3$), 1.15 (9H, s, CO$_2$C(C*H$_3$*)$_3$), 0.87 (3H, t, *J* 7.0, C*H$_3$*(CH$_2$)$_5$); ^{13}C NMR (101 MHz, CDCl$_3$): δ 149.5, 148.9, 146.8, 135.0, 134.7, 131.0, 129.8, 128.4, 127.7, 126.8, 126.7, 126.1, 114.9, 113.6, 111.2, 82.8, 56.1, 31.7, 30.3, 29.7, 29.2, 27.2, 25.9, 22.6, 17.1, 14.1; IR: ν_{max} (neat)/cm^{-1} 2928, 1742, 1497, 1369, 1247, 1207, 1151, 1070, 908; LRMS (ESI$^+$): *m/z* 510 ([M+H]$^+$, 100%), 532 ([M+Na]$^+$, 45%); HRMS (ESI$^+$) found 510.26648 [M+H]$^+$, $C_{30}H_{40}O_4NS^+$ requires 510.26726.

***tert*-Butyl 2-[3-methoxy-2-(methylthio)phenyl]-5-[4-(methoxycarbonyl)phenyl]-1*H*-pyrrole-1-carboxylate, 4j**

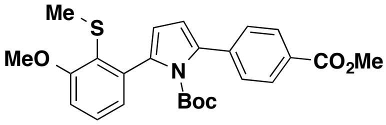

Prepared according to general procedure **G** using 3-methoxy-2-(methylthio)benzaldehyde **1i** (24.3 mg, 0.15 mmol, 1.0 equiv.), propargylic amine (methyl 4-{1-[(*tert*-butoxycarbonyl)amino]prop-2-yn-1-yl}benzoate **2l**) (56.4 mg, 0.195 mmol, 1.3 equiv.), [Rh(nbd$_2$)]BF$_4$ (2.8 mg, 5 mol%) and PNP(Cy) (3.2 mg, 5 mol%) in acetone (66 μL, 1 M). After stirring for 2 hrs at room

temperature, acetonitrile (1.5 mL) and *p*-TSA (42.8 mg, 0.23 mmol, 1.5 equiv.) were added and stirred at room temperature for a further 16 hrs. The crude product was purified by flash column chromatography (5-10% Et_2O in petrol) to afford *pyrrole* **4j** as colourless oil (52.7 mg, 77%).

^{1}H NMR (400 MHz, $CDCl_3$): δ 8.07 (2H, d, *J* 8.5, Ar-*H*), 7.50 (2H, d, *J* 8.5, Ar-*H*), 7.33 (1H, dd, *J* 8.0, 7.5, Ar-*H*), 7.04-6.97 (2H, m, Ar-*H*), 6.36 (1H, d, *J* 3.5, NCC*H*), 6.18 (1H, d, *J* 3.5, NCC*H*), 3.98 (3H, s, CO_2CH_3), 3.96 (3H, s, $ArOCH_3$), 2.26 (3H, s, SCH_3), 1.16 (9H, s, $CO_2C(CH_3)_3$)); ^{13}C NMR (101 MHz, $CDCl_3$): δ 159.6, 140.1, 139.2, 134.4, 130.4, 130.0, 129.7, 129.1, 128.3, 128.2, 123.4, 123.0, 120.8, 113.7, 112.7, 110.8, 83.9, 56.0, 52.1, 27.2, 17.9; IR: ν_{max} (neat)/cm^{-1} 2979, 2838, 1607, 1275, 1111, 994; LRMS (ESI$^+$): *m/z* 454 ([M+H]$^+$, 10%), 476 ([M+Na]$^+$, 100%); HRMS (ESI$^+$) found 454.13187 [M+H]$^+$, $C_{25}H_{28}O_5NS^+$ requires 454.16827.

2-Methoxy-6-{5-[2-(methylthio)-5-(trifluoromethyl)phenyl]-1*H*-pyrrol-2-yl}pyridine, 4k

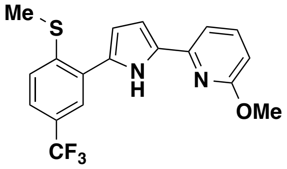

Prepared according to general procedure **G** using 2-(methylthio)-5-(trifluoromethyl)benzaldehyde **1n** (33.0 mg, 0.15 mmol, 1.0 equiv.), propargylic amine (*tert*-butyl [1-(6-methoxypyridin-2-yl)prop-2-yn-1-yl]carbamate **2m**) (59 mg, 0.195 mmol, 1.3 equiv.), $[Rh(nbd_2)]BF_4$ (4.1 mg, 7.5 mol%) and PNP(Cy) (5.2 mg, 7.5 mol%) in acetone (60 μL, 1 M). After stirring for 16 hrs at room temperature, acetonitrile (1.5 mL) and *p*-TSA (42.8 mg, 0.23 mmol, 1.5 equiv.) were added and stirred at room temperature for a further 4 hrs. The crude product was purified by flash column chromatography (5-10% Et_2O in petrol) to afford *pyrrole* **4k** as pale brown oil (41.0 mg, 74%).

^{1}H NMR (400 MHz, $CDCl_3$): δ 10.42 (1H, br s, N*H*), 7.76 (1H, td, *J* 1.5, 0.5, Ar-*H*), 7.59 (1H, dd, *J* 8.0, 7.5, Ar-*H*), 7.50-7.48 (1H, m, Ar-*H*), 7.44 (1H, d, *J* 8.5, Ar-*H*), 7.21 (1H, dd, *J* 7.5, 0.5, Ar-*H*), 6.79 (1H, dd, *J* 4.0, 2.5, NCC*H*), 6.66 (1H, dd, *J* = 4.0, 2.5, NCC*H*), 6.58 (1H, dd, *J* 8.0, 0.5, Ar-*H*), 4.03 (3H, s, OCH_3), 2.52 (3H, s, SCH_3); ^{13}C NMR (101 MHz, $CDCl_3$): δ 163.5, 147.6, 139.6, 139.3, 132.6, 132.0, 130.2, 127.9 (q, $^2J_{CF}$ 32.5), 127.7, 125.3 (q, $^3J_{CF}$ 3.5), 124.1(q, $^1J_{CF}$ 272.0), 123.5 (q, $^3J_{CF}$ 3.5), 112.0, 110.8, 108.0, 107.6, 53.3, 16.5; ^{19}F NMR (377 MHz, $CDCl_3$): δ -62.5; IR: ν_{max} (neat)/cm^{-1} 3429, 2923, 1573, 1471, 1423, 1374, 1328, 1260,1096, 907; LRMS (ESI$^+$): *m/z* 365 ([M+H]$^+$, 100%); HRMS (ESI$^+$) found 365.09313 [M+H]$^+$, $C_{18}H_{16}ON_2F_3S^+$ requires 365.09299.

***tert*-Butyl 2-[2-fluoro-6-(methylthio)phenyl]-3-hexyl-5-phenyl-1*H*-pyrrole-1-carboxylate, 4l**

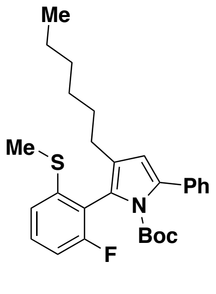

Prepared according to general procedure **G** using 2-fluoro-6-(methylthio)benzaldehyde **1j** (25.5 mg, 0.15 mmol, 1.0 equiv.), propargylic amine (*tert*-butyl (1-phenylnon-2-yn-1-yl)carbamate **2p**) (70.9 mg, 0.23 mmol, 1.3 equiv.), [Rh(nbd$_2$)]BF$_4$ (2.8 mg, 5 mol%) and dppm (2.9 mg, 5 mol%) in acetone (55 μL, 1 M). After stirring for 2 hrs at room temperature, acetonitrile (1.5 mL) and *p*-TSA (43 mg, 0.23 mmol, 1.5 equiv.) were added and stirred at room temperature for a further 4 hrs. The crude product was purified by flash column chromatography (10% Et$_2$O in petrol) to afford the *pyrrole* **4l** as colourless oil (34.0 mg, 50%).

^{1}H NMR (400 MHz, CDCl$_3$): δ 7.46-7.43 (2H, m, Ar-*H*), 7.41-7.35 (3H, m, Ar-*H*), 7.34-7.30 (1H, m, Ar-*H*), 7.02 (1H, d, *J* 8.0, Ar-*H*), 6.94 (1H, app td, *J* 8.5, 1.0, Ar-*H*), 6.29 (1H, s, NCC*H*), 2.42 (3H, s, SC*H$_3$*), 2.28-2.18 (2H, m, CH$_3$(CH$_2$)$_4$C*H$_2$*), 1.56-1.49 (2H, m, CH$_3$(CH$_2$)$_3$C*H$_2$*), 1.25 (6H, app td, *J* 10.5, 5.5, CH$_3$(C*H$_2$*)$_3$(CH$_2$)$_2$), 1.15 (9H, s, CO$_2$C(C*H$_3$*)$_3$), 0.87 (3H, t, *J* 7.0, C*H$_3$*(CH$_2$)$_5$); ^{13}C NMR (101 MHz, CDCl$_3$): δ 162.2, 159.7, 149.1, 143.6, 135.4 (d, $^1J_{CF}$ 190.0), 129.7 (d, $^2J_{CF}$ 19.5), 128.5, 127.7, 126.8, 122.3, 120.5 (d, $^2J_{CF}$ 19.0), 119.2 (d, $^3J_{CF}$ 8.5), 118.2, 114.2, 111.2 (d, $^3J_{CF}$ 8.5), 82.8, 31.6, 29.8, 29.0, 27.1, 25.9, 22.6, 15.3, 14.1; ^{19}F NMR (377 MHz, CDCl$_3$): δ -112.3; IR: ν_{max} (neat)/cm^{-1} 2925, 1743, 1605, 1482, 1306, 1153, 989; LRMS (ESI$^+$): *m/z* 468 ([M+H]$^+$, 100%), 490 ([M+Na]$^+$, 50%); HRMS (ESI$^+$) found 468.23627 [M+H]$^+$, C$_{28}$H$_{35}$O$_2$NFS$^+$ requires 468.23670.

***tert*-Butyl-2-(4-Bromophenyl)-5-[2-(methylthio)-5-(trifluoromethyl)-phenyl]-1*H*-pyrrole-1-carboxylate, 4m**

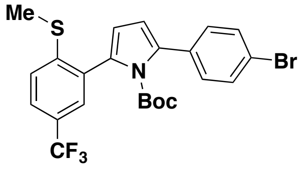

Prepared according to general procedure **G** using 2-(methylthio)-5-(trifluoromethyl)benzaldehyde **1n** (33 mg, 0.15 mmol, 1.0 equiv.), propargylic amine (*tert*-Butyl [1-(4-bromophenyl)prop-2-yn-1-yl]carbamate **2k**) (60.5 mg, 0.195 mmol, 1.3 equiv.), [Rh(nbd$_2$)]BF$_4$ (2.8 mg, 5 mol%) and PNP(Cy) (3.2 mg, 5

mol%) in acetone (57 μL, 1 M). After stirring for 2 mins at room temperature, acetonitrile (1.5 mL) and *p*-TSA (51.0 mg, 0.30 mmol, 2.0 equiv.) were added and stirred at room temperature for a further 3 hrs. The crude product was purified by flash column chromatography (5-10% Et_2O in petrol) to afford *pyrrole* **4m** as a waxy white sold (50.1 mg, 81%).

^{1}H NMR (400 MHz, $CDCl_3$): δ 7.62 (1H, ddd, *J* 8.5, 2.0, 0.5, Ar-*H*), 7.54-7.51 (3H, m, Ar-*H*), 7.30 (3H, app dq, *J* 9.0, 3.0, Ar-*H*), 6.32 (1H, d, *J* 3.5, NCC*H*), 6.27 (1H, d, *J* 3.5, NCC*H*), 2.45 (3H, s, SCH_3), 1.14 (9H, s, $CO_2C(CH_3)_3$); ^{13}C NMR (101 MHz, $CDCl_3$): δ 148.7, 145.3, 135.5, 133.7, 133.0, 132.1, 130.9, 130.4, 127.1 (q, $^3J_{CF}$ 3.5), 126.3 (q, $^2J_{CF}$ 33.0), 125.1 (q, $^3J_{CF}$ 3.5), 124.1 (q, $^3J_{CF}$ 262.0), 123.4, 121.4, 113.7, 113.3, 83.9, 27.1, 14.9; ^{19}F NMR (377 MHz, $CDCl_3$): δ -62.1; IR: ν_{max} (neat)/cm^{-1} 2943, 1749, 1609, 1475, 1369, 1171, 1121, 991, 792; LRMS (ESI$^+$): *m/z* 512 ([^{79}Br] [M+H]$^+$, 40%), 514 ([^{81}Br] [M+H]$^+$, 50%), 534 ([^{79}Br] [M+Na]$^+$, 90%), 536 ([^{81}Br] [M+Na]$^+$, 100%); HRMS (ESI$^+$) found 534.03119 [[^{79}Br] M+Na]$^+$, 536.02904 [[^{81}Br] M+Na]$^+$, $C_{23}H_{21}{}^{79}BrNO_2F_3SNa^+$ requires 534.03207, $C_{23}H_{21}{}^{81}BrNO_2SF_3Na^+$ requires 536.03002; mp: 88-89 °C (Et_2O/petrol).

***tert*-Butyl 2-[4-bromo-2-(methylthio)phenyl]-5-(thiophen-2-yl)-1*H*-pyrrole-1-carboxylate, 4n**

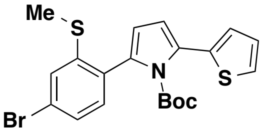

Prepared according to general procedure **G** using 4-bromo-2-(methylthio)benzaldehyde[3] **1e** (34.7 mg, 0.15 mmol, 1.0 equiv.), propargylic amine (*tert*-butyl [1-(thiophen-2-yl)prop-2-yn-1-yl]carbamate **2i**) (46.3 mg, 0.195 mmol, 1.3 equiv.), [Rh(nbd$_2$)]BF_4 (2.8 mg, 5 mol%) and PNP(Cy) (3.2 mg, 5 mol%) in acetone (69 μL, 1 M). After stirring for 2 hrs at room temperature, acetonitrile (1.5 mL) and *p*-TSA (42.8 mg, 0.23 mmol, 1.5 equiv.) were added and stirred at room temperature for a further 12 hrs. The crude product was purified by flash column chromatography (5-10% Et_2O in petrol) to afford *pyrrole* **4n** as white solid (49.2 mg, 73%).

^{1}H NMR (400 MHz, $CDCl_3$): δ 7.36-7.32 (3H, m, Ar-*H*), 7.16-7.13 (2H, m, Ar-*H*), 7.07 (1H, dd, *J* 5.0, 3.5, SCHC*H*), 6.40 (1H, d, *J* 3.5, NCC*H*), 6.21 (1H, d, *J* 3.5, NCC*H*), 2.41 (3H, s, SCH_3), 1.21 (9H, s, $CO_2C(CH_3)_3$); ^{13}C NMR (101 MHz, $CDCl_3$): δ 148.8, 142.2, 134.9, 132.7, 132.4, 131.7, 128.4, 127.5, 127.2, 126.7, 126.6, 125.5, 122.5, 114.6, 113.3, 83.8, 27.2, 15.3; IR: ν_{max} (neat)/cm^{-1} 2978, 1747, 1453, 1368, 1299, 1145, 1080, 791; LRMS (ESI$^+$): *m/z* 472 ([^{79}Br] [M+Na]$^+$, 95%), 474 ([^{81}Br] [M+Na]$^+$, 100%); HRMS (ESI$^+$) found 450.01863 [[^{79}Br] M+H]$^+$, 452.01656 [[^{81}Br]

M+H]+, $C_{20}H_{21}{}^{79}BrNO_2S_2{}^+$ requires 450.01916, $C_{20}H_{21}{}^{81}BrNO_2S_2{}^+$ requires 452.01711; mp: 85-86 °C (Et_2O/petrol).

tert-Butyl 2-[4-bromo-2-(methylthio)phenyl]-1_H_-pyrrole-1-carboxylate, 4o

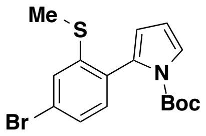

Prepared according to general procedure **G** using 4-bromo-2-(methylthio)benzaldehyde[3] **1e** (34.6 mg, 0.15 mmol, 1.0 equiv.), *N*-Boc-propargyl amine **2a** (30.2 mg, 0.195 mmol, 1.3 equiv.), $[Rh(nbd_2)]BF_4$ (2.8 mg, 5 mol%) and PNP(Cy) (3.2 mg, 5 mol%) in acetone (80 μL , 1 M). After stirring for 16 hrs at room temperature, acetonitrile (1.5 mL) and *p*-TSA (42.8 mg, 0.23 mmol, 1.5 equiv.) were added and stirred at room temperature for a further 3 hrs. The crude product was purified by flash column chromatography (5-10% Et_2O in petrol) to afford *pyrrole* **4o** as an off-white solid (42.6 mg, 80%).

^{1}H NMR (400 MHz, $CDCl_3$): δ 7.43 (1H, dd, *J*, 3.5, 2.0, NC*H*), 7.31-7.30 (2H, m, Ar-*H*), 7.1 (1H, d, *J* 8.5, Ar-*H*), 6.30 (1H, app t, *J* 3.5, NCC*H*), 6.17 (1H, dd, *J* 3.5, 2.0, NCHC*H*), 2.39 (3H, s, SC*H₃*), 1.33 (9H, s, $CO_2C(CH_3)_3$); ^{13}C NMR (101 MHz, $CDCl_3$): δ 148.9, 142.6, 132.6, 131.5, 127.1, 126.8, 126.3, 122.4, 122.0, 114.9, 110.7, 83.5, 27.5, 15.4; IR: ν_{max} (neat)/cm^{-1} 2923, 1740, 1493, 1449, 1399, 1370, 1338, 1312, 1257, 1150, 1082; LRMS (ESI+): *m/z* 368 ([^{79}Br] [M+H]+, 25%), 370 ([^{81}Br] [M+H]+, 30%), 390 ([^{79}Br] [M+Na]+, 95%), 392 ([^{81}Br] [M+H]+, 100%); HRMS (ESI+) found 390.0131 [[^{79}Br] [M+Na]+], 392.0110 [[^{81}Br] [M+Na]+], $C_{16}H_{18}{}^{79}BrNO_2SNa^+$ requires 390.0134, $C_{16}H_{18}{}^{81}BrNO_2SNa^+$ requires 392.0114; mp: 84-90 °C (Et_2O/petrol).

tert-Butyl 2-[4-bromo-2-(methylthio)phenyl]-5-cyclopropyl-3-(hydroxymethyl)-1_H_-pyrrole-1-carboxylate, 4p

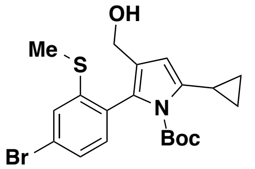

Prepared according to general procedure **G** using 4-bromo-2-(methylthio)benzaldehyde[3] **1e** (27.7 mg, 0.12 mmol, 1.0 equiv.), propargylic amine (*tert*-butyl [3-(benzyloxy)-1-cyclopropylprop-2-yn-1-yl]carbamate **2r**) (48.3 mg, 0.15 mmol, 1.3 equiv.), $[Rh(nbd_2)]BF_4$ (2.2 mg, 5 mol%) and dppm (2.3 mg, 5 mol%) in acetone (44 μL, 1 M). After stirring for 16 hrs at room temperature, acetonitrile (1.5 mL) and *p*-TSA (46.0 mg, 0.24 mmol,

2.0 equiv.) were added and stirred at room temperature for a further 3 hrs. The crude product was purified by flash column chromatography (5-10% Et_2O in petrol) to afford *pyrrole* **4p** as colourless oil (32.0 mg, 61%).

^{1}H NMR (400 MHz, $CDCl_3$): δ 7.54 (1H, d, *J* 2.0, Ar-*H*), 7.26 (1H, dd, *J* 8.5, 2.0, Ar-*H*), 6.99 (1H, d, *J* 8.5, Ar-*H*), 5.77 (1H, s, NCC*H*), 3.66 (2H, s, NCCCH_2OH), 2.14 (1H, ttd, *J* 8.5, 5.5, 1.0, NC(C*H*(CH_2)$_2$)), 1.58 (3H, s, SCH_3), 1.56 (9H, s, CO_2C(CH_3)$_3$), 0.91 (1H, dt, *J* 5.5, 2.0, NC(CHC$H_2$$CH_2$)), 0.69-0.65 (2H, m, NCCH(CH_2CH_2)); ^{13}C NMR (101 MHz, $CDCl_3$): δ 150.3, 141.0, 132.5, 130.9, 129.0, 128.1, 127.2, 125.4, 123.2, 118.5, 106.9, 84.5, 29.7, 27.7, 25.6, 9.3, 6.9; IR: ν_{max} (neat)/cm^{-1} 3285 (br), 2922, 1688, 1557, 1433, 1323, 1156, 1093; LRMS (ESI$^+$): *m/z* 460 ([^{79}Br] [M+Na]$^+$, 80%), 462 ([^{81}Br] [M+Na]$^+$, 75%), 492 ([^{79}Br] [M+MeOH+Na]$^+$, 100%), 494 ([^{81}Br] [M+MeOH+Na]$^+$, 90%); HRMS (ESI$^+$) found 492.00881 [[^{79}Br] [M+MeOH+Na]$^+$], 494.00654 [[^{81}Br] [M+MeOH+Na]$^+$], $C_{21}H_{28}{}^{79}BrNO_4SNa^+$ requires 492.00815, $C_{21}H_{28}{}^{81}BrNO_4SNa^+$ requires 494.00794.

***tert*-Butyl 2-[2-(methylthio)ethyl]-5-phenyl-1*H*-pyrrole-1-carboxylate, 4q**

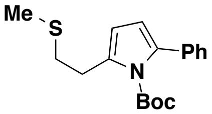

Prepared according to general procedure **G** using 3-(methylthio)propionaldehyde **1b** (15 μL, 0.15 mmol, 1.0 equiv.), propargylic amine (*tert*-butyl (1-phenylprop-2-yn-1-yl)carbamate **2d**) (45.1 mg, 0.195 mmol, 1.3 equiv.), [Rh(nbd$_2$)]BF_4 (2.8 mg, 5 mol%) and PNP(Cy) (3.2 mg, 5 mol%) in acetone (75 μL, 1 M). After stirring for 16 hrs at room temperature, acetonitrile (1.5 mL) and *p*-TSA (43.0 mg, 0.23 mmol, 1.5 equiv.) were added and stirred at room temperature for a further 6 hrs. The crude product was purified by flash column chromatography (5-10% Et_2O in petrol) to afford *pyrrole* **4q** as colourless oil (39.9 mg, 84%).

^{1}H NMR (400 MHz, $CDCl_3$): δ 7.40-7.36 (2H, m, Ar-*H*), 7.34-7.30 (3H, m, Ar-*H*), 6.15 (1H, d, *J* 3.5, NC(Ph)C*H*), 6.10 (1H, dt, *J* 3.5, 1.0, NC($CH_2CH_2SCH_3$)C*H*), 3.20 (2H, t, *J* 7.5, NCCH_2), 2.88-2.84 (2H, m, SCH_2), 2.20 (3H, s, SCH_3), 1.28 (9H, s, CO_2C(CH_3)$_3$); ^{13}C NMR (101 MHz, $CDCl_3$): δ 150.0, 135.5, 135.4, 135.3, 128.4, 127.8, 126.7, 112.2, 110.4, 83.6, 33.9, 29.1, 27.3, 15.6; IR: ν_{max} (neat)/cm^{-1} 2979, 1606, 1526, 1444, 1369, 1144, 908; LRMS (ESI$^+$): *m/z* 318 ([M+H]$^+$, 40%), 340 ([M+Na]$^+$, 100%); HRMS (ESI$^+$) found 340.13413 [M+Na]$^+$, $C_{18}H_{23}O_2NSNa^+$ requires 340.13417.

tert-Butyl 2-(2-bromophenyl)-5-[2-(methylthio)ethyl]-1*H*-pyrrole-1-carboxylate, 4r

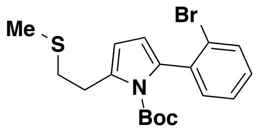

Prepared according to general procedure **G** using 3-(methylthio)propionaldehyde **1b** (15 μL, 0.15 mmol, 1.0 equiv.), propargylic amine (*tert*-butyl [1-(2-bromophenyl)prop-2-yn-1-yl]carbamate **2j**) (60.5 mg, 0.195 mmol, 1.3 equiv.), [Rh(nbd$_2$)]BF_4 (2.8 mg, 5 mol%) and PNP(Cy) (3.2 mg, 5 mol%) in acetone (75 μL, 1 M). After stirring for 16 hrs at room temperature, acetonitrile (1.5 mL) and *p*-TSA (51.0 mg, 0.30 mmol, 2.0 equiv.) were added and stirred at room temperature for a further 5 hrs. The crude product was purified by flash column chromatography (5-10% Et_2O in petrol) to afford *pyrrole* **4r** as colourless oil (46.2 mg, 78%).

^{1}H NMR (400 MHz, $CDCl_3$): δ 7.61-7.58 (1H, m, Ar-*H*), 7.36-7.31 (2H, m, Ar-*H*), 7.21 (1H, ddd, *J* 8.0, 6.5, 3.0, Ar-*H*), 6.12 (1H, dt, *J* 3.5, 1.0, NC($CH_2CH_2SCH_3$)C*H*), 6.07 (1H, d, *J* 3.5, NCC*H*), 3.23 (2H, t, *J* 7.5, NCCH_2), 2.85 (2H, t, *J* 7.5, SCH_2), 2.19 (3H, s, SCH_3), 1.23 (9H, s, $CO_2C(CH_3)_3$); ^{13}C NMR (101 MHz, $CDCl_3$): δ 149.4, 137.5, 135.4, 133.1, 131.9, 131.4, 128.8, 126.9, 125.1, 112.8, 110.5, 83.3, 33.8, 29.6, 27.3, 15.7; IR: ν_{max} (neat)/cm^{-1} 2921, 1742, 1369, 1312, 1148, 908; LRMS (ESI$^+$): *m/z* 418 ([^{79}Br] [M+Na]$^+$, 100%), 420 ([^{81}Br] [M+Na]$^+$, 60%); HRMS (ESI$^+$) found 418.04401 [[^{79}Br] M+Na]$^+$, 420.04180 [[^{81}Br] M+Na]$^+$, $C_{18}H_{22}{}^{79}BrNO_2SNa^+$ requires 418.0.4468, $C_{18}H_{22}{}^{81}BrNO_2SNa^+$ requires 420.04100.

tert-Butyl 2-[4-(methoxycarbonyl)phenyl]-5-[2-(methylthio)propyl]-1*H*-pyrrole-1-carboxylate , 4s

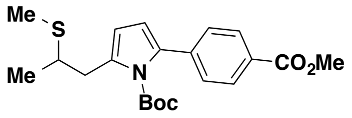

Prepared according to general procedure **G** using 3-(methylthio)butanal **1c** (18 μL, 0.15 mmol, 1.0 equiv.), propargylic amine (*tert*-butyl (1-phenylprop-2-yn-1-yl)carbamate **2l**) (56.0 mg, 0.195 mmol, 1.3 equiv.), [Rh(nbd$_2$)]BF_4 (2.8 mg, 5 mol%) and PNP(Cy) (3.2 mg, 5 mol%) in acetone (76 μL, 1 M). After stirring for 4 hrs at room temperature, acetonitrile (1.5 mL) and *p*-TSA (42.8 mg, 0.23 mmol, 1.5 equiv.) were added and stirred at room temperature for a further 4 hrs. The crude product was purified by flash column chromatography (5-10% Et_2O in petrol) to afford *pyrrole* **4s** as colourless oil (44.0 mg, 75%).

^{1}H NMR (400 MHz, $CDCl_3$): δ 8.05-8.03 (2H, m, Ar-*H*), 7.38-7.36 (2H, m, Ar-*H*), 6.22 (1H, d, *J* 3.5, NCC*H*), 6.10 (1H, d, *J* 3.5, NCC*H*), 3.96 (3H, s, CO_2CH_3), 3.25 (1H, dd, *J* 14.5, 6.0, $SCH(CH_3)CH_aH_b$), 3.10-3.02 (1H, m, SC*H*(CH_3)), 2.93 (1H, dd, *J* 14.5, 8.0, $SCH(CH_3)CH_aH_b$), 2.14 (3H, s, SCH_3), 1.33 (3H, d, *J* 7.0, $SCH(CH_3)$), 1.28 (9H, s, $CO_2C(CH_3)_3$); ^{13}C NMR (101 MHz, $CDCl_3$): δ 167.4, 150.3, 140.2, 136.2, 134.6, 129.6, 128.4, 128.3, 113.8, 112.5, 84.6, 52.5, 41.3, 36.4, 27.7, 20.9, 14.0; IR: ν_{max} (neat)/cm^{-1} 2923, 1721, 1609, 1453, 1308, 1276, 1144, 909; LRMS (ESI$^+$): *m/z* 412 ([M+Na]$^+$, 100%); HRMS (ESI$^+$) found 390.17340 [M+H]$^+$, $C_{21}H_{28}O_4NS^+$ requires 390.17336.

tert-Butyl 3-hexyl-2-[2-(methylthio)cyclohex-1-en-1-yl]-5-phenyl-1*H*-pyrrole-1-carboxylate, 4t

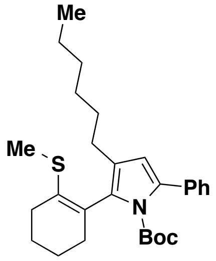

Prepared according to general procedure **G** using 2-(methylthio)cyclohex-1-enecarbaldehyde,[2] **1d** (23.5 mg, 0.15 mmol, 1.0 equiv.), propargylic amine (*tert*-butyl (1-phenylnon-2-yn-1-yl)carbamate **2p**) (71.0 mg, 0.195 mmol, 1.3 equiv.), [Rh(nbd$_2$)]BF_4 (2.8 mg, 5 mol%) and dppm (2.9 mg, 5 mol%) in acetone (56 μL, 1.0 M). After stirring for 4 hrs at room temperature, acetonitrile (1.5 mL) and *p*-TSA (42.8 mg, 0.23 mmol, 1.5 equiv.) were added and stirred at room temperature for a further 16 hrs. The crude product was purified by flash column chromatography (5-10% Et_2O in petrol) to afford *pyrrole* **4t** as colourless oil (42.8 mg, 63%).

^{1}H NMR (400 MHz, $CDCl_3$): δ 7.37-7.32 (4H, m, Ar-*H*), 7.28-7.25 (1H, m, Ar-*H*), 6.14 (1H, s, NCC*H*), 2.40 (2H, app d, *J* 3.0, $CH_2(CH_2)_4CH_3$), 2.30 (2H, ddd, *J* 8.5, 7.0, 3.0, $SCCH_2$), 2.14 (3H, s, SCH_3), 1.93-1.88 (2H, m, $CH_2(CH_2)_3CH_3$), 1.78-1.75 (2H, m, $SCCCH_2$), 1.63-1.46 (4H, m, $SCCH_2(CH_2)_2$), 1.39-1.32 (6H, m, $(CH_2)_3CH_3$), 1.31 (9H, s, $CO_2C(CH_3)_3$), 0.91 (3H, t, *J* 7.0, $CH_3(CH_2)_5$); ^{13}C NMR (101 MHz, $CDCl_3$): δ 149.6, 135.1, 134.3, 133.0, 132.5, 128.4, 128.2, 127.6, 126.4, 123.6, 113.6, 82.6, 32.8, 31.8, 30.1, 29.4, 29.3, 27.4, 25.9, 23.4, 22.7, 22.6, 22.5, 14.2; IR: ν_{max} (neat)/cm^{-1} 2927, 1742, 1601, 1488, 1467, 1304, 1259, 1155, 729; LRMS (ESI$^+$): *m/z* 454 ([M+H]$^+$, 100%), 476 ([M+Na]$^+$, 90%); HRMS (ESI$^+$) found 476.25847 [M+Na]$^+$, $C_{28}H_{39}O_2NSNa^+$ requires 476.25937.

tert-Butyl 2-cyclopropyl-5-[3-(methylthio)thiophen-2-yl]-1H-pyrrole-1-carboxylate, 4u

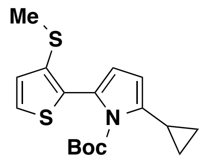

Prepared according to general procedure **G** using 3-(methylthio)thiophene-2-carbaldehyde **1m** (16.3 mg, 0.103 mmol, 1.0 equiv.), propargylic amine (*tert*-butyl (1-cyclopropylprop-2-yn-1-yl)carbamate **2n**) (26.2 mg, 0.134 mmol, 1.3 equiv.), [Rh(nbd$_2$)]BF$_4$ (1.9 mg, 5 mol%) and PNP(Cy) (2.2 mg, 5 mol%) in acetone (61 μL, 1 M). After stirring for 16 hrs at room temperature, acetonitrile (1.5 mL) and *p*-TSA (mg, 0.15 mmol, 1.0 equiv.) were added and stirred at room temperature for a further 4 hrs. The crude product was purified by flash column chromatography (5-10% Et$_2$O in petrol) to afford *pyrrole* **4u** as colourless oil (28.8 mg, 79%).

^{1}H NMR (400 MHz, CDCl$_3$): δ 7.29 (1H, d, *J* 5.5, SC*H*), 7.02 (1H, d, *J* 5.5, SCH$_3$CC*H*), 6.18 (1H, d, *J* 3.5, NCC*H*), 5.92 (1H, dd, *J* 3.5, 1.0, NC(CH(CH$_2$)$_2$)C*H*), 2.32 (3H, s, SC*H$_3$*), 2.29-2.25 (1H, m, NC(C*H*(CH$_2$CH$_2$))), 1.40 (9H, s, CO$_2$C(C*H$_3$*)$_3$), 0.90 (2H, dt, *J* 6.5, 2.0, NC(CH(C*H$_2$*CH$_2$))), 0.69-0.67 (2H, m, NC(CH(CH$_2$*CH$_2$*))); ^{13}C NMR (101 MHz, CDCl$_3$): δ 149.5, 140.4, 132.7, 132.0, 128.6, 124.5, 124.2, 115.0, 108.1, 83.2, 27.5, 17.9, 9.9, 7.0; IR: ν_{max} (neat)/cm^{-1} 3086, 1742, 1369, 1297, 1135, 1090, 971; LRMS (ESI$^+$): *m/z* 336 ([M+H]$^+$, 100%), 358 ([M+Na]$^+$, 90%); HRMS (ESI$^+$) found 358.09025 [M+Na]$^+$, $C_{17}H_{21}O_2NS_2Na^+$ requires 358.09059.

tert-Butyl 2-(furan-2-yl)-5-[3-(methylthio)thiophen-2-yl]-1H-pyrrole-1-carboxylate, 4v

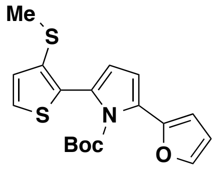

Prepared according to general procedure **G** using 3-(methylthio)thiophene-2-carbaldehyde **1m** (23.7 mg, 0.15 mmol, 1.0 equiv.), propargylic amine (*tert*-butyl [1-(furan-2-yl)prop-2-yn-1-yl]carbamate **2f**) (43.1 mg, 0.195 mmol, 1.3 equiv.), [Rh(nbd$_2$)]BF$_4$ (2.8 mg, 5 mol%) and PNP(Cy) (3.2 mg, 5 mol%) in acetone (83 μL, 1 M). After stirring for 3 hrs at room temperature, acetonitrile (1.5 mL) and *p*-TSA (42.8 mg, 0.23 mmol, 1.5 equiv.) were added and stirred at room temperature for a further 16 hrs. The crude product was purified by flash column chromatography (5-10% Et$_2$O in petrol) to afford *pyrrole* **4v** as a light brown oil (32.4 mg, 60%).

^{1}H NMR (400 MHz, $CDCl_3$): δ 7.49 (1H, dd, *J* 2.0, 1.0, OC*H*), 7.36 (1H, d, *J* 5.5, (CH_3)SCC*H*), 7.06 (1H, d, *J* 5.5, SC*H*), 6.55 (1H, dd, *J* 3.5, 1.0, OCHC*H*), 6.48 (1H, d, *J* 3.5, NC(CS)C*H*), 6.47 (1H, d, J 3.5, OCC*H*) 6.36 (1H, d, *J* 3.5, NC(CO)C*H*), 2.36 (s, SC*H₃*), 1.32 (9H, s, $CO_2C(CH_3)_3$); ^{13}C NMR (101 MHz, $CDCl_3$): δ 148.7, 146.8, 141.9, 133.3, 131.0, 128.6, 126.8, 126.4, 125.4, 115.5, 113.6, 111.0, 108.7, 83.9, 27.3, 18.0 ; IR: ν_{max} (neat)/cm^{-1} 2923, 2853, 1752, 1458, 1393, 1328, 1258, 1104, 1011, 843, 684; LRMS (ESI$^+$): *m/z* 362 ([M+H]$^+$, 75%), 384 ([M+Na]$^+$, 100%); HRMS (ESI$^+$) found 362.08800 [M+H]$^+$, $C_{18}H_{20}O_3NS^+$ requires 362.08791.

tert-Butyl 2-[2-(dimethylamino)phenyl]-5-methyl-1*H*-pyrrole-1-carboxylate, 4w

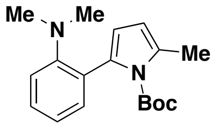

Prepared according to general procedure **G** using 2-(dimethylamino)benzaldehyde[5] **1g** (22.4 mg, 0.15 mmol, 1.0 equiv.), propargylic amine (*tert*-butyl but-3-yn-2-ylcarbamate **2b**) (33.0 mg, 0.195 mmol, 1.3 equiv.), [Rh(nbd$_2$)]BF_4 (2.8 mg, 5 mol%) and PNP(Cy) (3.2 mg, 5 mol%) in acetone (95 μL, 1 M). After stirring for 3 hrs at room temperature, acetonitrile (1.5 mL) and *p*-TSA (142.6 mg, 0.75 mmol, 5.0 equiv.) were added and stirred at room temperature for a further 2 hrs. The crude product was purified by flash column chromatography (5-10% Et_2O in petrol) to afford *pyrrole* **4w** as pale yellow oil (33.1 mg, 73%).

^{1}H NMR (400 MHz, $CDCl_3$): δ 7.28 (1H, d, *J* 10.0, Ar-*H*), 7.25-7.23 (1H, m, Ar-*H*), 6.96 (1H, app td, *J* 7.5, 1.0, Ar-*H*), 6.91 (1H, dd, *J* 8.5, 1.0, Ar-*H*), 6.06 (1H, d, *J* 3.0, NC(Ar)C*H*), 5.99 (1H, dt, *J* 3.0, 1.0, NC(CH_3)C*H*), 2.51 (6H, s, $N(CH_3)_2$), 2.48 (3H, d, *J* 1.0, NC(CH_3)CH), 1.29 (9H, s, $CO_2C(CH_3)_3$); ^{13}C NMR (101 MHz, $CDCl_3$): δ 150.0, 133.2, 132.1, 130.6, 130.4, 128.0, 127.9, 120.8, 116.5, 111.4, 110.1, 82.5, 42.5, 27.5, 15.4; IR: ν_{max} (neat)/cm^{-1} 2922, 2870, 1677, 1599, 1478, 1450, 1260, 1162, 751; LRMS (ESI$^+$): *m/z* 301 ([M+H]$^+$, 100%); HRMS (ESI$^+$) found 301.19044 [M+H]$^+$, $C_{18}H_{25}O_2N_2^+$ requires 301.19105.

tert-Butyl 2-methyl-5-(2-(pyrrolidin-1-yl)phenyl)-1_H_-pyrrole-1-carboxylate, 4x

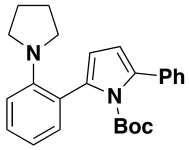

Prepared according to general procedure **G** using 2-(pyrrolidin-1-yl)benzaldehyde[5] **1h** (26.3 mg, 0.15 mmol, 1.0 equiv.), propargylic amine (*tert*-butyl (1-phenylprop-2-yn-1-yl)carbamate **2d**) (45.1 mg, 0.195 mmol, 1.3 equiv.), [Rh(nbd$_2$)]BF$_4$ (2.8 mg, 5 mol%) and PNP(Cy) (3.2 mg, 5 mol%) in acetone (79 μL, 1 M). After stirring for 16 hrs at room temperature, acetonitrile (1.5 mL) and *p*-TSA (43.8 mg, 0.23 mmol, 1.5 equiv.) were added and stirred at room temperature for a further 3 hrs. The crude product was purified by flash column chromatography (10% Et$_2$O in petrol) to afford *pyrrole* **4x** as pale yellow oil (40.2 mg, 69%).

^{1}H NMR (400 MHz, CDCl$_3$): δ 7.40-7.36 (4H, m, Ar-*H*), 7.35-7.30 (1H, m, Ar-*H*), 7.28-7.24 (2H, m, Ar-*H*), 6.83 (1H, app td, *J* 7.5, 1.5, Ar-*H*), 6.76 (1H, d, *J* 8.0, Ar-*H*), 6.27 (1H, d, *J* 3.5, NC(Ar)C*H*), 6.18 (1H, d, *J* 3.5, NC(Ar)C*H*), 2.95 (4H, t, *J* 6.0, -C*H*$_2$NC*H*$_2$-), 1.81-1.78 (4H, t, *J* 6.0, -C*H*$_2$CH$_2$NCH$_2$C*H*$_2$-), 1.14 (9H, s, CO$_2$C(C*H*$_3$)$_3$); ^{13}C NMR (101 MHz, CDCl$_3$): δ 149.5, 148.5, 135.3, 134.9, 134.2, 132.1, 128.6, 128.5, 127.8, 126.9, 122.1, 117.4, 114.0, 112.3, 112.0, 83.0, 49.4, 31.0, 27.0; IR: ν_{max} (neat)/cm^{-1} 2924, 2853, 1599, 1484, 1366, 1303, 1180, 1027, 978; LRMS (ESI$^+$): *m/z* 389 ([M+H]$^+$, 100%); HRMS (ESI$^+$) found 389.22244 [M+H]$^+$, $C_{25}H_{29}O_2N_2^+$ requires 389.22235.

8. Functionalising existing pyrroles

tert-Butyl 3-bromo-2-cyclopropyl-4-hexyl-5-[2-(methylthio)phenyl]-1_H_-pyrrole-1-carboxylate, 5a

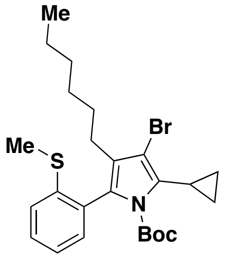

Pyrrole (*tert*-butyl 5-cyclopropyl-3-hexyl-2-[2-(methylthio)phenyl]-1*H*-pyrrole-1-carboxylate **4h**) (62.0 mg, 0.15 mmol, 1.0 equiv.), was dissolved in THF (1 mL) and cooled to -78 °C. *N*-Bromosuccinimide (53.4 mg, 0.3 mmol, 2.0 equiv.) in THF (1.2 mL) was added dropwise to the stirring solution of pyrrole. The reaction

mixture was allowed to warm to room temperature for 16 hrs. After which, sat. $NaHCO_{3\,(aq)}$ (2 mL) was added at room temperature. The product was extracted with EtOAc (3 × 5 mL) and washed with brine (10 mL). The organic layer was dried over $MgSO_4$, filtered and concentrated under reduced pressure. The crude product was purified by flash column chromatography (5% EtOAc in petrol) to yield brominated pyrrole **5a** as an off-white oil (64.0 mg, 87%).

^{1}H NMR (400 MHz, $CDCl_3$): δ 7.38-7.34 (1H, m, Ar-*H*), 7.20 (1H, d, *J* 8.0, Ar-*H*), 7.18-7.16 (2H, m, Ar-*H*), 2.37 (3H, s, S*CH*$_3$), 2.26-2.09 (2H, m, $CH_3(CH_2)_4C$*H*$_2$), 2.02-1.95 (1H, m, NCC*H*), 1.44-1.37 (2H, m, $CH_3(CH_2)_3C$*H*$_2$), 1.23 (9H, s, $CO_2C(C$*H*$_3)_3$), 1.21-1.16 (6H, m, $CH_3(C$*H*$_2)_3$), 1.04-0.92 (3H, m, NCCH(C*H*$_2$C*H*$_a$H$_b$)), 0.84 (3H, t, *J* 7.0, C*H*$_3(CH_2)_5$), 0.73-0.67 (1H, m, NCCH(CH_2CH_a*H*$_b$)); ^{13}C NMR (101 MHz, $CDCl_3$): δ 148.7, 140.5, 132.1, 131.2, 128.4, 127.0, 124.4, 124.1, 124.0, 123.9, 104.5, 82.8, 31.4, 29.6, 29.1, 27.2, 25.2, 22.5, 15.0, 14.1, 8.6, 7.8, 7.3; IR: ν_{max} (neat)/cm^{-1} 2925, 1744, 1577, 1459, 1335, 1155, 1078, 965; LRMS (ESI$^+$): *m/z* 492 ([^{79}Br] [M+H]$^+$, 95%), 494 ([^{81}Br] [M+H]$^+$, 100%), 514 ([^{79}Br] [M+Na]$^+$, 48%), 516 ([^{81}Br] [M+H]$^+$, 50%); HRMS (ESI$^+$) found 514.13841 [[^{79}Br] M+Na]$^+$, 516.13630 [[^{81}Br] M+Na]$^+$, $C_{25}H_{34}{}^{79}BrNO_2SNa^+$ requires 514.13858, $C_{25}H_{34}{}^{81}BrNO_2SNa^+$ requires 516.13654; mp: 99-101 °C (DCM/hexane).

1-[2-Cyclopropyl-4-hexyl-5-(2-(methylthio)phenyl]-1*H*-pyrrol-3-yl)ethan-1-one, 5b

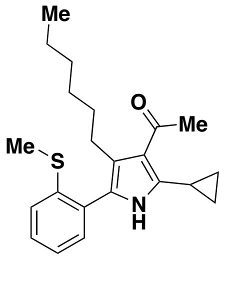

Prepared according to a procedure adapted from Frost *et. al.*[15] Pyrrole (*tert*-butyl 5-cyclopropyl-3-hexyl-2-[2-(methylthio)phenyl]-1*H*-pyrrole-1-carboxylate **4h**) (124 mg, 0.30 mmol, 1.0 equiv.) in nitromethane (0.7 mL) was added dropwise to a solution of indium (III) trifluoromethanesulfonate (16.9 mg, 0.03 mmol, 10 mol%) and lithium perchlorate (31.9 mg, 0.30 mmol, 1.0 equiv.) in nitromethane (0.1 mL) at 50 °C, followed by the addition of acetic anhydride (43 *μ*L, 0.45 mmol, 1.5 equiv.). The reaction mixture was stirred at 50 °C for 16 hrs and then quenched by the addition of water (2 mL). The product was extracted with DCM (3 × 5 mL) and washed with brine (10 mL). The organic layer was dried over $MgSO_4$, filtered and concentrated under reduced pressure. The crude product was purified by flash column chromatography (5-10% EtOAc in petrol) to yield acylated pyrrole **5b** as a white solid (84.0 mg, 79%).

^{1}H NMR (400 MHz, $CDCl_3$): δ 8.17 (1H, br s, N*H*), 7.35 (1H, ddd, *J* 8.0, 7.0, 1.5, Ar-*H*), 7.28-7.25 (2H, m, Ar-*H*), 7.20 (1H, app td, *J* 7.5, 1.5, Ar-*H*), 2.62 (3H, s, COC*H₃*), 2.35 (3H, s, SC*H₃*), 2.30 (1H, app dt, *J* 10.0, 4.0, NHCC*H*), 1.48 (2H, app ddd, *J* 10.0, 5.0, 2.0, $CH_3(CH_2)_4C\mathit{H_2}$), 1.26-1.20 (8H, m, $CH_3(C\mathit{H_2})_4$), 1.10-1.05 (2H, m, $NCCH(C\mathit{H_2}CH_2)$), 0.85-0.81 (5H, m, $C\mathit{H_3}(CH_2)_5$ and $NCCH(CH_2C\mathit{H_2})$; ^{13}C NMR (101 MHz, $CDCl_3$): δ 195.2, 139.0, 138.5, 131.5, 130.7, 128.5, 125.5, 125.07, 125.03, 124.7, 122.1, 31.6, 31.3, 31.1, 29.4, 25.7, 22.7, 15.3, 14.1, 10.3, 7.4; IR: ν_{max} (neat)/cm^{-1} 3249 (br), 2954, 2854, 1622, 1444, 1361, 908; LRMS (ESI^+): *m/z* 356 ($[M+H]^+$, 100%); HRMS (ESI^+) found 356.20450 $[M+H]^+$, $C_{22}H_{30}OSNNa^+$ requires 356.20426; mp: 88-89 °C (DCM/hexane).

3-Hexyl-2-[2-(methylthio)phenyl]-5-phenyl-1*H*-pyrrole, 5c

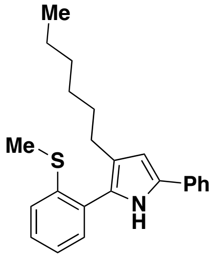

Pyrrole (*tert*-butyl 3-hexyl-2-[2-(methylthio)phenyl]-5-phenyl-1*H*-pyrrole-1-carboxylate **4g**) (30.6 mg, 0.07 mmol, 1.0 equiv.) was dissolved in DCM (2 mL). Trifluoroacetic acid (0.6 mL, 8.4 *µ*mol, 0.12 equiv.) was added to the pyrrole dropwise and stirred at room temperature for 2 hrs. The reaction mixture was diluted with DCM (5 mL) and neutralised with sat. $NaHCO_3$ $_{(aq.)}$. The aqueous layer was extracted with DCM (3 × 5 mL) and the combined organic extracts were washed with brine (10 mL), dried over $MgSO_4$, filtered and concentrated under reduced pressure. The crude product required no further purifications and afforded deprotected **5c** pyrrole as a colourless oil (23.3 mg, 98%).

^{1}H NMR (400 MHz, $CDCl_3$): δ 8.62 (1H, br s, N*H*), 7.55-7.52 (2H, m, Ar-*H*), 7.41-7.34 (5H, m, Ar-*H*), 7.26-7.20 (2H, m, Ar-*H*), 6.55 (1H, app d, *J* 3.0, NCC*H*), 2.51 (2H, t, *J* 8.0, $CH_3(CH_2)_4C\mathit{H_2}$), 2.38 (3H, s, SC*H₃*), 1.63 (2H, app t, *J* 7.5, $CH_3(CH_2)_3C\mathit{H_2}$), 1.37-1.25 (6H, m, $CH_3(C\mathit{H_2})_3$), 0.90 (3H, t, *J* 7.0, $C\mathit{H_3}(CH_2)_5$); ^{13}C NMR (101 MHz, $CDCl_3$): δ 138.2, 132.7, 131.9, 131.3, 131.1, 128.8, 128.0, 127.4, 126.1, 126.0, 124.9, 124.8, 123.5, 106.9, 31.7, 31.1, 29.2, 26.3, 22.7, 15.6, 14.1; IR: ν_{max} (neat)/cm^{-1} 3430 (br), 3057, 2954, 2854, 1606, 1377, 1201, 907; HRMS (FI^+) found 349.1862 $[M]^+$, $C_{23}H_{27}SN^+$ requires 349.1864.

9. Synthesis of pyrroles *via* a telescoped hydroacylation-Suzuki-cyclisation

General procedure H for the three component coupling synthesis of pyrroles as exemplified by the formation of *tert*-butyl 2-[4'-acetyl-4-(trifluoromethyl)-(1,1'-biphenyl)-2-yl]-5-phenyl-1*H*-pyrrole-1-carboxylate, 6a

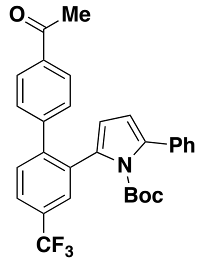

An oven-dried microwave vial was charged with [Rh(nbd)$_2$BF$_4$] (7.5 mg, 10 mol%), PNP(Cy) (4.2 mg, 5 mol%) and dcpm (4.1 mg, 5 mol%). Once under an inert atmosphere, they were dissolved in acetone (1 mL). Hydrogen gas was bubbled through the solution at room temperature for 1-2 mins in order to generate the active catalyst species. The hydrogen gas was purged using nitrogen gas, and this was bubbled through the catalyst to dryness. The dry catalyst was dissolved in acetone (96 μL, 1.0 M with respect to aldehyde) and this was transferred to a nitrogen-filled microwave vial containing 2-(methylthio)-5-(trifluoromethyl)benzaldehyde **1n** (44 mg, 0.20 mmol, 1.0 equiv.) and propargylic amine (*tert*-butyl (1-phenylprop-2-yn-1-yl)carbamate **2d**) (60.1 mg, 0.26 mmol, 1.3 equiv.). The reaction mixture was then stirred at 55 °C for 5 mins. It was then transferred to an oven-dried microwave vial charged with silver carbonate (55.2 mg, 0.20 mmol, 1.0 equiv.) and 4-acetylphenylboronic acid (49.2 mg, 0.30 mmol, 1.5 equiv.) using acetone (0.47 mL, 0.3 M with respect to aldehyde minus starting materials). The vessel was heated at 55 °C for a further 5 hrs, after which it was cooled to room temperature, opened to air, and filtered through a plug of silica with DCM (3 mL). *p*-TSA (76.1 mg, 0.40 mmol, 2.0 equiv) was added to the solution and stirred at room temperature until complete. After 6 hrs the solution was diluted with DCM (5 mL) and neutralised by the addition of sat. $NaHCO_3$ $_{(aq)}$ (10 mL) in a separatory funnel. The aqueous mixture was extracted with DCM (3 × 5 mL) and the combined organic extracts were washed with brine (10 mL) and dried over $MgSO_4$. The solvent was removed *in vacuo* to obtain the crude product and this was purified by flash column chromatography (5-10% EtOAc in petrol) to afford title *pyrrole* **6a** as a light brown solid (51.0 mg, 50%).

^{1}H NMR (400 MHz, $CDCl_3$): δ 7.96-7.93 (2H, m, Ar-*H*), 7.75-7.71 (2H, m, Ar-*H*), 7.58 (1H, d, *J* 8.0, Ar-*H*), 7.40-7.37 (2H, m, Ar-*H*), 7.33-7.32 (3H, m, Ar-*H*), 7.21-7.19 (2H, m, Ar-*H*), 6.17 (1H, d, *J* 3.5, NCC*H*), 6.14 (1H, d, *J* 3.5, NCC*H*), 2.64 (3H, s, COCH_3), 1.01 (9H, s, $CO_2C(CH_3)_3$); ^{13}C NMR (101 MHz, $CDCl_3$): δ 197.7, 148.7, 145.0, 143.6, 136.8, 135.8, 134.3, 134.2, 133.1, 129.9, 129.8 (q, $^{2}J_{CF}$ 31.5) 129.2, 129.0, 128.1, 127.7, 127.3, 127.2 (q, $^{3}J_{CF}$ 3.5), 124.3 (q, $^{3}J_{CF}$ 3.5), 123.9 (q, $^{1}J_{CF}$ 252.0), 114.7, 113.0, 83.8, 27.1, 26.7; ^{19}F NMR (377 MHz, $CDCl_3$): δ -62.3; IR: ν_{max} (neat)/cm^{-1} 2974, 2361, 2169, 1746, 1684, 1397; LRMS (ESI$^+$): *m/z* 528 ([M+Na]$^+$, 100%); HRMS (ESI$^+$) found 528.17477 [M+Na]$^+$, $C_{30}H_{26}O_3F_3NNa^+$ requires 528.17570; mp: 146-147 °C (EtOAc/petrol).

***tert*-Butyl 2-[5-bromo-4'-methoxy-(1,1'-biphenyl)-2-yl]-5-methyl-1*H*-pyrrole-1-carboxylate, 6b**

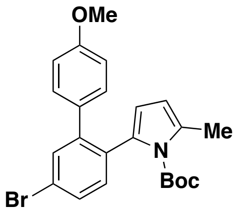

Prepared according to general procedure **H** using 4-bromo-2-(methylthio)benzaldehyde[3] **1e** (58 mg, 0.25 mmol, 1.0 equiv.), propargylic amine (*tert*-butyl but-3-yn-2-ylcarbamate **2b**) (56 mg, 0.33 mmol, 1.3 equiv.), [Rh(nbd$_2$)]BF_4 (9.3 mg, 10 mol%) , PNP(Cy) (5.3 mg, 5 mol%) and dcpm (5.1 mg, 5 mol%) in acetone (0.14 mL, 1 M). After 5 mins at 55 °C it was transferred to silver carbonate (69 mg, 0.25 mmol, 1.0 equiv.) and 4-methoxyphenylboronic acid (50 mg, 0.33 mmol, 1.5 equiv.) using acetone (0.46 mL). After stirring for 4 hrs at 55 °C the reaction was filtered and stirred with DCM (4 mL) and *p*-TSA (48 mg, 0.25 mmol, 1.0 equiv.) at room temperature for a further 3 hrs. The crude product was purified by flash column chromatography (10-40% DCM in petrol) to afford *pyrrole* **6b** as an off-white solid (43.8 mg, 43%).

^{1}H NMR (400 MHz, $CDCl_3$): δ 7.54 (1H, d, *J* 2.0, Ar-*H*), 7.45 (1H, dd, *J* 8.0, 2.0, Ar-*H*), 7.21 (1H, d, *J* 8.0, Ar-*H*), 7.12-7.10 (2H, m, Ar-*H*), 6.83-6.81 (2H, m, Ar-*H*), 5.91 (2H, app s, (NCC*H*)$_2$), 3.82 (3H, s, OCH_3), 2.36 (3H, s, NCCH_3), 1.26 (9H, s, $CO_2C(CH_3)_3$); ^{13}C NMR (101 MHz, $CDCl_3$): δ 158.8, 149.4, 142.3, 133.1, 132.5, 132.4, 132.3, 132.2, 131.8, 129.8, 129.2, 121.3, 113.6, 113.4, 110.6, 83.2, 55.3, 27.6, 16.0; IR: ν_{max} (neat)/cm^{-1} 2979, 1739, 1610, 1513, 1250, 1177, 1093, 983; LRMS (ESI$^+$): *m/z* 442 ([^{79}Br] [M+H]$^+$, 95%), 444 ([^{81}Br] [M+H]$^+$, 100%), 464 ([^{79}Br] [M+Na]$^+$, 80%), 466 ([^{81}Br] [M+Na]$^+$, 70%); HRMS (ESI$^+$) found 464.08330 [[^{79}Br] [M+Na]$^+$], 466.8122 [[^{81}Br] [M+Na]$^+$], $C_{23}H_{24}{}^{79}BrNO_3Na^+$

requires 464.08318, $C_{23}H_{24}{}^{81}BrNO_3Na^+$ requires 466.08113; mp: 133-134 °C (DCM/petrol).

tert-Butyl 2-[4-(methoxycarbonyl)phenyl]-5-[4'-methyl-4-(trifluoromethyl)-(1,1'-biphenyl)-2-yl]-1*H*-pyrrole-1-carboxylate, 6c

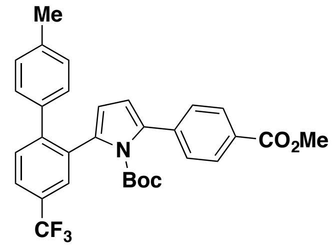

Prepared according to general procedure **H** using 2-(methylthio)-5-(trifluoromethyl)benzaldehyde **1n** (44 mg, 0.20 mmol, 1.0 equiv.), propargylic amine (methyl 4-{1-[(*tert*-butoxycarbonyl)amino]prop-2-yn-1-yl}benzoate) (75 mg, 0.26 mmol, 1.3 equiv.), $[Rh(nbd_2)]BF_4$ (7.5 mg, 10 mol%) , PNP(Cy) (4.2 mg, 5 mol%) and dcpm (4.1 mg, 5 mol%) in acetone (81 μL, 1 M). After 5 mins at 55 °C it was transferred to silver carbonate (55 mg, 0.20 mmol, 1.0 equiv.) and 4-tolylboronic acid (41 mg, 0.30 mmol, 1.5 equiv.) using acetone (0.45 mL). After stirring for 6 hrs at 55 °C the reaction was filtered and stirred with DCM (3 mL) and *p*-TSA (38 mg, 0.20 mmol, 1.0 equiv.) at room temperature for a further 3 hrs. The crude product was purified by flash column chromatography (5-10% EtOAc in petrol) to afford *pyrrole* **6c** as a pale brown solid (51.3 mg, 55%).

^{1}H NMR (400 MHz, $CDCl_3$): δ 8.03-8.00 (2H, m, Ar-*H*), 7.71-7.68 (2H, m, Ar-*H*), 7.56 (1H, d, *J* 8.5, Ar-*H*), 7.29 (2H, app td, *J* 4.0, 2.0, Ar-*H*), 7.15 (4H, br s, Ar-*H*), 6.24 (1H, d, *J* 3.5, NCC*H*), 6.16 (1H, d, *J* 3.5, NCC*H*), 3.95 (3H, s, CO_2CH_3), 2.40 (3H, s, ArCH_3), 1.04 (9H, s, $CO_2C(CH_3)_3$); ^{13}C NMR (101 MHz, $CDCl_3$): δ 166.9, 148.5, 144.9, 139.0, 137.2, 137.0, 135.5, 134.9, 133.8, 129.9, 129.0, 128.9 (q, $^2J_{CF}$ 32.5), 128.8, 128.7, 128.6, 128.5, 127.0 (q, $^3J_{CF}$ 3.5), 124.8 (q, $^3J_{CF}$ 3.5), 124.0 (q, $^1J_{CF}$ 270.0), 114.6, 114.0, 84.0, 52.1, 27.1, 21.1; ^{19}F NMR (377 MHz, $CDCl_3$): δ -62.3; IR: ν_{max} (neat)/cm^{-1} 2931, 1747, 1720, 1611, 1396, 1327, 1169, 1045; LRMS (ESI$^+$): *m/z* 536 ([M+H]$^+$, 75%), 558 ([M+Na]$^+$, 100%); HRMS (ESI$^+$) found 536.20435 [M+H]$^+$, $C_{31}H_{29}O_4F_3N^+$ requires 536.20432; mp: 128-129 °C (EtOAc/petrol).

10. One-pot synthesis of dihydropyrroles

General procedure I for the synthesis of dihydropyrroles as exemplified by the synthesis of 5-[2-(methylthio)phenyl]-2-phenyl-3,4-dihydro-2*H*-pyrrole, 7a

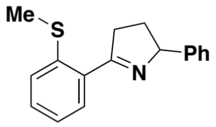

An oven-dried microwave vial was charged with [Rh(nbd)$_2$BF$_4$] (11.2 mg, 5 mol%) and PNP(Cy) (12.7 mg, 5 mol%). Once under an inert atmosphere, they were dissolved in acetone (1 mL). Hydrogen gas was bubbled through the solution at room temperature for 1-2 mins in order to generate the active catalyst species. The hydrogen gas was purged using nitrogen gas, and this was bubbled through the catalyst to dryness. The dry catalyst was dissolved in acetone (0.34 mL, 1.0 M with respect to aldehyde minus starting materials) and this was transferred to a nitrogen-filled microwave vial containing 2-(methylthio)benzaldehyde **1a** (77 μL, 0.60 mmol, 1.0 equiv.) and *tert*-butyl (1-phenylallyl)carbamate[7] **10a** (182 mg, 0.78 mmol, 1.3 equiv.). The reaction mixture, once homogenous (on occasion, sonication was required), was then stirred at 55 °C and monitored by TLC until complete. After 10 mins, the reaction vessel was opened to air followed by the addition of acetonitrile (3 mL) and *p*-TSA (114 mg, 0.6 mmol, 1.0 equiv.). The reaction mixture was further stirred until complete. After 16 hrs, the solution was diluted with acetonitrile (5 mL) and neutralised by the addition of sat. NaHCO$_3$ $_{(aq)}$ (10 mL) in a separatory funnel. The aqueous mixture was extracted with EtOAc (3 × 10 mL) and the combined organic extracts were washed with brine (20 mL) and dried over MgSO$_4$. The solvent was removed *in vacuo* to obtain the crude product and this was purified by flash column chromatography (10% Et$_2$O in petrol) to afford title *dihydropyrrole* **7a** as a pale yellow solid (134 mg, 84%).

^{1}H NMR (400 MHz, CDCl$_3$): δ 7.59 (1H, dd, *J* 7.5, 1.5, Ar-*H*), 7.52-7.49 (2H, m, Ar-*H*), 7.45-7.36 (4H, m, Ar-*H*), 7.32-7.28 (1H, m, Ar-*H*), 7.24 (1H, ddd, *J* 7.5, 7.0, 1.5, Ar-*H*), 5.47 (1H, tt, *J* 8.0, 2.0, NC*H*), 3.23-3.08 (2H, m, NCCH_2), 2.68-2.60 (1H, m, NCHCH_aH$_b$), 2.50 (3H, s, SCH_3), 1.90 (1H, ddd, *J* 9.5, 9.0, 8.0, NCHCH$_a$$H_b$); ^{13}C NMR (101 MHz, CDCl$_3$): δ 174.2, 144.7, 140.1, 132.9, 129.9, 129.8, 128.4, 126.6, 126.5, 125.2, 123.8, 76.7, 37.9, 32.6, 16.5; IR: ν_{max} (neat)/cm^{-1} 3059, 2918, 1612, 1557, 1470, 1329, 1084; LRMS (ESI$^+$): *m/z* 268 ([M+H]$^+$, 100%); HRMS (ESI$^+$) found 268.11560 [M+H]$^+$, C$_{17}$H$_{18}$NS$^+$ requires 268.11545; mp: 52-53 °C (DCM/hexane).

2-Methyl-5-[2-(methylthio)phenyl]-3,4-dihydro-2*H*-pyrrole, 7b

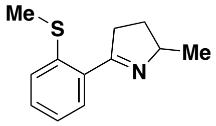

Prepared according to general procedure **I** using 2-(methylthio)benzaldehyde **1a** (19 μL, 0.15 mmol, 1.0 equiv.), allylic amine (*tert*-butyl but-3-en-2-ylcarbamate **10b**) (33.4 mg, 0.195 mmol, 1.3 equiv.), [Rh(nbd$_2$)]BF$_4$ (2.8 mg, 5 mol%) and PNP(Cy) (3.2 mg, 5 mol%) in acetone (98 μL, 1 M). After stirring for 3 hrs at 55 °C, acetonitrile (1.5 mL) and *p*-TSA (30.0 mg, 0.15 mmol, 1.0 equiv.) were added and stirred at room temperature for a further 3 hrs. The crude product was purified by flash column chromatography (10-50% Et$_2$O in petrol) to afford *dihydropyrrole* **7b** as a colourless oil (18.4 mg, 60%).

^{1}H NMR (400 MHz, CDCl$_3$): δ 7.49-7.46 (1H, m, Ar-*H*), 7.37-7.35 (1H, m, Ar-*H*), 7.31 (1H, dd, *J* 8.0, 1.5, Ar-*H*), 7.20-7.17 (1H, m, Ar-*H*), 4.47-4.38 (1H, m, NC*H*), 3.11 (1H, dddd, *J* 17.0, 9.5, 5.0, 2.0, NCHCH_aH$_b$), 3.00-2.91 (1H, m, NCCH_aH$_b$), 2.46 (3H, s, SCH_3), 2.25 (1H, dddd, *J* 12.5, 9.5, 7.5, 5.0, NCHCH$_a$$H_b$), 1.61-1.56 (1H, m, NCCH$_a$$H_b$), 1.43-1.42 (3H, d *J* 6.5, NCHCH_3); ^{13}C NMR (101 MHz, CDCl$_3$): δ 172.4, 139.3, 133.5, 130.2, 129.5, 125.3, 124.0, 69.0, 37.7, 31.0, 22.2, 16.4; IR: ν_{max} (neat)/cm^{-1} 2962, 2924, 1613, 1559, 1470, 1290, 908; LRMS (ESI$^+$): *m/z* 206 ([M+H]$^+$, 100%); HRMS (ESI$^+$) found 206.09988 [M+H]$^+$, $C_{12}H_{16}NS^+$ requires 206.09980.

5-[4-Bromo-2-(methylthio)phenyl]-2-(thiophen-3-yl)-3,4-dihydro-2*H*-pyrrole, 7c

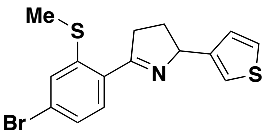

Prepared according to general procedure **I** using 4-bromo-2-(methylthio)benzaldehyde[3] **1e** (45.8 mg, 0.20 mmol, 1.0 equiv.), allylic amine (*tert*-butyl [1-(thiophen-2-yl)allyl]carbamate **10c**) (62.2 mg, 0.26 mmol, 1.3 equiv.), [Rh(nbd$_2$)]BF$_4$ (3.7 mg, 5 mol%) and PNP(Cy) (4.2 mg, 5 mol%) in acetone (89 μL, 1 M). After stirring for 16 hrs at 55 °C, acetonitrile (2 mL) and *p*-TSA (152 mg, 0.80 mmol, 4.0 equiv.) were added and stirred at room temperature for a further 6 hrs. The crude product was purified by flash column chromatography (10-50% Et$_2$O in petrol) to afford *dihydropyrrole* **7c** as an off white solid (58 mg, 82%).

^{1}H NMR (400 MHz, $CDCl_3$): δ 7.43 (1H, d, *J* 2.0, Ar-*H*), 7.40 (1H, d, *J* 8.0, Ar-*H*), 7.34-7.29 (3H, m, Ar-*H*), 7.15 (1H, dd, *J* 5.0, 1.5, Ar-*H*), 5.50 (1H, t, *J* 7.5, NC*H*), 3.11-3.06 (2H, m, NCCH_2), 2.61-2.53 (1H, m, NCHC$H_a$$H_b$), 2.48 (3H, s, S$CH_3$), 1.98-1.90 (1H, m, NCHC$H_a$$H_b$); ^{13}C NMR (101 MHz, $CDCl_3$): δ 172.7, 145.2, 142.9, 131.3, 131.0, 127.4, 126.7, 126.5, 125.8, 124.5, 120.2, 73.2, 37.4, 31.2, 16.6; IR: ν_{max} (neat)/cm^{-1} 2918, 1573, 1466, 1330, 1273, 1081, 988; LRMS (ESI$^+$): *m/z* 352 ([^{79}Br] [M+H]$^+$, 95%), 354 ([^{81}Br] [M+H]$^+$, 100%); HRMS (ESI$^+$) found 351.98270 [[^{79}Br] M+H]$^+$], 353.98037 [[^{81}Br] M+Na]$^+$], $C_{15}H_{15}{}^{79}BrNS_2{}^+$ requires 351.98238, $C_{15}H_{15}{}^{81}BrNS_2{}^+$ requires 352.98033; mp: 59-61 °C (Et_2O/petrol).

2-Cyclopropyl-5-[4-methoxy-2-(methylthio)phenyl]-3,4-dihydro-2*H*-pyrrole, 7d

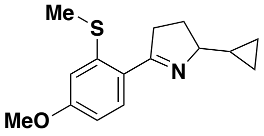

Prepared according to general procedure **I** using 4-methoxy-2-(methylthio)benzaldehyde **1k** (36.5 mg, 0.20 mmol, 1.0 equiv.), allylic amine (*tert*-butyl (1-cyclopropylallyl)carbamate **10f**) (51.3 mg, 0.26 mmol, 1.3 equiv.), [Rh(nbd$_2$)]BF_4 (3.7 mg, 5 mol%) and PNP(Cy) (4.2 mg, 5 mol%) in acetone (110 μL, 1 M). After stirring for 16 hrs at 55 °C, acetonitrile (2 mL) and *p*-TSA (38 mg, 0.20 mmol, 1.0 equiv.) were added and stirred at room temperature for a further 3 hrs. The crude product was purified by flash column chromatography (10-50% Et_2O in petrol) to afford *dihydropyrrole* **7d** as a colourless oil (25.1 mg, 48%).

^{1}H NMR (400 MHz, $CDCl_3$): δ 7.44 (1H, d, *J* 8.5, Ar-*H*), 6.82 (1H, d, *J* 2.5, Ar-*H*), 6.68 (1H, dd, *J* 8.5, 2.5, Ar-*H*), 4.02-3.97 (1H, m, NC*H*), 3.87 (3H, s, OCH_3), 3.01 (1H, dddd, *J* 12.0, 9.5, 4.5, 2.0, NCHC$H_a$$H_b$), 2.91 (1H, dddd, *J* 16.5, 9.5, 8.0, 1.5, NHCHC$H_a$$H_b$), 2.43 (3H, s, S$CH_3$), 2.21-2.18 (1H, m, NCHC*H*(CH_2)$_2$), 1.74 (1H, app d, *J* 8.5, NCC$H_a$$H_b$), 1.04-1.02 (1H, m, NCC$H_a$$H_b$), 0.55-0.45 (4H, m, NCHCH(CH_2)$_2$); ^{13}C NMR (101 MHz, $CDCl_3$): δ 172.2, 160.4, 142.0, 131.3, 125.9, 111.4, 108.1, 55.3, 37.2, 30.9, 28.8, 16.7, 16.4, 2.5; IR: ν_{max} (neat)/cm^{-1} 3078, 2959, 1593, 1556, 1435, 1332, 1247, 1229, 1045, 907; LRMS (ESI$^+$): *m/z* 262 ([M+H]$^+$, 100%); HRMS (ESI$^+$) found 262.12549 [M+H]$^+$, $C_{15}H_{20}NOS^+$ requires 262.12601.

5-[2,4-Bis(methylthio)phenyl]-2-phenyl-3,4-dihydro-2*H*-pyrrole, 7e

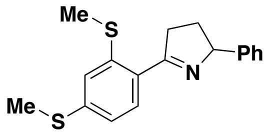

Prepared according to general procedure **I** using 2,4-bis(methylthio)benzaldehyde **1l** (29.7 mg, 0.15 mmol, 1.0 equiv.), allylic amine (*tert*-butyl (1-phenylallyl)carbamate[7] **10a**) (45.5 mg, 0.195 mmol, 1.3 equiv.), [Rh(nbd$_2$)]BF_4 (2.8 mg, 5 mol%) and PNP(Cy) (3.2 mg, 5 mol%) in acetone (70 μL, 1 M). After stirring for 1 hr at 55 °C, acetonitrile (1.5 mL) and *p*-TSA (57.2 mg, 0.30 mmol, 2.0 equiv.) were added and stirred at room temperature for a further 2 hrs. The crude product was purified by flash column chromatography (10-20% Et_2O in petrol) to afford *dihydropyrrole* **7e** as an off-white solid (40.9 mg, 87%).

^{1}H NMR (400 MHz, $CDCl_3$): δ 7.51 (1H, d, *J* 8.0, Ar-*H*), 7.47-7.45 (2H, m, Ar-*H*), 7.40-7.36 (2H, m, Ar-*H*), 7.28-7.25 (1H, m, Ar-*H*), 7.19 (1H, d, *J* 2.0, Ar-*H*), 7.06 (1H, dd, *J* 8.0, 2.0, Ar-*H*), 5.45 (1H, t, *J* 8.0, NC*H*), 3.15-3.07 (2H, m, NCCH_2), 2.65-2.58 (1H, m, NCHCH_aH$_b$), 2.56 (3H, s, SCH_3), 2.49 (3H, s, SCH_3), 1.90-1.81 (1H, m, NCHCH$_a$$H_b$); ^{13}C NMR (101 MHz, $CDCl_3$): δ 173.4, 144.8, 141.2, 141.1, 130.4, 129.1, 128.4, 126.58, 126.50, 122.1, 120.9, 76.6, 37.6, 32.4, 16.4, 15.4; IR: ν_{max} (neat)/cm^{-1} 2919, 1493, 1473, 1331, 1269, 1114, 1085, 968; LRMS (ESI$^+$): *m/z* 314 ([M+H]$^+$, 100%); HRMS (ESI$^+$) found 314.10234 [M+H]$^+$, $C_{18}H_{20}NS_2^+$ requires 314.10317; mp: 73-74 °C (DCM/hexane).

5-[3-(Methylthio)thiophen-2-yl]-2-phenyl-3,4-dihydro-2*H*-pyrrole, 7f

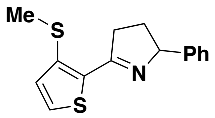

Prepared according to general procedure **I** using 3-(methylthio)thiophene-2-carbaldehyde **1m** (31.6 mg, 0.20 mmol, 1.0 equiv.), allylic amine (*tert*-butyl (1-phenylallyl)carbamate[7] **10a**) (60.6 mg, 0.26 mmol, 1.3 equiv.), [Rh(nbd$_2$)]BF_4 (3.7 mg, 5 mol%) and PNP(Cy) (4.2 mg, 5 mol%) in acetone (100 μL, 1 M). After stirring for 16 hrs at 55 °C, acetonitrile (3.0 mL) and *p*-TSA (57.2 mg, 0.30 mmol, 2.0 equiv.) were added and stirred at room temperature for a further 3 hrs. The crude product was purified by flash column chromatography (10-20% Et_2O in petrol) to afford *dihydropyrrole* **7f** as an off-white solid (41.6 mg, 76%).

^{1}H NMR (400 MHz, $CDCl_3$): δ 7.45 (1H, d, *J* 5.5, Ar-*H*), 7.37-7.33 (4H, m, Ar-*H*), 7.28-7.25 (1H, m, Ar-*H*), 7.07 (1H, d, *J* 5.5, Ar-*H*), 5.40-5.35 (1H, m, NC*H*), 3.23 (1H, dddd, *J* 14.5, 9.5, 4.5, 2.5, NCCH_aH$_b$), 3.14-3.05 (1H, m, NCCH$_a$$H_b$), 2.66-2.59

(1H, m, NCHCH_aH$_b$), 2.58 (3H, s, SCH_3), 1.91 (1H, dddd, *J* 12.5, 9.5, 8.0, 7.5, NCHCH$_a$$H_b$); ^{13}C NMR (101 MHz, CDCl$_3$): δ 168.1, 144.8, 137.6, 130.1, 128.4, 127.9, 127.3, 126.6, 126.5, 75.5, 37.8, 33.0, 17.4; IR: ν_{max} (neat)/cm^{-1} 2921, 1659, 1590, 1494, 1426, 1319, 1260, 1051; LRMS (ESI$^+$): *m/z* 274 ([M+H]$^+$, 100%); HRMS (ESI$^+$) found 274.07187 [M+H]$^+$, $C_{15}H_{16}NS_2^+$ requires 274.07187; mp: 57-58 °C (Et$_2$O/petrol).

2-(4-Methoxyphenyl)-5-[3-(methylthio)thiophen-2-yl]-3,4-dihydro-2*H*-pyrrole, 7g

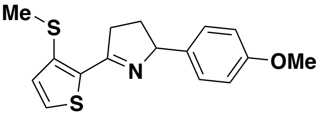

Prepared according to general procedure **I** using 3-(methylthio)thiophene-2-carbaldehyde **1m** (31.6 mg, 0.20 mmol, 1.0 equiv.), allylic amine (*tert*-butyl [1-(4-methoxyphenyl)allyl]carbamate **10d**) (79 mg, 0.26 mmol, 1.5 equiv.), [Rh(nbd$_2$)]BF$_4$ (3.7 mg, 5 mol%) and PNP(Cy) (4.2 mg, 5 mol%) in acetone (89 μL, 1 M). After stirring for 16 hrs at 55 °C, acetonitrile (3.0 mL) and *p*-TSA (57.2 mg, 0.30 mmol, 2.0 equiv.) were added and stirred at room temperature for a further 2 hrs. The crude product was purified by flash column chromatography (10-20% Et$_2$O in petrol) to afford *dihydopyrrole* **7g** as a very pale yellow solid (54.6 mg, 90%).

^{1}H NMR (400 MHz, CDCl$_3$): δ 7.45 (1H, d, *J* 5.5, Ar-*H*), 7.29-7.27 (2H, m, Ar-*H*), 7.07 (1H, d, *J* 5.5, Ar-*H*), 6.92-6.88 (2H, m, Ar-*H*), 5.34-5.30 (1H, m, NC*H*), 3.82 (3H, s, OCH_3), 3.22 (1H, dddd, *J* 16.5, 9.5, 4.5, 2.0, NCCH_aH$_b$), 3.12-3.03 (1H, m, NCCH$_a$$H_b$), 2.61-2.56 (1H, m, NCHCH_aH$_b$), 2.58 (3H, s, SCH_3) 1.88 (1H, dddd, *J* 12.5, 9.5, 8.0, 7.5, NCHCH$_a$$H_b$); ^{13}C NMR (101 MHz, CDCl$_3$): δ 167.8, 137.6, 137.0, 130.2, 129.1, 127.9, 127.5, 127.3, 113.8, 74.9, 55.3, 37.8, 33.1, 17.4; IR: ν_{max} (neat)/cm^{-1} 2953, 2833, 1612, 1511, 1464, 1351, 1319, 1244, 1035; LRMS (ESI$^+$): *m/z* 304 ([M+H]$^+$, 100%); HRMS (ESI$^+$) found 304.08220 [M+H]$^+$, $C_{16}H_{18}NOS_2^+$ requires 304.08243; mp: 79-80 °C (Et$_2$O/petrol).

5-[4-Bromo-2-(methylthio)phenyl]-2-[4-(trifluoromethyl)phenyl]-3,4-dihydro-2*H*-pyrrole, 7h

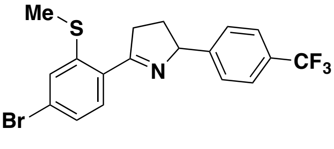

Prepared according to general procedure **I** using 4-bromo-2-(methylthio)benzaldehyde[3] **1e** (46 mg, 0.20 mmol, 1.0 equiv.), allylic amine (*tert*-butyl {1-[4-(trifluoromethyl)phenyl]allyl}carbamate) (90 mg, 0.3

mmol, 1.5 equiv.), [Rh(nbd$_2$)]BF_4 (3.7 mg, 5 mol%) and PNP(Cy) (4.2 mg, 5 mol%) in acetone (64 μL, 1 M). After stirring for 16 hrs at 55 °C, acetonitrile (3.0 mL) and *p*-TSA (57.2 mg, 0.30 mmol, 2.0 equiv.) were added and stirred at room temperature for a further 3 hrs. The crude product was purified by flash column chromatography (10-20% Et_2O in petrol) to afford *dihydropyrrole* **7h** as a very pale yellow solid (69.6 mg, 84%).

^{1}H NMR (400 MHz, $CDCl_3$): δ 7.62 (4H, app q, *J* 11.5, Ar-*H*), 7.45-7.42 (2H, m, Ar-*H*), 7.34 (1H, dd, *J* 8.5, 2.0, Ar-*H*), 5.47 (1H, t, *J* 8.0, NC*H*), 3.15-3.10 (2H, m, NCCH_2), 2.66 (1H, dtd, *J* 12.5, 8.0, 4.5, NCHCH_aH$_b$), 2.49 (3H, s, SCH_3), 1.83 (1H, app dq, *J* 12.5, 9.0, NCHCH$_a$$H_b$); ^{13}C NMR (101 MHz, $CDCl_3$): δ 173.7, 148.5, 143.1, 131.4, 130.6, 128.9 (q, $^2J_{CF}$ 32.5), 127.4, 126.8, 126.7, 126.1 (q, $^1J_{CF}$ 205.5), 125.4 (q, $^3J_{CF}$ 3.5), 124.7, 76.3, 37.7, 32.2, 16.5; ^{19}F NMR (377 MHz, $CDCl_3$): δ -62.3; IR: ν_{max} (neat)/cm^{-1} 2360, 1618, 1574, 1540, 1325, 1122, 1106; LRMS (ESI$^+$): *m/z* 414 ([^{79}Br] [M+H]$^+$, 95%), 416 ([^{81}Br] [M+H]$^+$, 100%); HRMS (ESI$^+$) found 435.9951 [[^{79}Br] M+Na]$^+$], 437.9929 [[^{81}Br] M+Na]$^+$], $C_{18}H_{15}{}^{79}BrNF_3SNa^+$ requires 435.9953, $C_{18}H_{15}{}^{81}BrNF_3SNa^+$ requires 437.9926; mp: 80-82 °C (Et_2O/petrol).

11. Reduction of dihydropyrrole products

General procedure J for the synthesis of *cis*-pyrrolidines as exemplified by the synthesis of 2-[2-(methylthio)phenyl]-5-phenylpyrrolidine, 8a

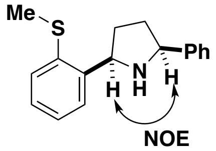

Dihydropyrrole (5-[2-(methylthio)phenyl]-2-phenyl-3,4-dihydro-2*H*-pyrrole **7a**) (17.0 mg, 0.06 mmol, 1.0 equiv.), was dissolved in toluene (0.2 mL) and cooled to -78 °C. Diisobutylaluminium hydride solution (0.18 mL, 1.0 M in toluene, 0.18 mmol, 3.0 equiv.) was added dropwise and the solution was stirred at -78 °C for 1 hr. The reaction was then quenched by the addition of sat. $NH_4Cl_{(aq)}$ (0.16 mL) and warmed to room temperature. The reaction mixture was diluted with DCM, dried over $MgSO_4$, filtered and concentrated i*n vacuo*. The reaction gave the *cis*-diastereoisomer as the only isomer detectable by crude ^{1}H NMR spectroscopy. The crude product was purified by flash column chromatography (5% EtOAc in petrol) to yield title *pyrrolidine* **8a** as a colourless oil (15.6 mg, 96%).

^{1}H NMR (400 MHz, $CDCl_3$): δ 8.00-7.98 (1H, m, Ar-*H*), 7.61 (2H, d, *J* 7.5, Ar-*H*), 7.41 (2H, app t, *J* 7.5, Ar-*H*), 7.33-7.25 (4H, m, Ar-*H*), 4.78 (1H, t, *J* 7.5, NHC*H*(Ar-SCH$_3$)), 4.40 (1H, t, *J* 7.5, NHC*H*(Ph)), 2.54 (3H, s, SCH_3), 2.49-2.44 (1H, m,

NHCH(Ar-SCH_3)C$H_a$$H_b$), 2.36-2.25 (2H, m, N*H* and NHCH(Ph)C$H_a$$H_b$), 1.89-1.81 (1H, m, NHCH(Ph)$CH_a$$H_b$), 1.75 (1H, tdd, *J* 11.5, 5.5, 2.5, NHCH(Ar-SCH_3)$CH_a$$H_b$); ^{13}C NMR (101 MHz, $CDCl_3$): δ 145.2, 143.4, 136.2, 128.3, 127.2, 126.9, 126.8, 126.3, 125.4, 125.2, 62.2, 58.4, 34.5, 32.8, 16.1; IR: ν_{max} (neat)/cm^{-1} 3329, 3059, 3026, 2867, 1603, 1491, 1460, 1394, 1124, 907; LRMS (ESI$^+$): *m/z* 270 ([M+H]$^+$, 100%); HRMS (ESI$^+$) found 270.13096 [M+H]$^+$, $C_{17}H_{20}NS^+$ requires 270.13110.

Cis-selectivity from a DIBAL-H reduction was confirmed by NOE experiments, which displayed in-space interactions between the two hydrogen atoms shown.

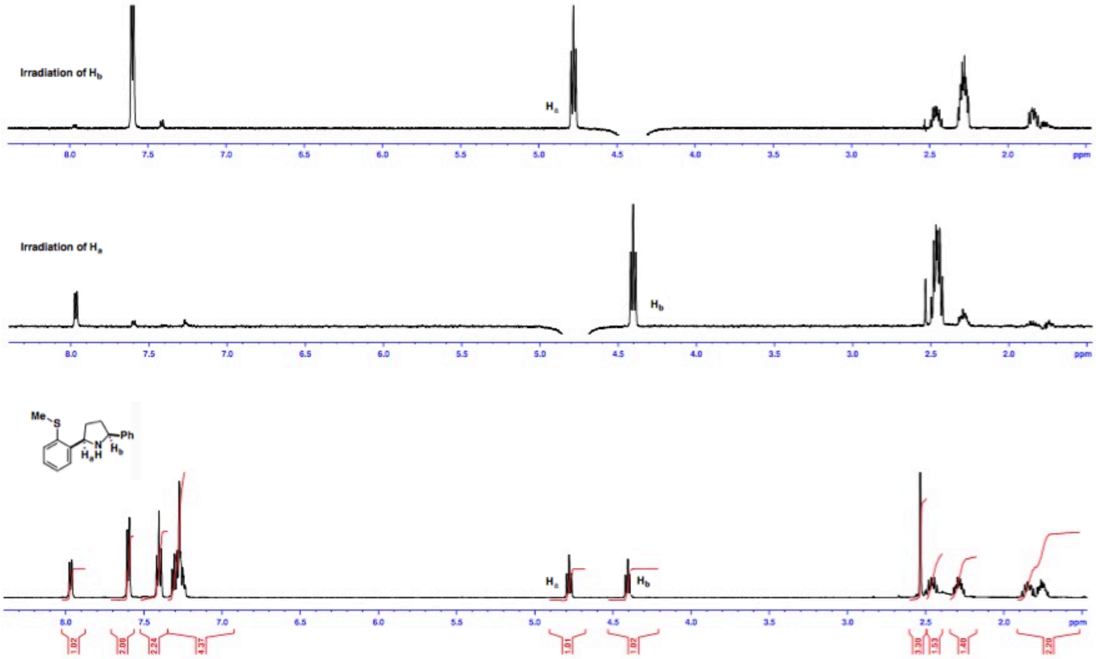

2-[3-(Methylthio)thiophen-2-yl]-5-phenylpyrrolidine, 8b

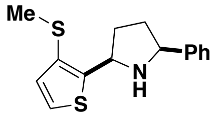

Prepared according to general procedure **J** using dihydropyrrole (5-[3-(methylthio)thiophen-2-yl]-2-phenyl-3,4-dihydro-2*H*-pyrrole **7f**), (15 mg, 0.06 mmol, 1.0 equiv.), diisobutylaluminium hydride solution (0.18 mL, 1.0 M in toluene, 0.18 mmol, 3.0 equiv.) in toluene (0.2 mL). After stirring for 2 hrs at -78 °C sat. NH_4Cl $_{(aq)}$ (0.16 mL) was added and the reaction gave the *cis*-diastereoisomer as the only isomer detectable by crude ^{1}H NMR spectroscopy. The crude product was purified by flash column chromatography (15% Et_2O in petrol) to yield *pyrrolidine* **8b** as a colourless oil (14.9 mg, 90%).

^{1}H NMR (400 MHz, $CDCl_3$): δ 7.58-7.56 (2H m, Ar-*H*), 7.38 (2H, dd, *J* 8.0, 7.0, Ar-*H*), 7.29 (1H, app dt, *J* 7.5, 1.5, Ar-*H*), 7.23 (1H, d, *J* 5.0, Ar-*H*), 7.04 (1H, d, *J* 5.0, Ar-

H), 4.91-4.87 (1H, m, NHC*H*CS), 4.37 (1H, t, *J* 7.5, NHC*H*(Ph)), 2.45 (3H, s, SC*H*3), 2.40-2.34 (1H, m, NHCH(CS)C*Ha*Hb), 2.29-2.22 (1H, m, NHCH(Ph)C*Ha*Hb), 1.86 (2H, tdt, *J* 7.5, 3.5, 2.80, NHCH(CS)CHa*Hb* and NHCH(Ph)CHa*Hb*), 1.29 (1H, br s, N*H*); ^{13}C NMR (101 MHz, $CDCl_3$): δ 150.0, 145.1, 129.7, 129.5, 128.3, 126.9, 126.7, 123.2, 62.3, 56.3, 34.7, 34.4, 19.1; IR: ν_{max} (neat)/cm^{-1} 2920, 1491, 1452, 1319, 1246, 1099, 908; LRMS (ESI$^+$): *m/z* 276 ([M+H]$^+$, 100%); HRMS (ESI$^+$) found 276.08766 [M+H]$^+$, $C_{15}H_{18}NS_2^+$ requires 276.08752.

2-(4-Methoxyphenyl)-5-[3-(methylthio)thiophen-2-yl]pyrrolidine, 8c

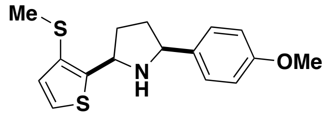

Prepared according to general procedure **J** using dihydropyrrole (2-(4-methoxyphenyl)-5-[3-(methylthio)thiophen-2-yl]-3,4-dihydro-2*H*-pyrrole **7g**), (40 mg, 0.13 mmol, 1.0 equiv.), diisobutylaluminium hydride solution (0.39 mL, 1.0 M in toluene, 0.39 mmol, 3.0 equiv.) in toluene (0.8 mL). After stirring for 1 hr at -78 °C sat. $NH_4Cl_{(aq)}$ (0.35 mL) was added and the reaction gave the *cis*-diastereoisomer as the only isomer detectable by crude ^{1}H NMR spectroscopy. The crude product was purified by flash column chromatography (15% Et_2O in petrol) to yield *pyrrolidine* **8c** as a colourless oil (35.6 mg, 90%).

^{1}H NMR (400 MHz, $CDCl_3$): δ 7.50-7.47 (2H, m, Ar-*H*), 7.22 (1H, d, *J* 5.0, Ar-*H*), 7.03 (1H, d, *J* 5.0, Ar-*H*), 6.94-6.90 (2H, m, Ar-*H*), 4.86 (1H, dd, *J* 8.0, 6.0, NHC*H*(CS)), 4.31 (1H, t, *J* 7.5, NHC*H*(Ar-OCH3)), 3.84 (3H, s, OC*H*3), 2.52 (1H, br s, N*H*), 2.44 (3H, s, SC*H*3), 2.39-2.34 (1H, m, NHCH(CS)C*Ha*Hb), 2.23-2.16 (1H, m, NHCH(Ar-OCH3)C*Ha*Hb), 1.88-1.80 (2H, m, NHCH(CS)CHa*Hb* and NHCH(Ar-OCH3)CHa*Hb*); ^{13}C NMR (101 MHz, $CDCl_3$): δ 158.6, 150.1, 137.1, 129.7, 127.7, 123.2, 123.0, 113.6, 61.8, 56.1, 55.3, 34.7, 34.4, 19.1; IR: ν_{max} (neat)/cm^{-1} 2965, 2833, 1611, 1584, 1511, 1440, 1244, 1035; LRMS (ESI$^+$): *m/z* 306 ([M+H]$^+$, 100%); HRMS (ESI$^+$) found 306.09800 [M+H]$^+$, $C_{16}H_{20}NOS_2^+$ requires 306.09808.

2-[4-Bromo-2-(methylthio)phenyl]-5-(thiophen-3-yl)pyrrolidine, 8d

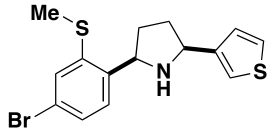

Prepared according to general procedure **J** using dihydropyrrole (5-[4-bromo-2-(methylthio)phenyl]-2-(thiophen-3-yl)-3,4-dihydro-2*H*-pyrrole **7c**) (40 mg, 0.11 mmol, 1.0 equiv.), diisobutylaluminium hydride solution (0.33 mL, 1.0 M in toluene, 0.33 mmol, 3.0 equiv.) in toluene (0.5 mL). After stirring for 2 hrs at -78 °C sat. $NH_4Cl_{(aq)}$ (0.3 mL) was added and the reaction gave the *cis*-

diastereoisomer as the only isomer detectable by crude ^{1}H NMR spectroscopy. The crude product was purified by flash column chromatography (15% Et_2O in petrol) to yield *pyrrolidine* **8d** as a colourless oil (31.1 mg, 77%).

^{1}H NMR (400 MHz, $CDCl_3$): δ 7.76 (1H, d, *J* 8.0, Ar-*H*), 7.34 (1H, dd, *J* 5.0, 3.0, Ar-*H*), 7.32-7.28 (3H, m, Ar-*H*), 7.21 (1H, dd, *J* 5.0, 1.5, Ar-*H*), 4.63 (1H, app t, *J* 7.5, NHC*H*(CCHS)), 4.46 (1H, t, *J* 8.0, NHC*H*(Ar-Br)), 2.51 (3H, s, SCH_3), 2.41 (1H, dtd, *J* 12.5, 8.0, 7.5, NHCH(CCHS)CH_aH$_b$), 2.25-2.19 (1H, m, NHCH(Ar-Br)CH_aH$_b$), 1.86-1.77 (1H, m, NHCH(Ar-Br)CH$_a$$H_b$), 1.65 (1H, dddd, *J* 12.0, 9.5, 7.5, 5.0, NHCH(CCHS)CH$_a$$H_b$), 1.49 (1H, br s, N*H*); ^{13}C NMR (101 MHz, $CDCl_3$): δ 146.6, 142.2, 127.8, 127.7, 127.1, 126.6, 125.7, 120.9, 120.1, 58.1, 57.7, 33.3, 32.6, 28.4, 15.8; IR: ν_{max} (neat)/cm^{-1} 2969, 2920, 1575, 1552, 1492, 1327, 1084, 907; LRMS (ESI$^+$): *m/z* 354 ([^{79}Br] [M+H]$^+$, 95%), 356 ([^{81}Br] [M+H]$^+$, 100%); HRMS (ESI$^+$) found 353.99802 [[^{79}Br] M+H]$^+$], 355.99585 [[^{81}Br] M+H]$^+$], $C_{15}H_{17}{}^{79}BrS_2^+$ requires 353.99803, $C_{15}H_{17}{}^{81}BrNS_2^+$ requires 355.99598.

2-[4-Bromo-2-(methylthio)phenyl]-5-[4-(trifluoromethyl)-phenyl]-pyrrolidine, 8e

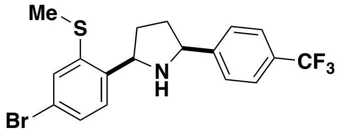

Prepared according to general procedure **J** using dihydropyrrole (5-[4-bromo-2-(methylthio)phenyl]-2-[4-(trifluoromethyl)phenyl]-3,4-dihydro-2*H*-pyrrole **7h**) (46 mg, 0.11 mmol, 1.0 equiv.), diisobutylaluminium hydride solution (0.33 mL, 1.0 M in toluene, 0.33 mmol, 3.0 equiv.) in toluene (0.6 mL). After stirring for 1 hr at -78 °C sat. NH_4Cl $_{(aq)}$ (0.3 mL) was added and the reaction gave the *cis*-diastereoisomer as the only isomer detectable by crude ^{1}H NMR spectroscopy. The crude product was purified by flash column chromatography (15% Et_2O in petrol) to yield *pyrrolidine* **8e** as a colourless oil (42.1 mg, 92%).

^{1}H NMR (400 MHz, $CDCl_3$): δ 7.82 (1H, d, *J* 8.0, Ar-*H*), 7.66 (4H, app q, *J* 9.0, Ar-*H*), 7.35-7.29 (2H, m, Ar-*H*), 4.68 (1H, t, *J* 7.5, NHC*H*(Ar-Br)), 4.42 (1H, t, *J* 8.0, NHC*H*(Ar-CF$_3$)), 2.52 (3H, s, SCH_3), 2.49-2.40 (1H, m, NHCH(Ar-Br)CH_aH$_b$), 2.33-2.26 (1H, m, NHCH(Ar-CF$_3$)CH_aH$_b$), 2.13 (1H, br s, N*H*), 1.80-1.70 (1H, m, NHCH(Ar-CF$_3$)CH$_a$$H_b$), 1.70-1.61 (1H, m, NHCH(Ar-Br)CH$_a$$H_b$); ^{13}C NMR (101 MHz, $CDCl_3$): δ 149.4, 141.9, 138.6, 129.1 (q, $^2J_{CF}$ 32.0), 127.8, 127.7, 127.1, 127.0, 125.2 (q, $^3J_{CF}$ 3.5), 124.2 (q, $^1J_{CF}$ 247.5), 121.0, 61.5, 57.9, 34.4, 32.5, 15.8; ^{19}F NMR (377 MHz, $CDCl_3$): δ -62.3; IR: ν_{max} (neat)/cm^{-1} 2943, 1619, 1575, 1460, 1324, 1163, 1066; LRMS (ESI$^+$): *m/z* 416 ([^{79}Br] [M+H]$^+$, 95%), 418 ([^{81}Br] [M+H]$^+$, 100%); HRMS (ESI$^+$) found 416.0304 [[^{79}Br] M+H]$^+$], 418.0282 [[^{81}Br] M+H]$^+$], $C_{18}H_{18}{}^{79}BrF_3S^+$ requires 416.0289, $C_{18}H_{18}{}^{81}BrNF_3S^+$ requires 418.0312.

2-Cyclopropyl-5-[4-methoxy-2-(methylthio)phenyl]pyrrolidine, 8f

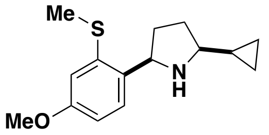

Prepared according to general procedure **J** using dihydropyrrole (2-cyclopropyl-5-[4-methoxy-2-(methylthio)phenyl]-3,4-dihydro-2*H*-pyrrole **7d**) (20 mg, 0.08 mmol, 1.0 equiv.), diisobutylaluminium hydride solution (0.24 mL, 1.0 M in toluene, 0.24 mmol, 3.0 equiv.) in toluene (0.2 mL). After stirring for 2 hrs at -78 °C sat. NH_4Cl $_{(aq)}$ (0.22 mL) was added and the reaction gave the *cis*-diastereoisomer as the only isomer detectable by crude ^1H NMR spectroscopy. The crude product was purified by flash column chromatography (15% Et_2O in petrol) to yield *pyrrolidine* **8f** as a colourless oil (18.1 mg, 88%).

^1H NMR (400 MHz, $CDCl_3$): δ 7.62 (1H, d, *J* 8.5, Ar-*H*), 6.77 (1H, d, *J* 2.5, Ar-*H*), 6.72 (1H, dd, *J* 8.5, 2.5, Ar-*H*), 4.46 (1H, t, *J* 7.5, NHC*H*(Ar)), 3.83 (3H, s, OCH_3), 2.61 (1H, app q, *J* 7.5, NHC*H*(CH(CH_2)$_2$)), 2.48 (3H, s, SCH_3), 2.24-2.17 (1H, m, NHCH(Ar)C$H_a$$H_b$), 2.02-1.94 (2H, m, N*H* and NHCH(CH(CH_2)$_2$)C$H_a$$H_b$), 1.75-1.66 (1H, m, NHCH(CH(CH_2)$_2$)C$H_a$$H_b$), 1.66-1.57 (1H, m, NHCH(Ar)C$H_a$$H_b$), 0.96-0.91 (1H, m, NHCH(C*H*(CH_2)$_2$)), 0.53-0.46 (2H, m, NHCH(CH(CH_2)(C$H_a$$H_b$)), 0.31-0.27 (1H, m, NHCH(CH(CH_2)(C$H_a$$H_b$)), 0.24-0.19 (1H, m, NHCH(CH(CH_2)(C$H_a$$H_b$)); ^{13}C NMR (101 MHz, $CDCl_3$): δ 158.6, 137.6, 135.1, 127.1, 111.7, 109.7, 63.7, 57.9, 55.3, 32.5, 30.7, 16.2, 16.0, 3.1, 2.0; IR: ν_{max} (neat)/cm^{-1} 2999, 2832, 1597, 1571, 1484, 1436, 1290, 1242, 1053; LRMS (ESI^+): *m/z* 264 ($[M+H]^+$, 100%); HRMS (ESI^+) found 264.14158 $[M+H]^+$, $C_{15}H_{22}NOS^+$ requires 264.14166.

2-(2-(Methylthio)phenyl)-5-phenylpyrrolidine, 8g

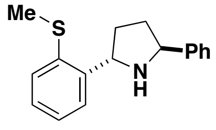

Dihydropyrrole (5-[2-(methylthio)phenyl]-2-phenyl-3,4-dihydro-2*H*-pyrrole **7a**) (120 mg, 0.45 mmol, 1.0 equiv.), was dissolved in acetic acid (2.25 mL). Sodium borohydride (42 mg, 1.13 mmol, 2.5 equiv.) was added and the solution was stirred at room temperature for 1 hr. The reaction was then quenched by the addition of water (10 mL) and potassium carbonate (0.1 g). The aqueous layer was extracted with EtOAc (3 × 10 mL) and the combined organic layers with washed with brine (20 mL), dried over $MgSO_4$ and concentrated under reduced pressure. The reaction gave the *trans*- to *cis*-diastereoisomers in a ratio of 3:1 (*trans*:*cis*) and they were separated by column chromatography (5-20% Et_2O in

petrol) to yield *trans-pyrrolidine* **8g** as the major diastereoisomer as a colourless oil (83 mg, 68%).

^{1}H NMR (400 MHz, $CDCl_3$): δ 7.64 (1H, dd, *J* 8.5, 1.0, Ar-*H*), 7.48-7.45 (2H, m, Ar-*H*), 7.39 (2H, dd, *J* 8.5, 7.0, Ar-*H*), 7.31-7.28 (1H, m, Ar-*H*), 7.27-7.21 (3H, m, Ar-*H*), 5.00 (1H, t, *J* 7.0, NHC*H*(Ar-SCH_3)CH_2), 4.57 (1H, t, *J* 7.0, NHC*H*(Ph)CH_2), 2.56-2.50 (1H, m, NHCH(Ar-SCH_3)CH_aH$_b$), 2.53 (3H, s, SCH_3), 2.46-2.38 (1H, m, NHCH(Ph)CH_aH$_b$), 2.09 (1H, br s, N*H*), 1.98-1.91 (1H, m, NHCH(Ph)CH$_a$$H_b$), 1.83 (1H, app ddt, *J* 12.0, 9.0, 7.0, NHCH(Ar-SCH_3)CH$_a$$H_b$); ^{13}C NMR (101 MHz, $CDCl_3$): δ 145.9, 143.7, 136.4, 128.5, 127.2, 126.8, 126.4, 125.6, 125.36, 125.17, 62.3, 58.8, 35.4, 33.6, 16.1; IR: ν_{max} (neat)/cm^{-1} 2920, 1612, 1586, 1492, 1436, 1272, 1060; LRMS (ESI$^+$): *m/z* 270 ([M+H]$^+$, 100%); HRMS (ESI$^+$) found 270.13023 [M+H]$^+$, $C_{15}H_{22}NOS^+$ requires 270.13110.

trans-selectivity from a sodium borohydride reduction was confirmed by NOE experiments, which displayed a lack of in-space interactions between the two hydrogen atoms shown in comaparison to the *cis*-pyrrolidine.

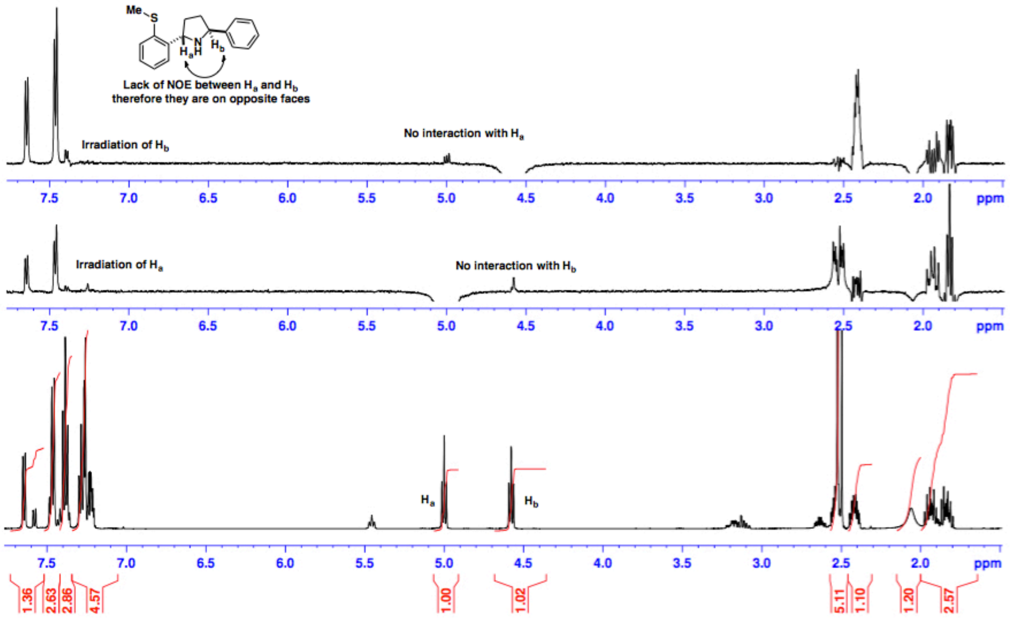

2-Allyl-2-[2-(methylthio)phenyl]-5-phenylpyrrolidine, 8h

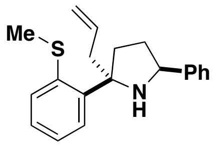

Dihydropyrrole (5-[2-(methylthio)phenyl]-2-phenyl-3,4-dihydro-2*H*-pyrrole **7a**) (50 mg, 0.19 mmol, 1.0 equiv.) was dissolved in THF (1.9 mL) and cooled to 0 °C.

Allylmagnesium bromide (0.36 mL, 0.8 M in THF, 0.29 mmol, 1.5 equiv.) was added dropwise and the solution was warmed to room temperature over 30 mins. The reaction was then quenched by the addition of sat. $NH_4Cl_{(aq)}$ (3 mL) and the aqueous layer was extracted with EtOAc (3 × 5 mL) and the combined organic layers were washed with brine (10 mL), dried over $MgSO_4$ and concentrated under reduced pressure. The reaction gave the *cis*- to *trans*-diastereoisomers in a ratio of 25:1 (*cis*:*trans*) and they were separated by column chromatography (20% Et_2O in petrol) to yield title *cis-pyrrolidine* **8h** as a colourless oil (53.6 mg, 91%).

^{1}H NMR (400 MHz, $CDCl_3$): δ 7.97-7.95 (1H, m, Ar-*H*), 7.53 (2H, dd, *J* 8.0, 1.0, Ar-*H*), 7.39-7.34 (3H, m, Ar-*H*), 7.29-7.20 (3H, m, Ar-*H*), 5.37 (1H, dddd, *J* 17.0, 10.0, 9.0, 5.5, $NHCCH_2CHCH_2$), 5.09-4.99 (2H, m, $NHCCH_2CHCH_2$), 4.44 (1H, dd, *J* 9.0, 6.5, NHC*H*), 3.37 (1H, dd, *J* 14.0, 5.5, $NHCCH_aH_bCHCH_2$), 2.65 (1H, dd, *J* 14.0, 9.0, $NHCCH_aH_bCHCH_2$), 2.54 (3H, s, SCH_3), 2.48-2.38 (3H, m, N*H* and NCH(Ph)CH_2), 2.25 (1H, dddd, *J* 12.0, 7.5, 6.5, 4.5, $NCCH_aH_bCH_2CH(Ph)$), 1.69 (1H, app dq, *J* 12.0, 9.0, $NCCH_aH_bCH_2CH(Ph)$); ^{13}C NMR (101 MHz, $CDCl_3$): δ 146.9, 145.7, 135.4, 135.2, 128.5, 128.4, 128.2, 126.9, 126.8, 126.7, 124.9, 118.0, 66.6, 60.4, 43.8, 38.1, 35.2, 18.0; IR: ν_{max} (neat)/cm^{-1} 2975, 2922, 1637, 1584, 1435, 1256, 1049; LRMS (ESI$^+$): *m/z* 310 ([M+H]$^+$, 100%); HRMS (ESI$^+$) found 310.16204 [M+H]$^+$, $C_{20}H_{24}NS^+$ requires 310.16240.

Cis-selectivity from allylmagnesium bromide addition was confirmed by NOE experiments, which displayed in-space interactions between the two hydrogen atoms shown.

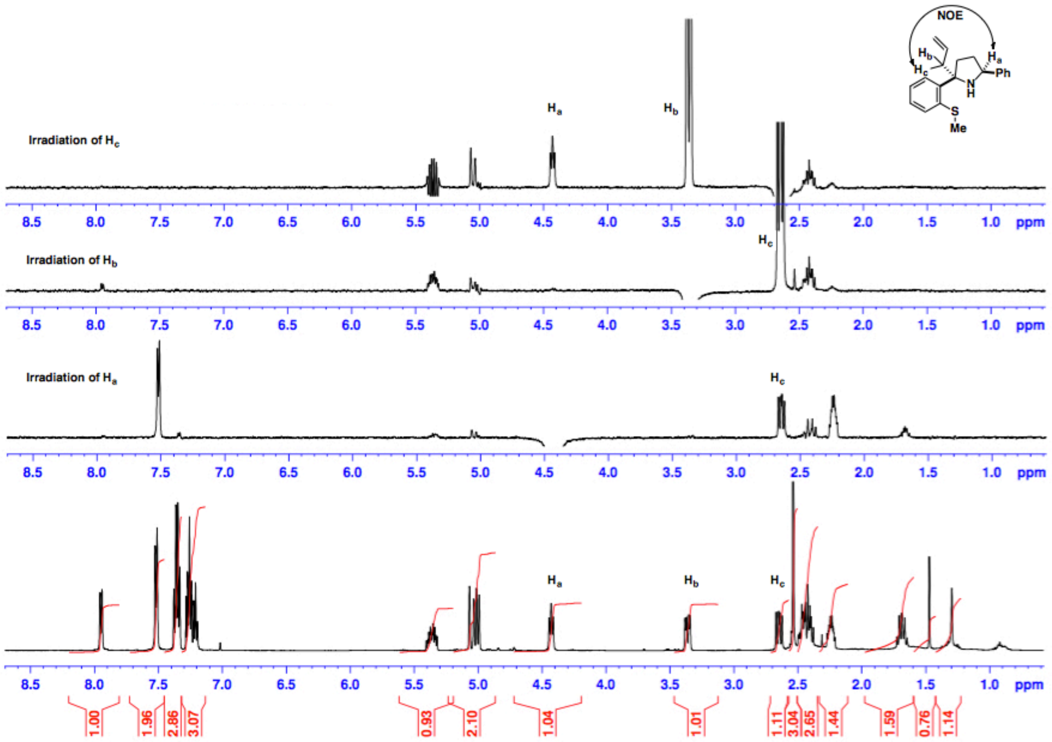

11. References

1. I. Pernik, J. F. Hooper, A. B. Chaplin, A. S. Weller, M. C. Willis, *ACS Catal.*, **2012**, *2*, 2779-2786.
2. C. González-Rodríguez, R. J. Pawley, A. B. Chaplin, A. L. Thompson, A. S. Weller, M. C. Willis, *Angew. Chem. Int. Ed.*, **2011**, *50*, 5134-5138.
3. M. Arambasic, J. F. Hooper, M. C. Willis, *Org. Lett.*, **2013**, *15*, 5162-5165.
4. S-J. Poingdestre, J. D. Goodacre, A. S. Weller, M. C. Willis, *Chem. Commun.*, **2012**, *48*, 6354-6356.
5. M. Castaing, S. L, Wason, B. Estepa, J. F. Hooper, M. C. Willis, *Angew. Chem. Int. Ed.*, **2013**, *52*, 13280-13283.
6. E. Hernando, R. Gomez Arryas, J. C. Carretero, *Chem. Commun.*, **2012**, *48*, 9622-9624.
7. T. Mecozzi, M. Petrini, *J. Org. Chem.*, **1999**, *64*, 8970-8972.
8. L. Huang, W. D. Wulff. *J. Am. Chem. Soc.*, **2011**, *133*, 8892-8895.
9. C. G. Goodman, D. T. Do, J. S. Johnson, *Org. Lett.*, **2013**, *15*, 2446-2449.
10. S. A. Tsai, M. E. Tauchert, R. G. Bergman, J. A. Ellman, *J. Am. Chem. Soc.*, **2011**, *133*, 1248-1250.
11. G. C. Tsui, F. Menard, M. Lautens, *Org. Lett.*, **2010**, *12*, 2456-2459.
12. J. F. Teichert, S. Zhang, A. W. Van Zijl, J. W. Slaa, A. J. Minnaard, B. L. Feringa, *Org. Lett.*, 2010, 12, 4658-4660.
13. E. S. Lee, H. S. Yeom, J. H. Hwang, S. Shin, *Eur. J. Org. Chem.*, **2007**, 3503-3507.
14. D. J. Wiex, D. Marković, M. Ueda, J. F. Hartwig, *Org. Lett.*, **2009**, *11*, 2944-2947.
15. K. K. Chauhan, C. G. Frost, *J. Chem. Soc. Perkin Trans. 1*, **2000**, 3015.

Aldehyde 1i: ^{1}H NMR (400 MHz, $CDCl_3$)

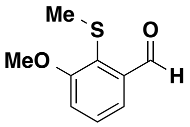

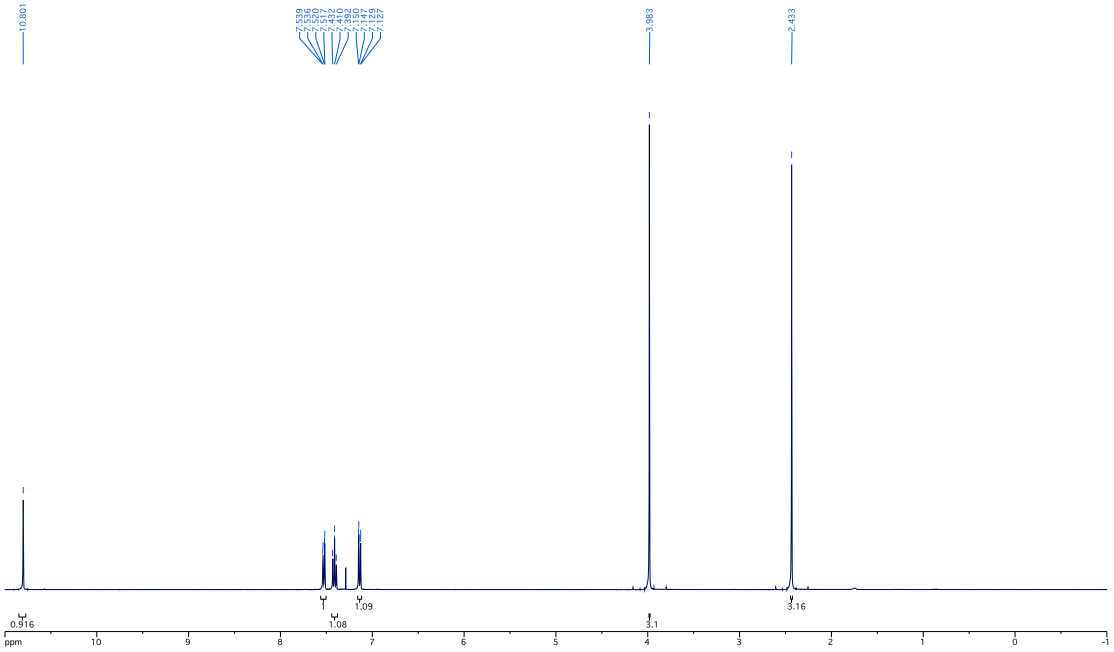

Aldehyde 1i: ^{13}C NMR (101 MHz, $CDCl_3$)

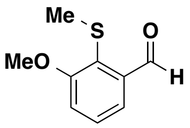

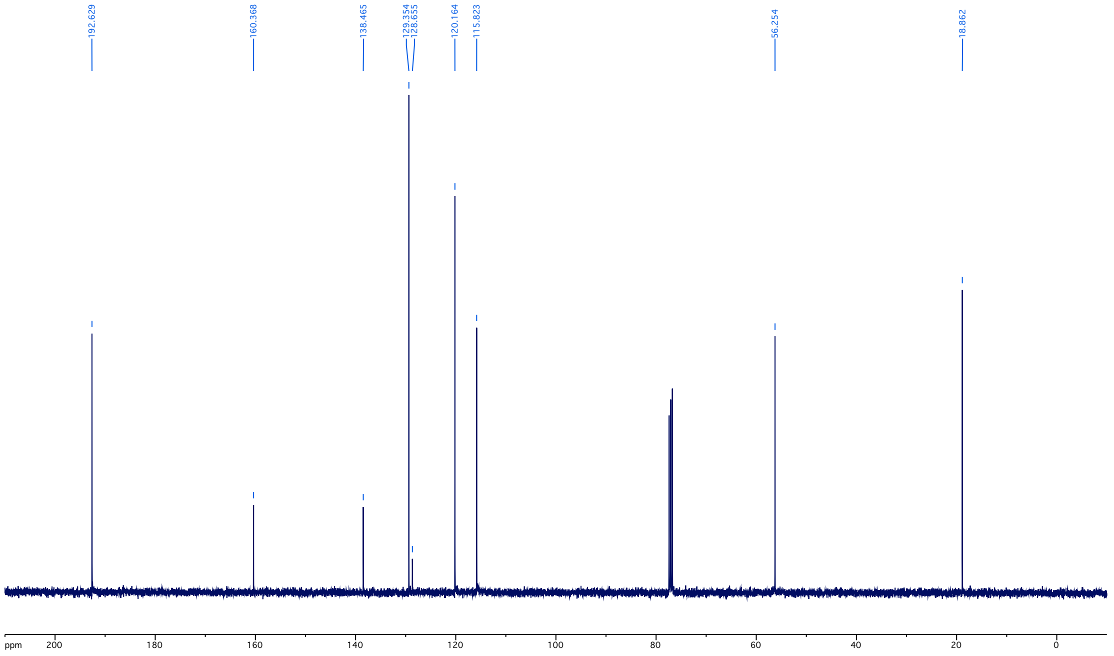

Aldehyde 1j: ^{1}H NMR (400 MHz, $CDCl_3$)

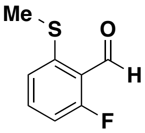

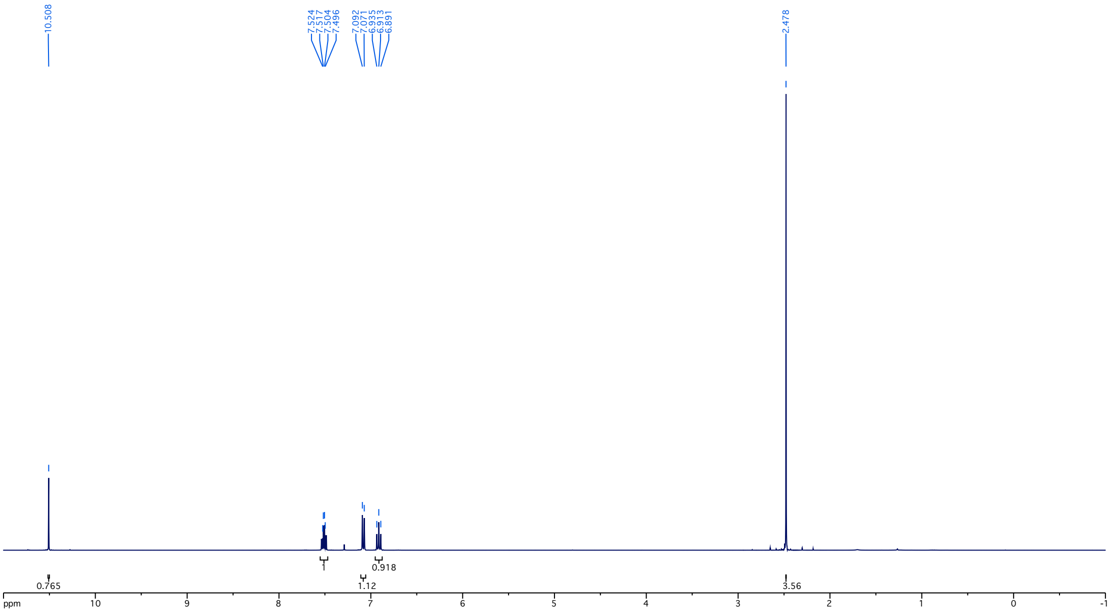

Aldehyde 1j: ^{13}C NMR (101 MHz, $CDCl_3$)

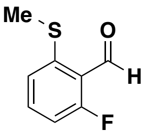

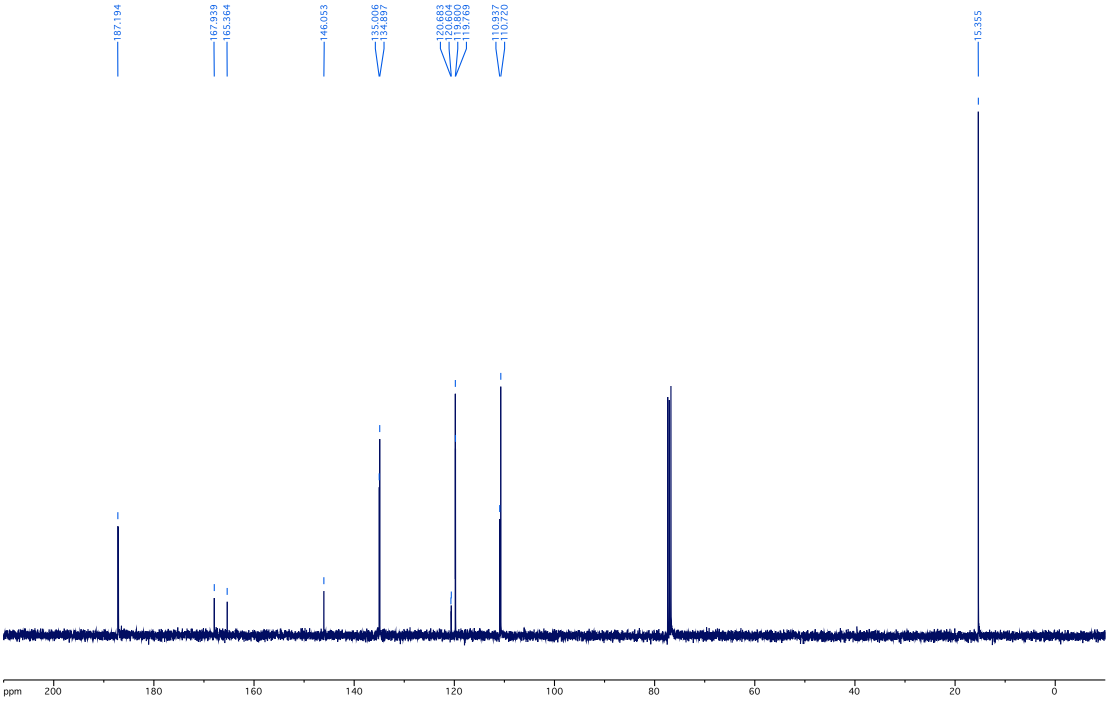

Aldehyde 1j: ^{19}F NMR (377 MHz, $CDCl_3$)

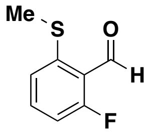

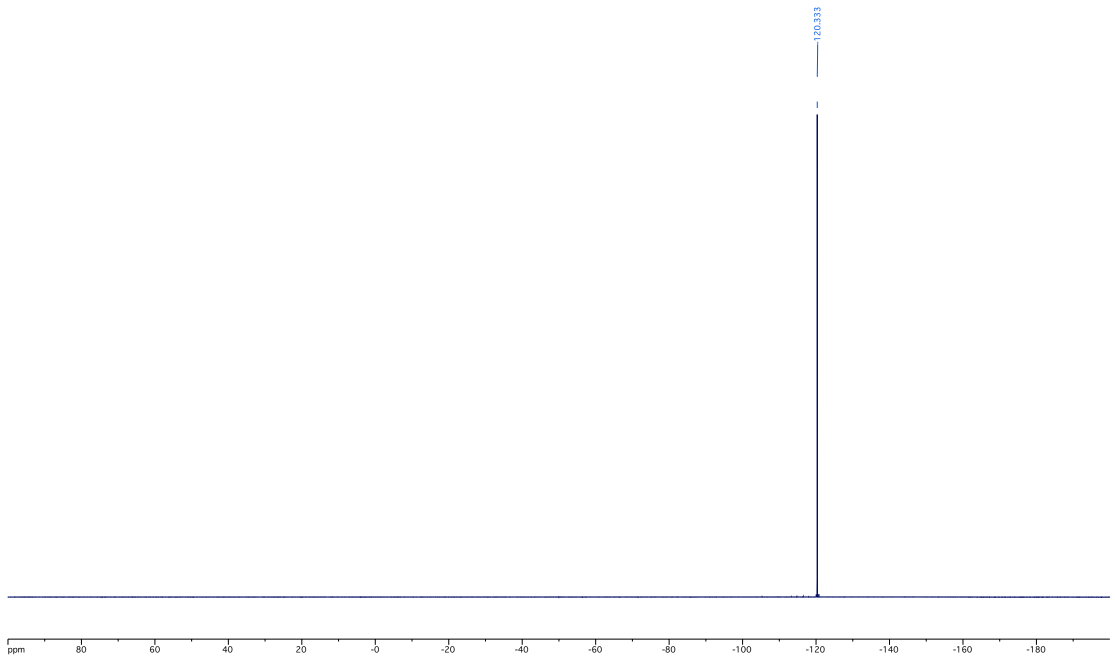

Aldehyde 1k: ^{1}H NMR (400 MHz, $CDCl_3$)

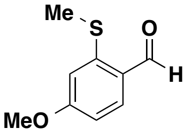

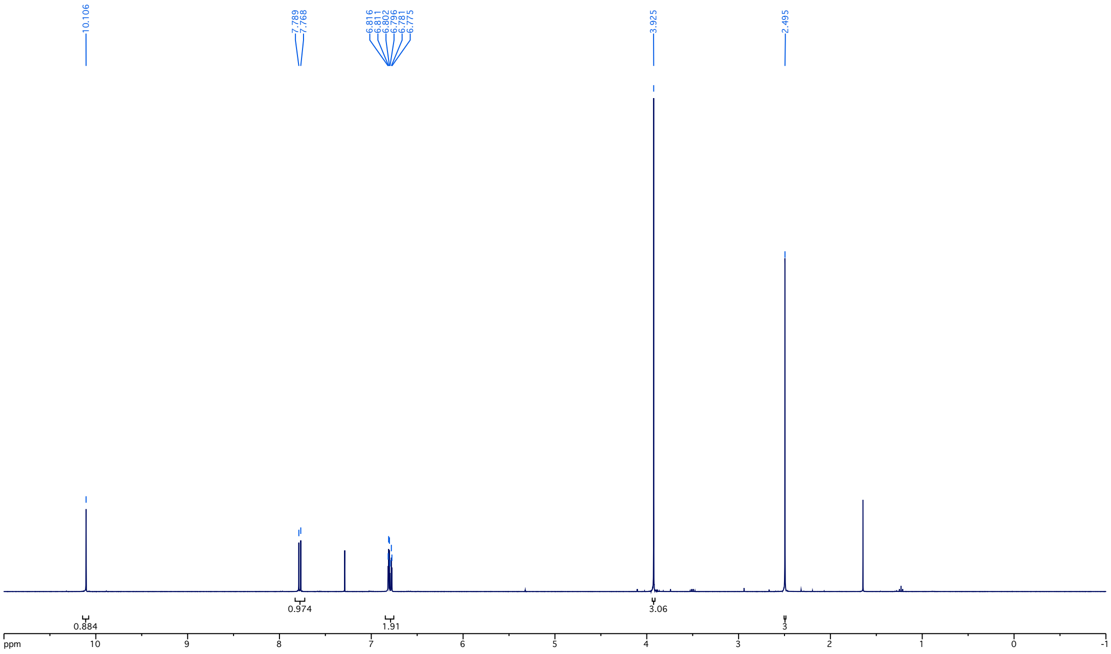

Aldehyde 1k: ^{13}C NMR (101 MHz, $CDCl_3$)

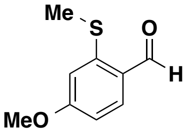

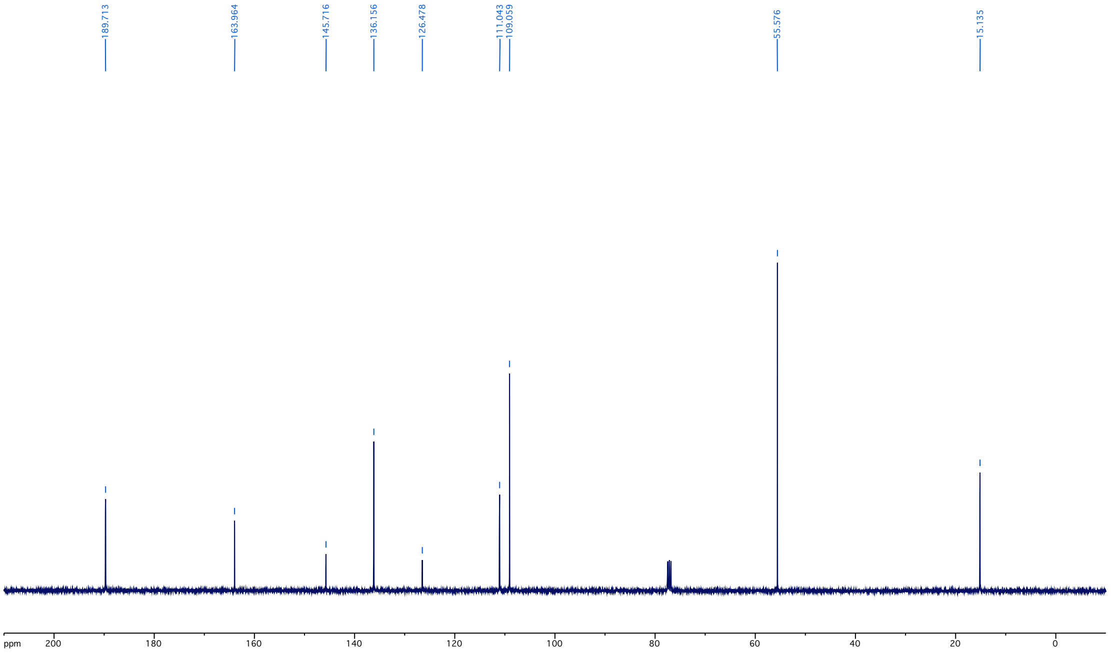

Aldehyde 1l: ^{1}H NMR (400 MHz, $CDCl_3$)

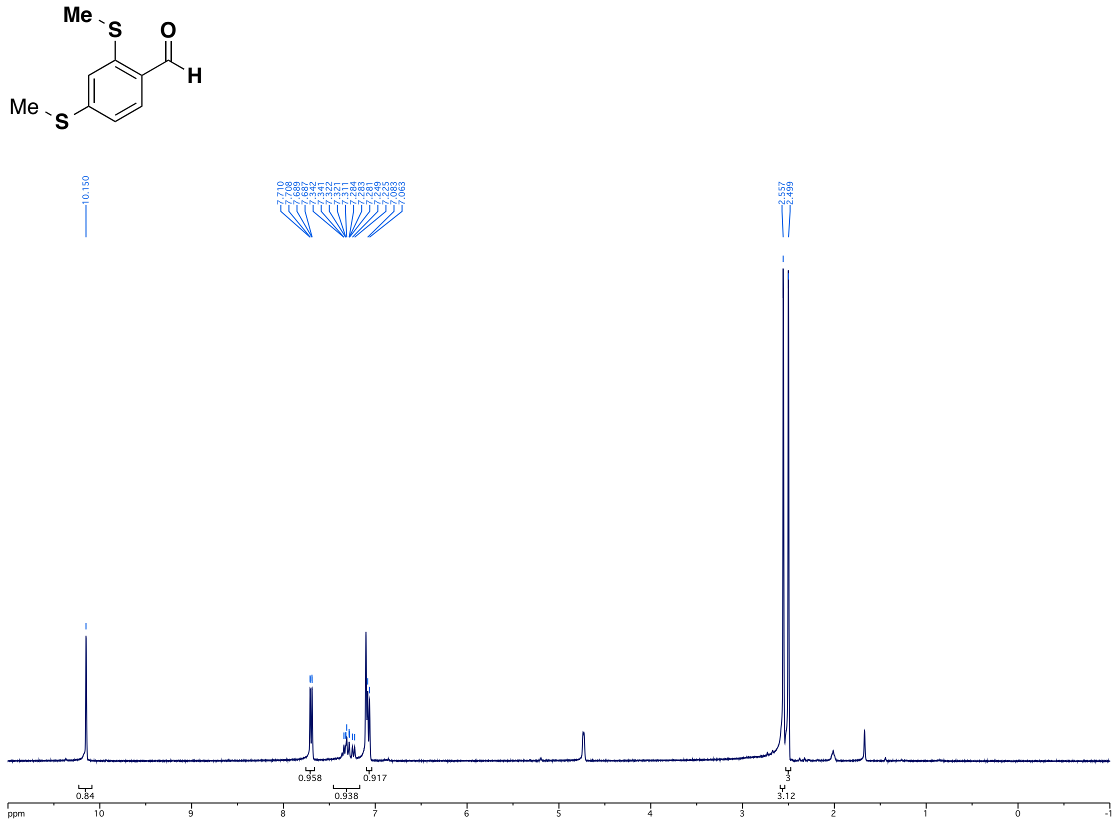

Aldehyde 1l: ^{13}C NMR (101 MHz, $CDCl_3$)

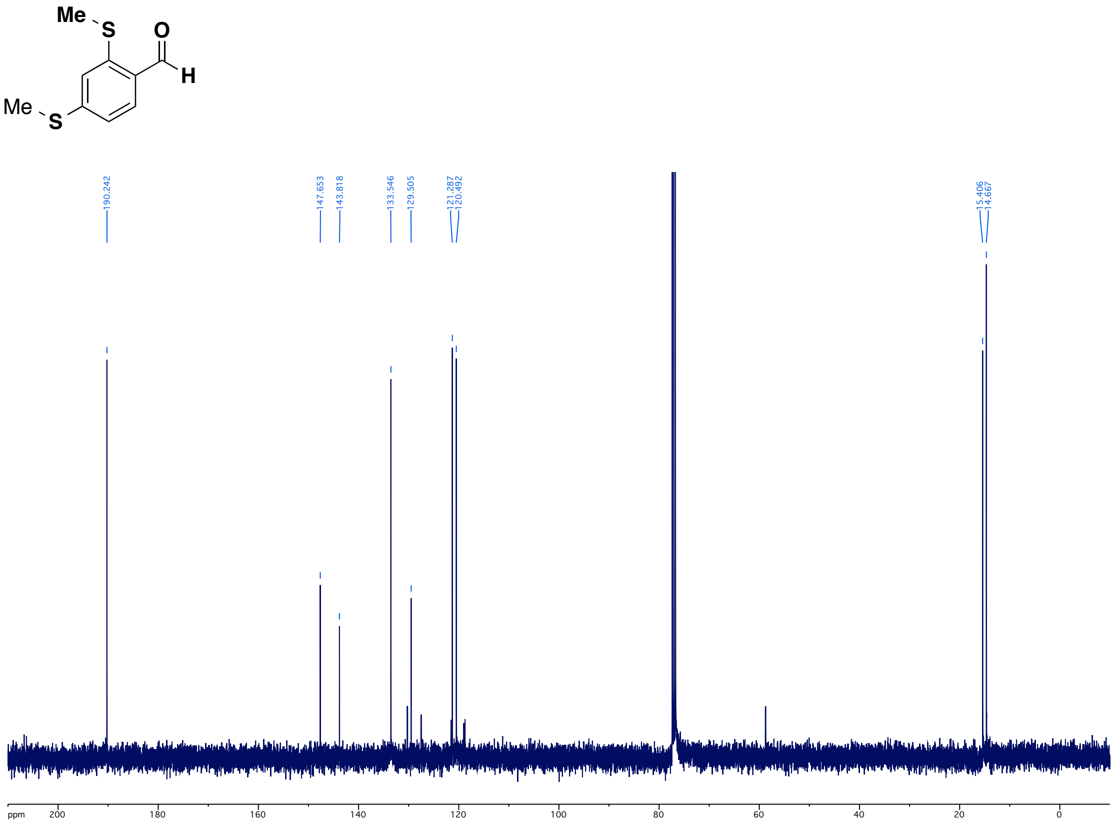

Aldehyde 1m: ^{1}H NMR (400 MHz, $CDCl_3$)

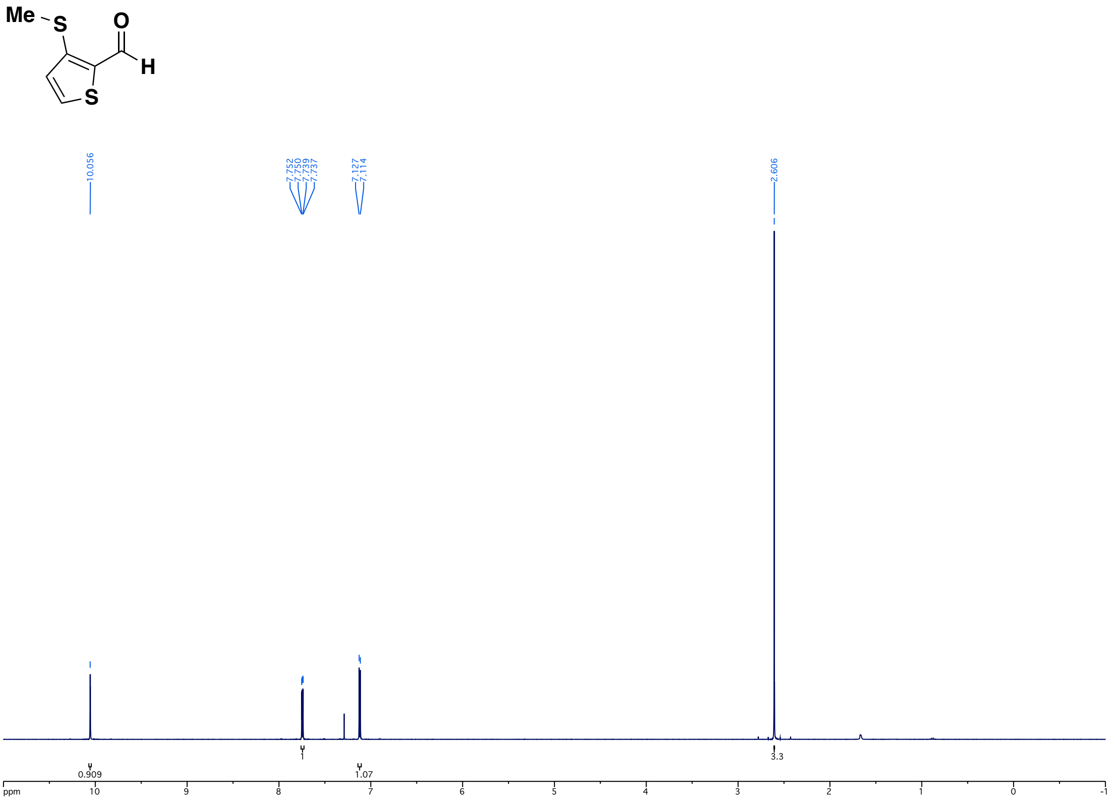

Aldehyde 1m: ^{13}C NMR (101 MHz, $CDCl_3$)

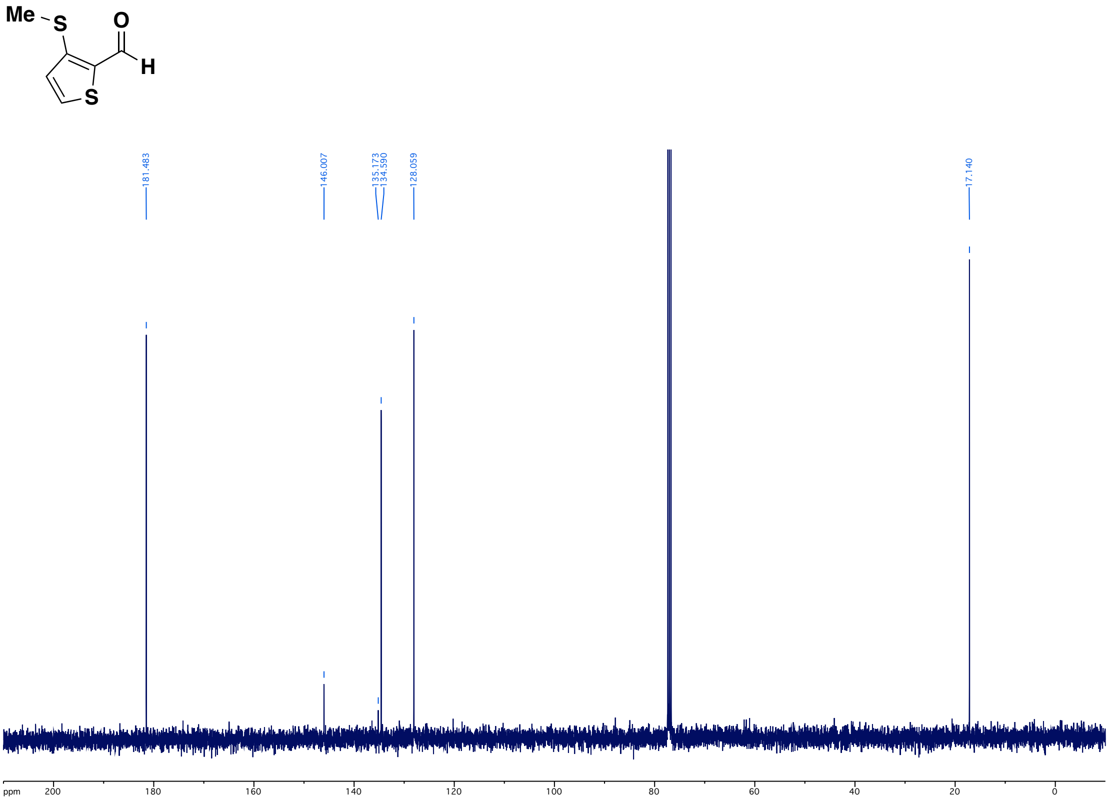

Aldehyde 1n: ^{1}H NMR (400 MHz, $CDCl_3$)

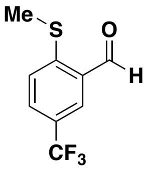

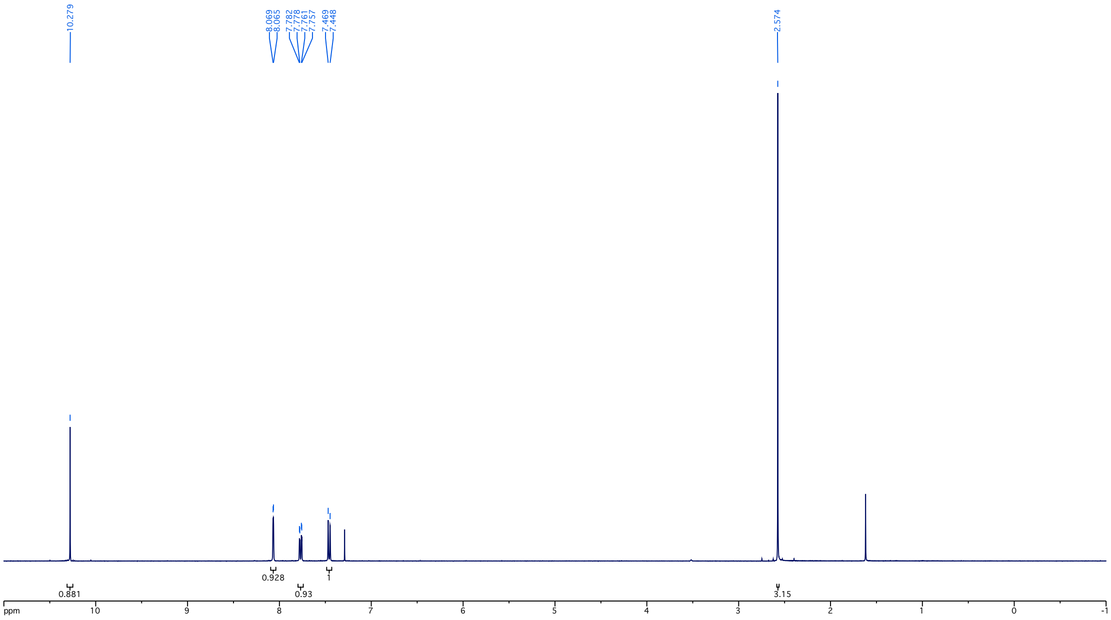

Aldehyde 1n: ^{13}C NMR (126 MHz, $CDCl_3$)

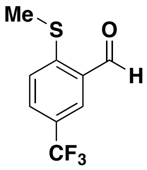

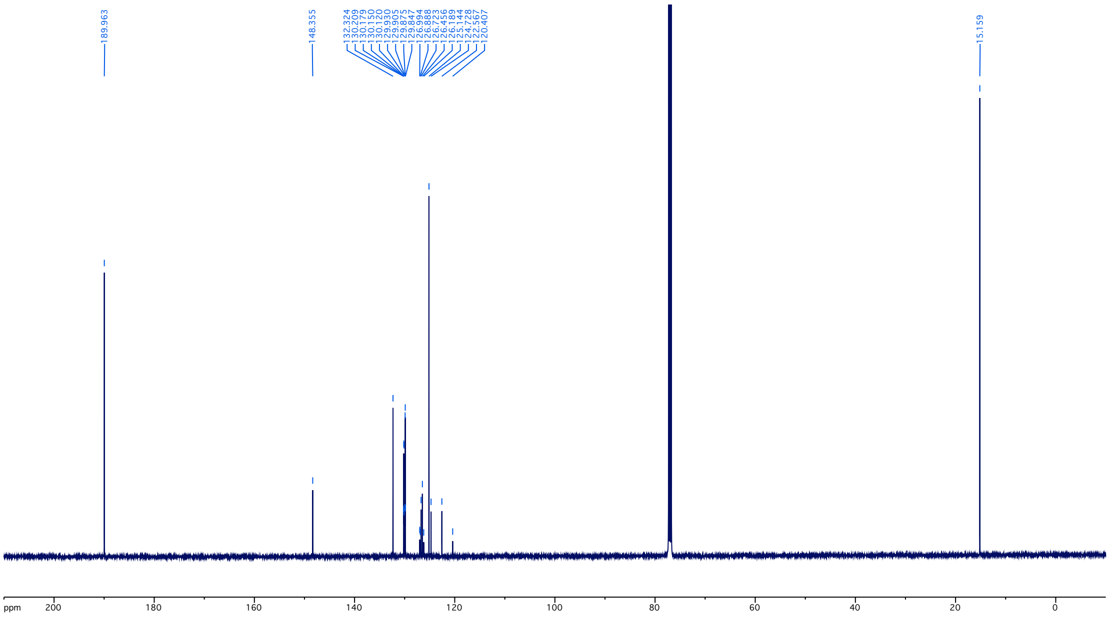

Aldehyde 1n: ^{19}F NMR (377 MHz, $CDCl_3$)

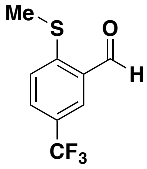

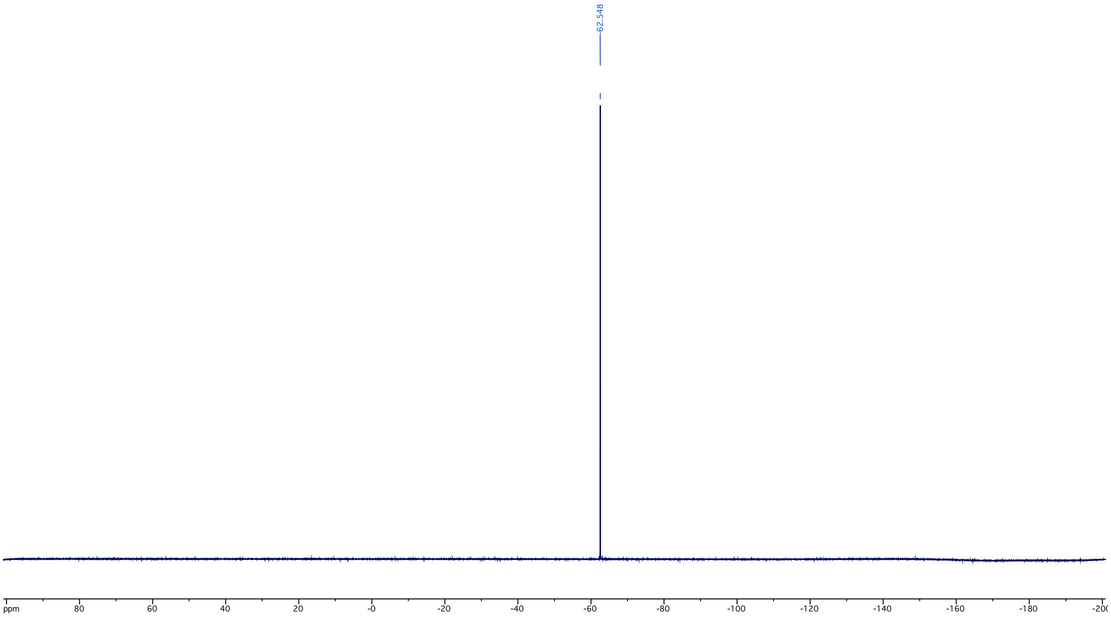

𝛼-Amido sulfone 9j: ^{1}H NMR (400 MHz, $CDCl_3$):

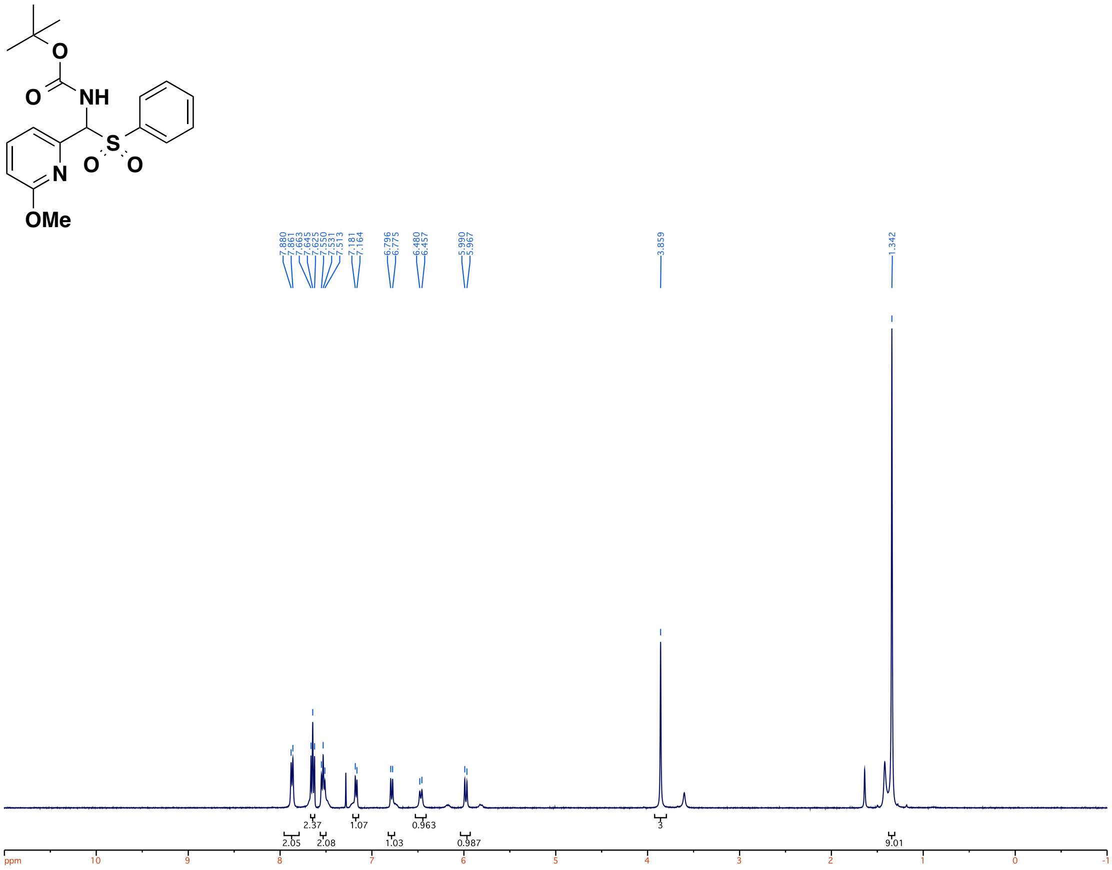

𝛼-Amido sulfone 9j: ^{13}C NMR (101 MHz, $CDCl_3$):

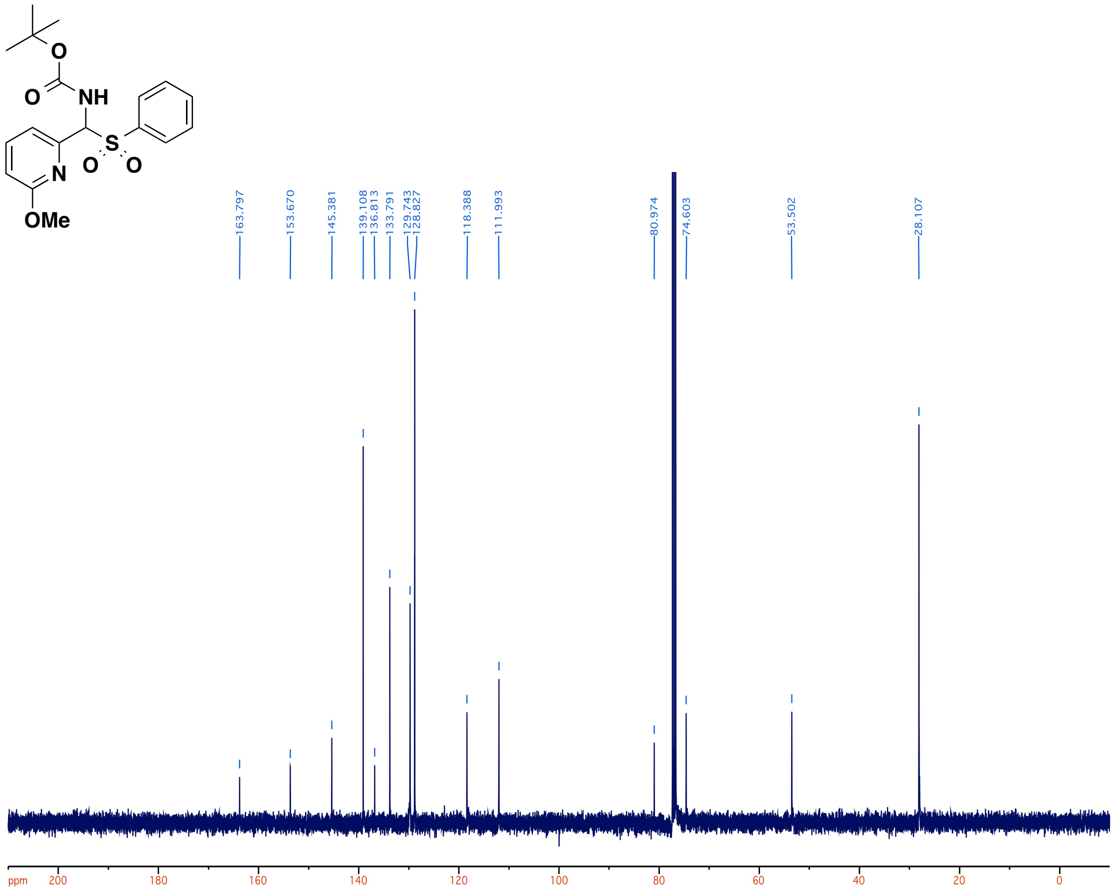

α-Amido sulfone 9k: ^{1}H NMR (400 MHz, CD_2Cl_2)

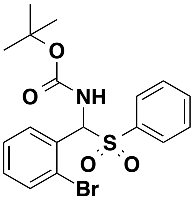

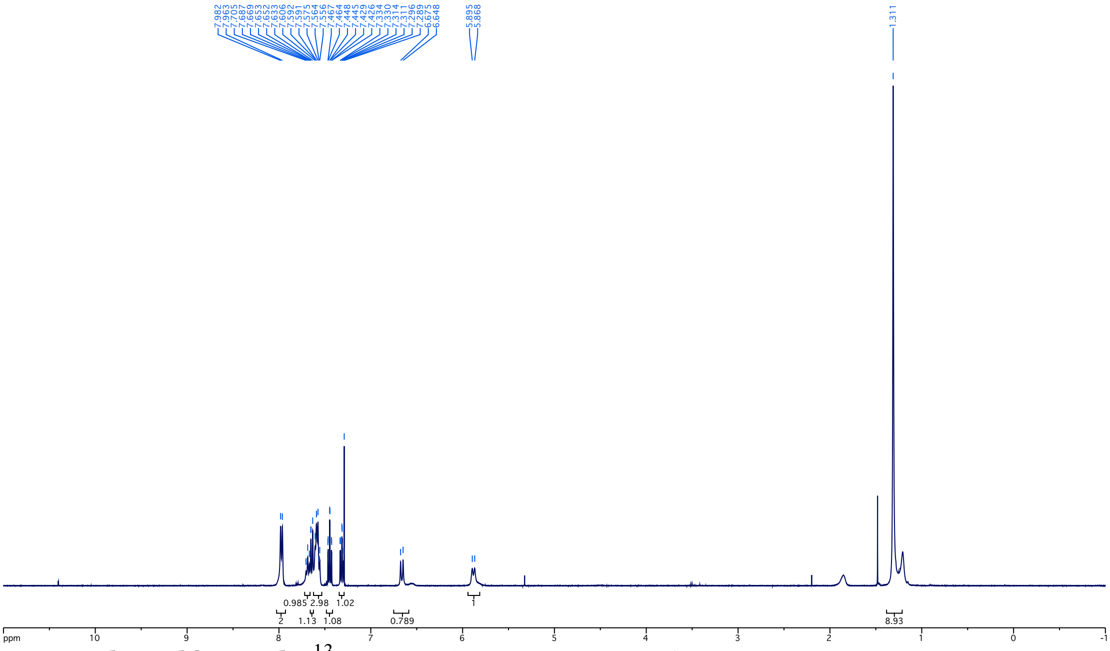

α-Amido sulfone 9k: ^{13}C NMR (101 MHz, CD_2Cl_2)

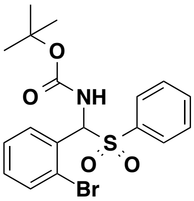

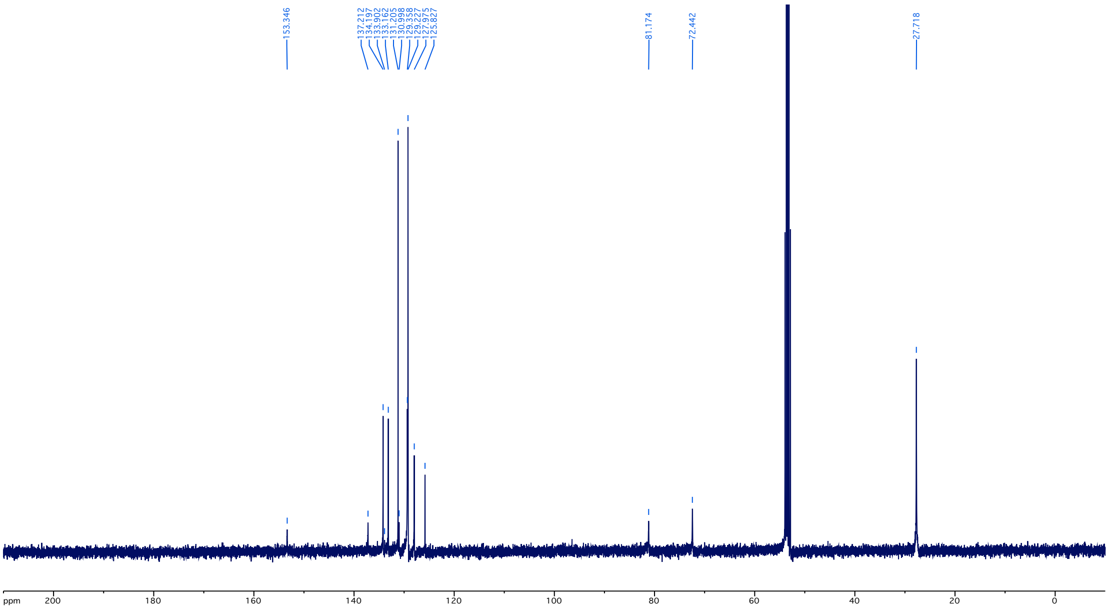

α-Amido Sulfone 9l: ^{1}H NMR (400 MHz, $CDCl_3$)

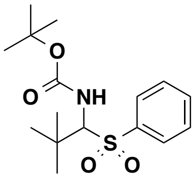

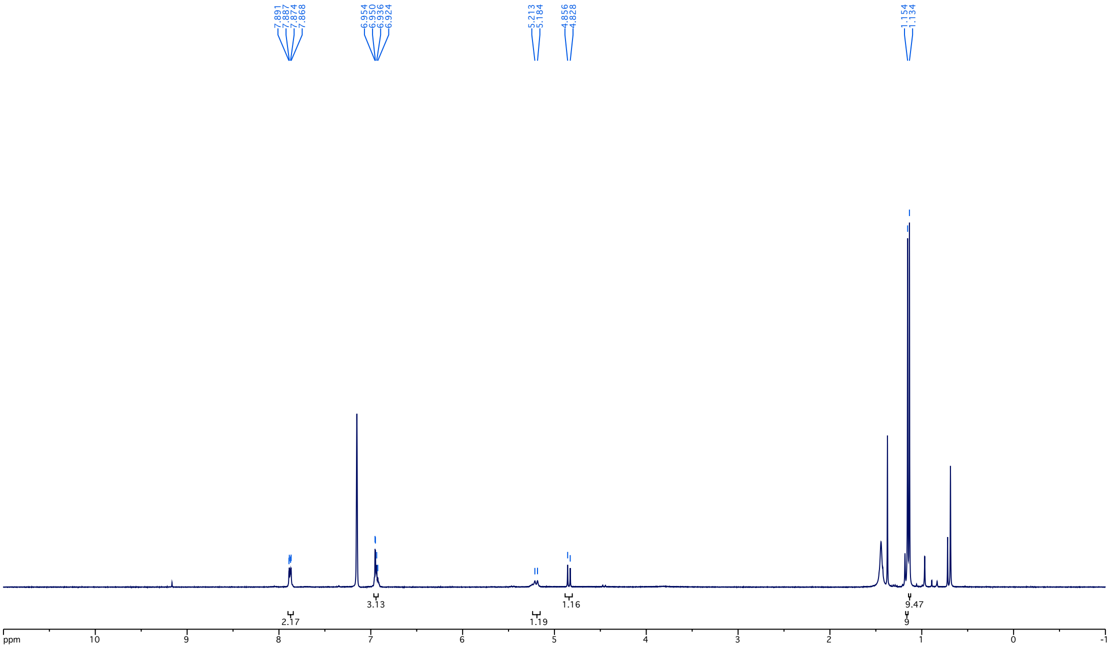

α-Amido Sulfone 9l: ^{13}C NMR (101 MHz, $CDCl_3$)

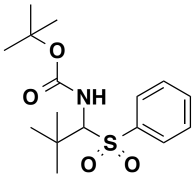

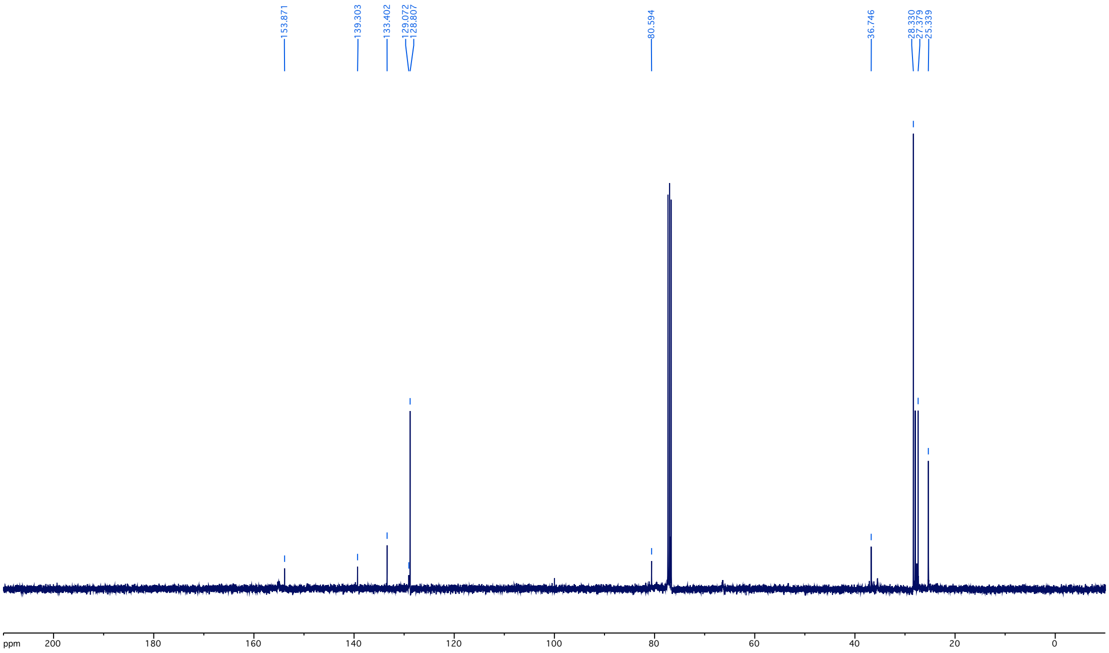

α-Amido Sulfone 9m: ^{1}H NMR (400 MHz, $CDCl_3$)

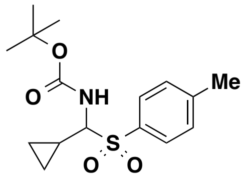

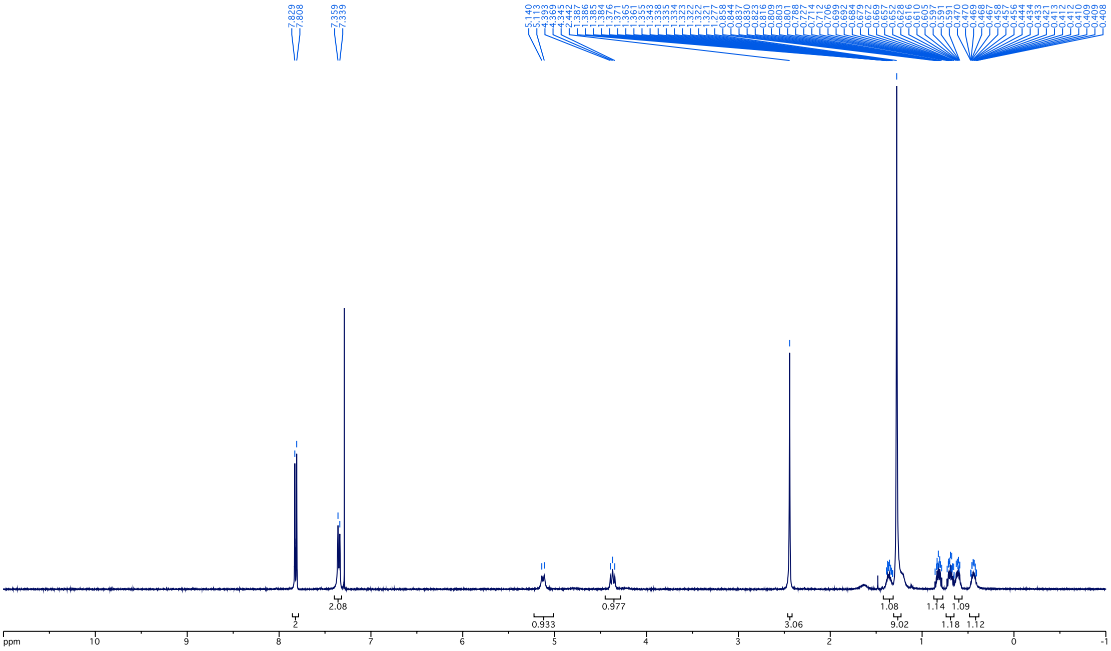

α-Amido Sulfone 9m: ^{13}C NMR (101 MHz, $CDCl_3$)

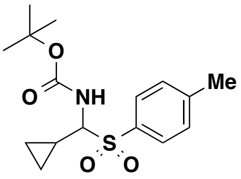

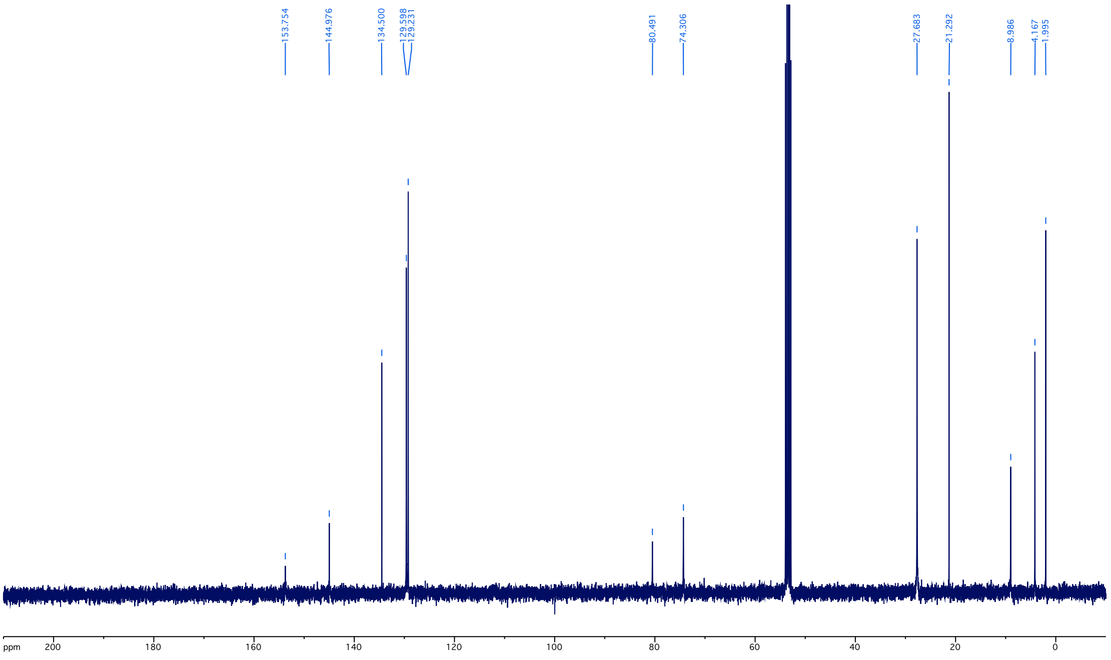

Propargylic Amine 2b: ^{1}H NMR (400 MHz, $CDCl_3$)

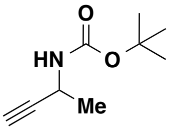

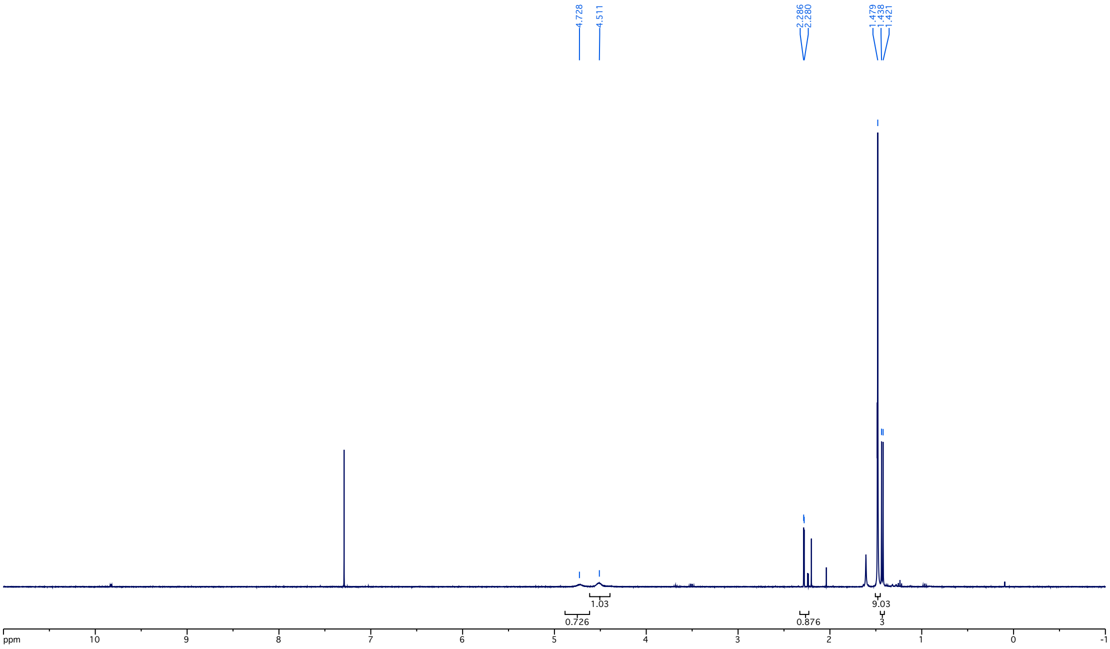

Propargylic Amine 2b: ^{13}C NMR (101 MHz, $CDCl_3$)

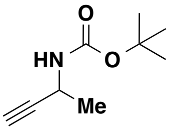

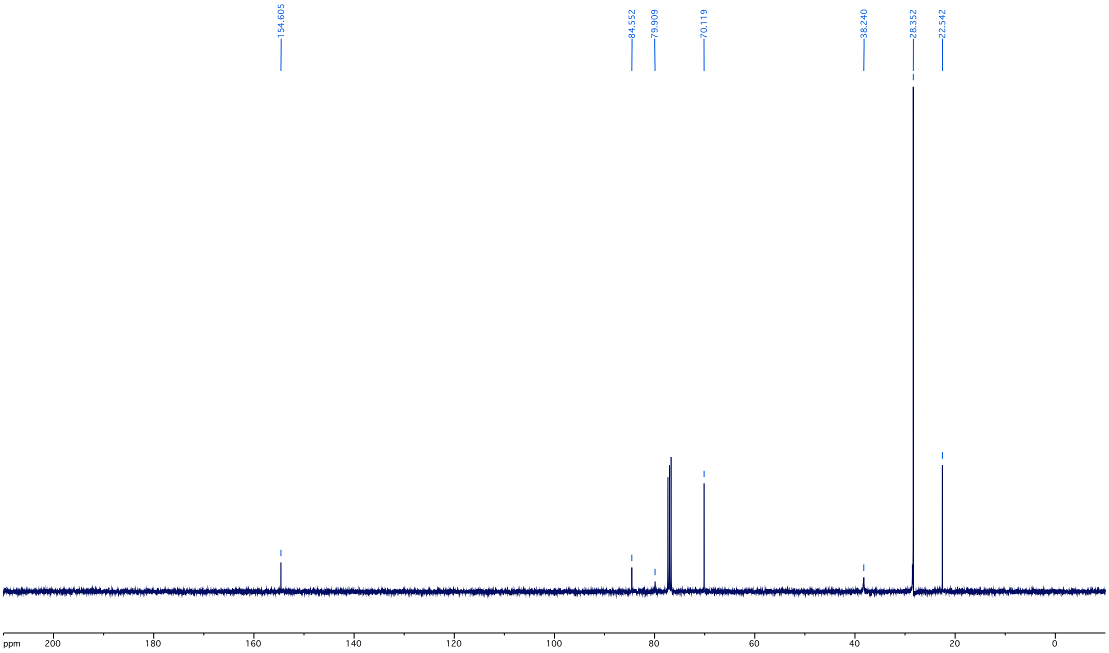

Propargylic Amine 2c: ^{1}H NMR (400 MHz, $CDCl_3$)

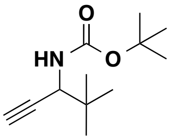

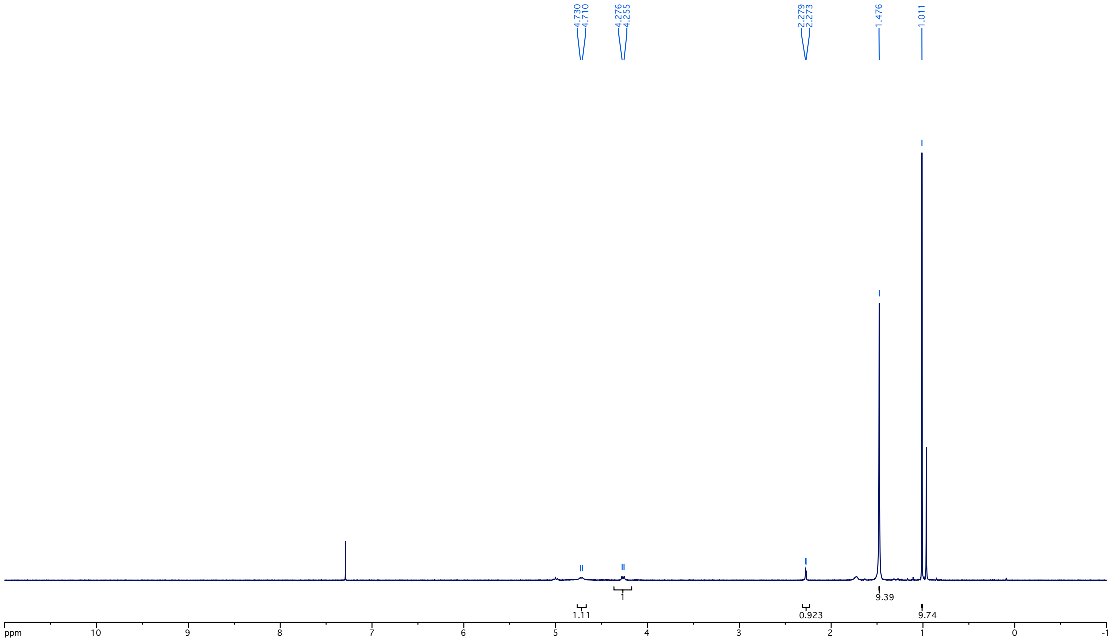

Propargylic Amine 2c: ^{13}C NMR (101 MHz, $CDCl_3$)

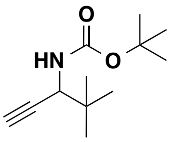

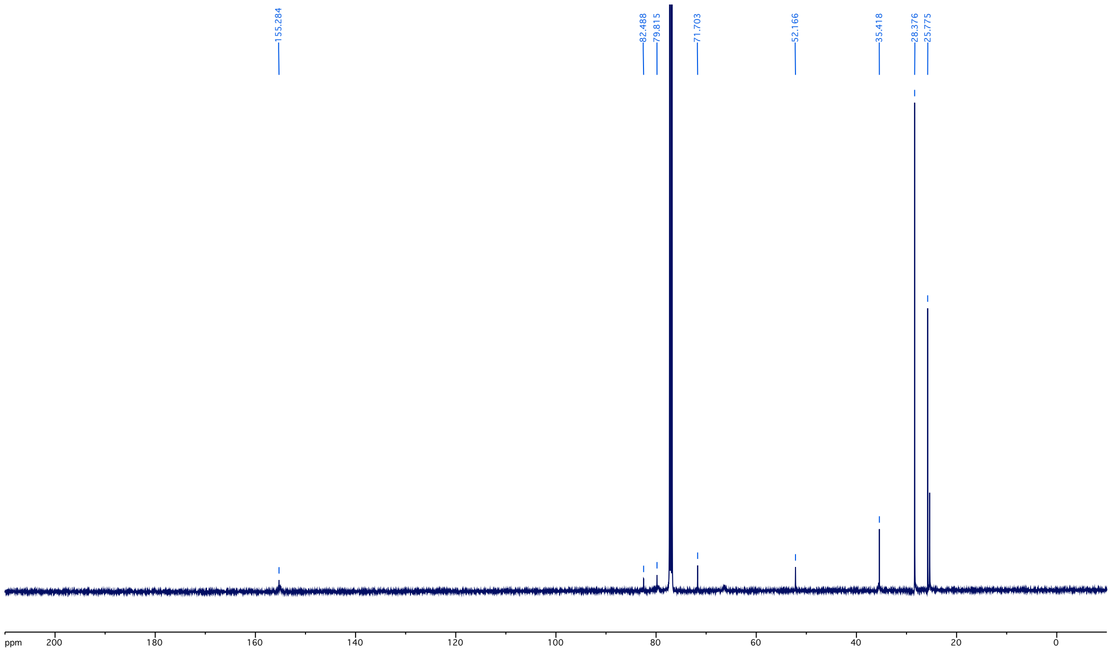

Propargylic Amine 2d: ^{1}H NMR (400 MHz, $CDCl_3$)

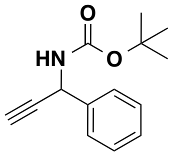

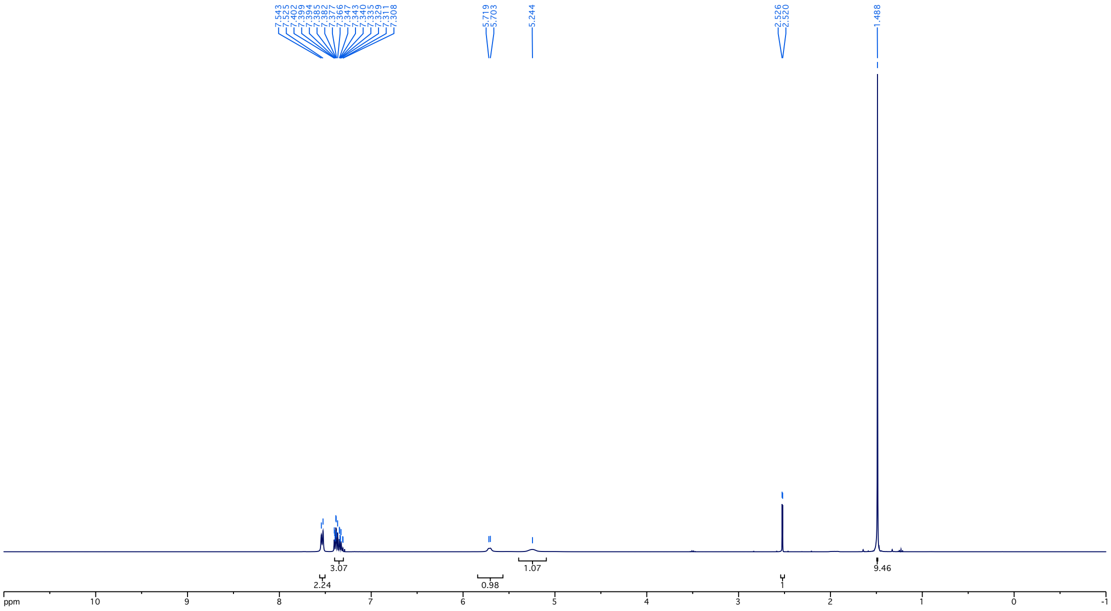

Propargylic Amine 2d: ^{13}C NMR (101 MHz, $CDCl_3$)

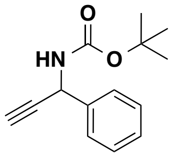

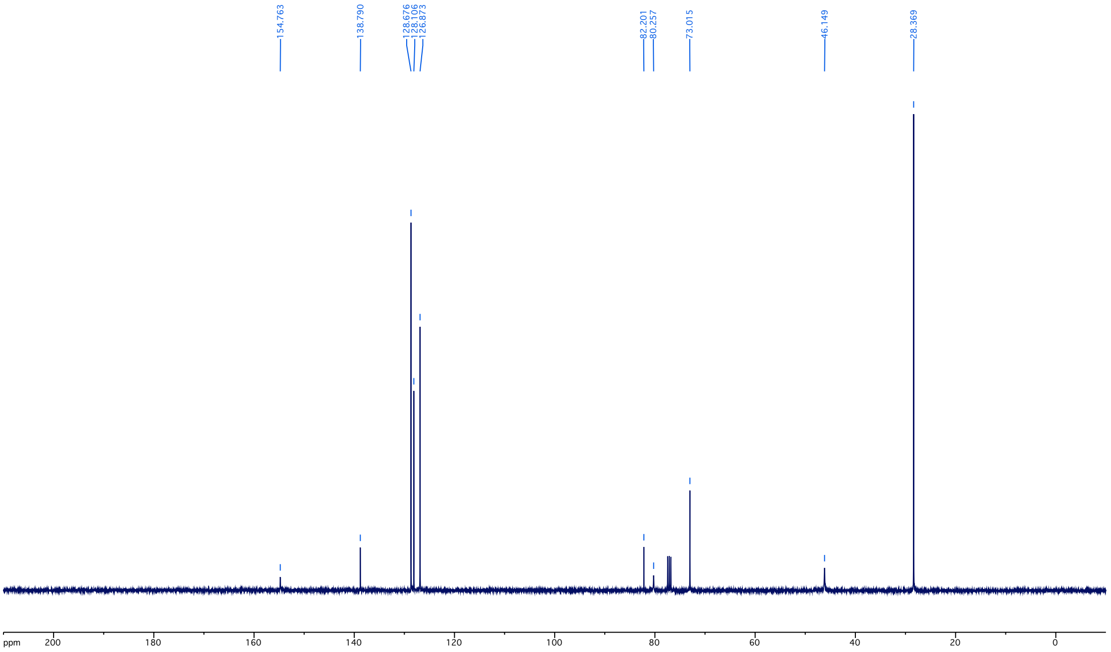

Propargylic Amine 2e: ^{1}H NMR (400 MHz, $CDCl_3$)

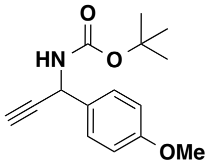

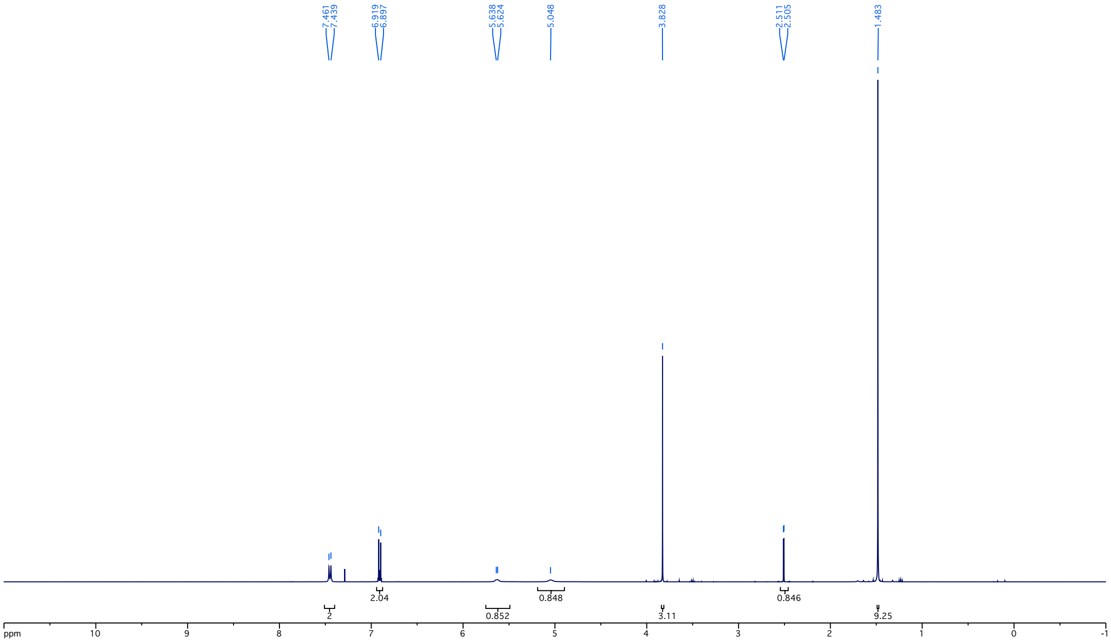

Propargylic Amine 2e: ^{13}C NMR (101 MHz, $CDCl_3$)

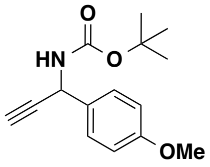

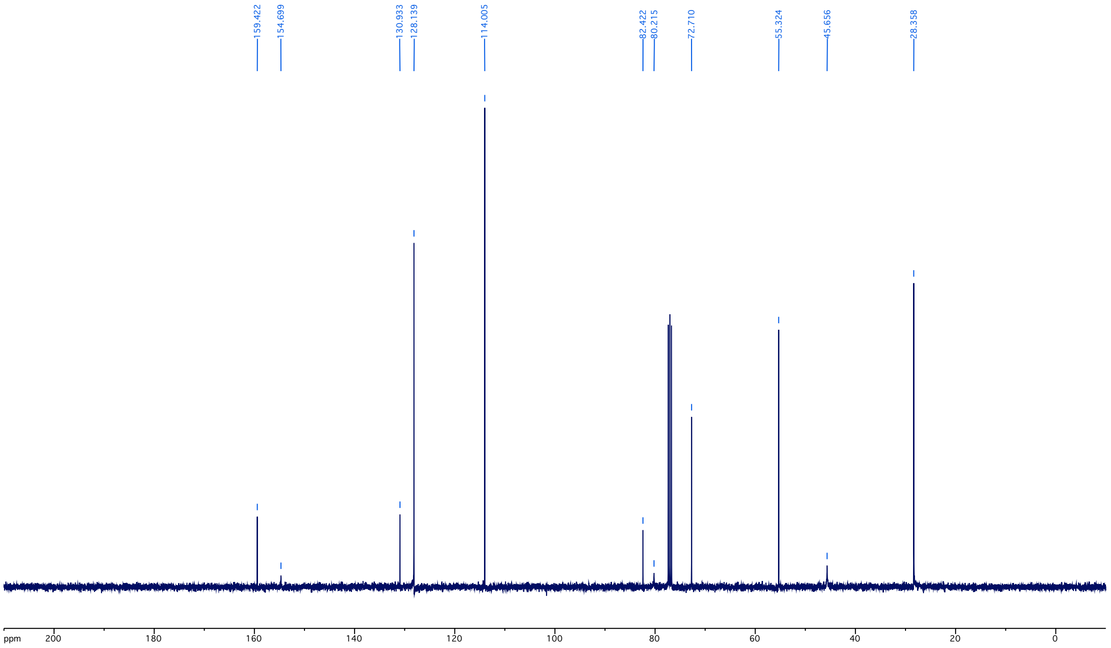

Propargylic Amine 2f: ^{1}H NMR (400 MHz, $CDCl_3$)

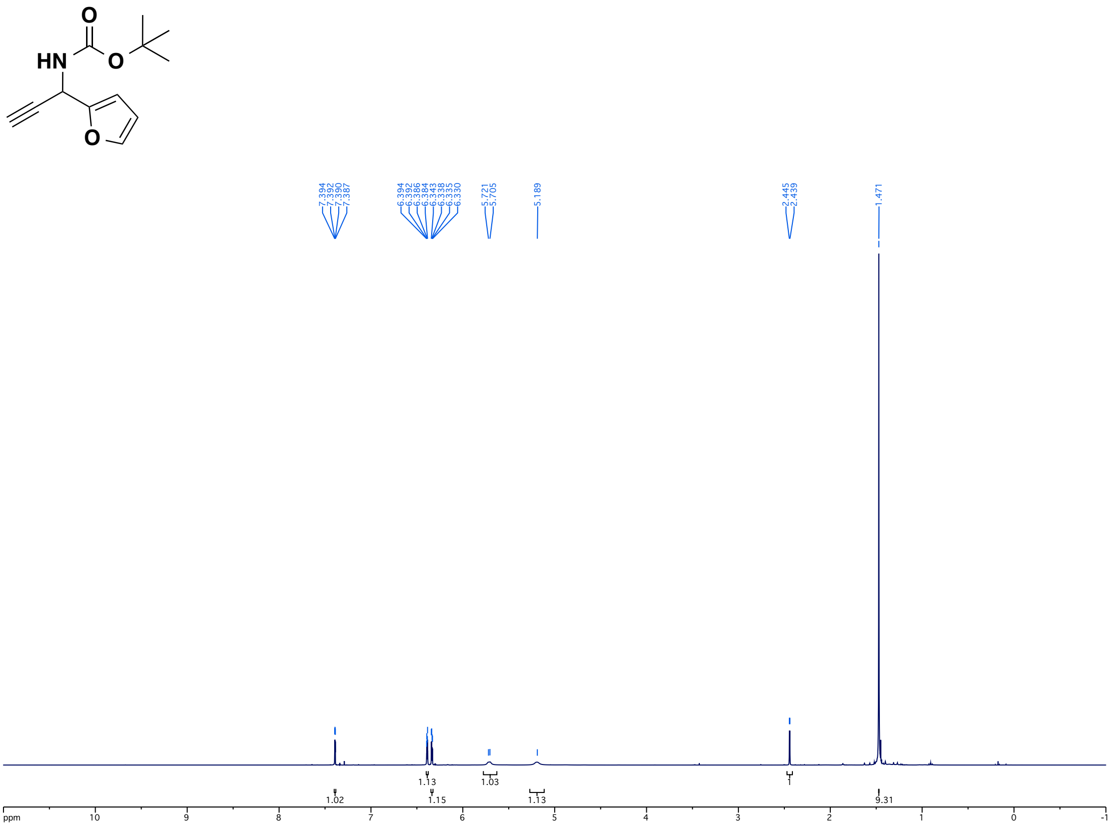

Propargylic Amine 2f: ^{13}C NMR (101 MHz, $CDCl_3$)

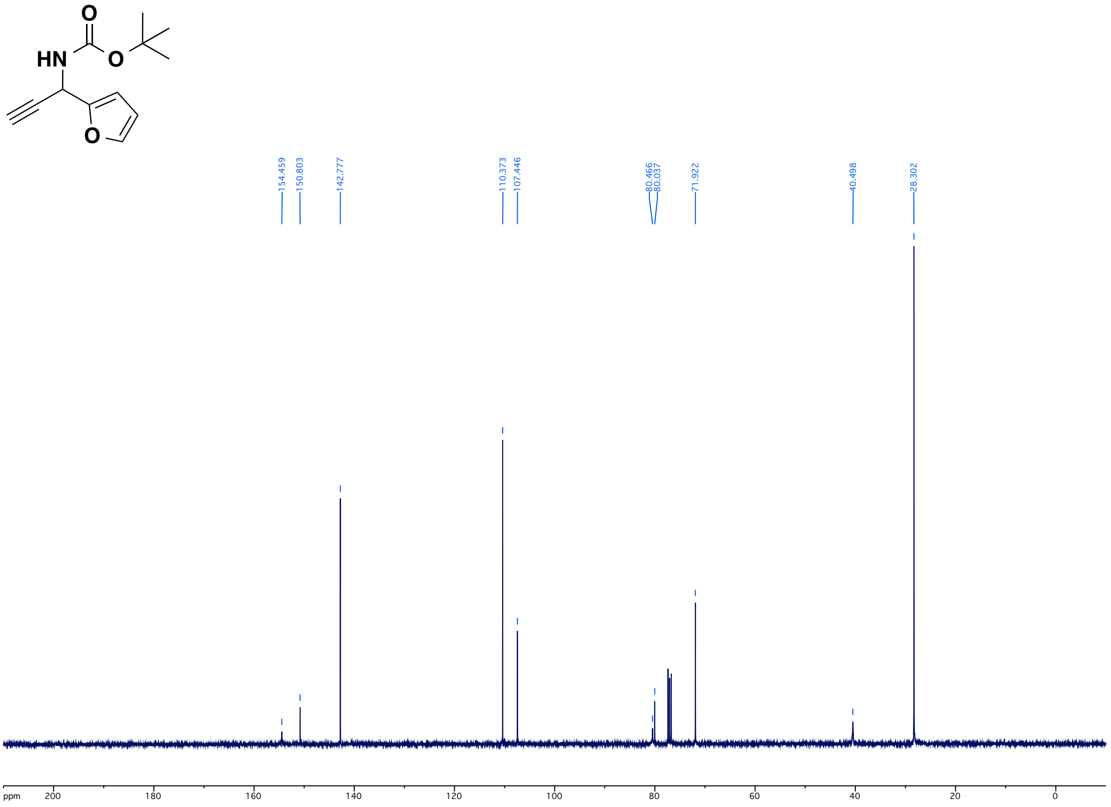

Propargylic Amine 2g: ^{1}H NMR (400 MHz, $CDCl_3$)

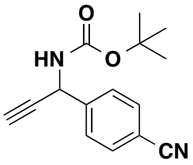

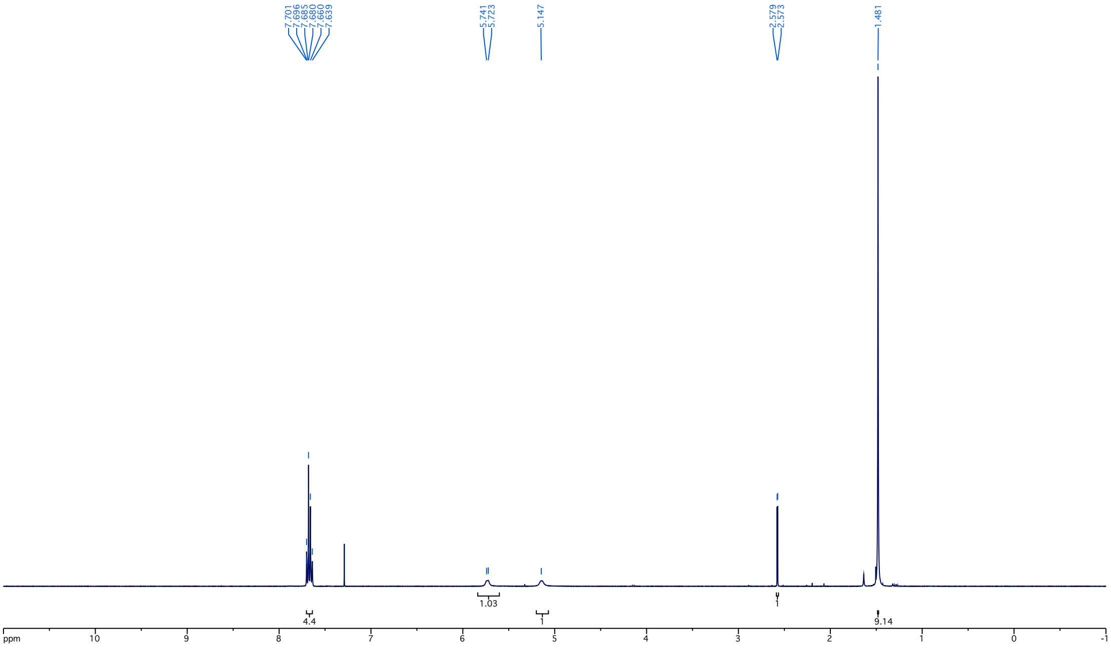

Propargylic Amine 2g: ^{13}C NMR (101 MHz, $CDCl_3$)

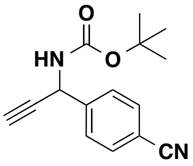

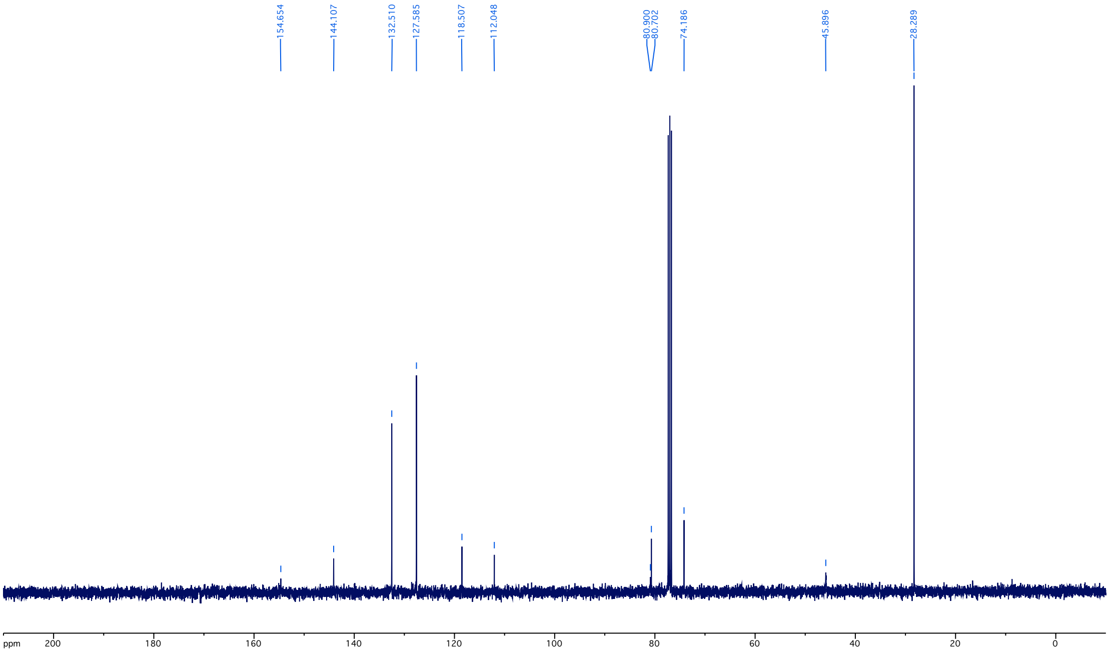

Propargylic Amine 2h: ^{1}H NMR (400 MHz, $CDCl_3$)

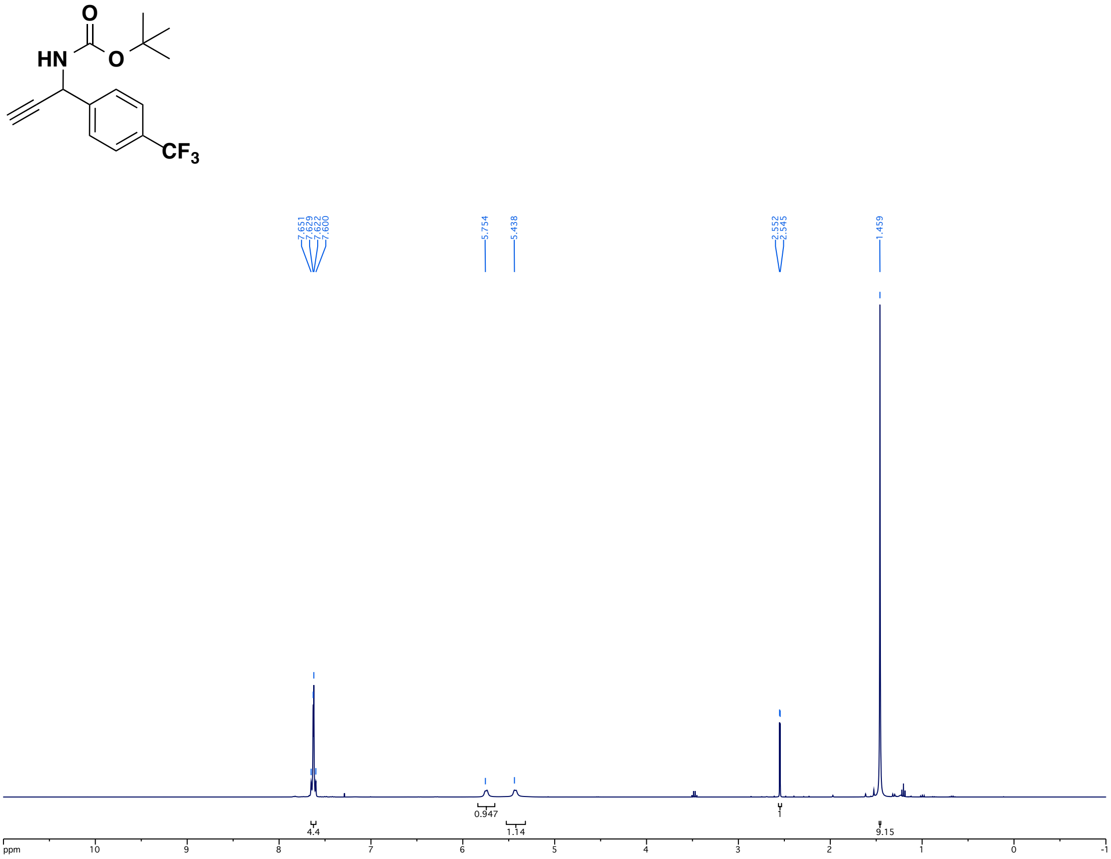

Propargylic Amine 2h: ^{13}C NMR (101 MHz, $CDCl_3$)

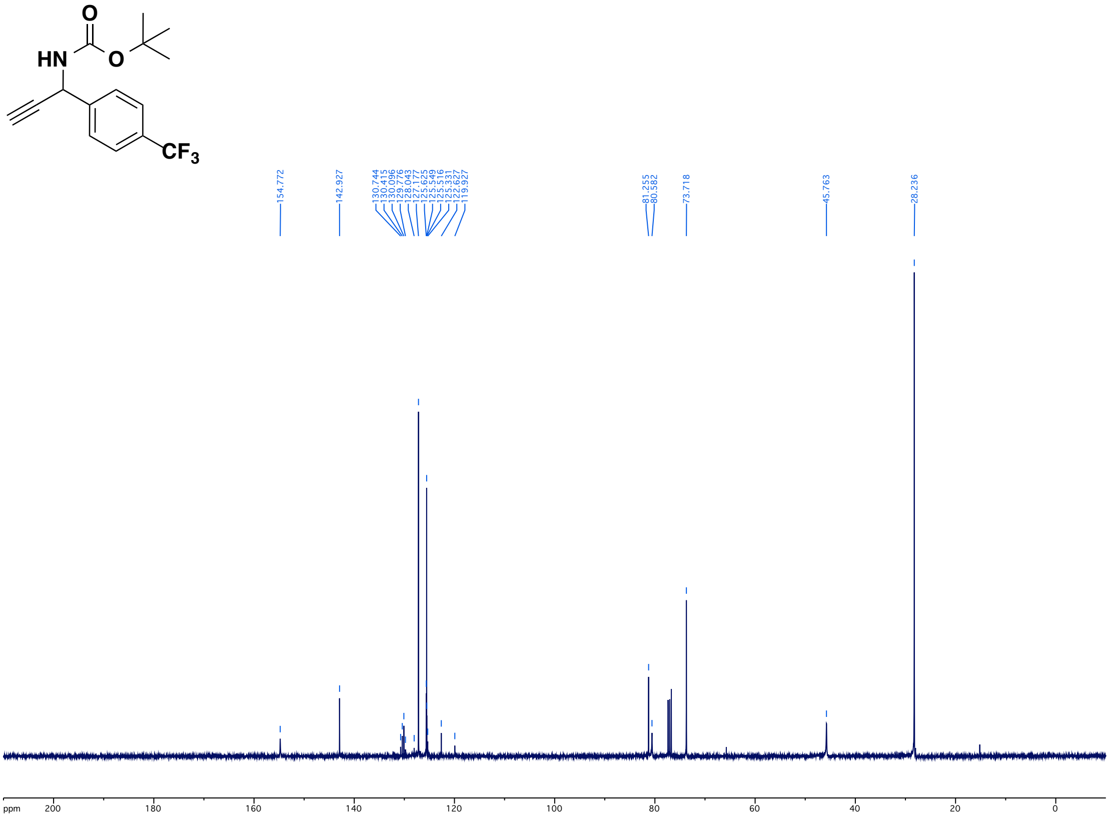

Propargylic Amine 2h: ^{19}F NMR (377 MHz, $CDCl_3$)

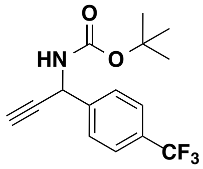

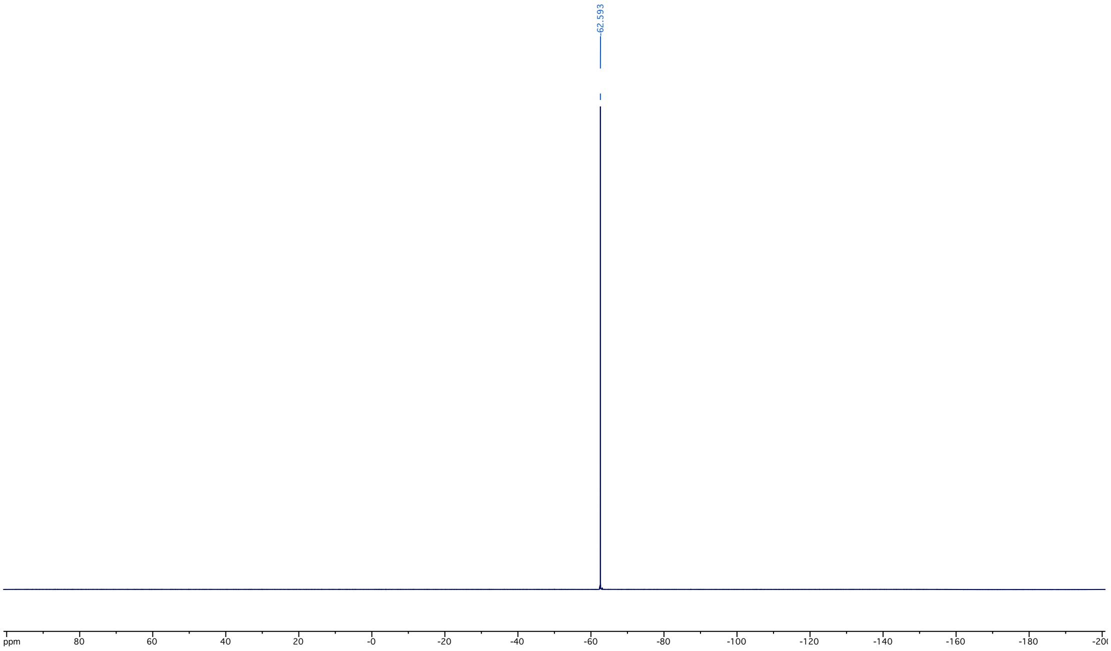

Propargylic Amine 2i: ^{1}H NMR (400 MHz, $CDCl_3$)

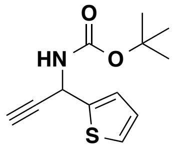

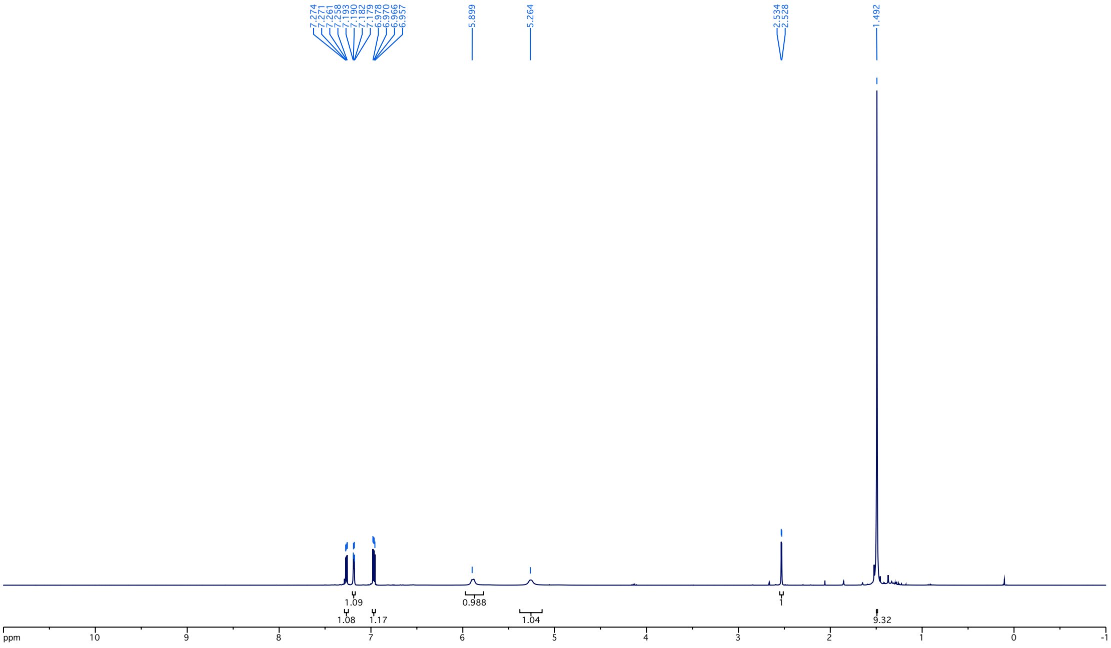

Propargylic Amine 2i: ^{13}C NMR (101 MHz, $CDCl_3$)

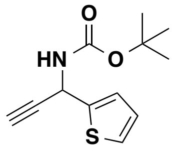

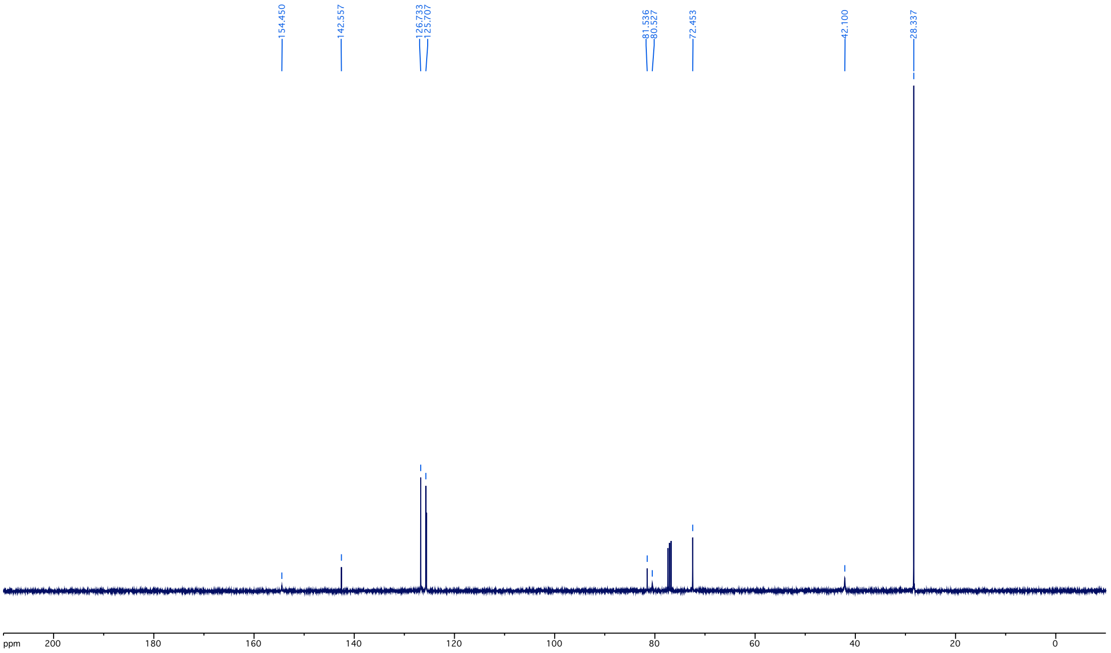

Propargylic Amine 2j: ^{1}H NMR (400 MHz, $CDCl_3$)

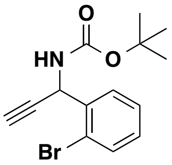

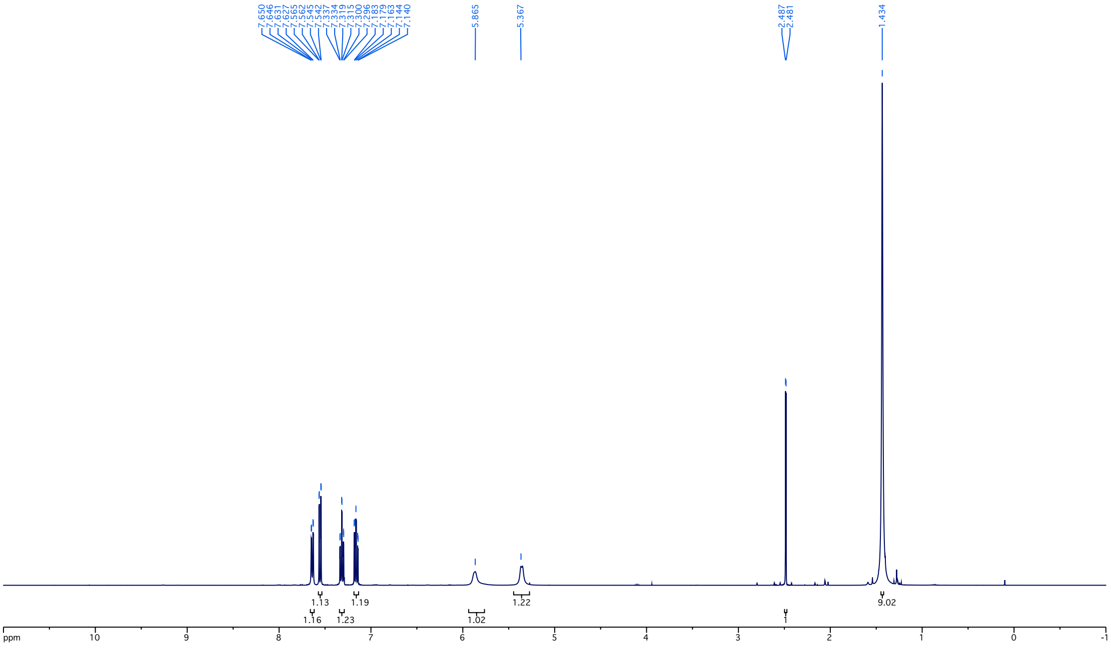

Propargylic Amine 2j: ^{13}C NMR (101 MHz, $CDCl_3$)

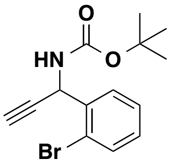

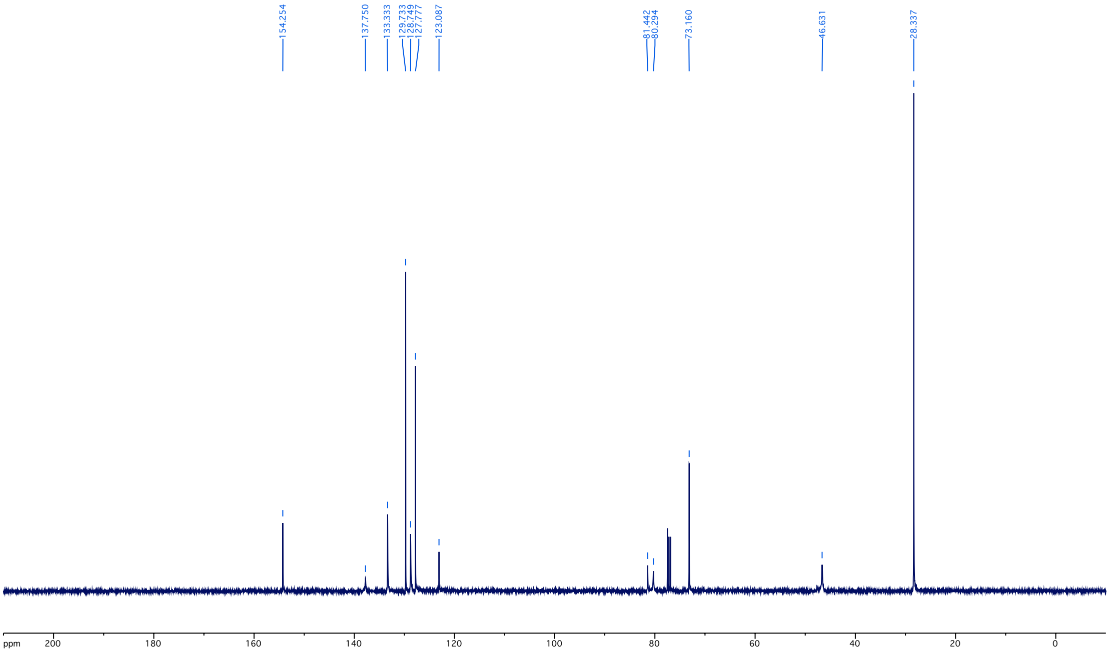

Propargylic Amine 2k: ^{1}H NMR (400 MHz, $CDCl_3$)

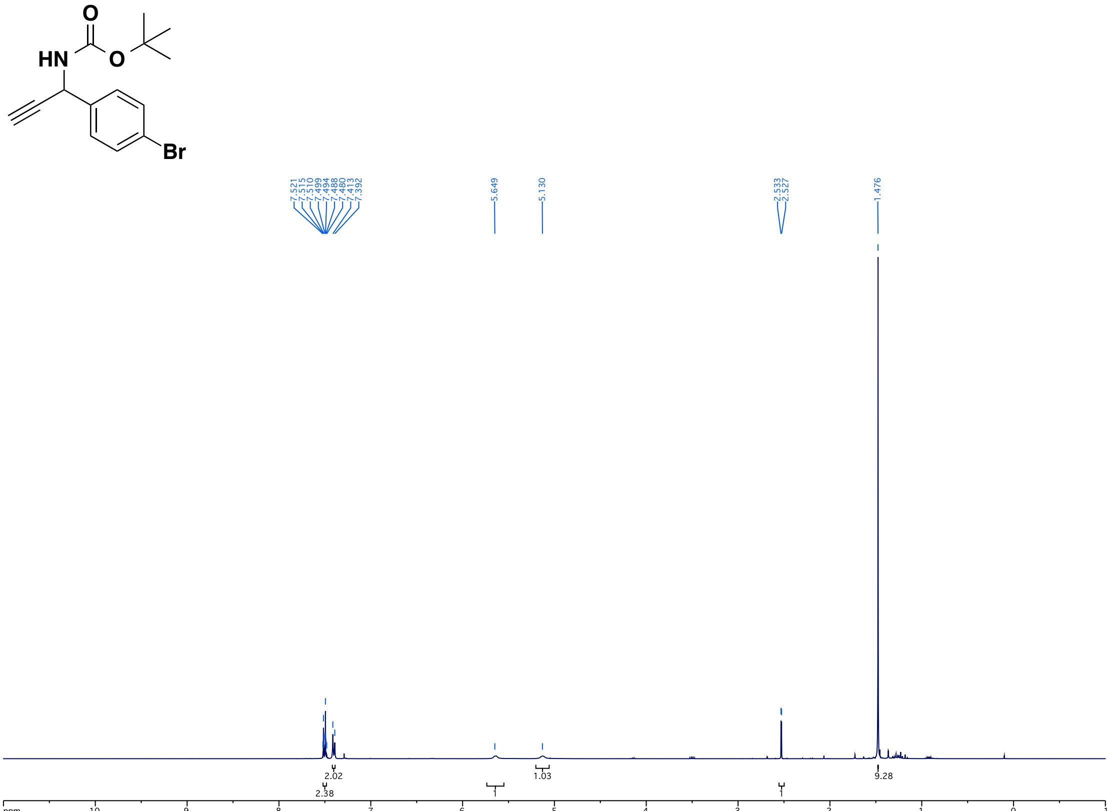

Propargylic Amine 2k: ^{13}C NMR (101 MHz, $CDCl_3$)

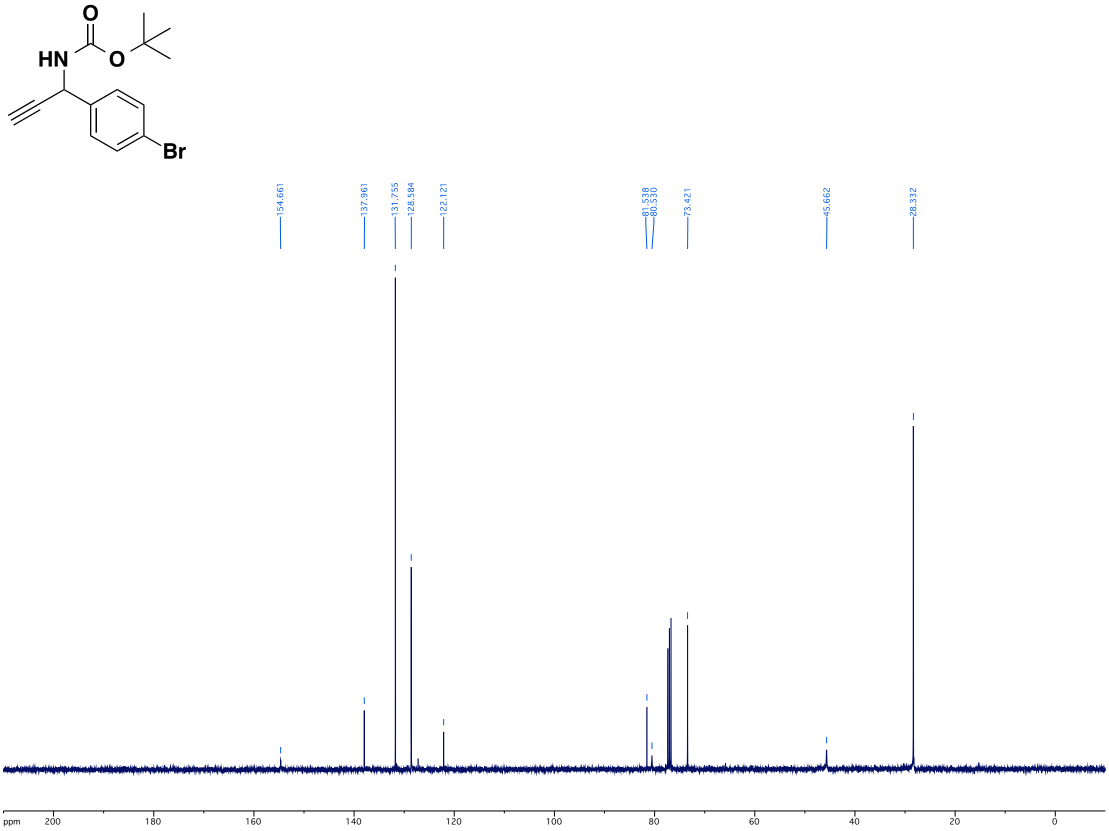

Propargylic Amine 2l: ^{1}H NMR (400 MHz, $CDCl_3$)

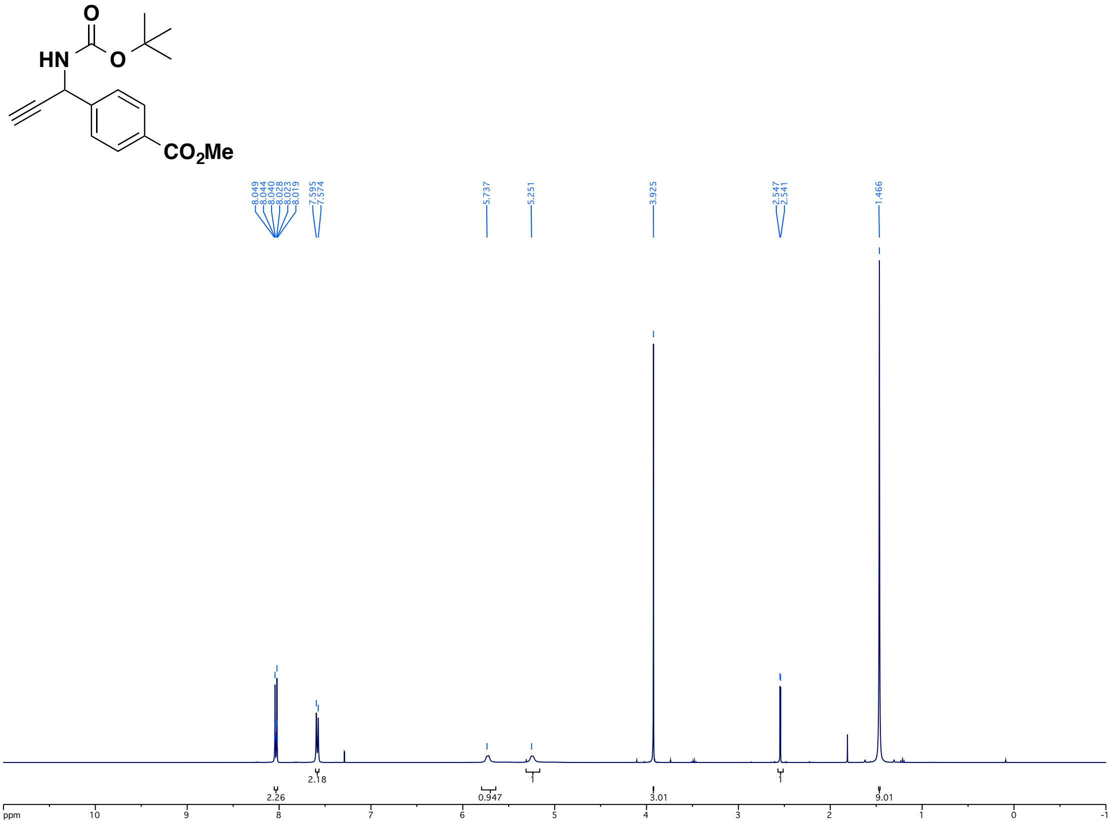

Propargylic Amine 2l: ^{13}C NMR (101 MHz, $CDCl_3$)

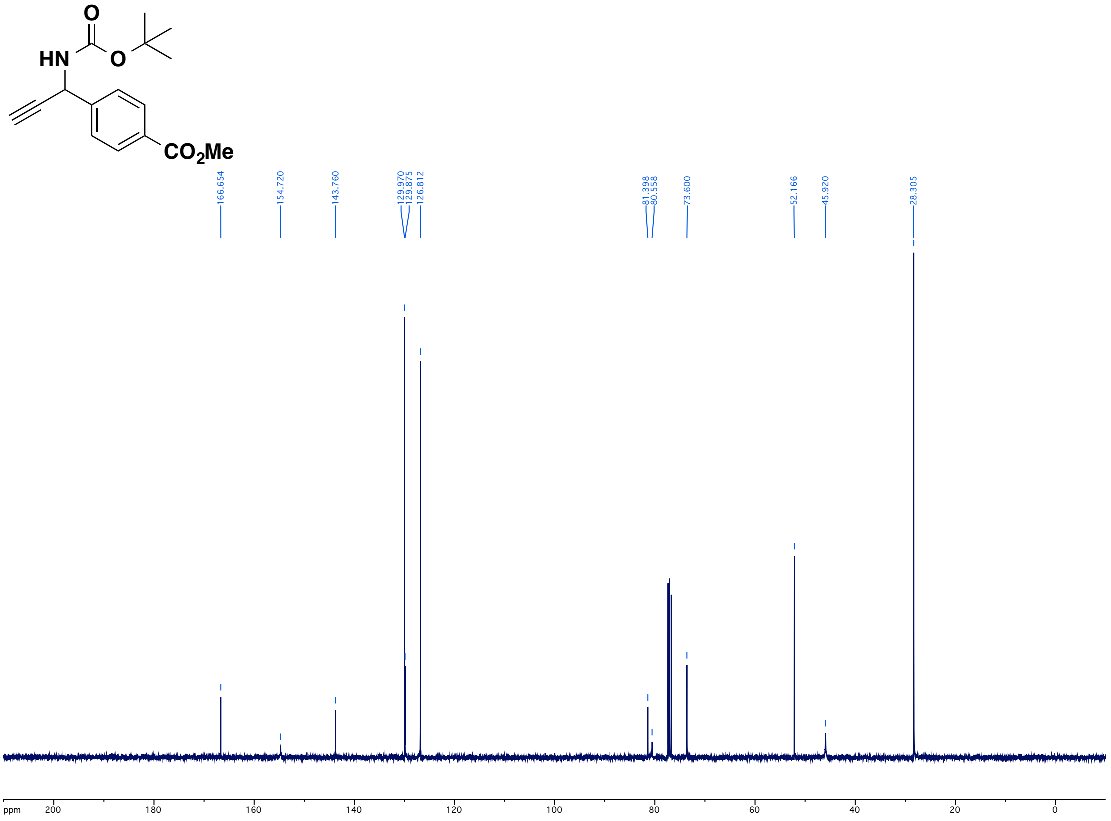

Propargylic Amine 2m: ^{1}H NMR (400 MHz, $CDCl_3$)

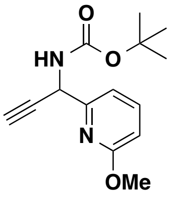

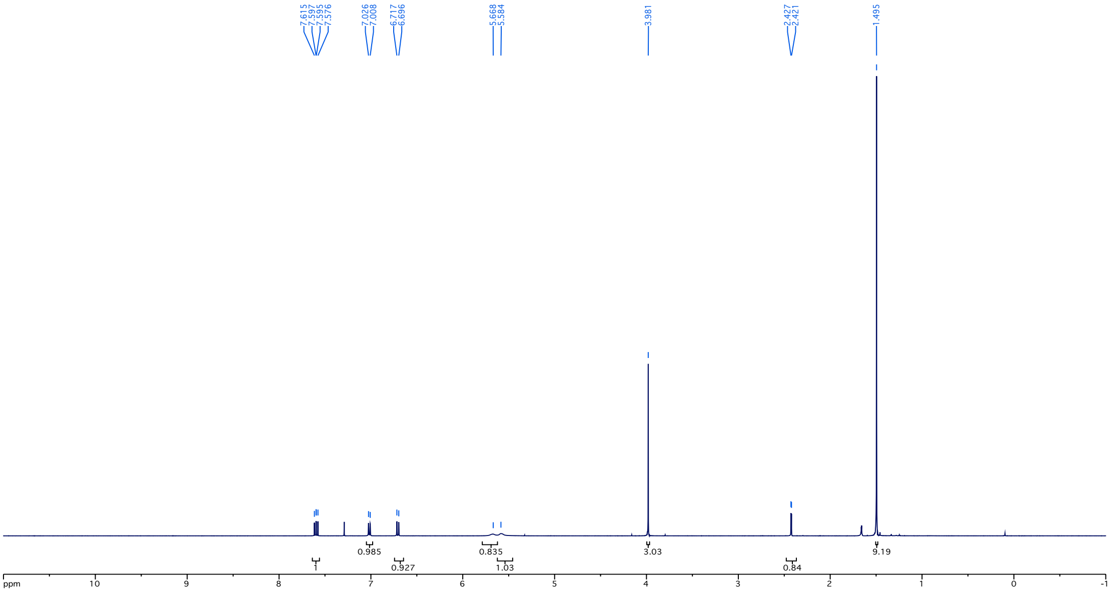

Propargylic Amine 2m: ^{13}C NMR (101 MHz, $CDCl_3$)

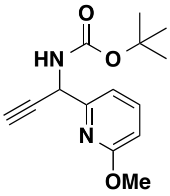

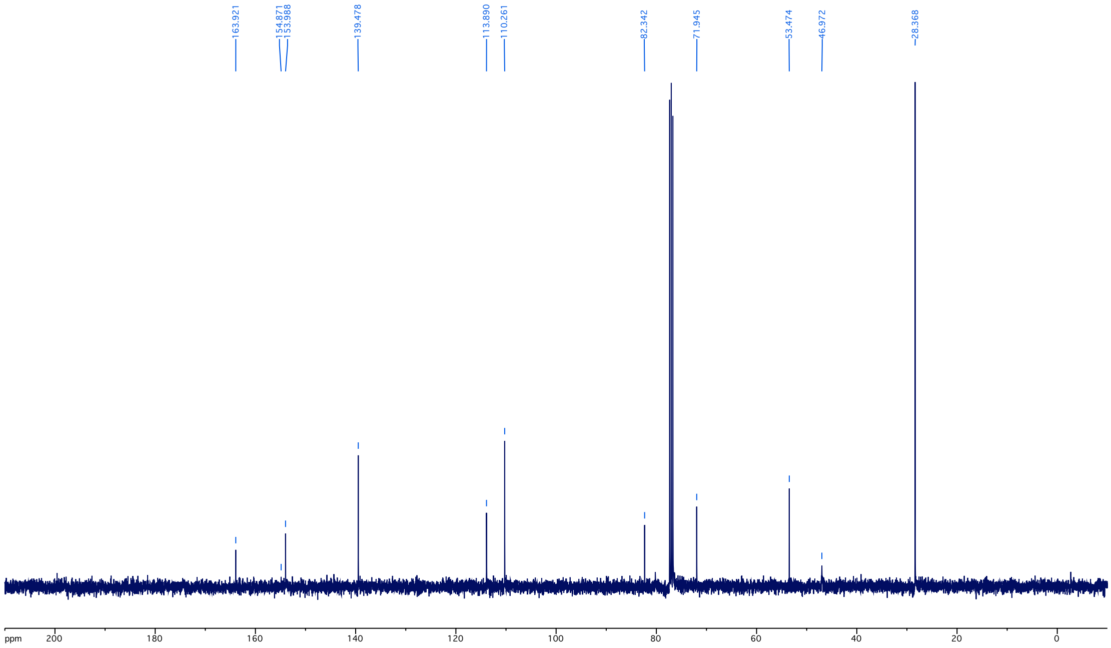

Propargylic Amine 2n: ^{1}H NMR (400 MHz, $CDCl_3$)

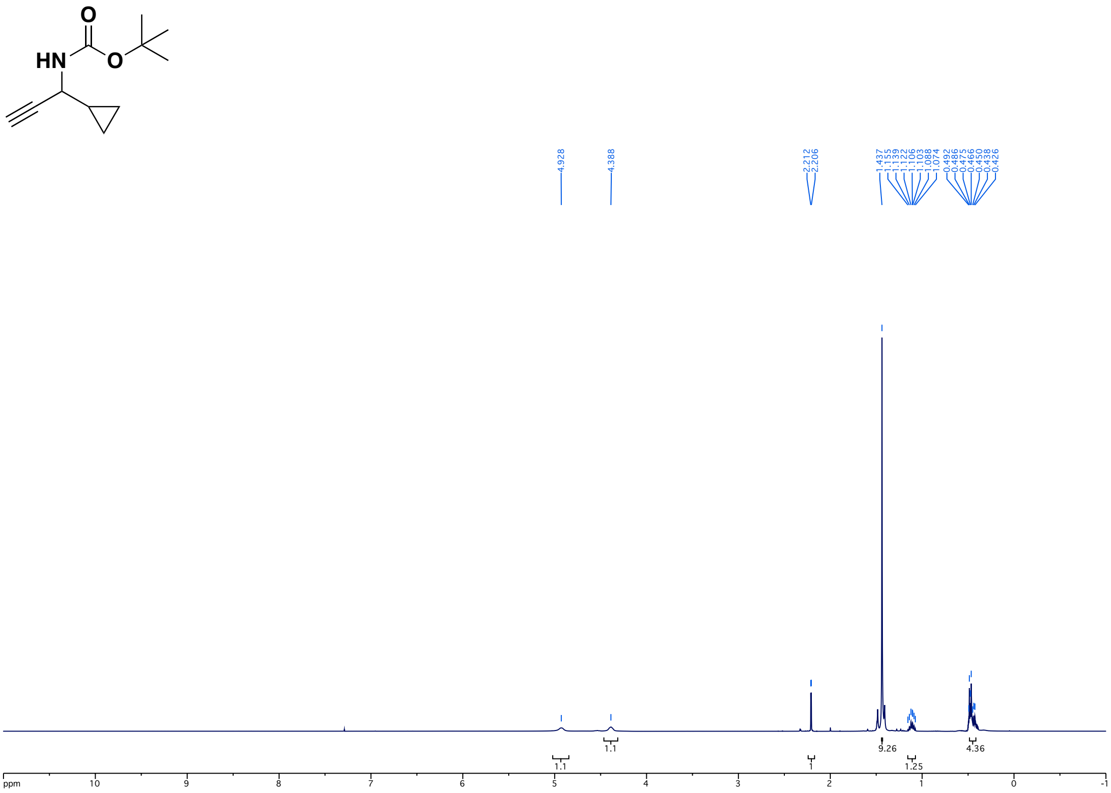

Propargylic Amine 2n: ^{13}C NMR (101 MHz, $CDCl_3$)

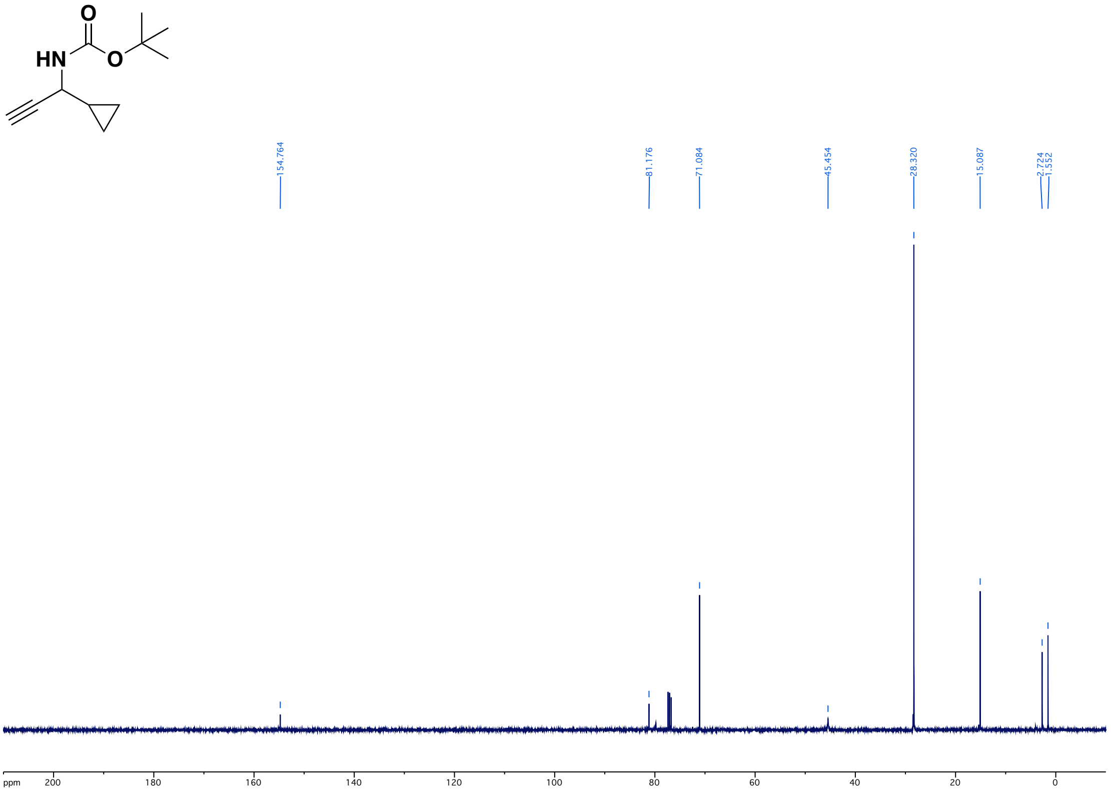

Propargylic Amine 2o: ^{1}H NMR (400 MHz, $CDCl_3$)

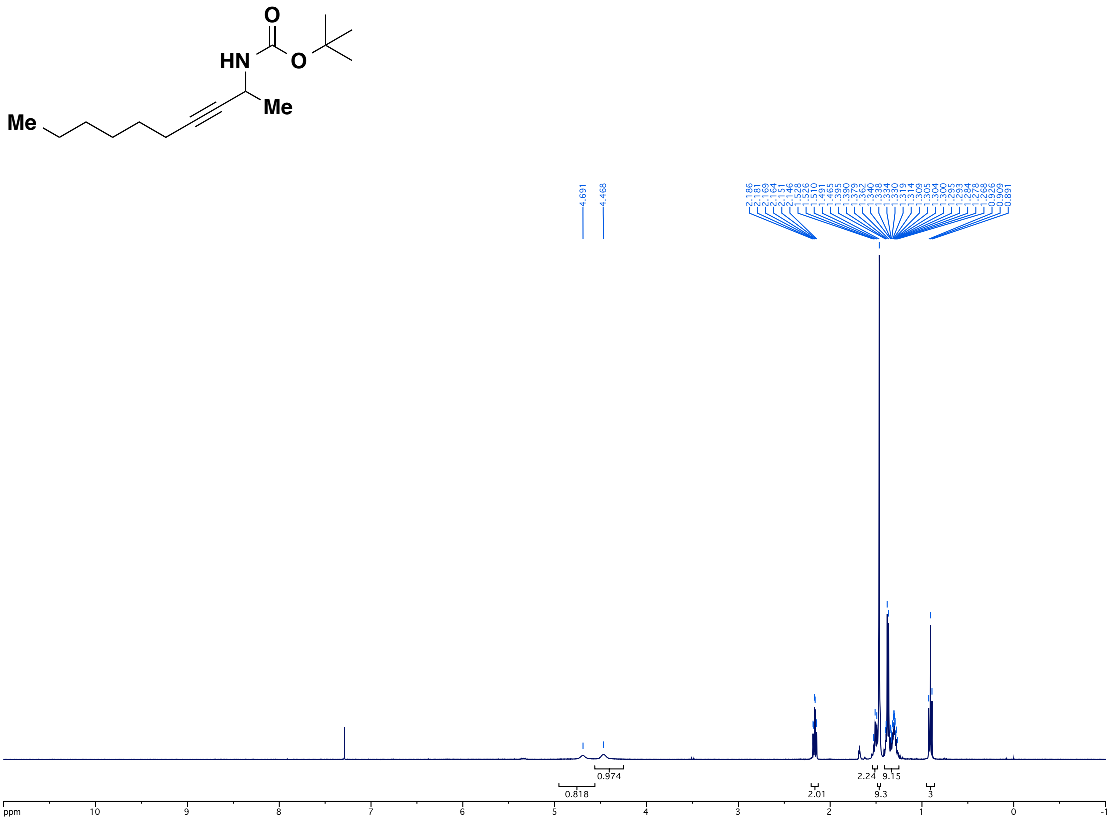

Propargylic Amine 2o: ^{13}C NMR (101 MHz, $CDCl_3$)

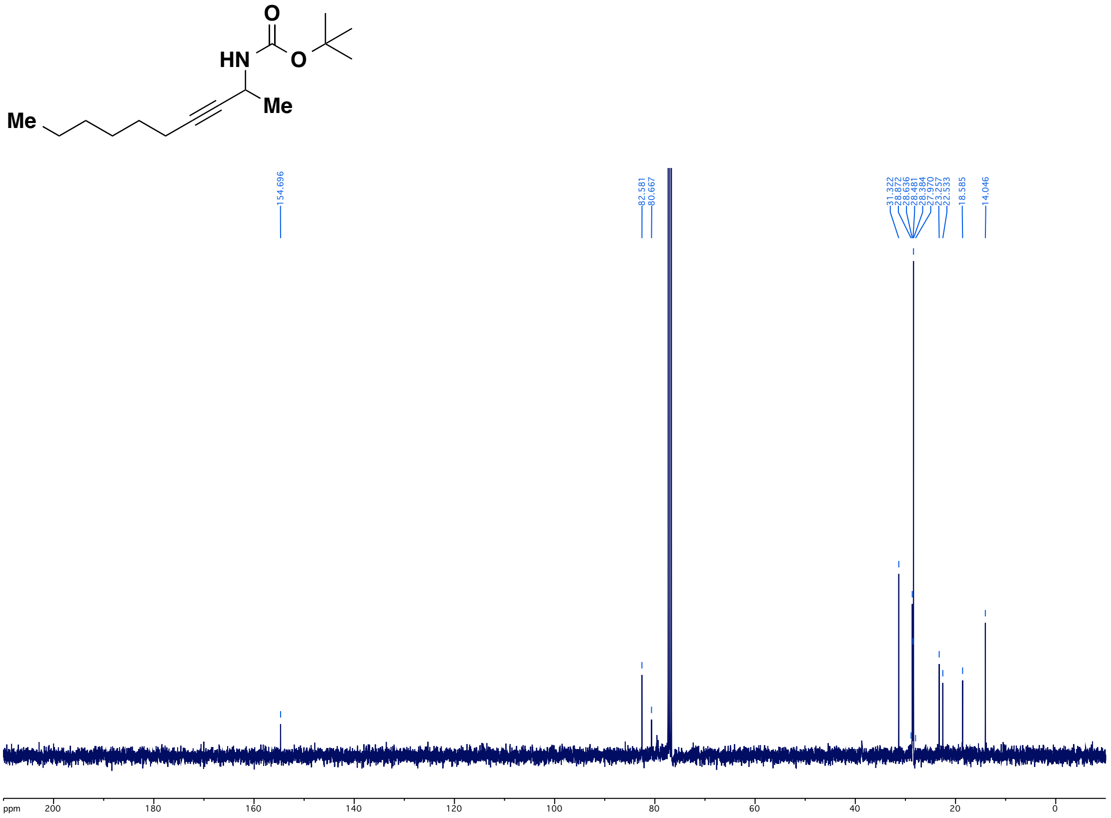

Propargylic Amine 2p: ^{1}H NMR (400 MHz, $CDCl_3$)

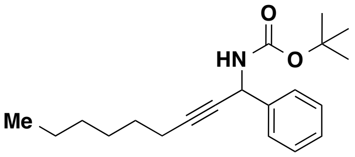

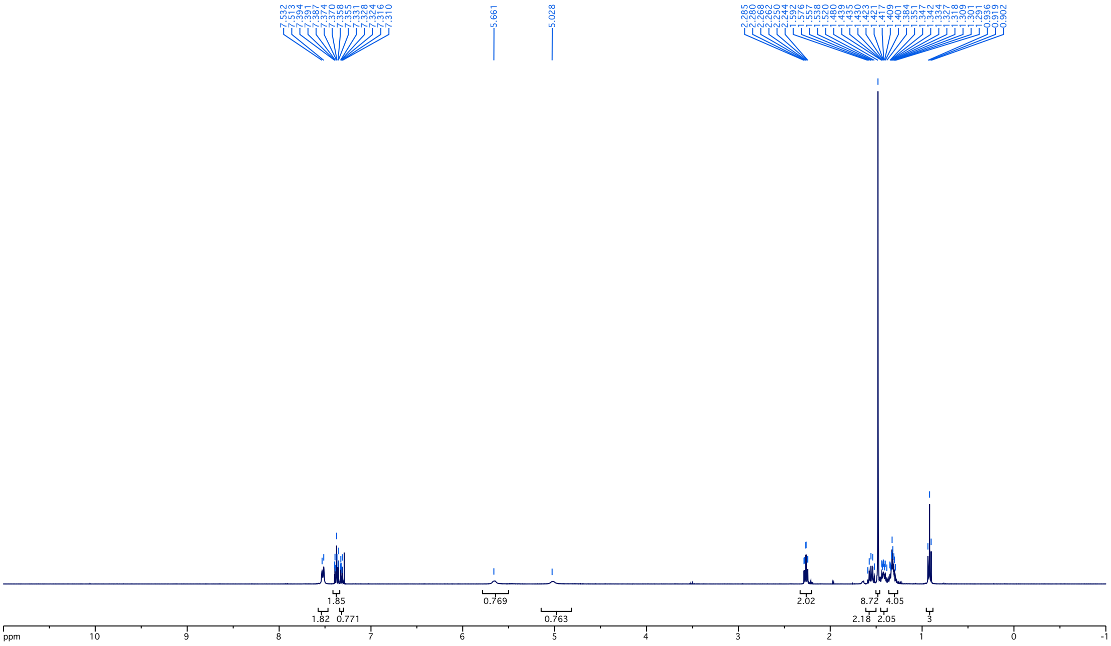

Propargylic Amine 2p: ^{13}C NMR (101 MHz, $CDCl_3$)

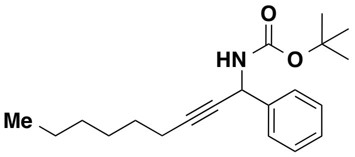

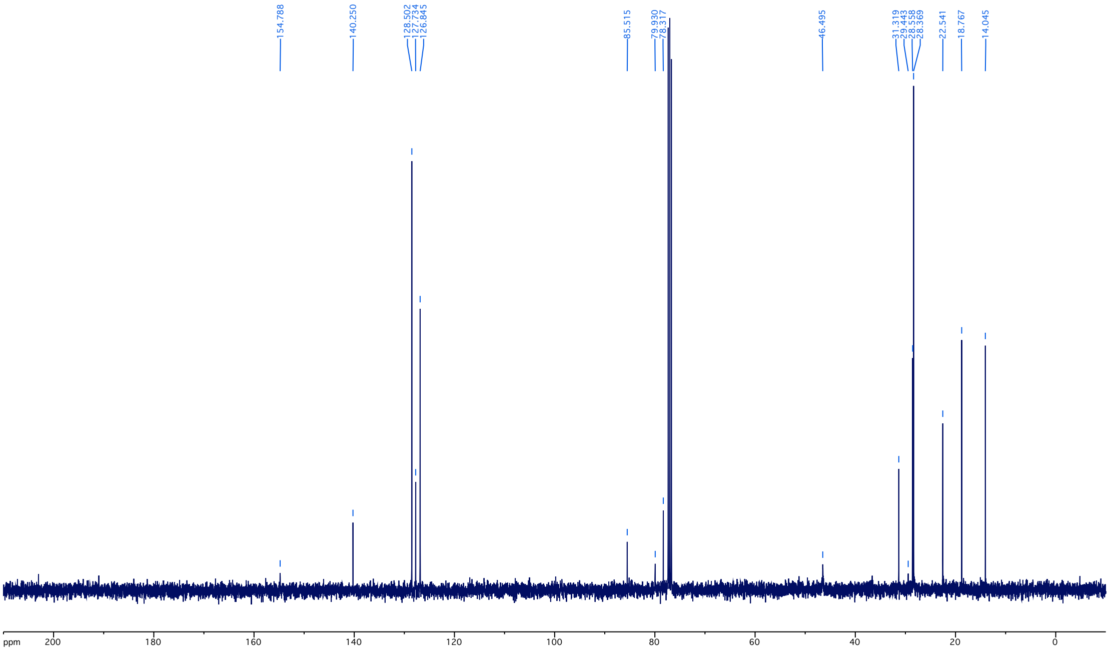

Propargylic Amine 2q: ^{1}H NMR (400 MHz, $CDCl_3$)

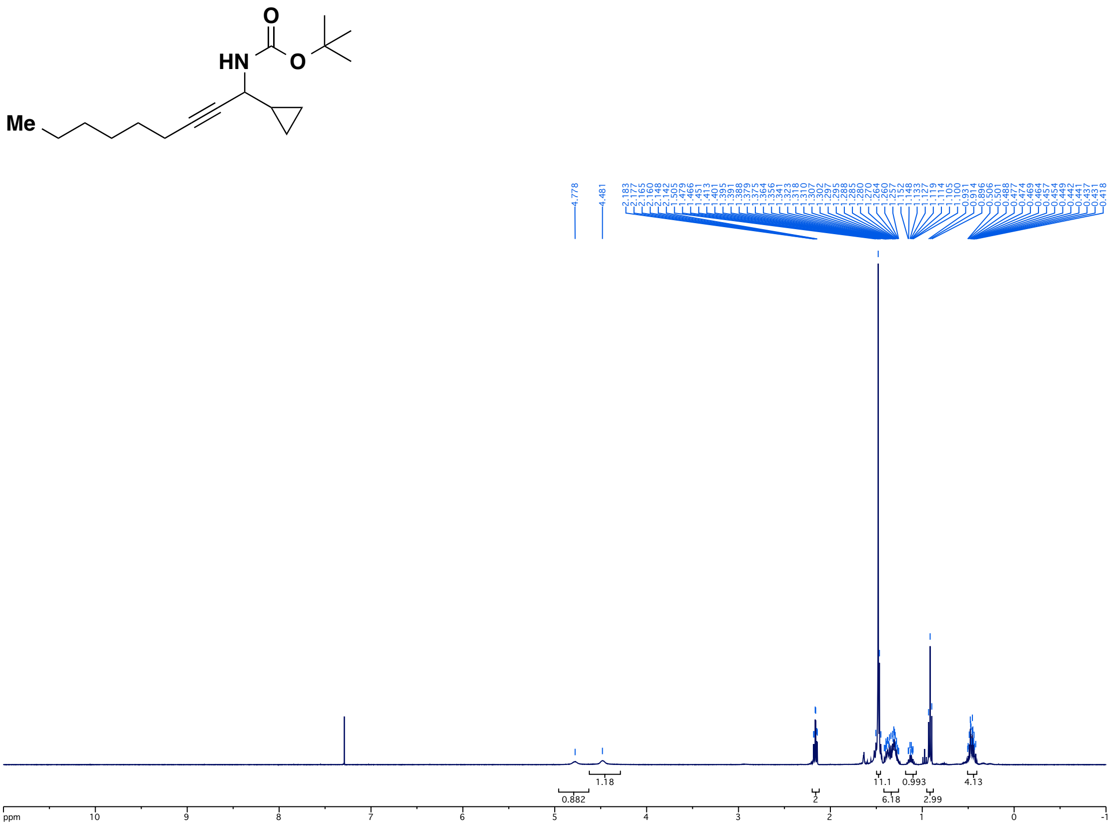

Propargylic Amine 2q: ^{13}C NMR (101 MHz, $CDCl_3$)

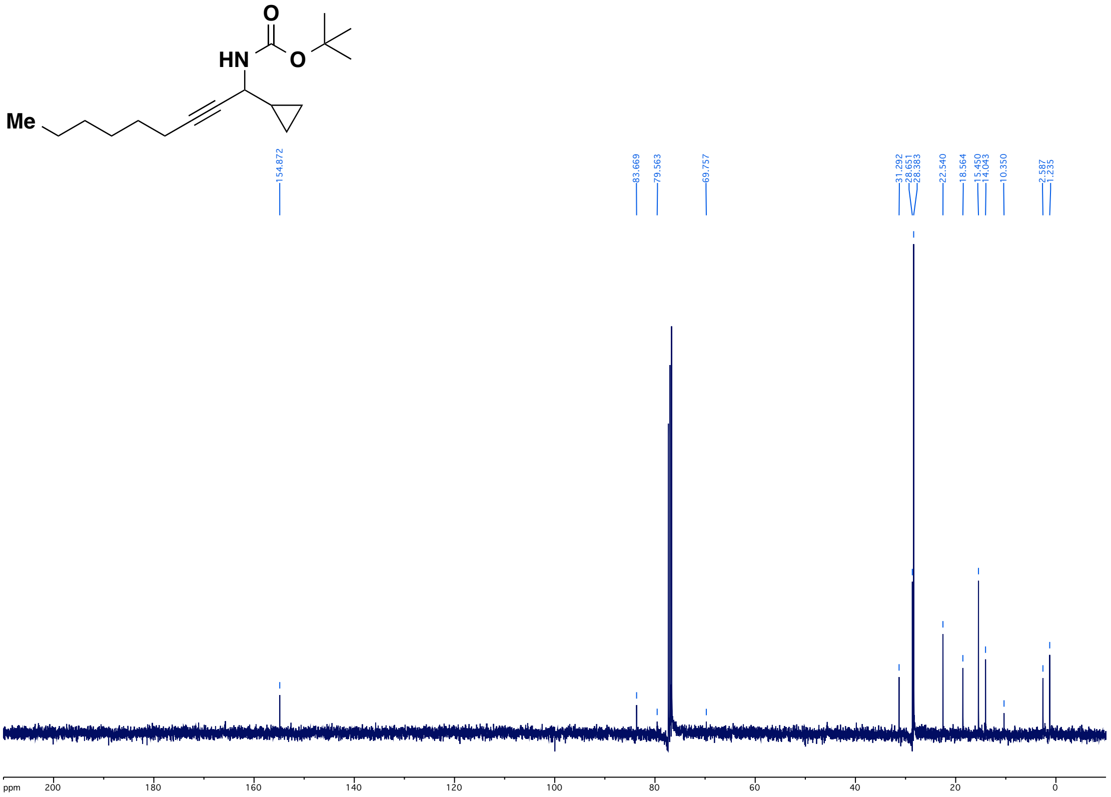

Propargylic Amine 2r: ^{1}H NMR (400 MHz, $CDCl_3$)

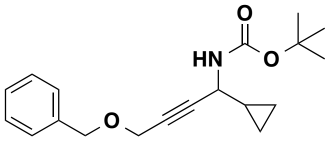

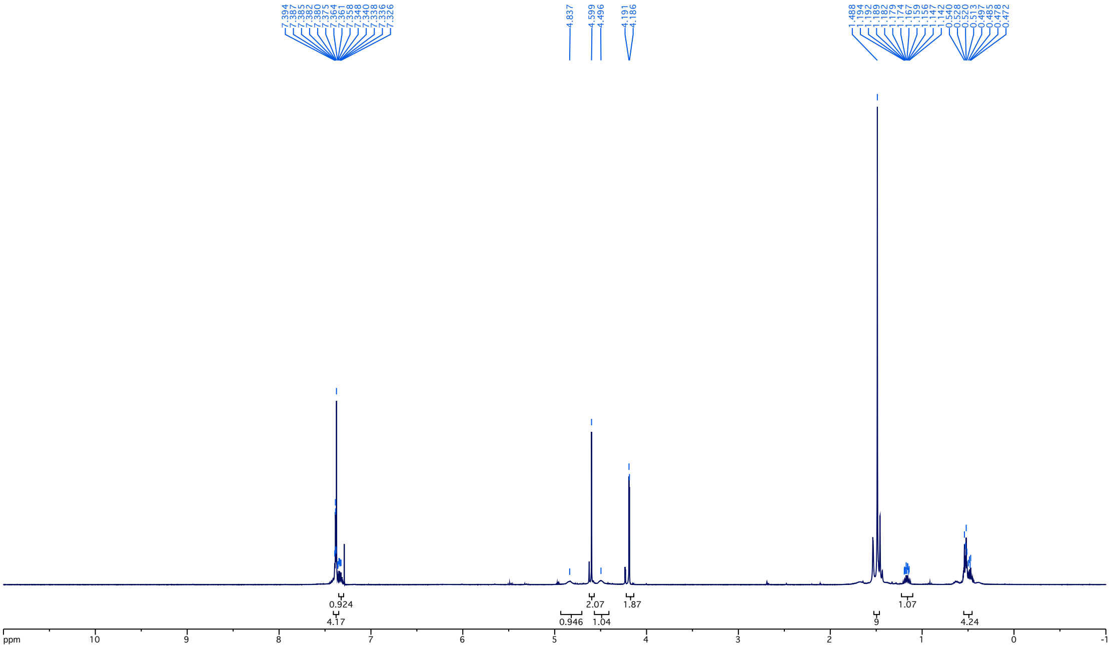

Propargylic Amine 2r: ^{13}C NMR (101 MHz, $CDCl_3$)

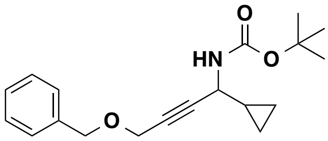

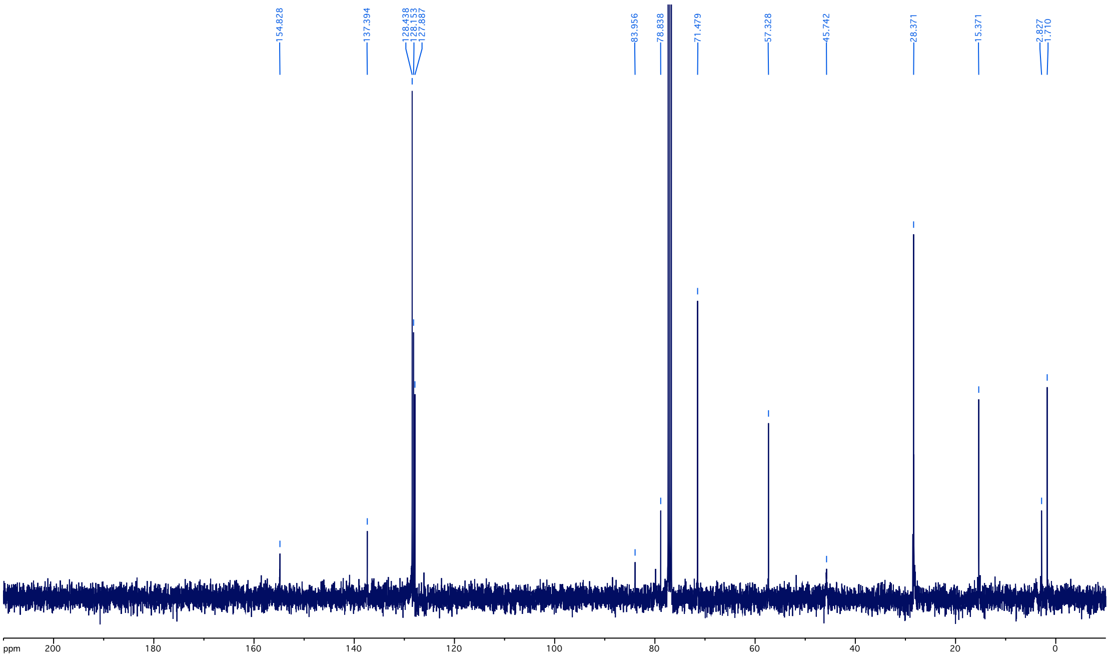

Allylic Amine 10b: ^{1}H NMR (400 MHz, $CDCl_3$)

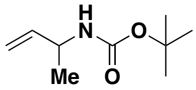

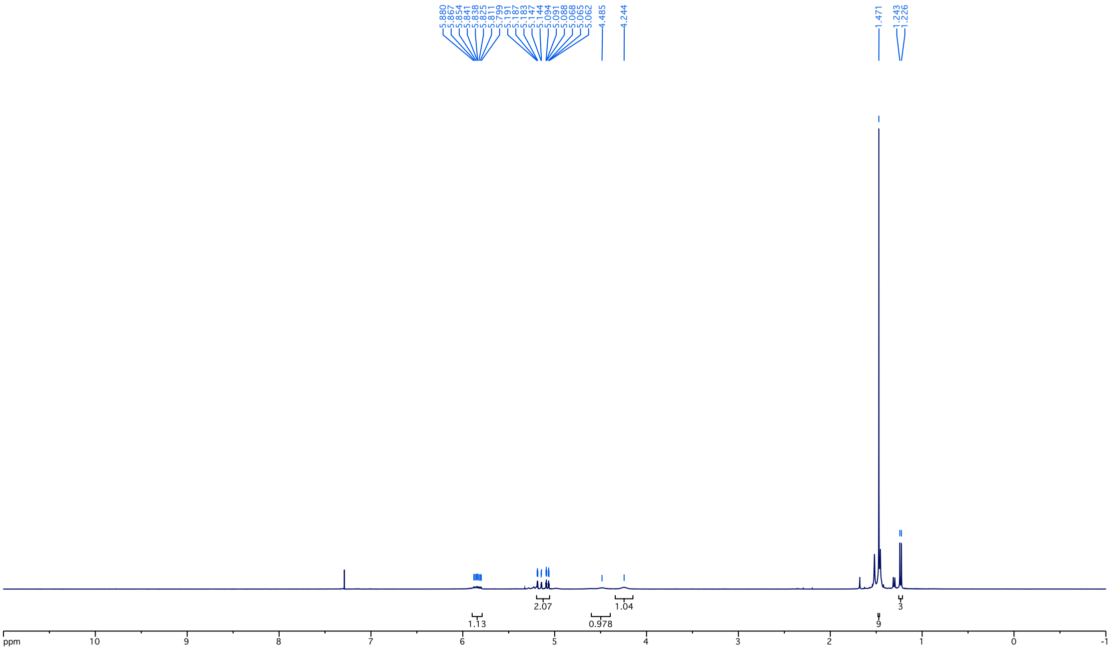

Allylic Amine 10b: ^{13}C NMR (101 MHz, $CDCl_3$)

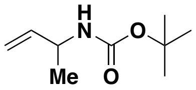

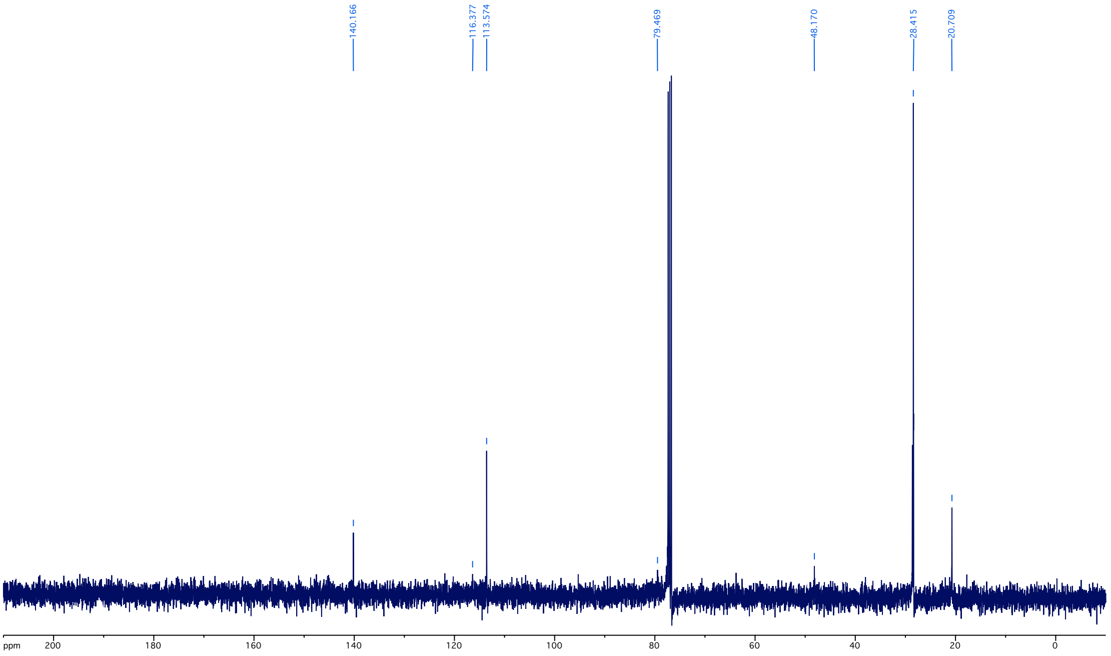

Allylic Amine 10c: ^{1}H NMR (400 MHz, $CDCl_3$)

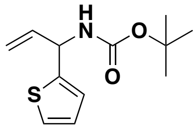

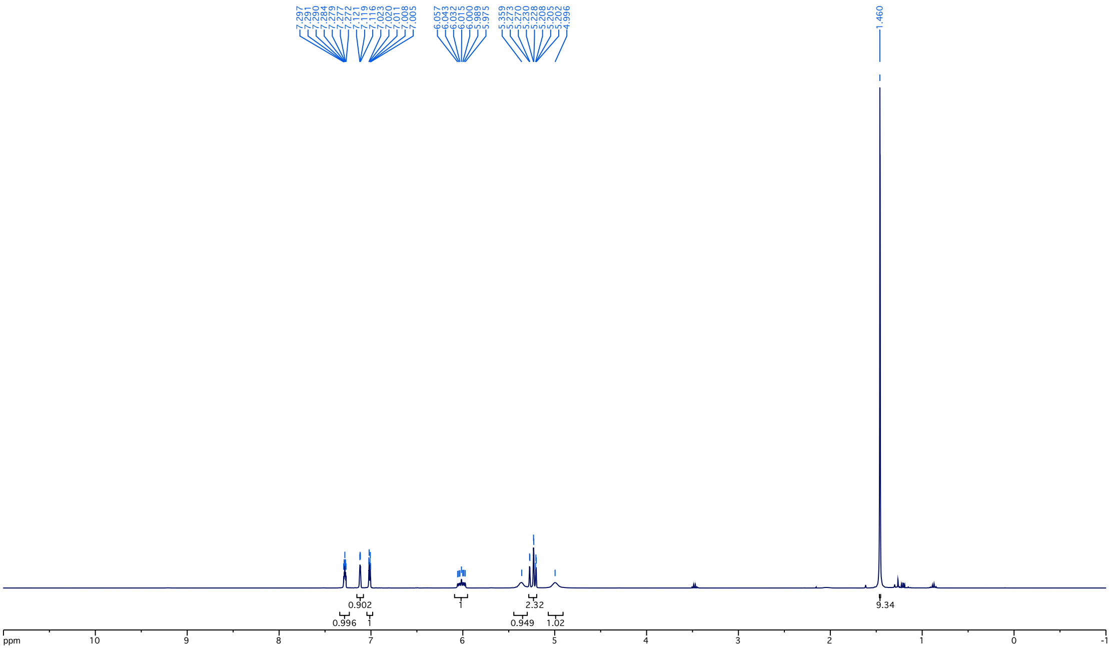

Allylic Amine 10c: ^{13}C NMR (101 MHz, $CDCl_3$)

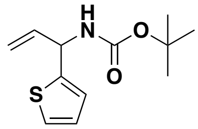

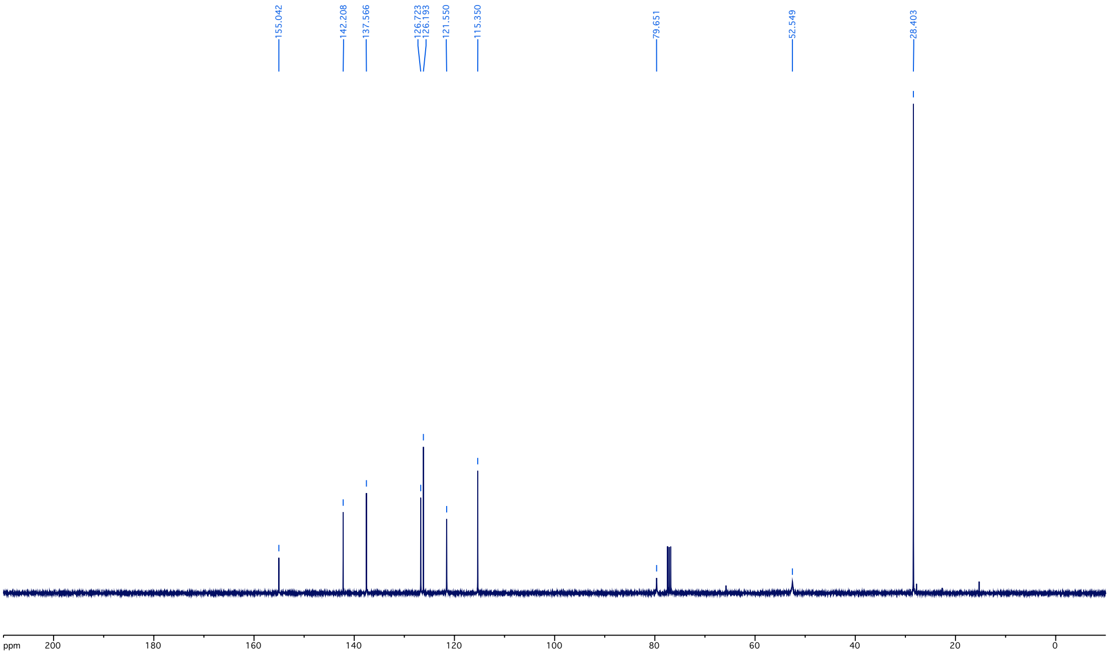

Allylic Amine 10d: ^{1}H NMR (400 MHz, $CDCl_3$)

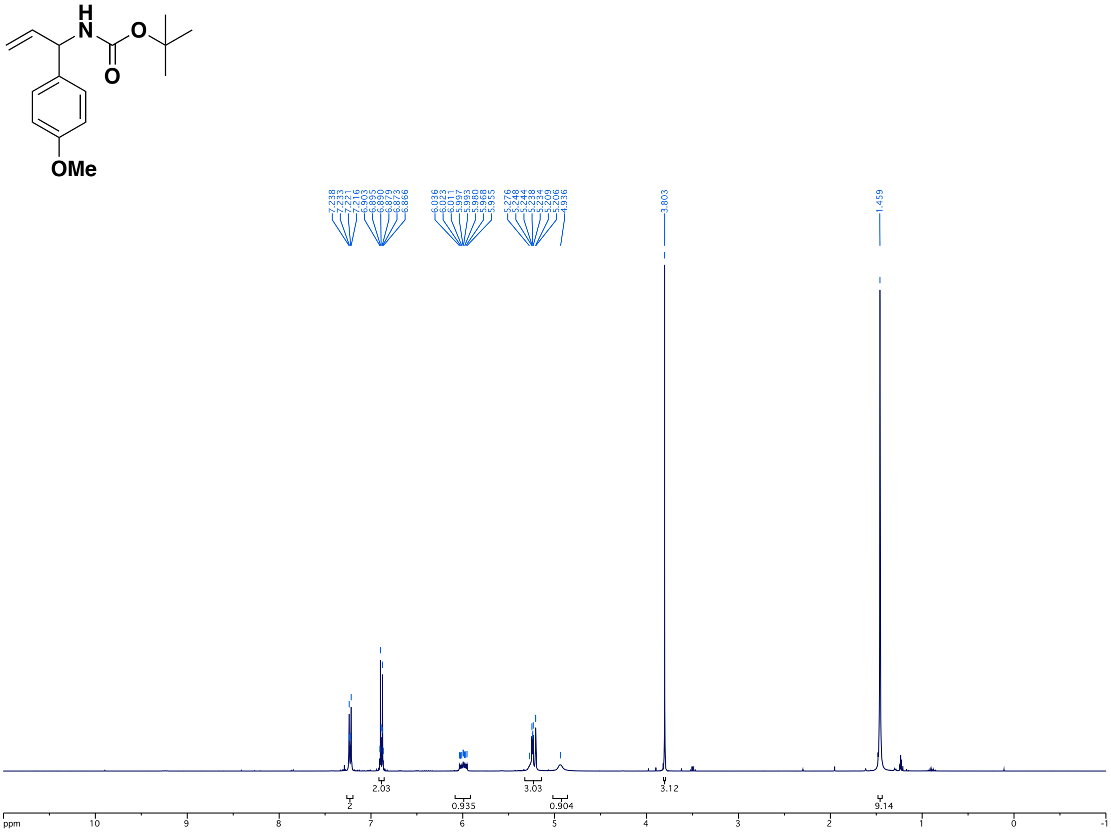

Allylic Amine 10d: ^{13}C NMR (101 MHz, $CDCl_3$)

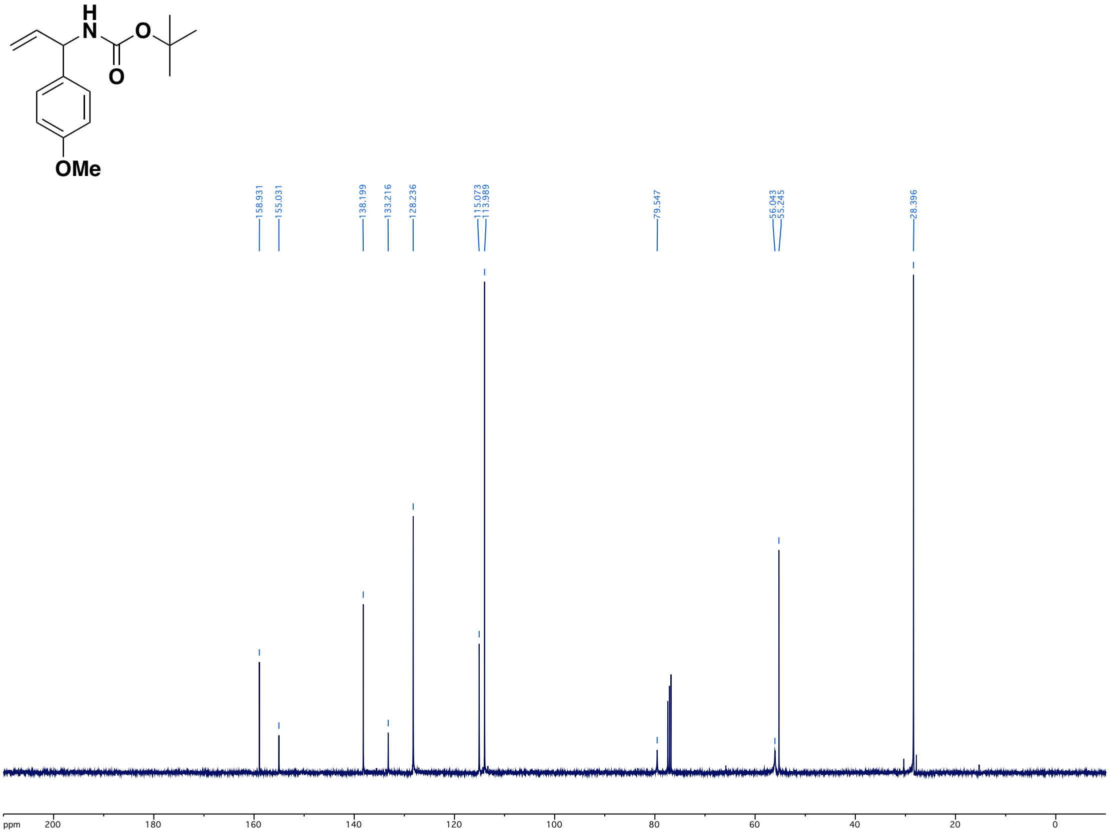

Allylic Amine 10e: ^{1}H NMR (400 MHz, $CDCl_3$)

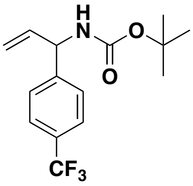

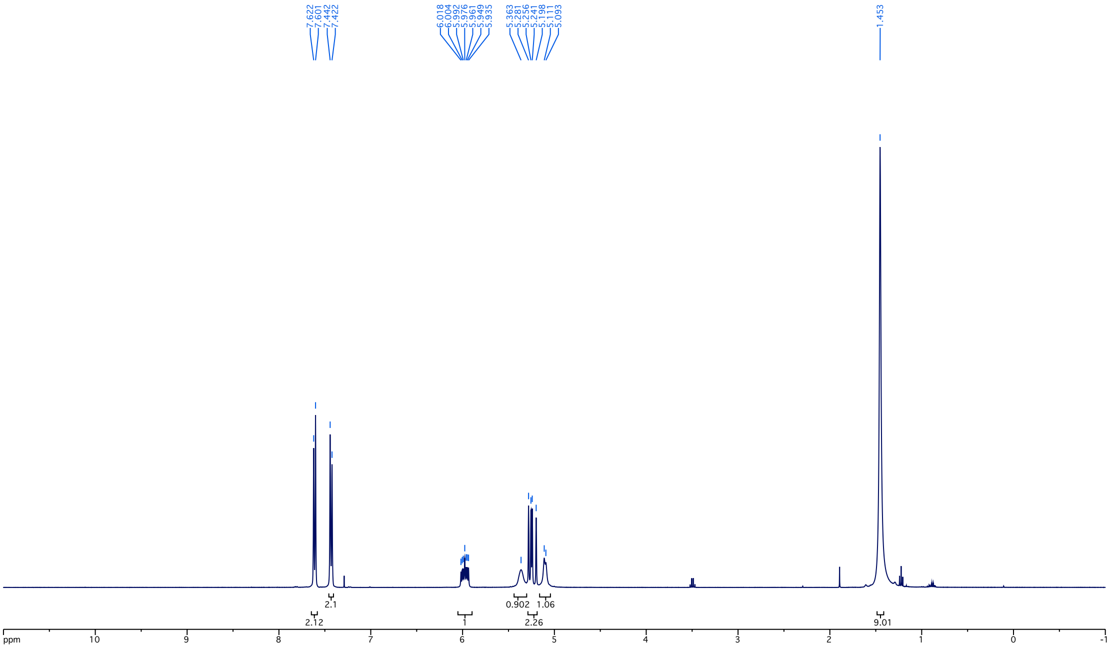

Allylic Amine 10e: ^{13}C NMR (101 MHz, $CDCl_3$)

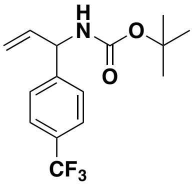

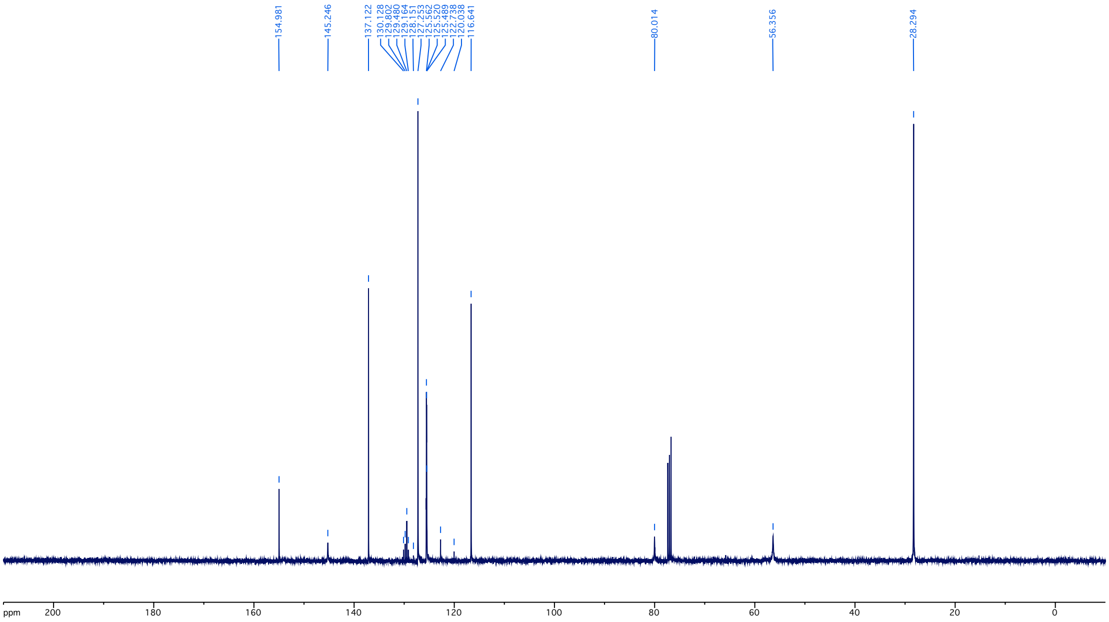

Allylic Amine 10e: ^{19}F NMR (377 MHz, $CDCl_3$)

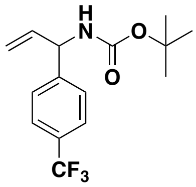

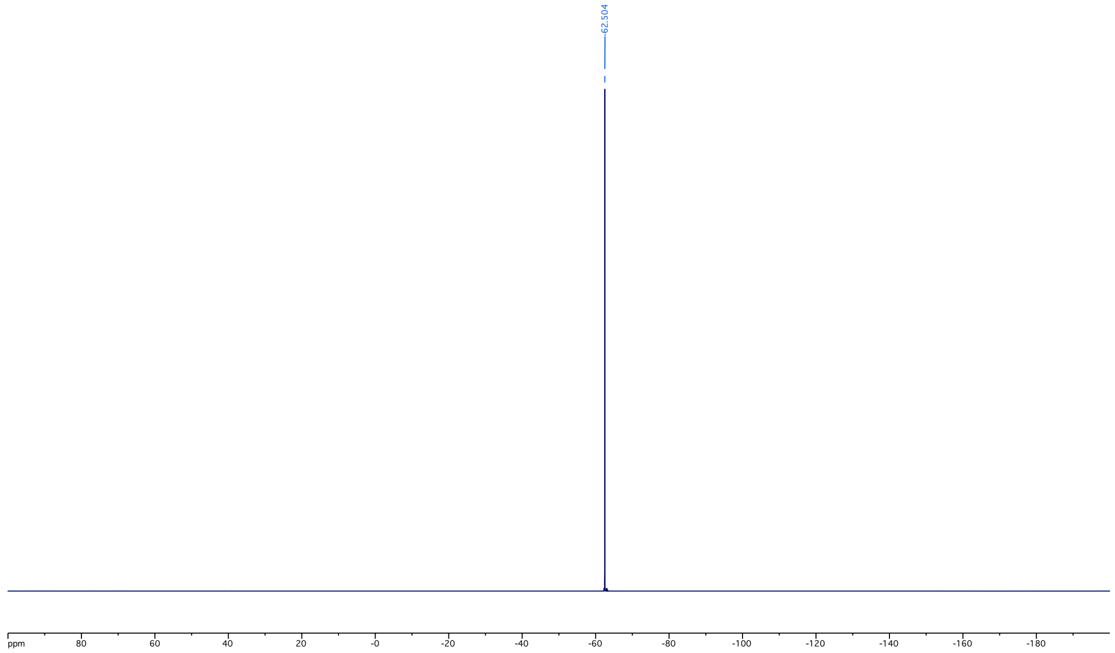

Allylic Amine 10f: ^{1}H NMR (400 MHz, $CDCl_3$)

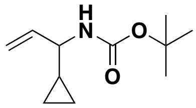

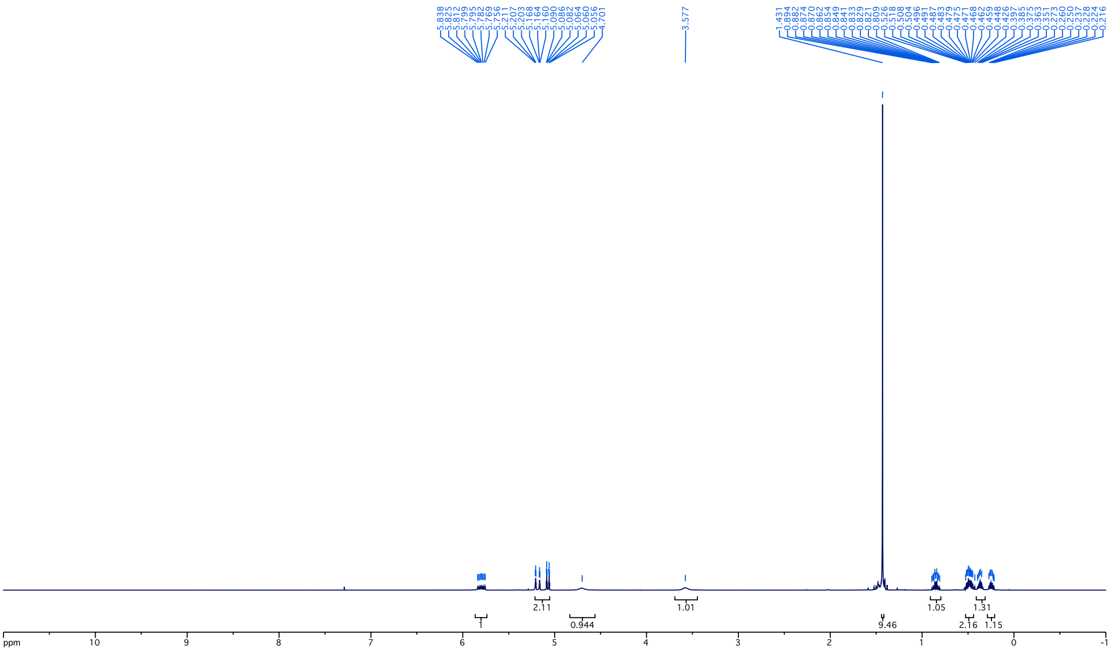

Allylic Amine 10f: ^{13}C NMR (101 MHz, $CDCl_3$)

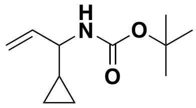

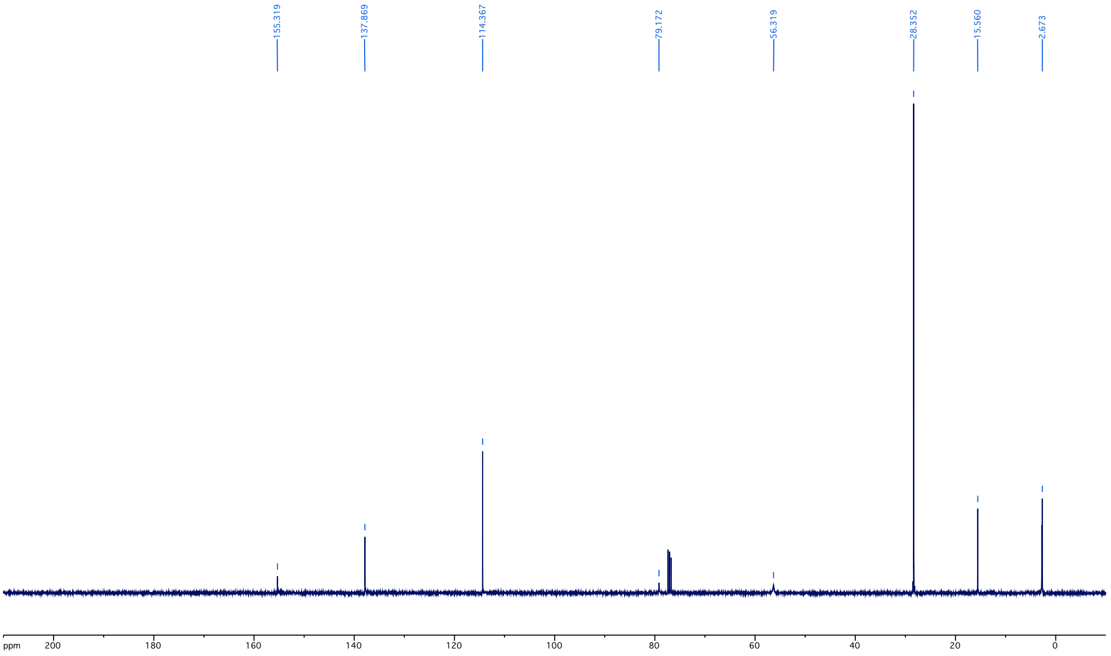

γ-Amino enone 3a: ^{1}H NMR (400 MHz, $CDCl_3$)

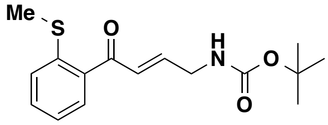

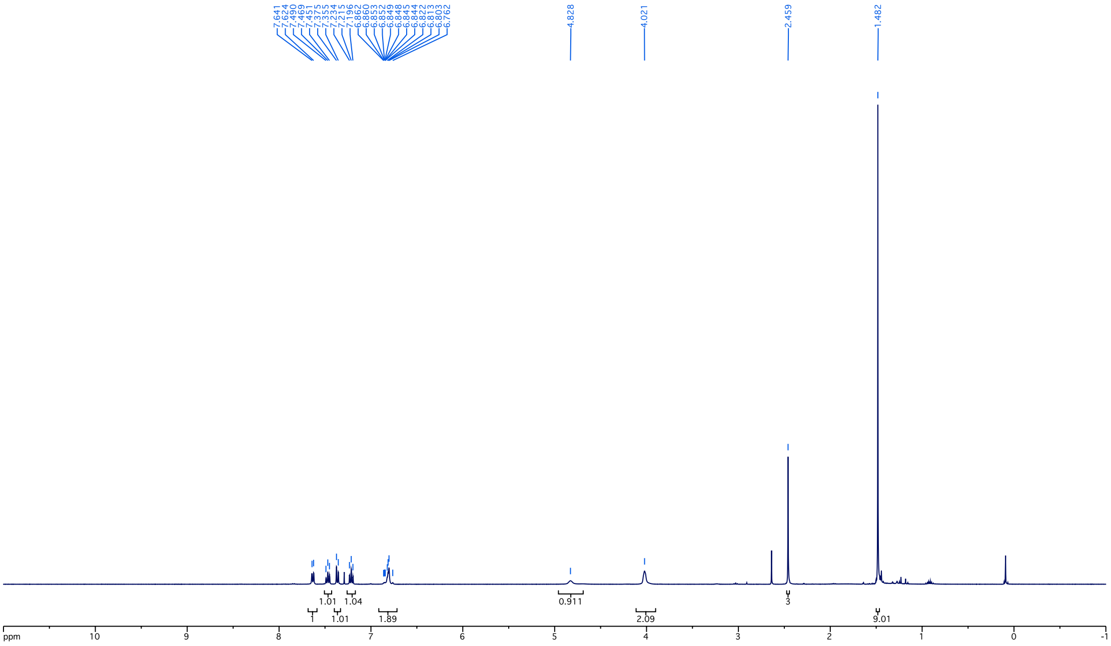

γ-Amino enone 3a: ^{13}C NMR (101 MHz, $CDCl_3$)

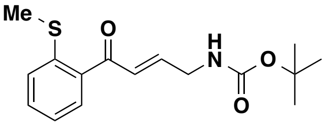

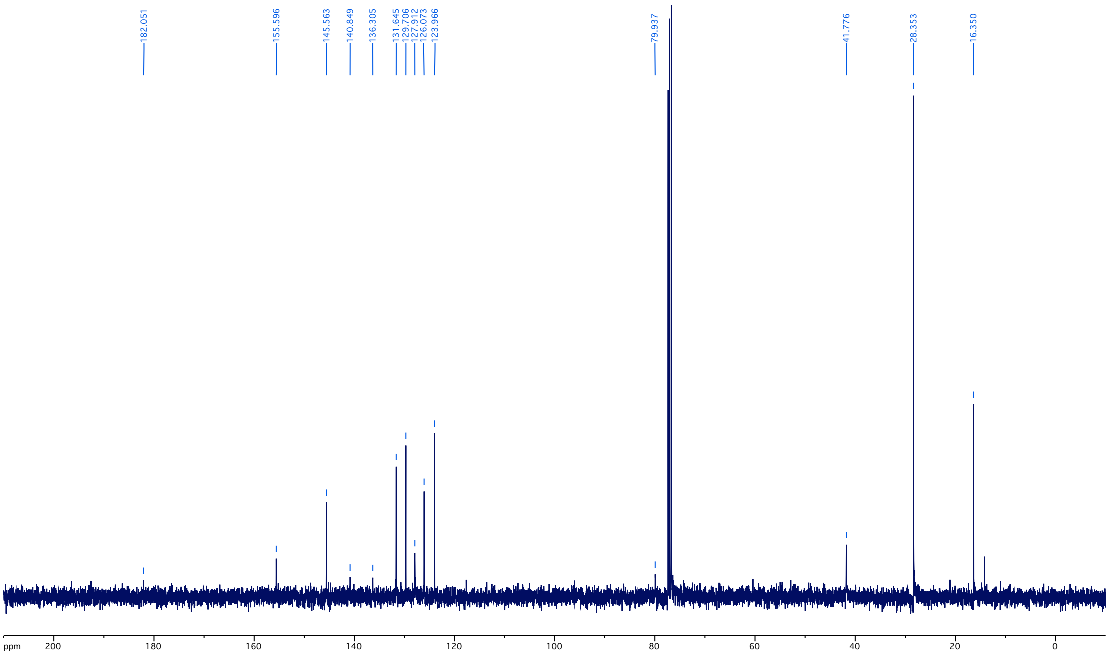

γ-Amino enone 3a’: ^{1}H NMR (400 MHz, $CDCl_3$)

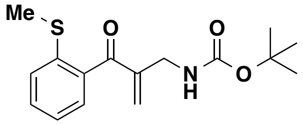

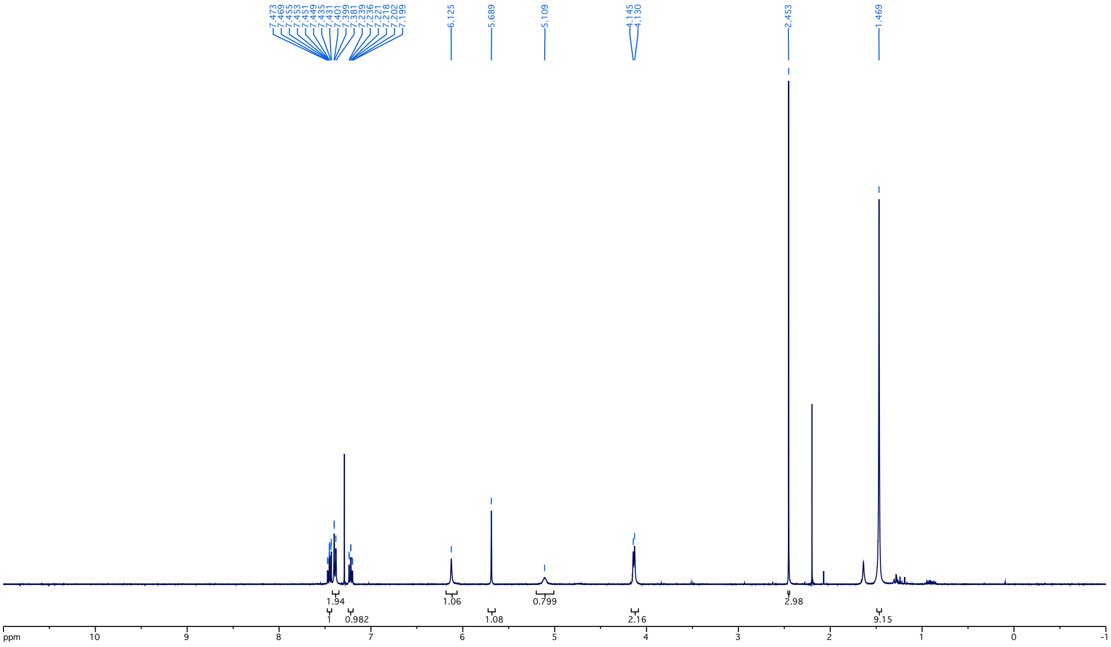

γ-Amino enone 3a’: ^{13}C NMR (101 MHz, $CDCl_3$)

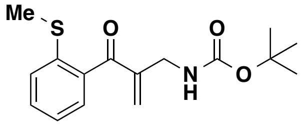

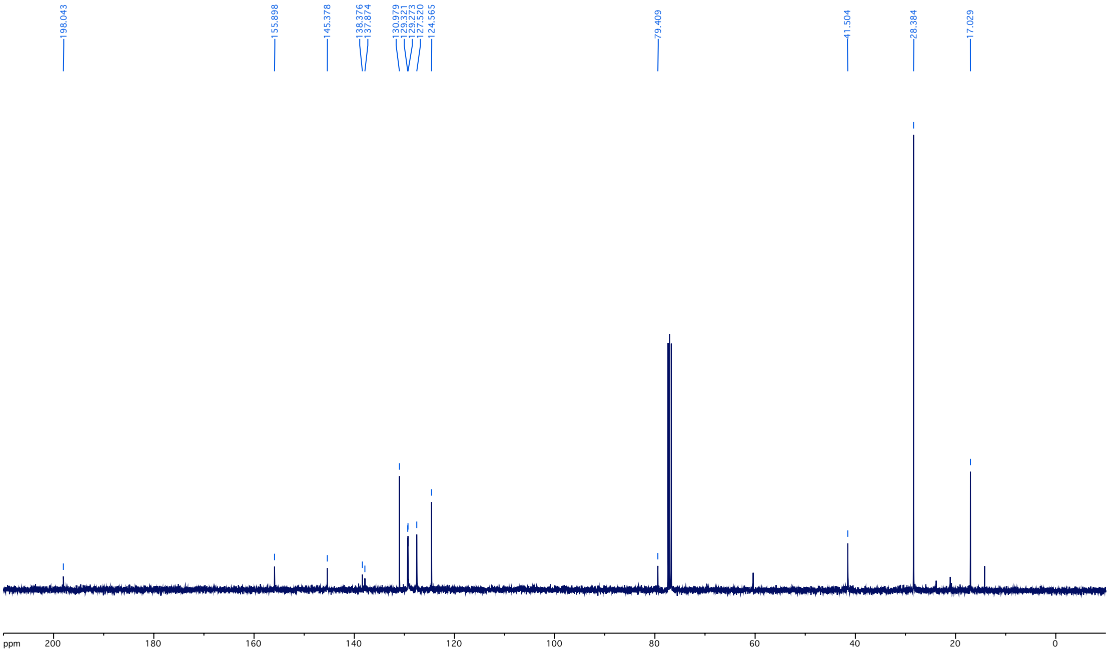

γ-Amino enone 3a-CBz: ^{1}H NMR (400 MHz, $CDCl_3$)

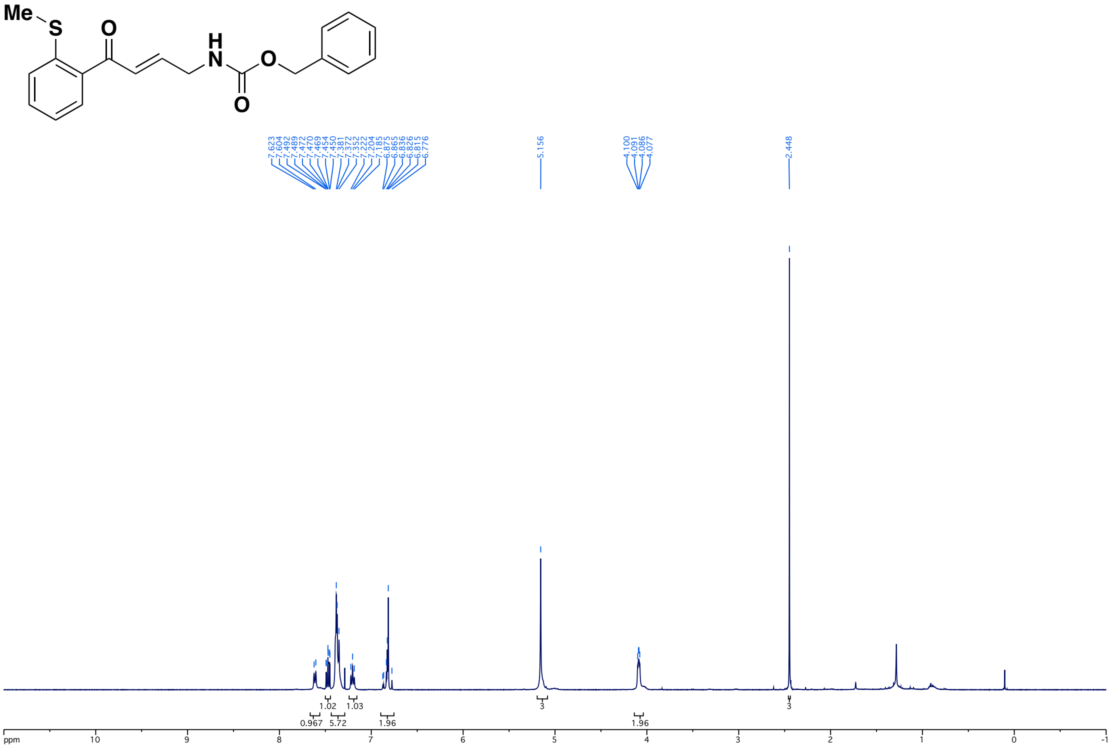

γ-Amino enone 3a-CBz: ^{13}C NMR (101 MHz, $CDCl_3$)

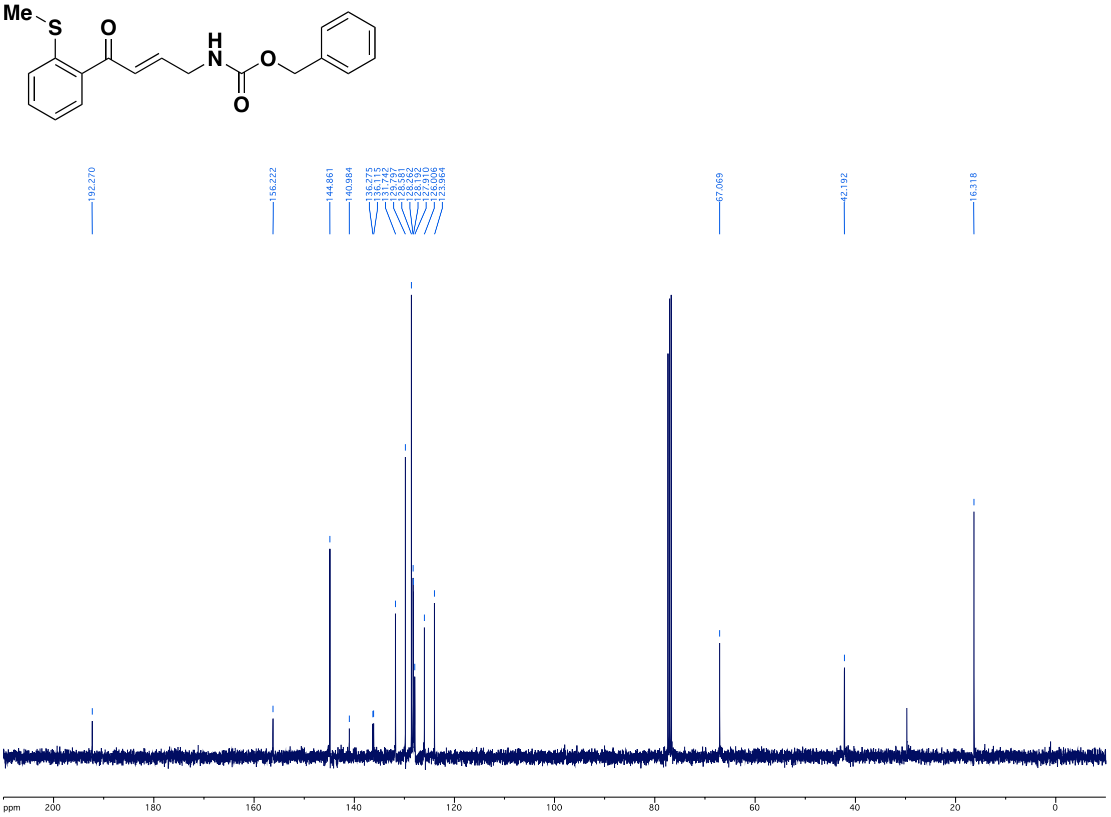

γ-Amino enone 3a'-CBz: ^{1}H NMR (400 MHz, $CDCl_3$)

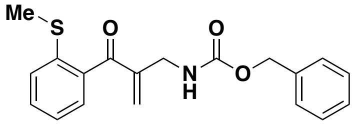

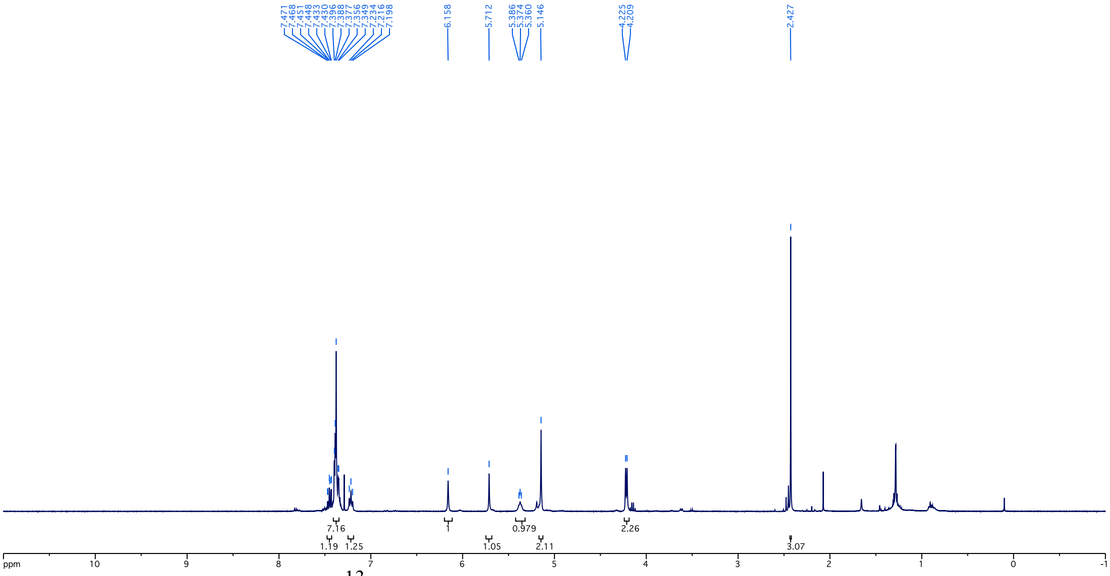

γ-Amino enone 3a'-CBz: ^{13}C NMR (101 MHz, $CDCl_3$)

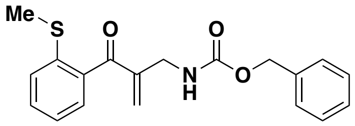

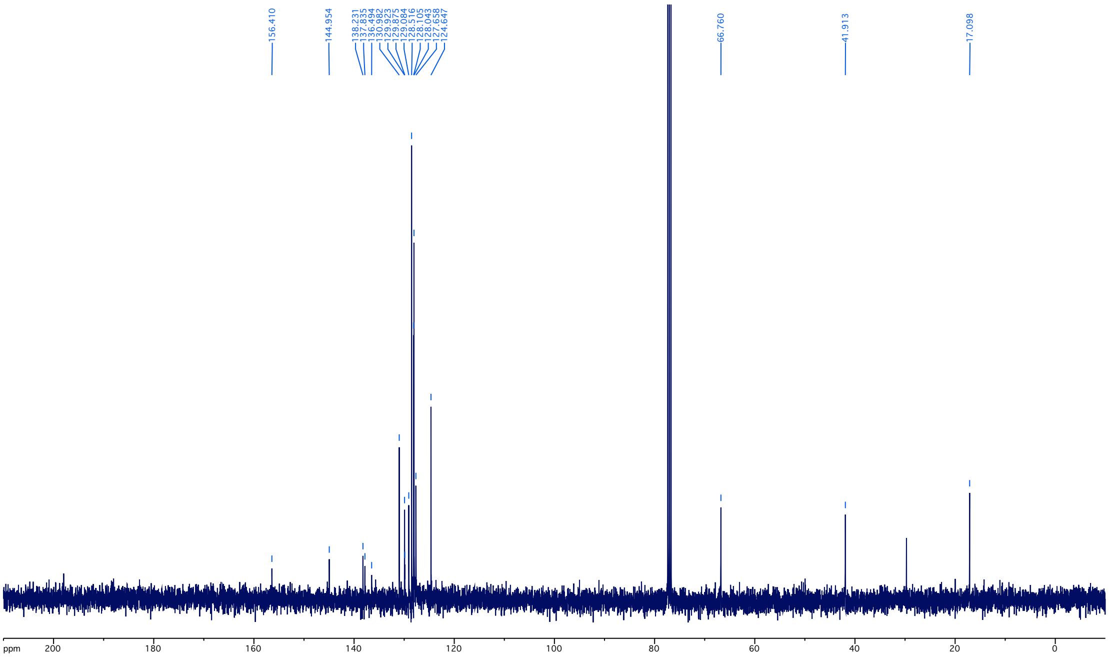

γ-Amino enone 3a-Ts: ^{1}H NMR (500 MHz, $CDCl_3$)

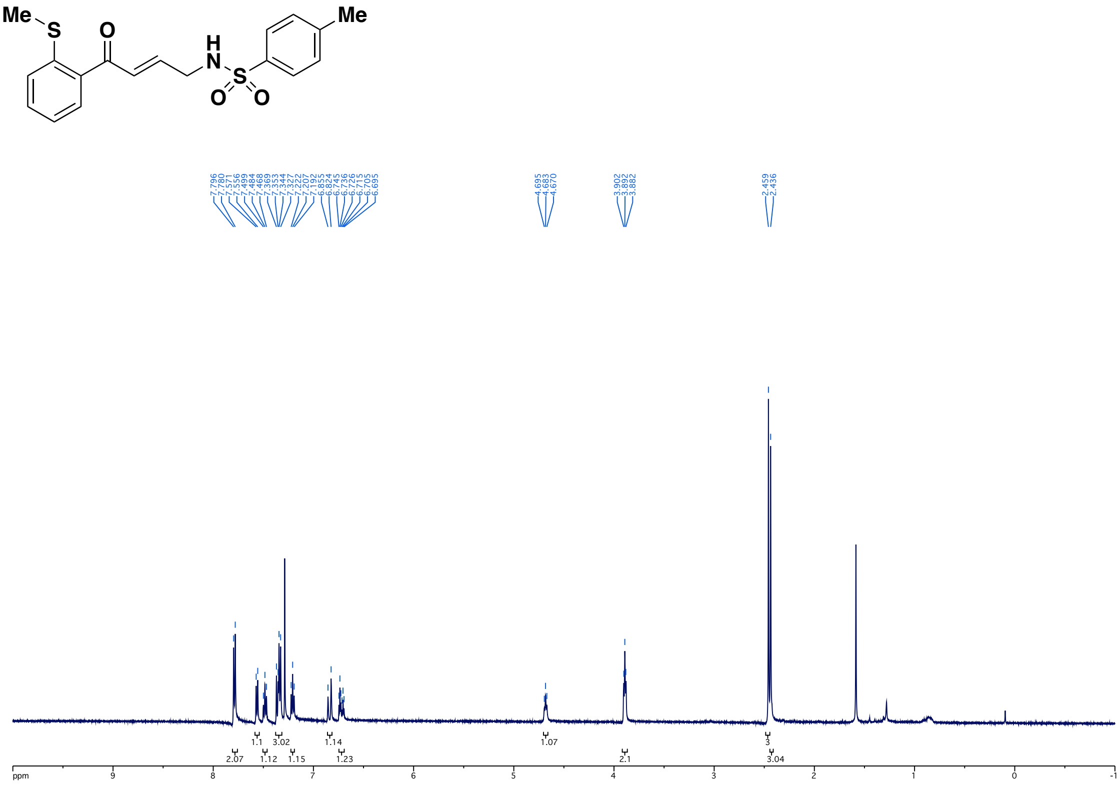

γ-Amino enone 3a-Ts: ^{13}C NMR (125 MHz, $CDCl_3$)

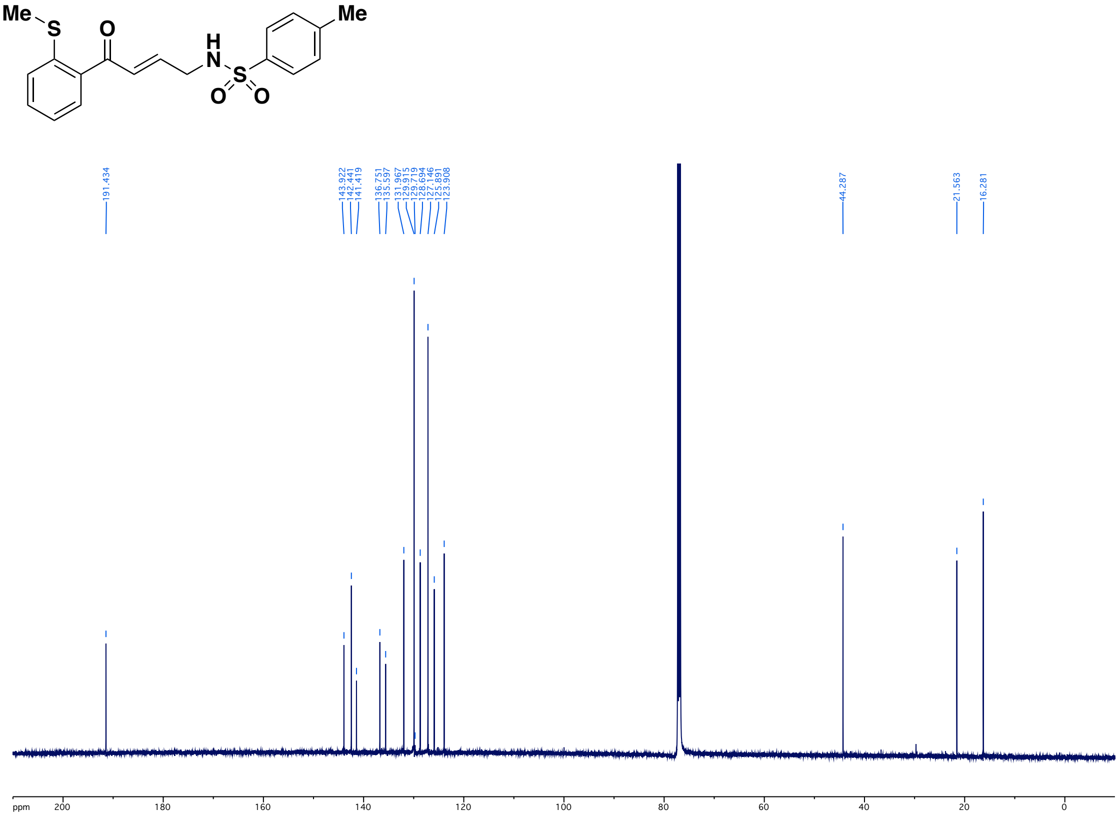

γ-Amino enone 3a'-Ts: ^{1}H NMR (400 MHz, $CDCl_3$)

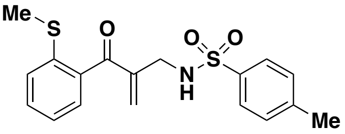

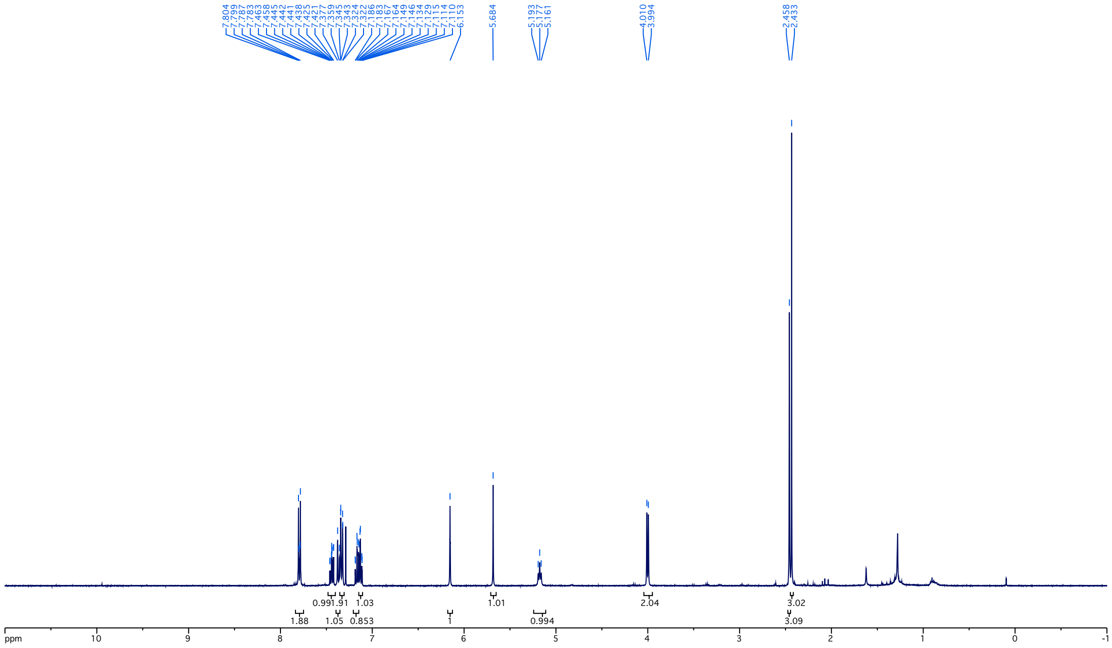

γ-Amino enone 3a'-Ts: ^{13}C NMR (101 MHz, $CDCl_3$)

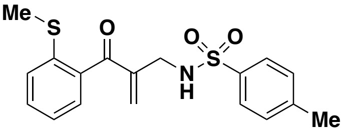

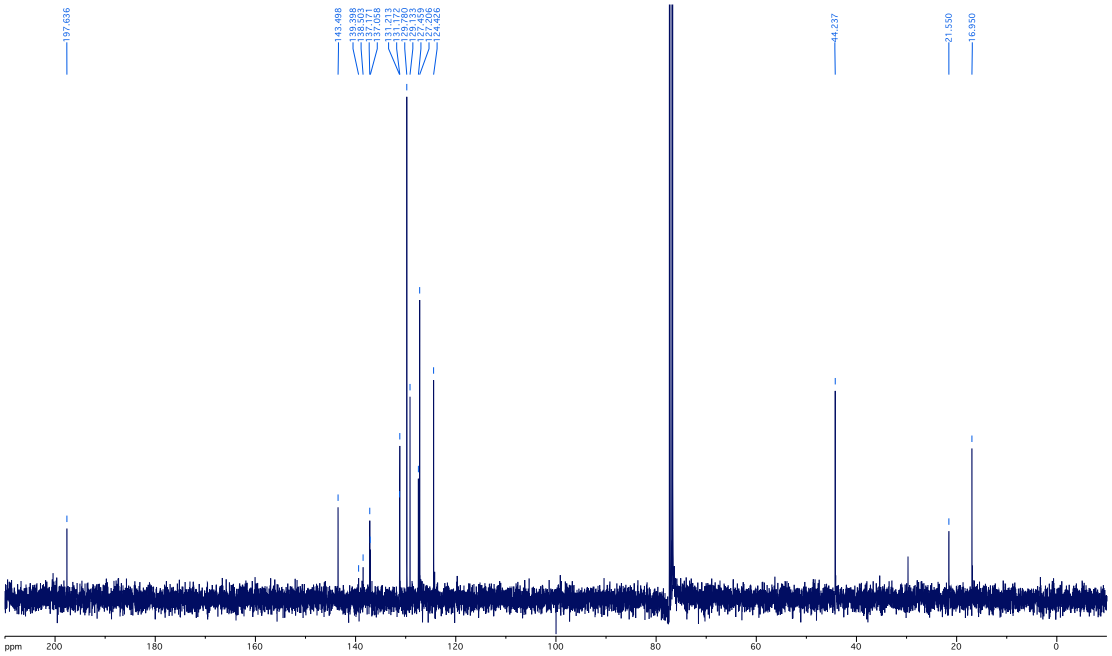

γ-Amino enone 3b: ^{1}H NMR (400 MHz, $CDCl_3$)

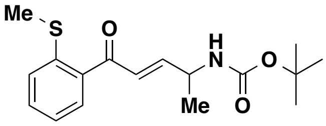

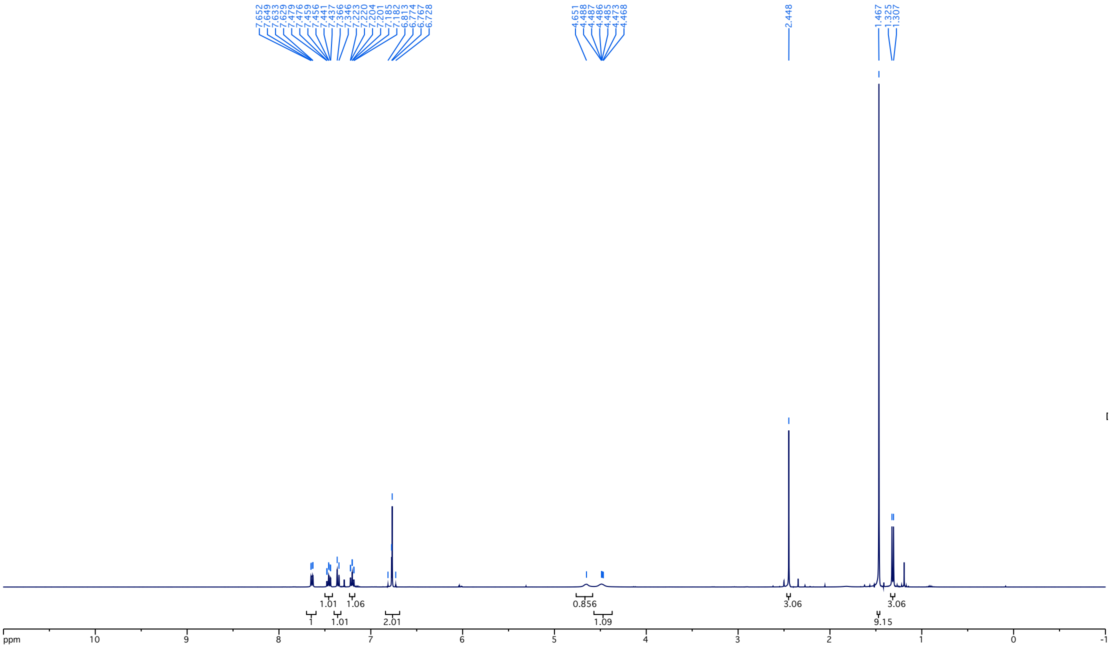

γ-Amino enone 3b: ^{13}C NMR (101 MHz, $CDCl_3$)

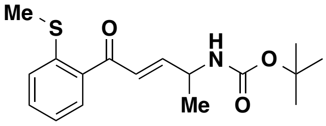

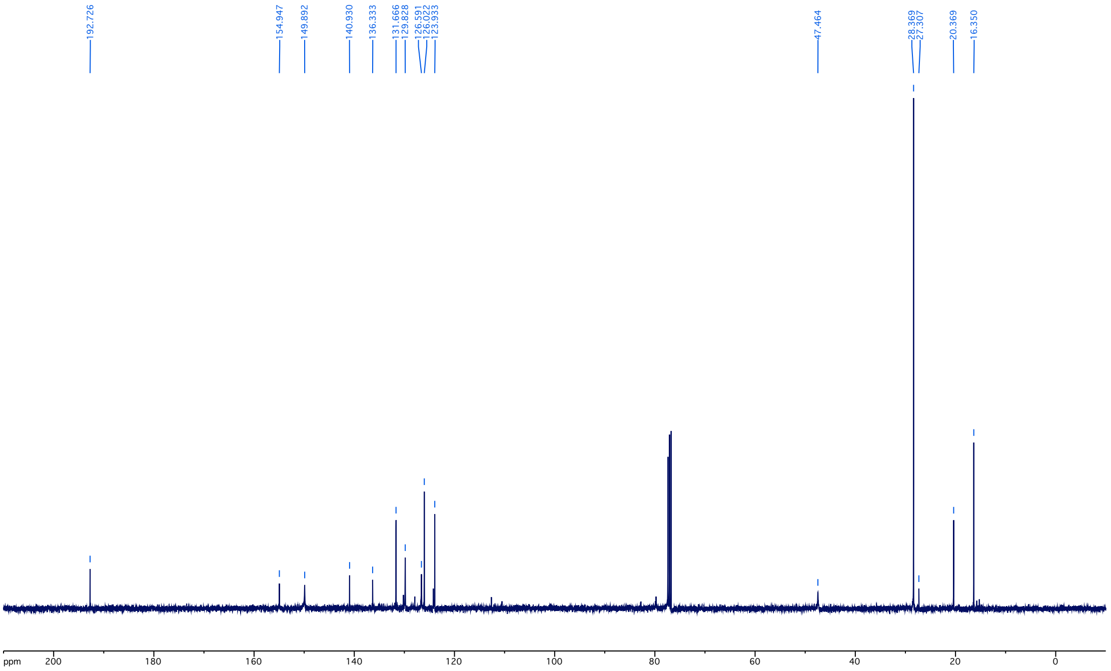

γ-Amino enone 3c: ^{1}H NMR (400 MHz, $CDCl_3$)

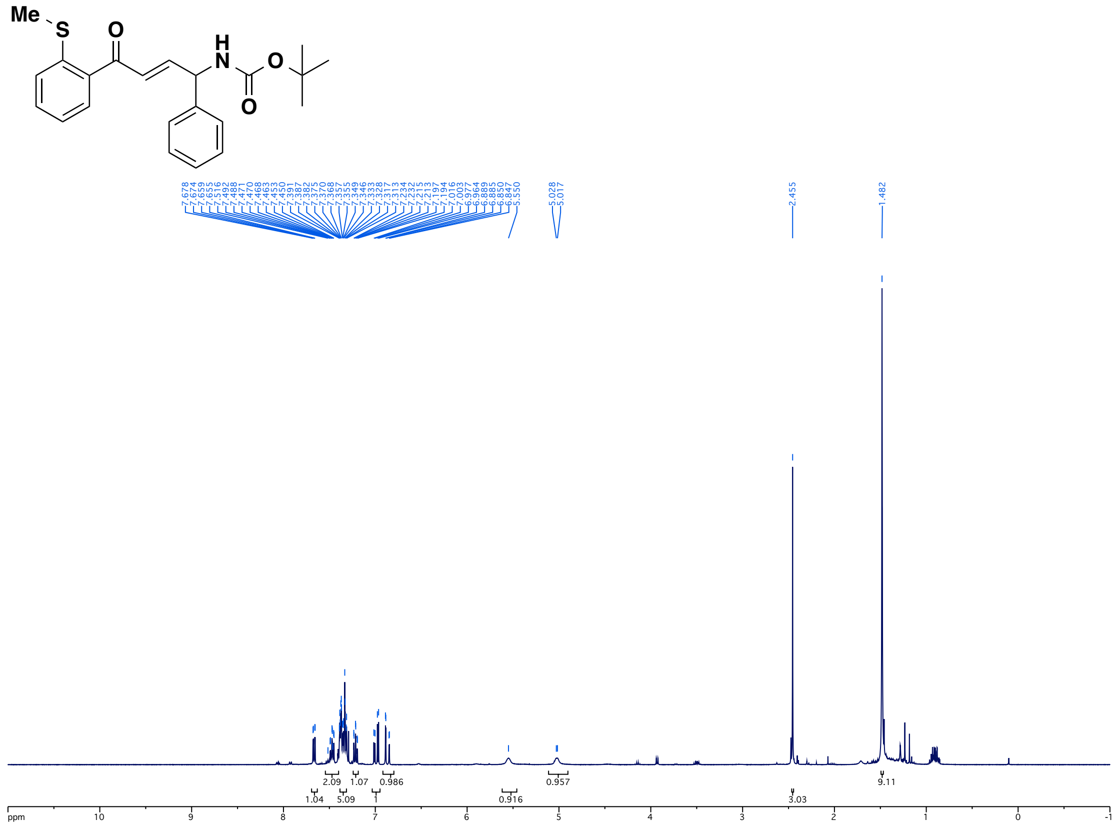

γ-Amino enone 3c: ^{13}C NMR (101 MHz, $CDCl_3$)

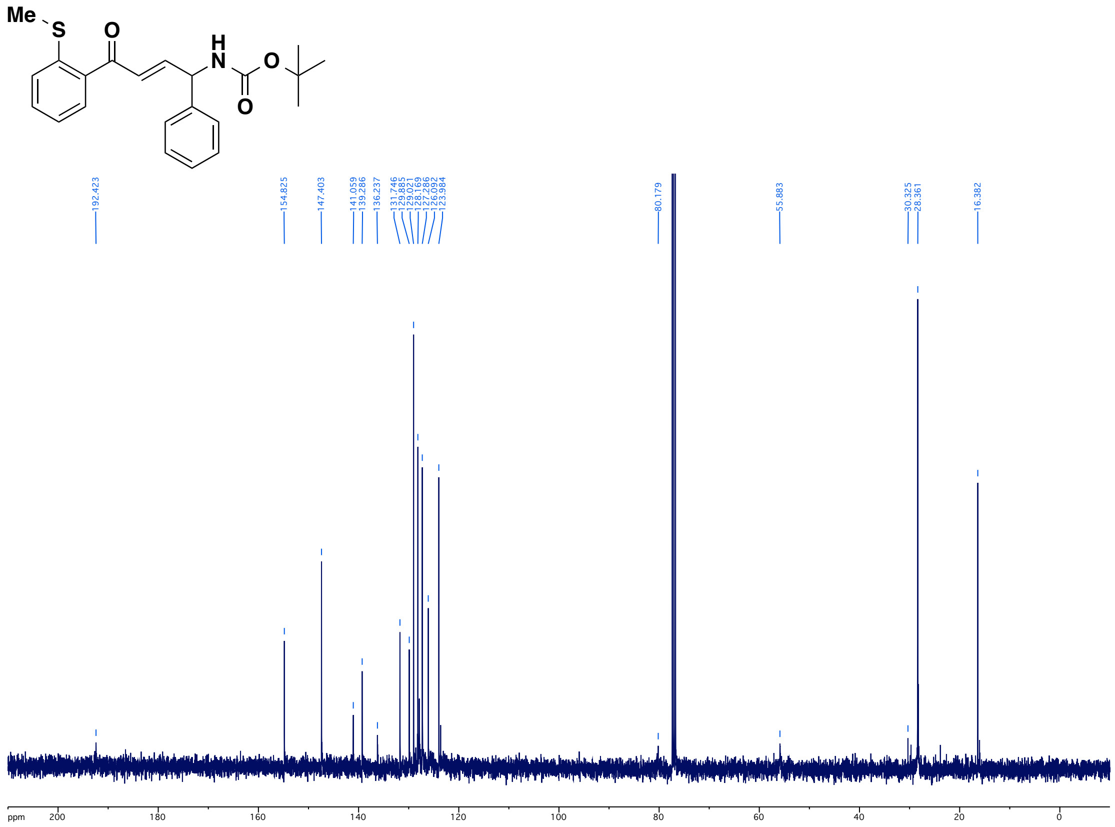

γ-Amino enone 3d: ^{1}H NMR (400 MHz, $CDCl_3$)

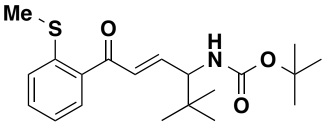

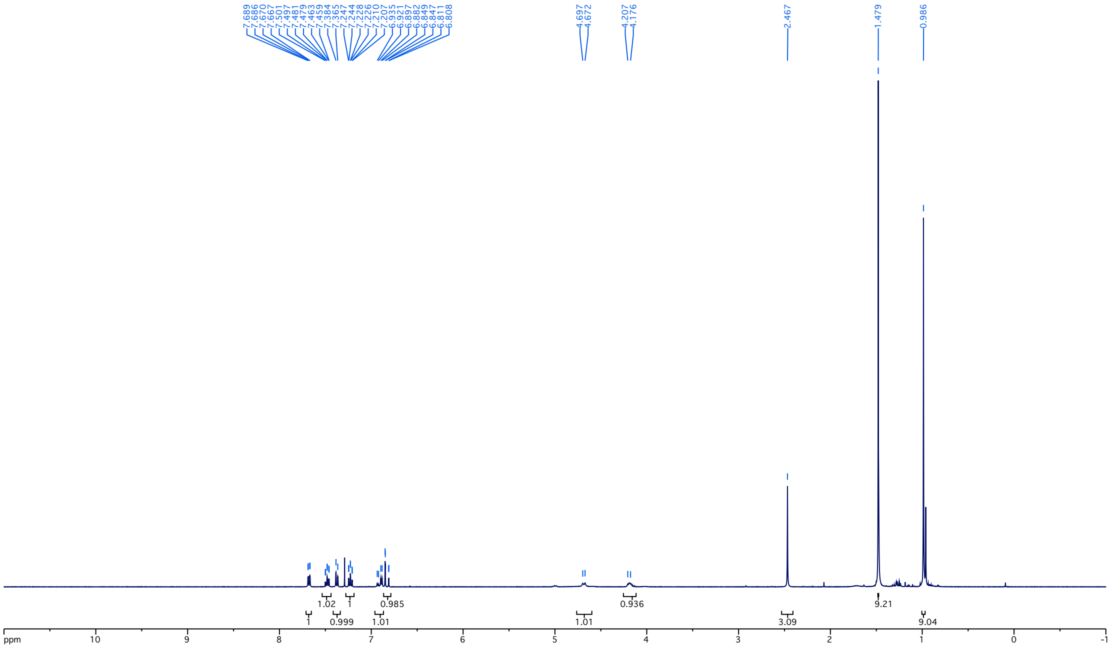

γ-Amino enone 3d: ^{13}C NMR (101 MHz, $CDCl_3$)

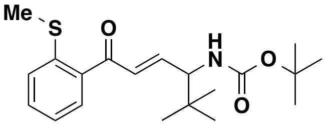

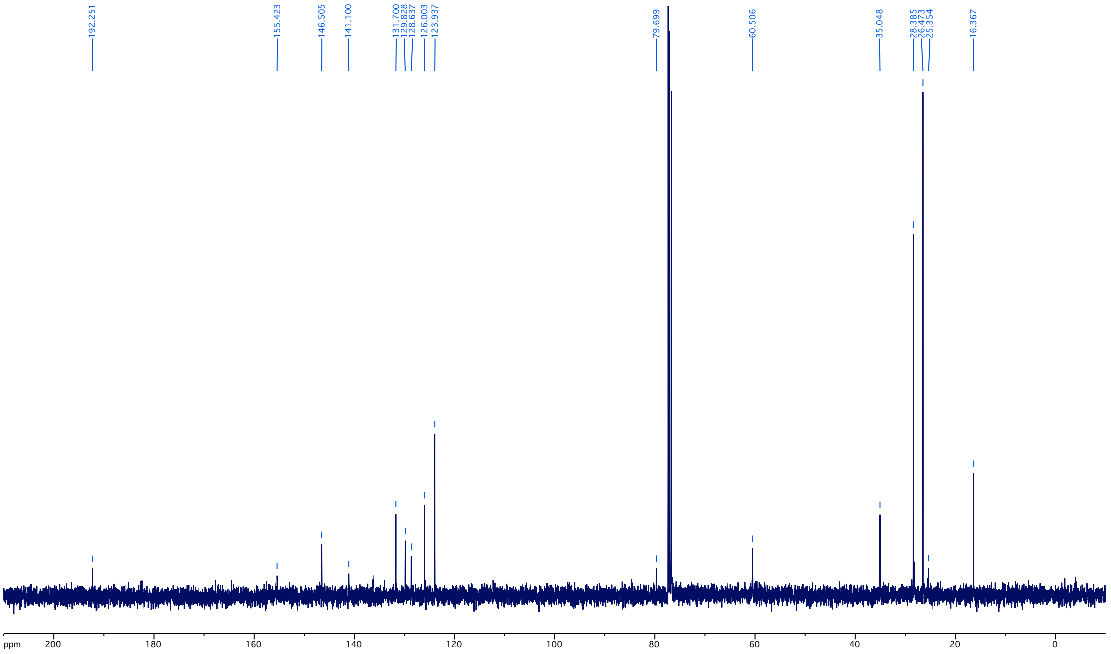

γ-Amino enone 3e: ^{1}H NMR (400 MHz, $CDCl_3$)

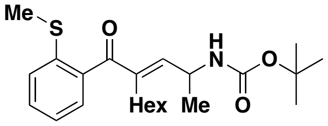

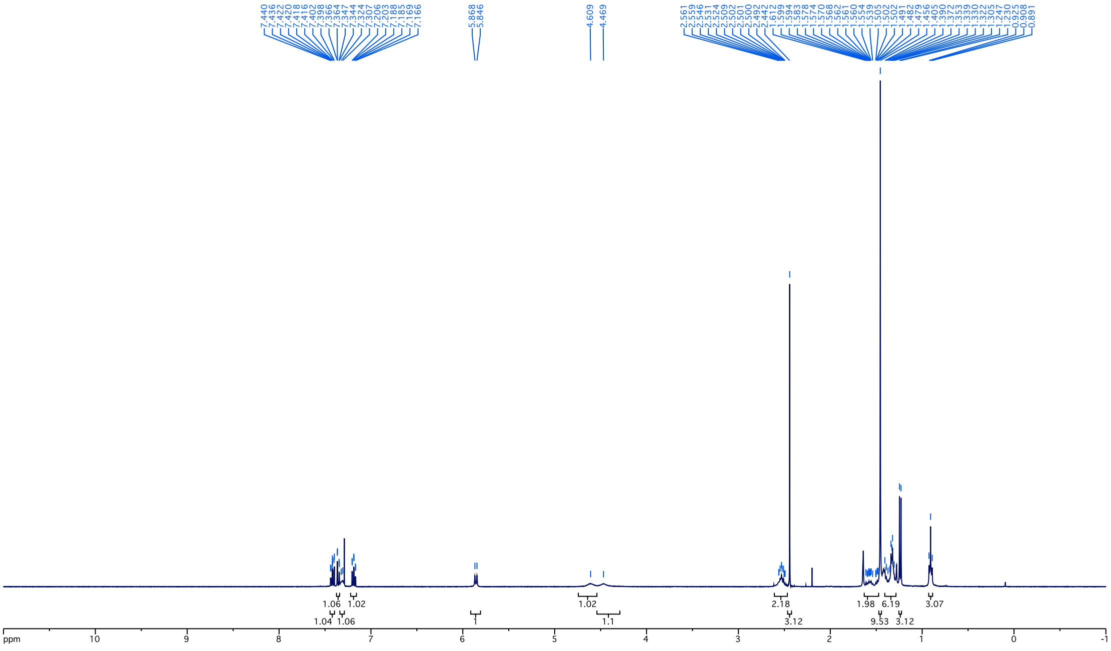

γ-Amino enone 3e: ^{13}C NMR (101 MHz, $CDCl_3$)

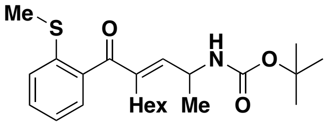

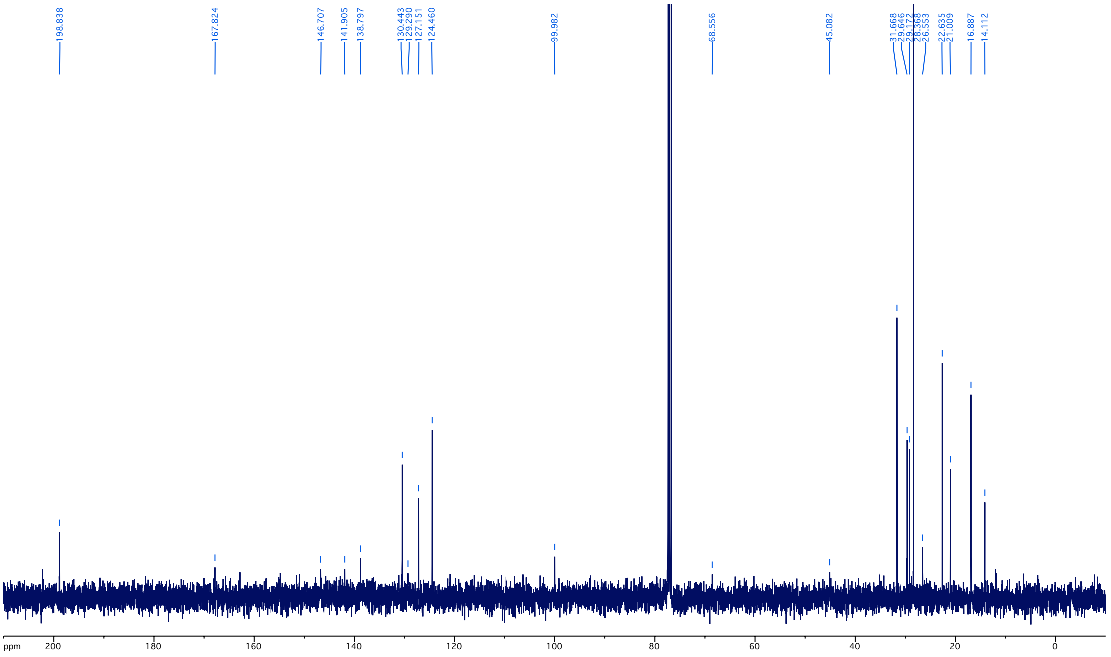

γ-Amino enone 3f: ^{1}H NMR (400 MHz, $CDCl_3$)

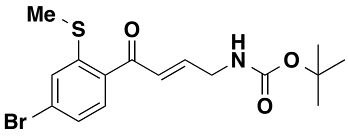

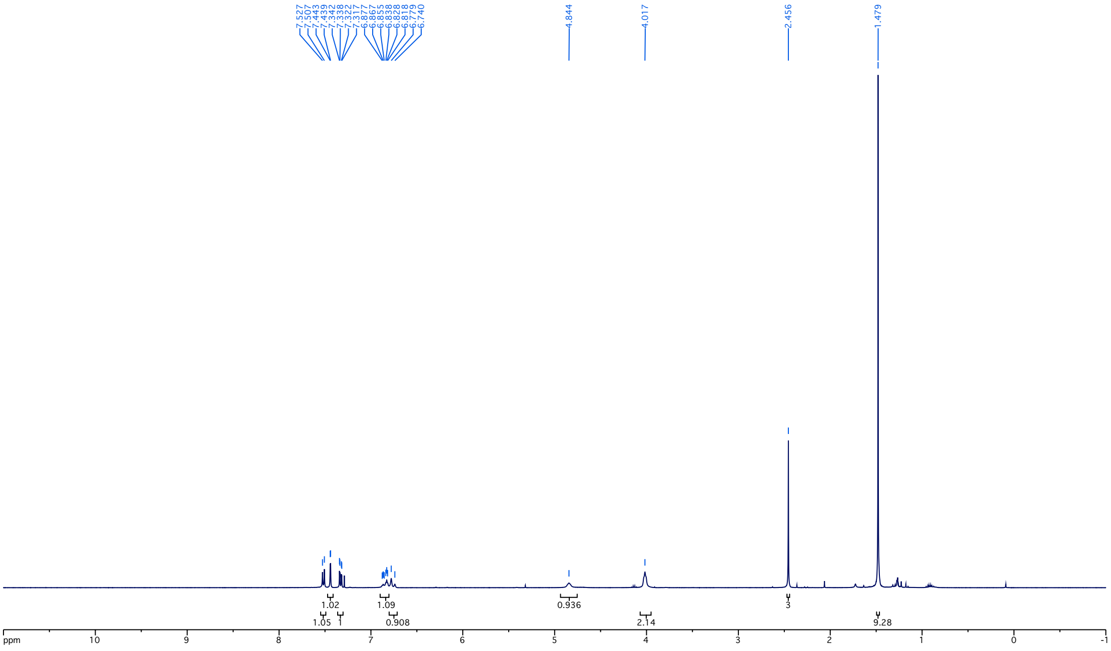

γ-Amino enone 3f: ^{13}C NMR (101 MHz, $CDCl_3$)

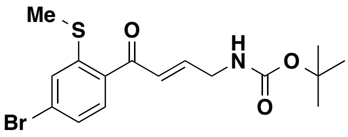

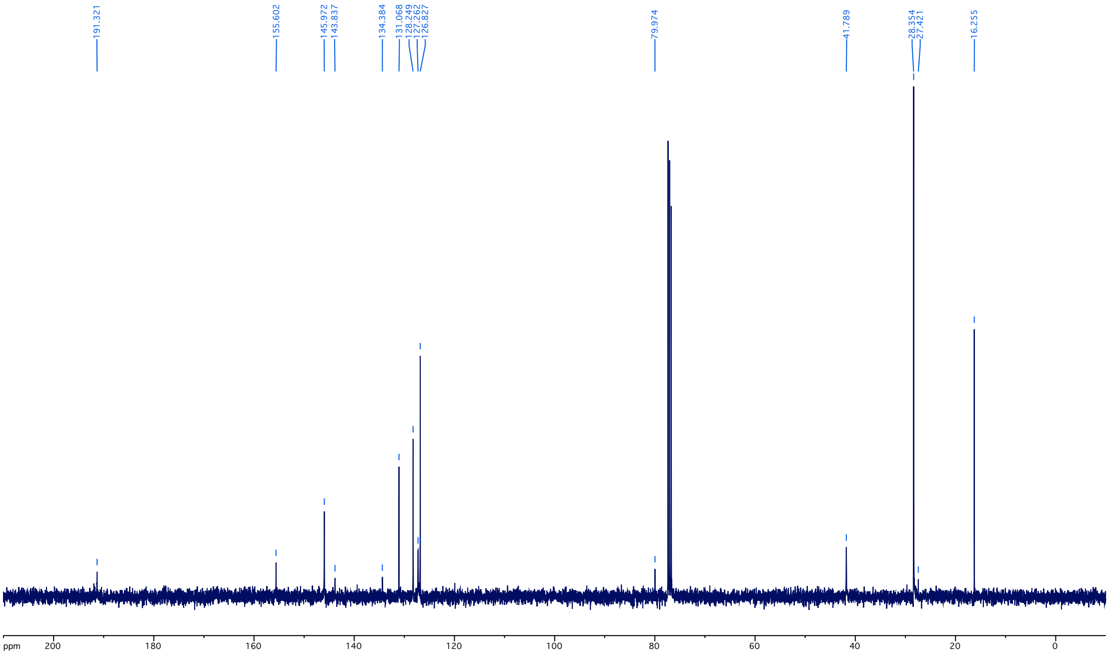

γ-Amino enone 3g: ^{1}H NMR (400 MHz, $CDCl_3$)

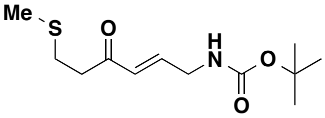

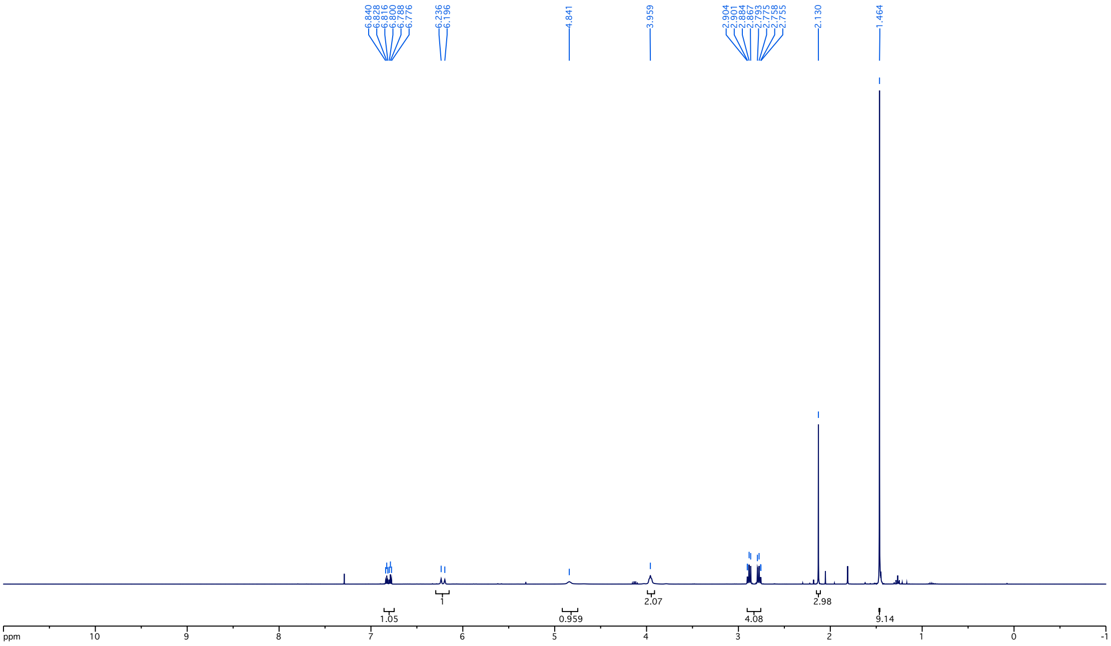

γ-Amino enone 3g: ^{13}C NMR (101 MHz, $CDCl_3$)

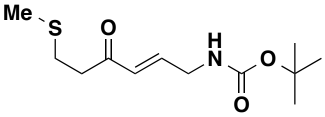

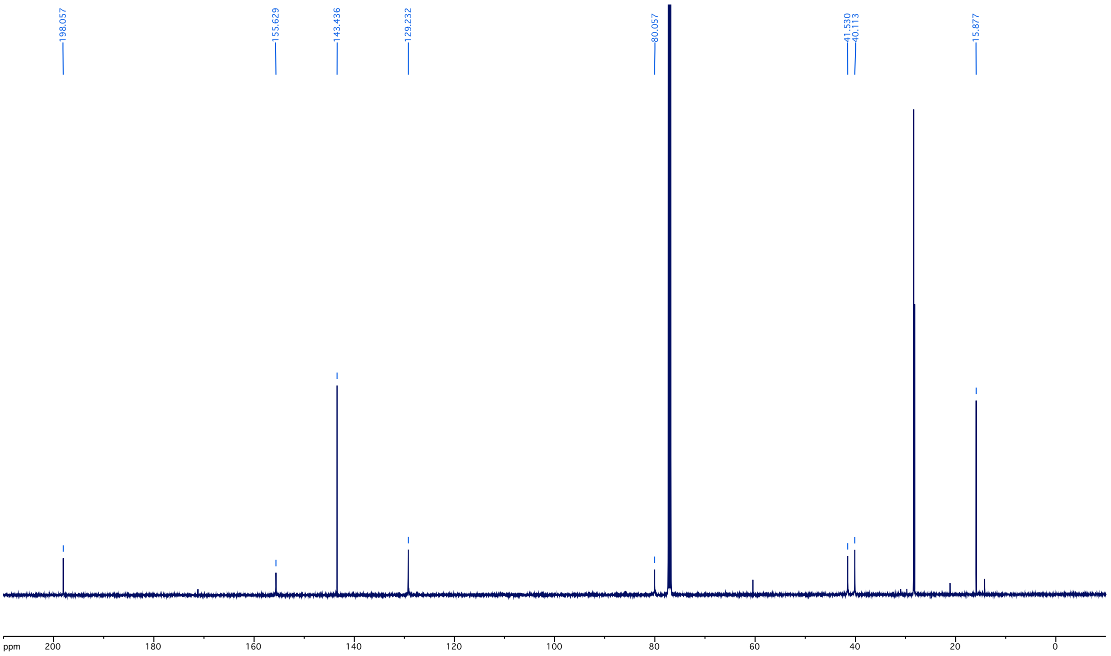

γ-Amino enone 3h: ^{1}H NMR (400 MHz, $CDCl_3$)

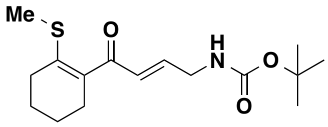

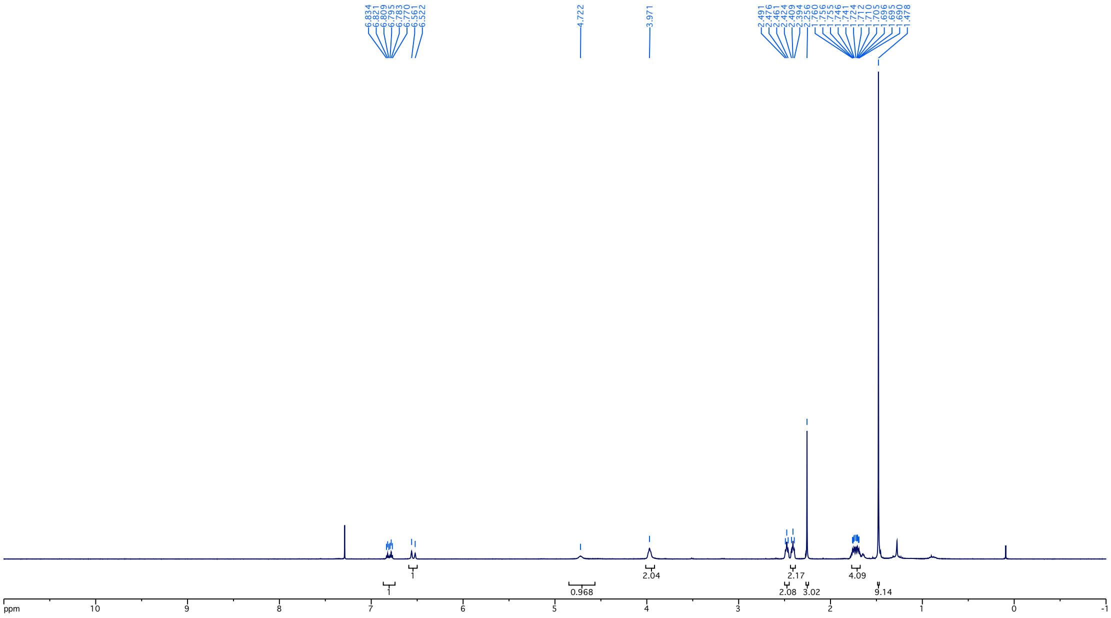

γ-Amino enone 3h: ^{13}C NMR (101 MHz, $CDCl_3$)

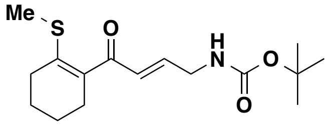

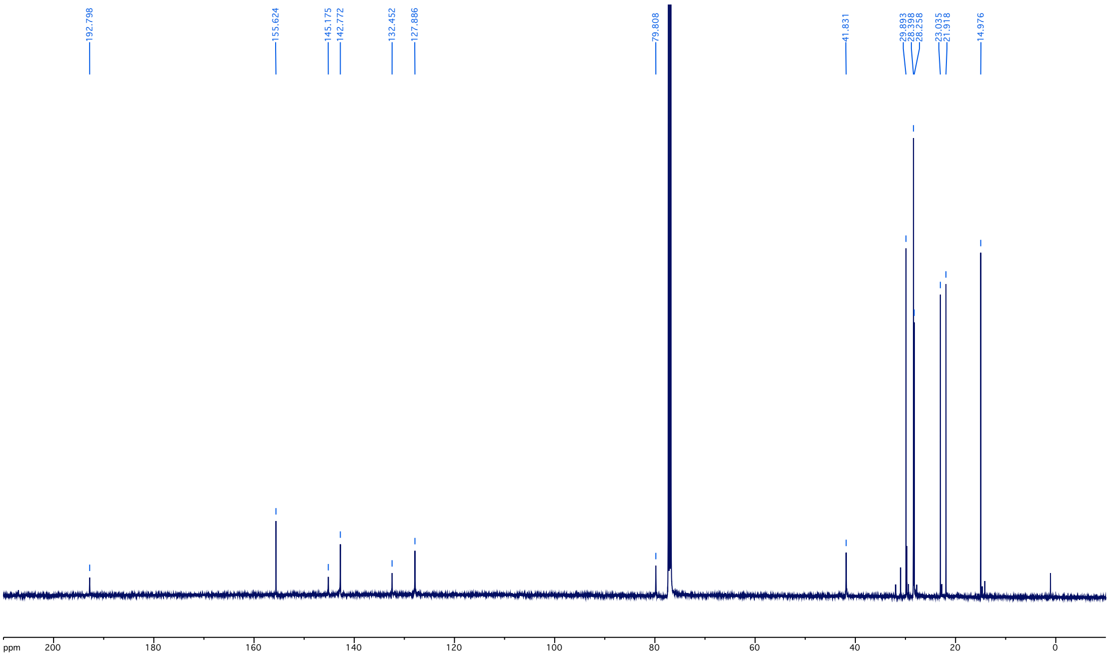

γ-Amino enone 3i: ^{1}H NMR (400 MHz, $CDCl_3$)

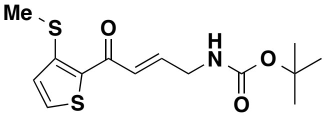

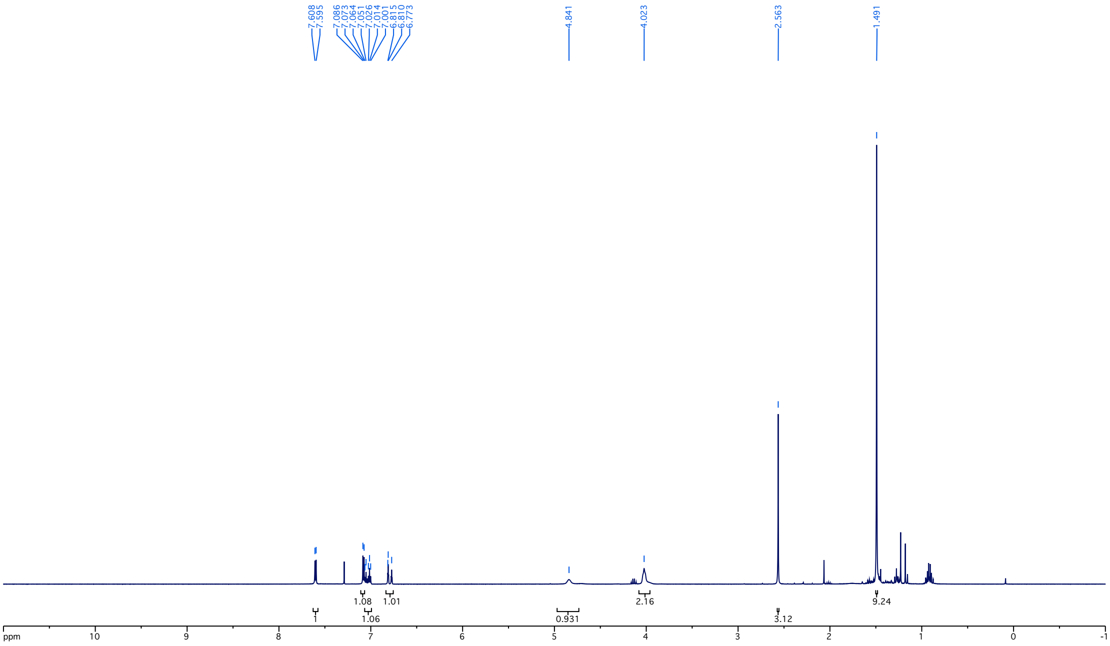

γ-Amino enone 3i: ^{13}C NMR (101 MHz, $CDCl_3$)

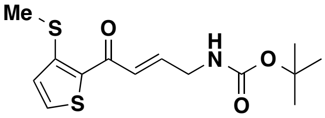

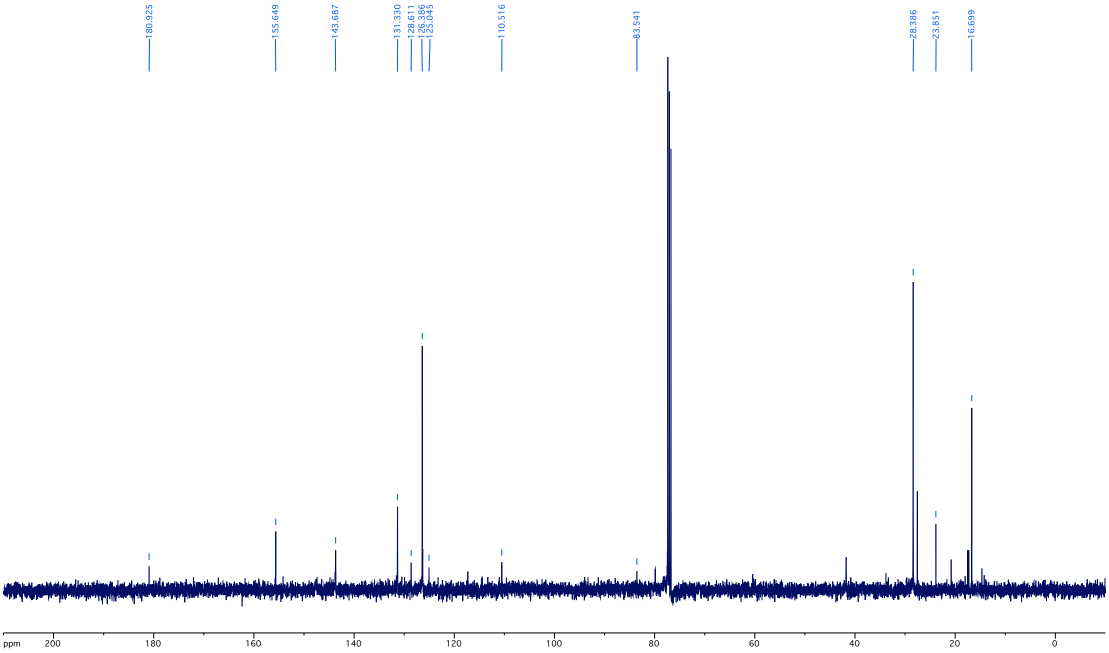

γ-Amino enone 3j: ^{1}H NMR (400 MHz, $CDCl_3$)

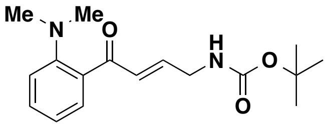

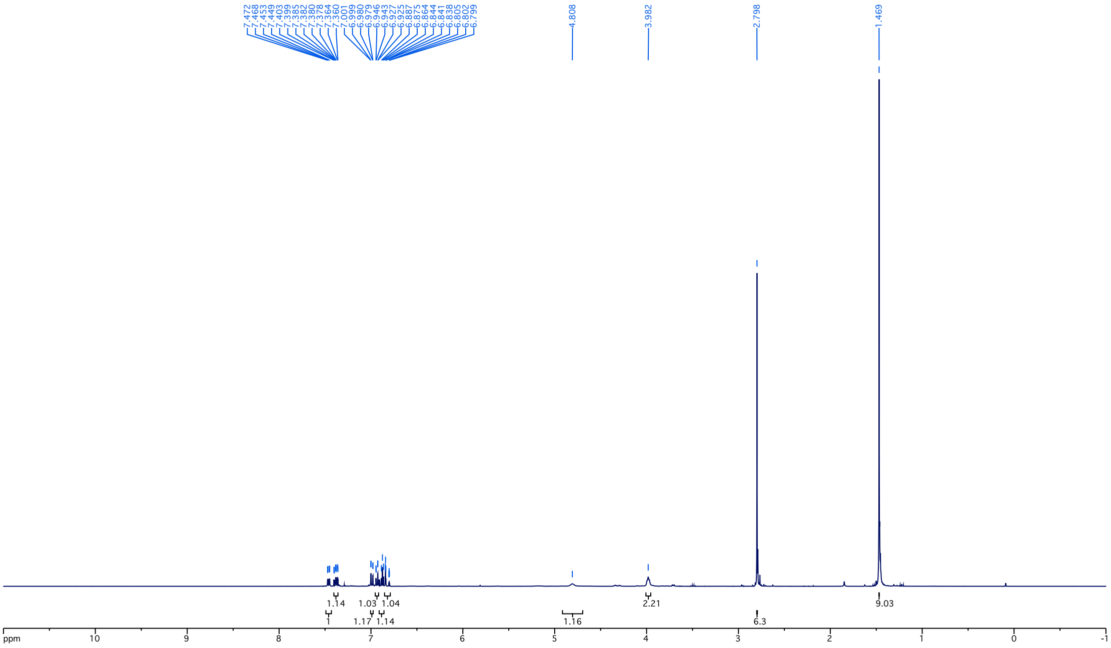

γ-Amino enone 3j: ^{13}C NMR (101 MHz, $CDCl_3$)

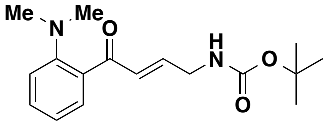

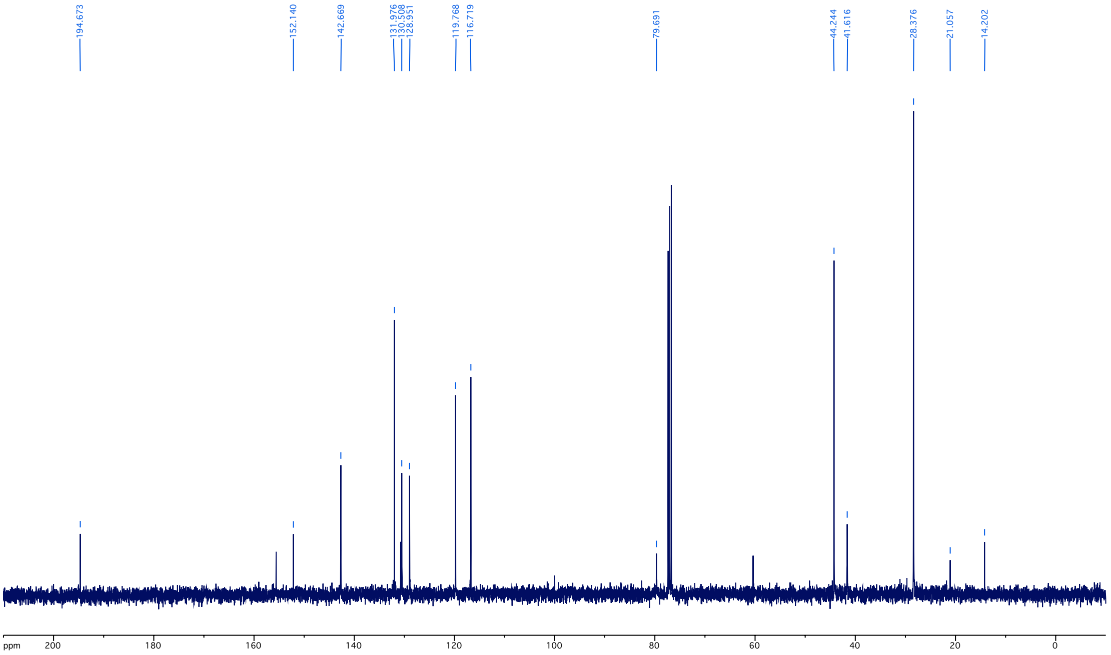

Pyrrole 4a: ^{1}H NMR (400 MHz, $CDCl_3$)

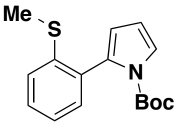

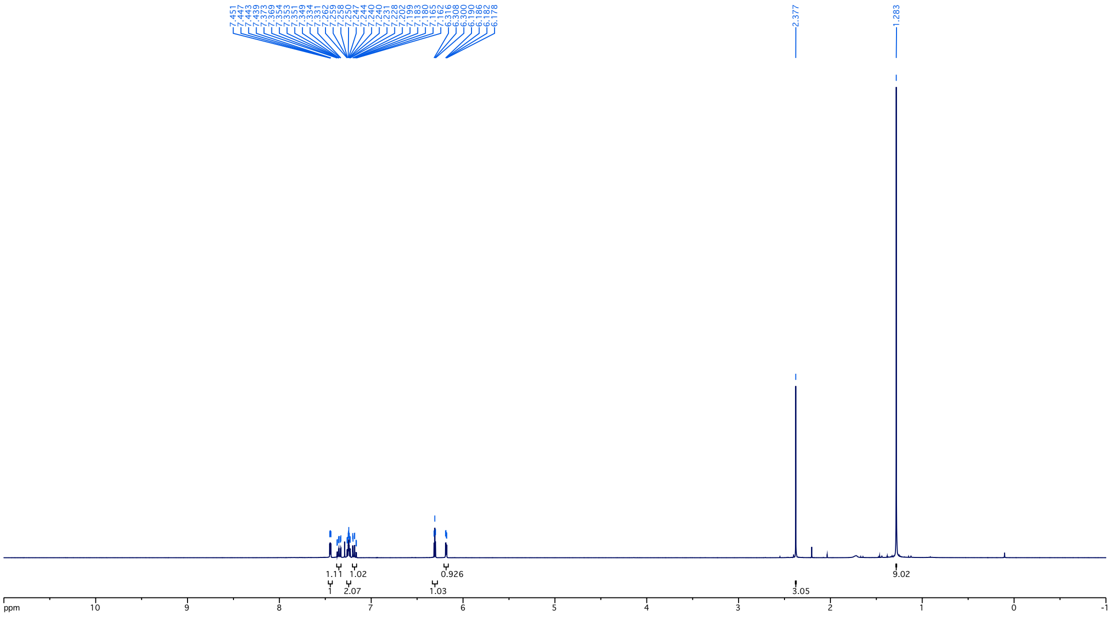

Pyrrole 4a: ^{13}C NMR (101 MHz, $CDCl_3$)

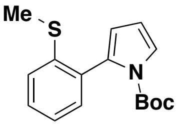

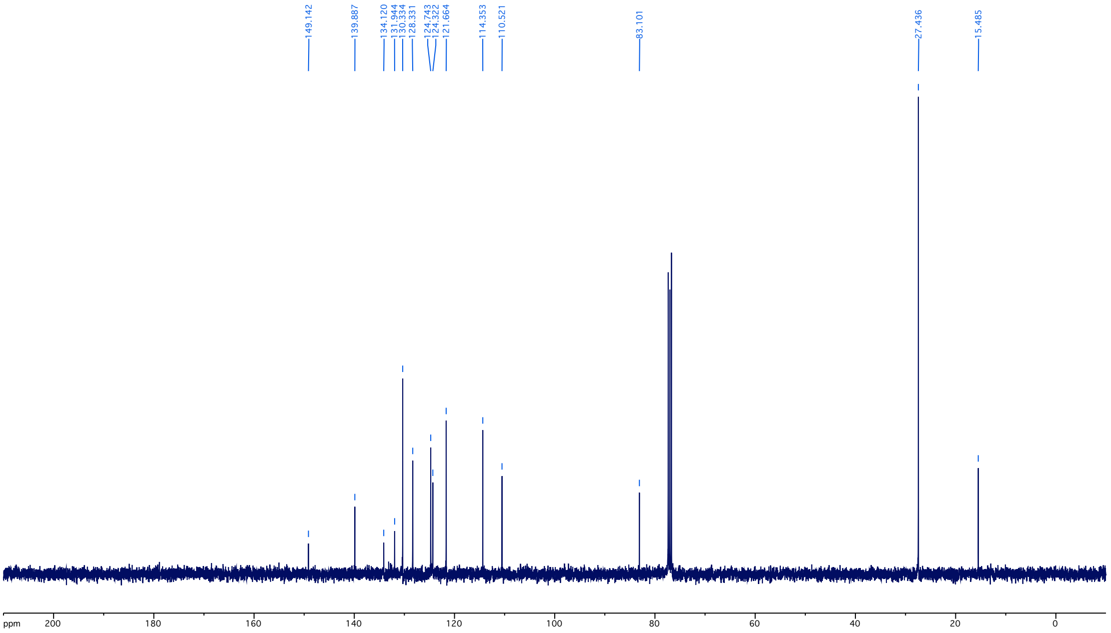

Pyrrole 4b: ^{1}H NMR (400 MHz, $CDCl_3$)

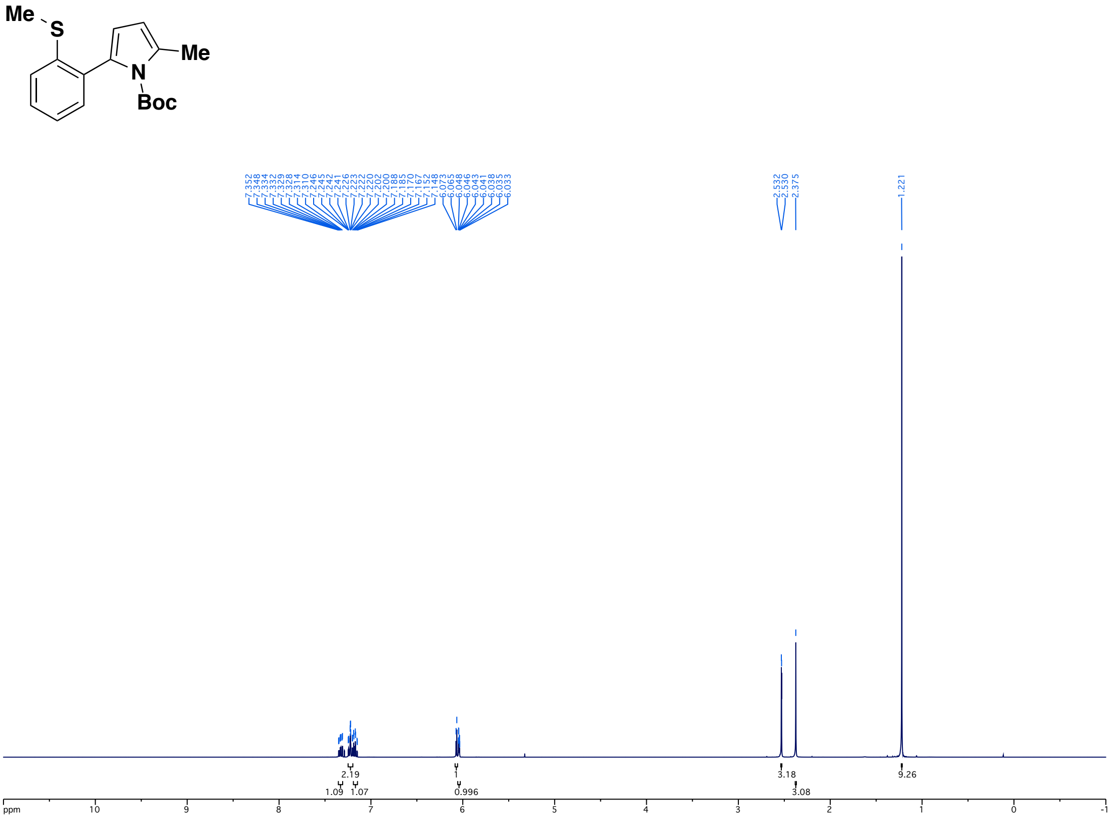

Pyrrole 4b: ^{13}C NMR (101 MHz, $CDCl_3$)

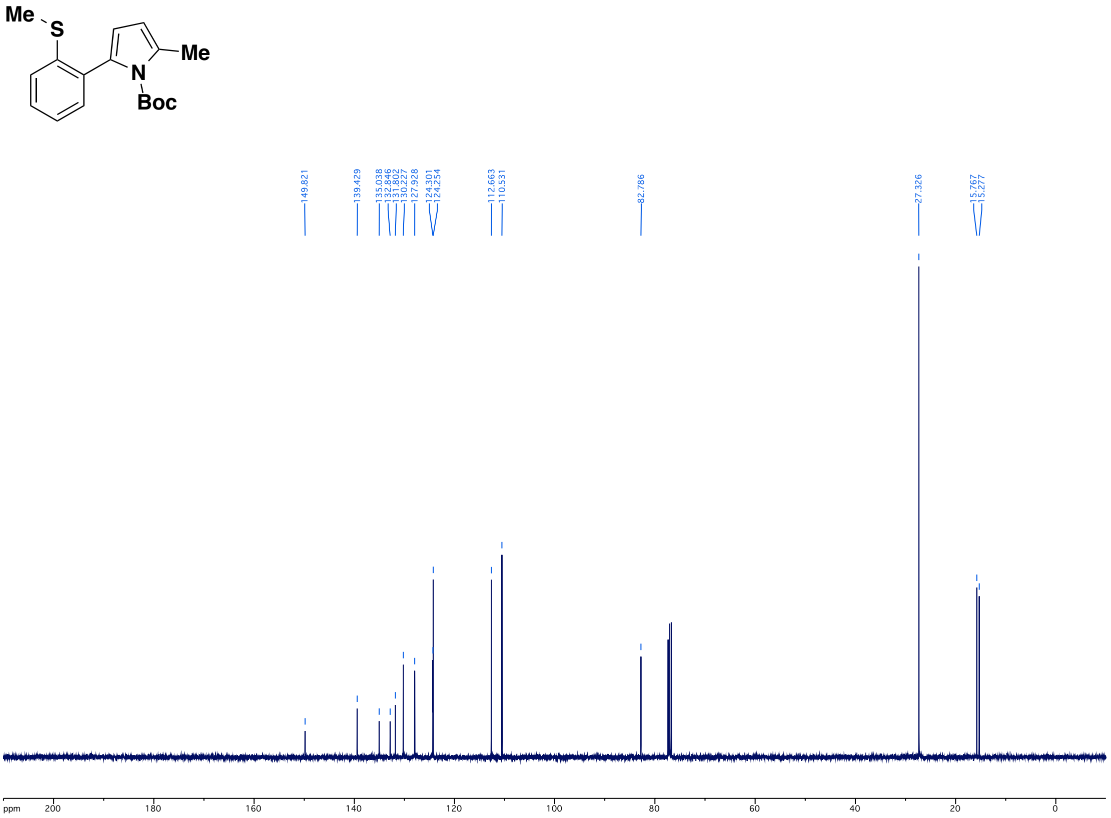

Pyrrole 4c: ^{1}H NMR (400 MHz, $CDCl_3$)

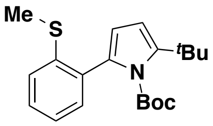

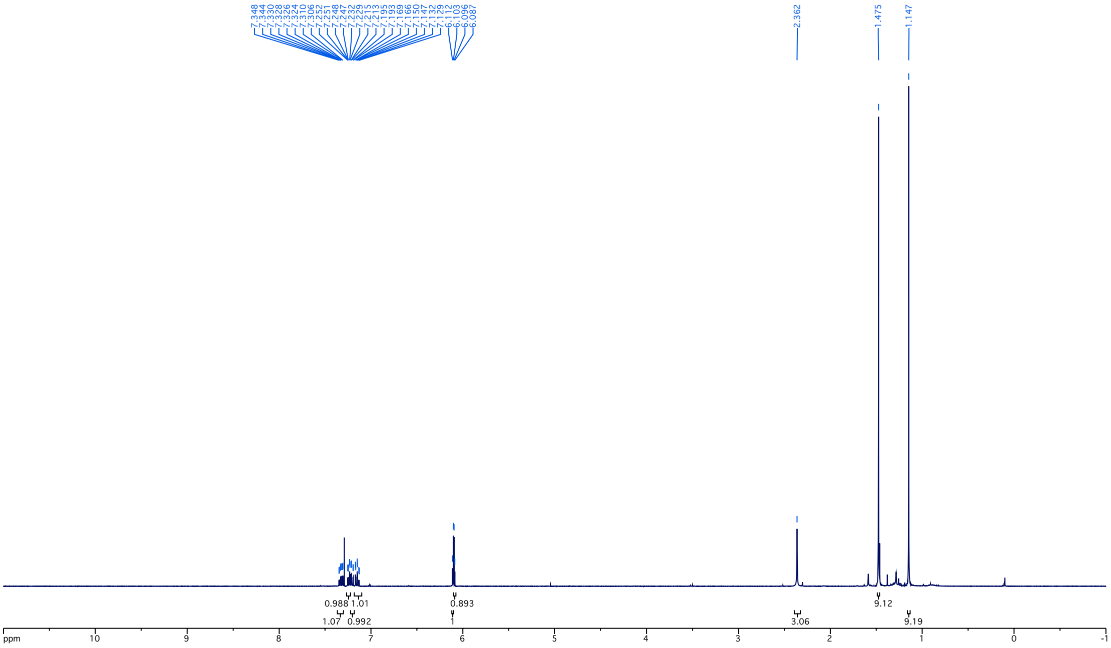

Pyrrole 4c: ^{13}C NMR (101 MHz, $CDCl_3$)

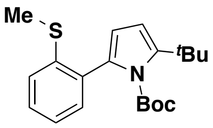

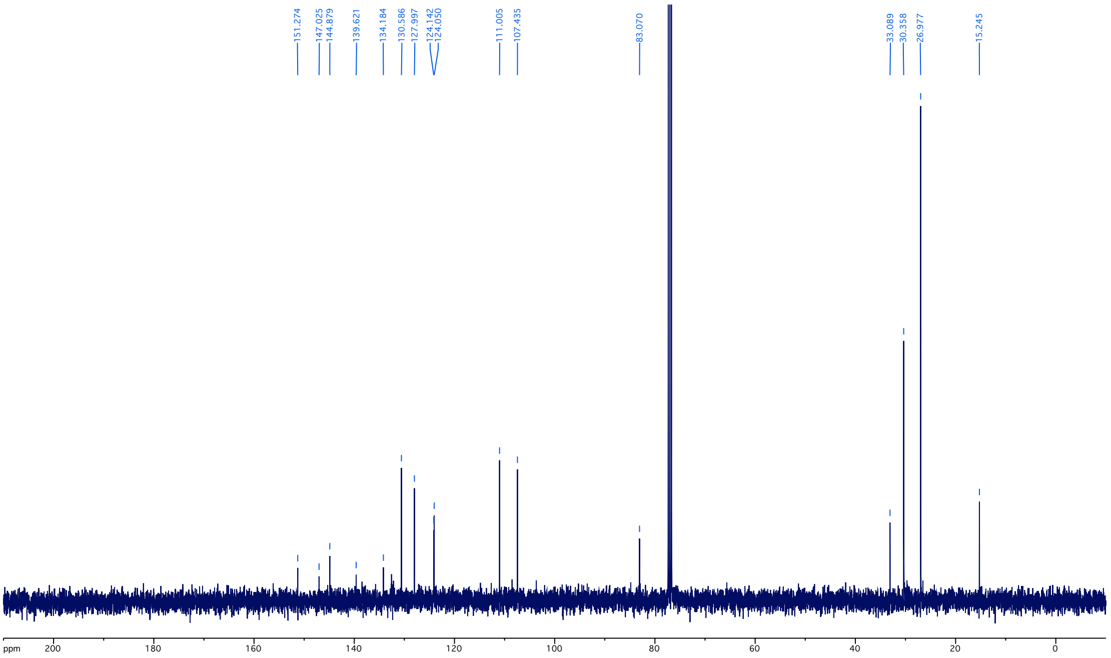

Pyrrole 4d: ^{1}H NMR (400 MHz, $CDCl_3$)

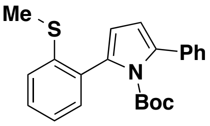

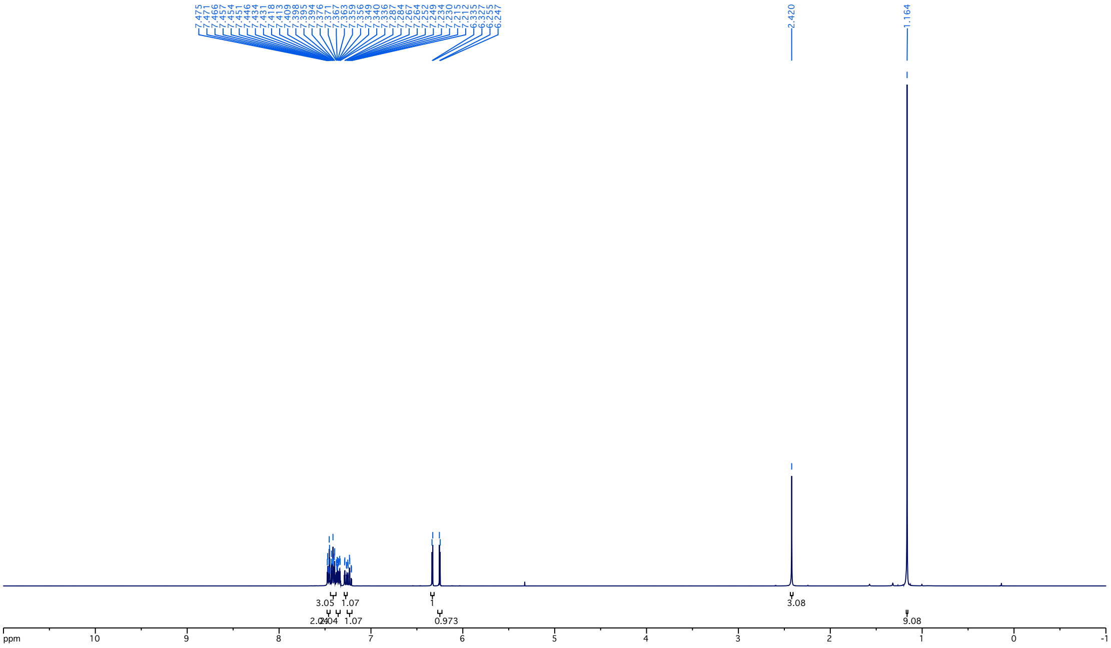

Pyrrole 4d: ^{13}C NMR (101 MHz, $CDCl_3$)

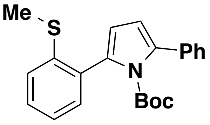

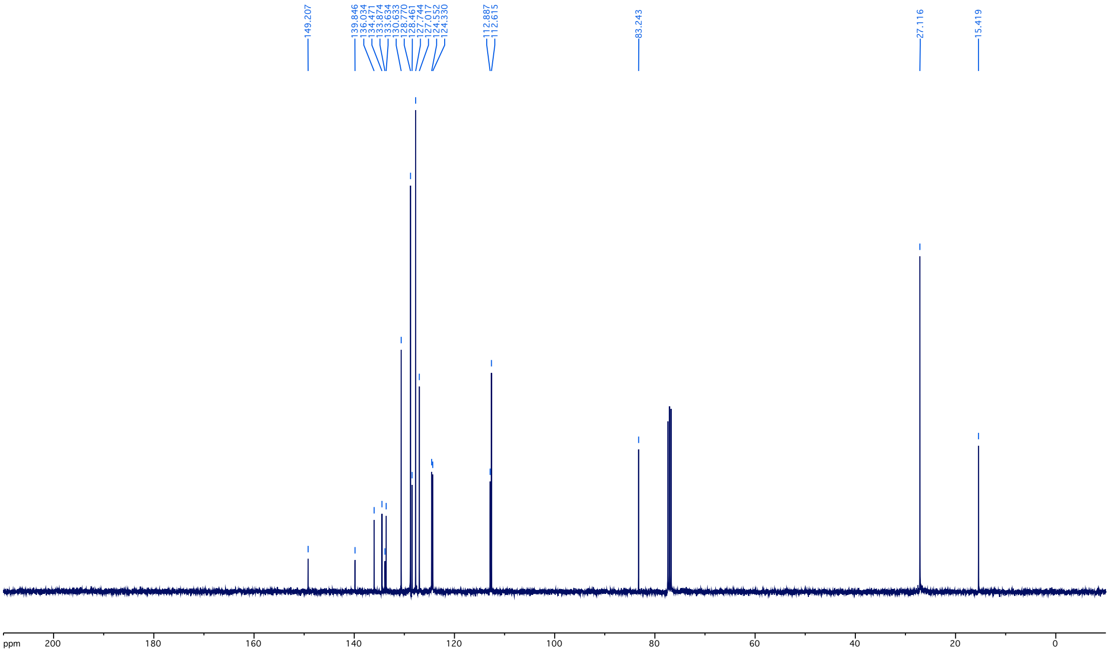

Pyrrole 4e: ^{1}H NMR (400 MHz, $CDCl_3$)

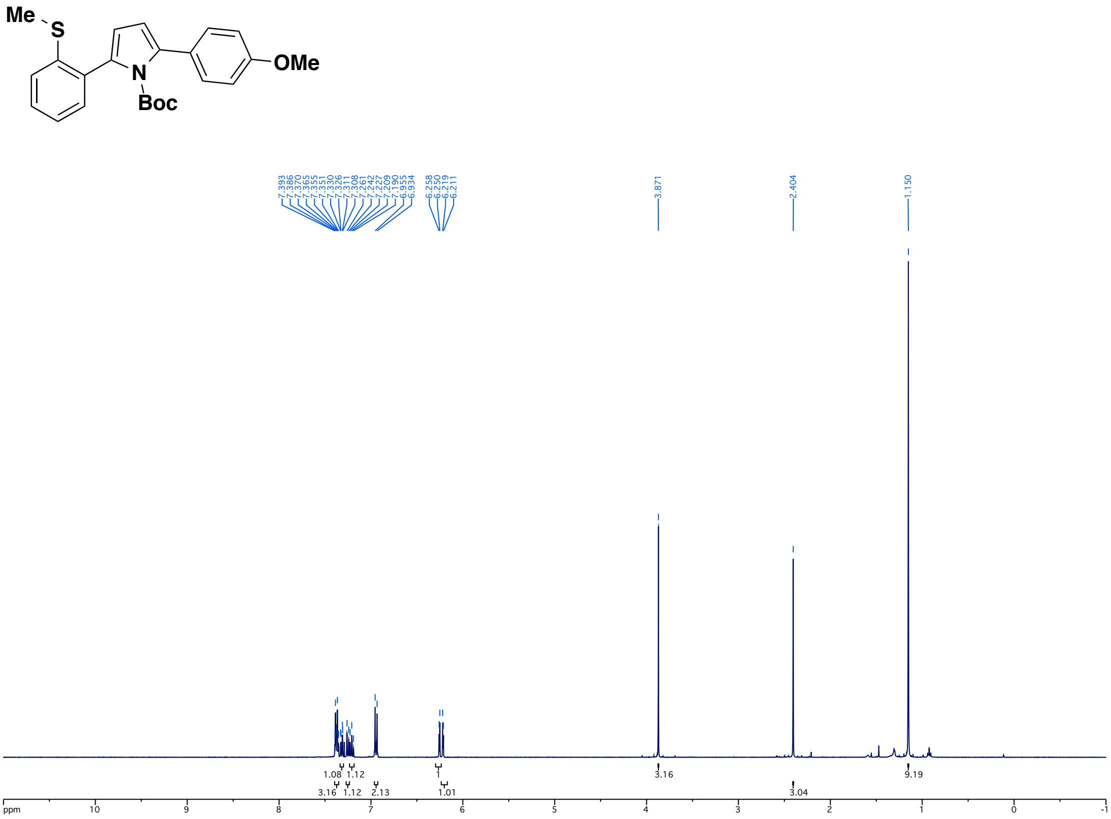

Pyrrole 4e: ^{13}C NMR (101 MHz, $CDCl_3$)

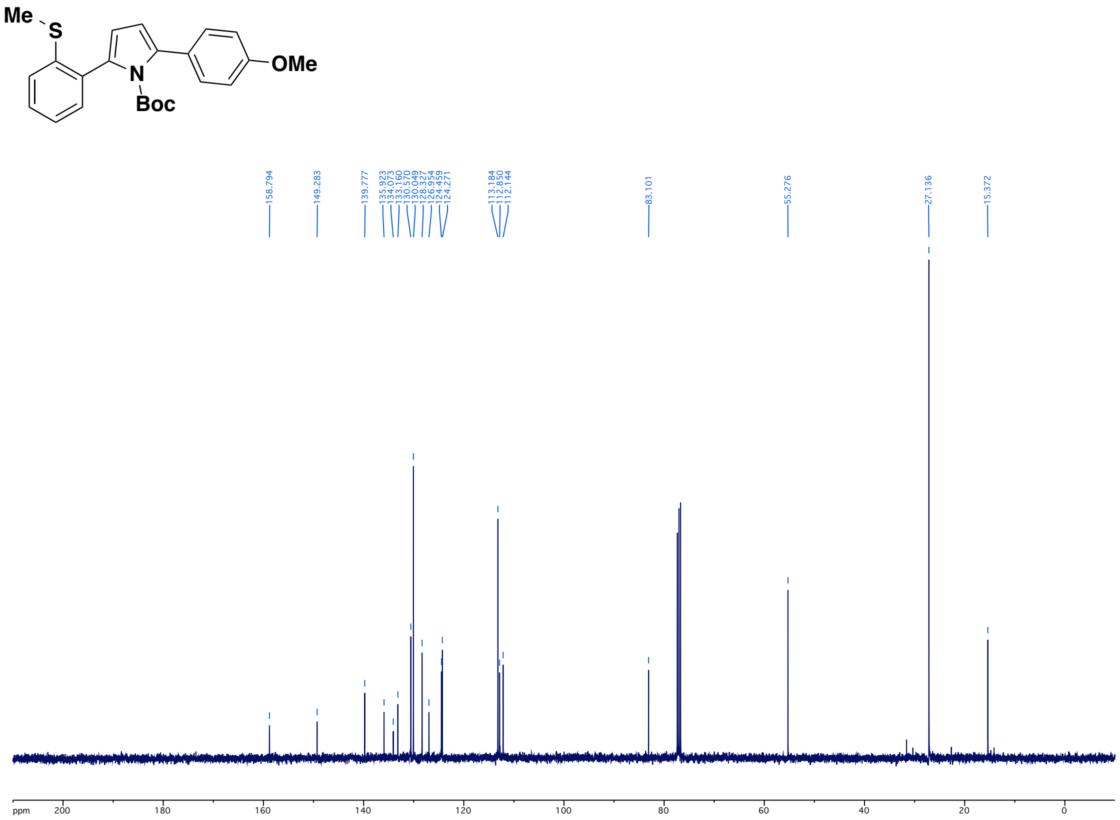

Pyrrole 4f: ^{1}H NMR (400 MHz, $CDCl_3$)

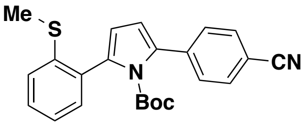

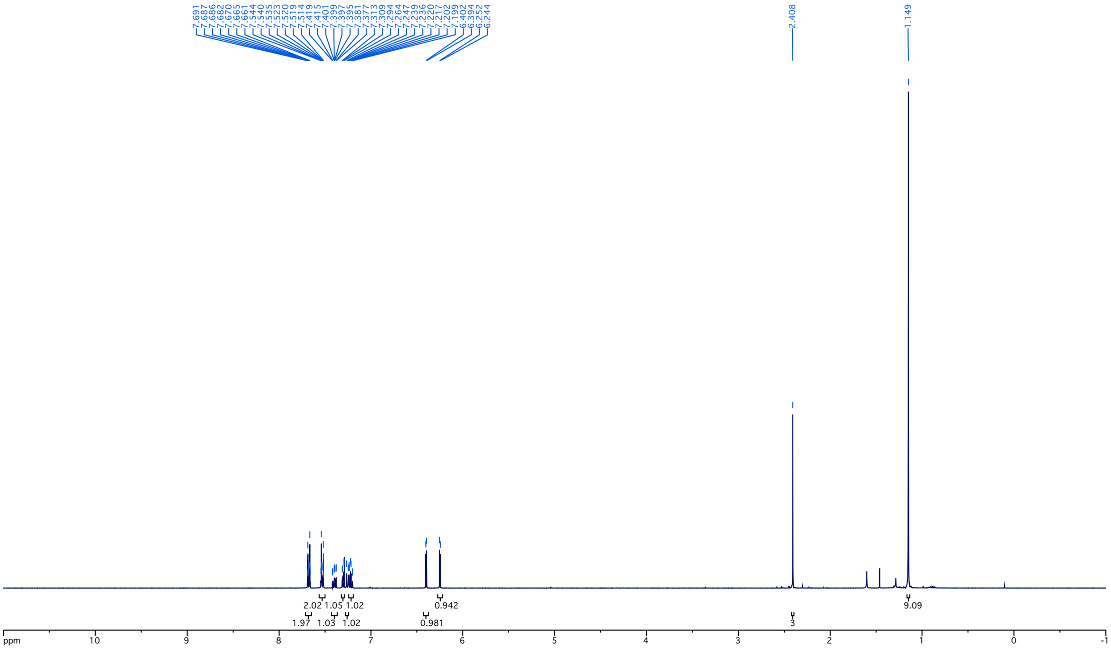

Pyrrole 4f: ^{13}C NMR (101 MHz, $CDCl_3$)

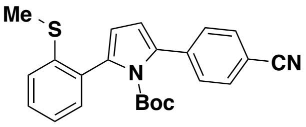

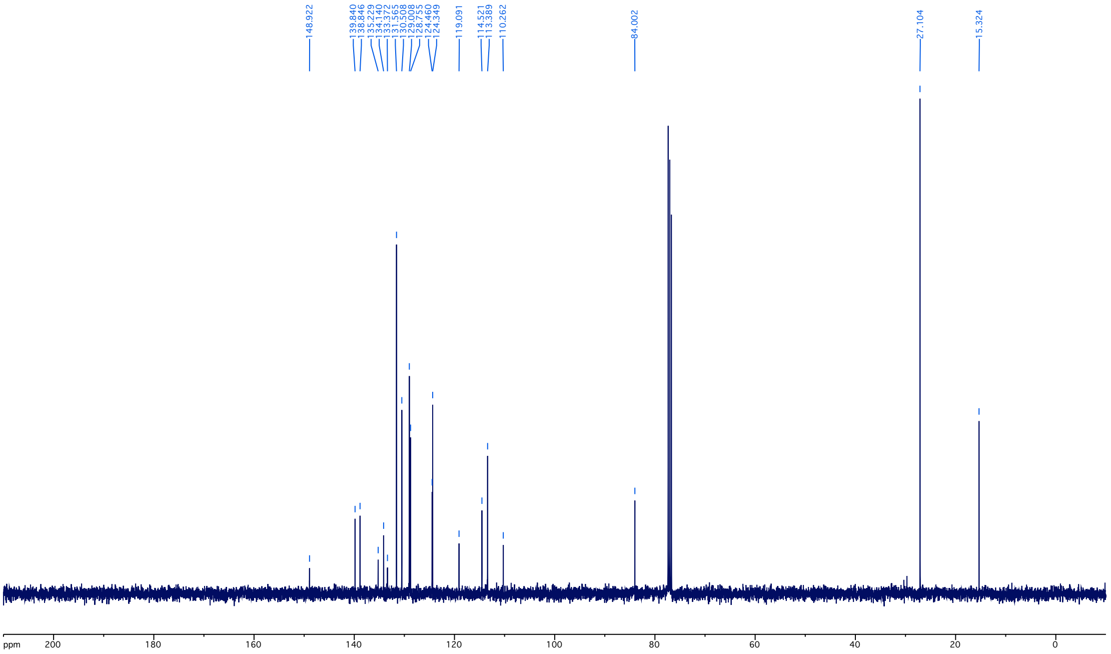

Pyrrole 4g: ^{1}H NMR (400 MHz, $CDCl_3$)

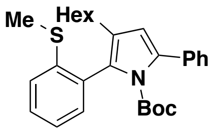

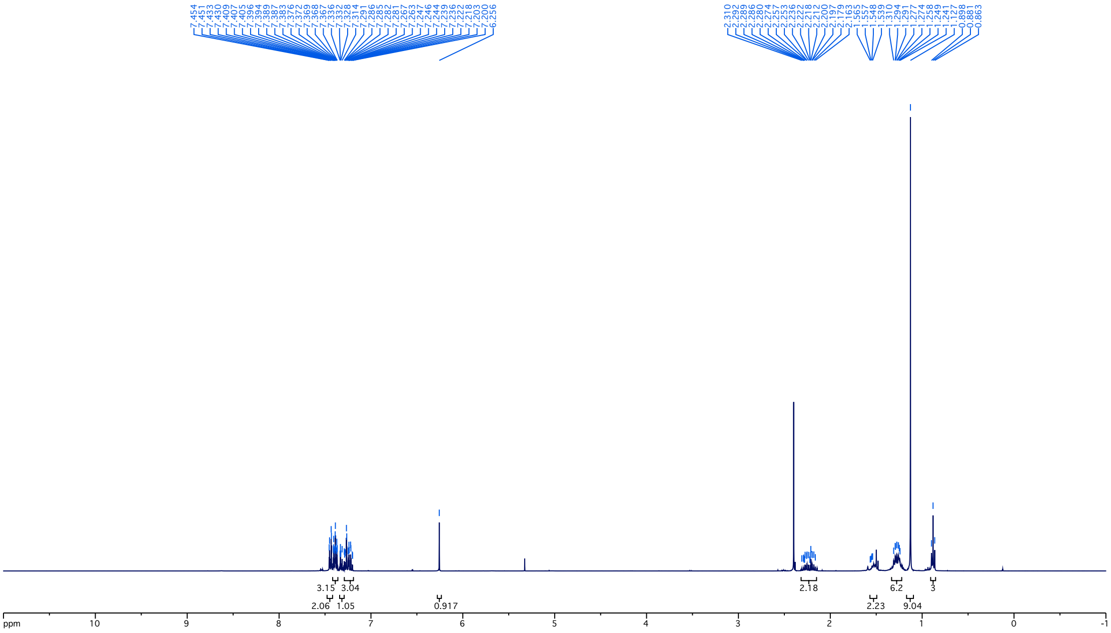

Pyrrole 4g: ^{13}C NMR (101 MHz, $CDCl_3$)

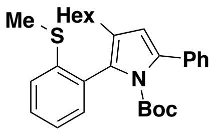

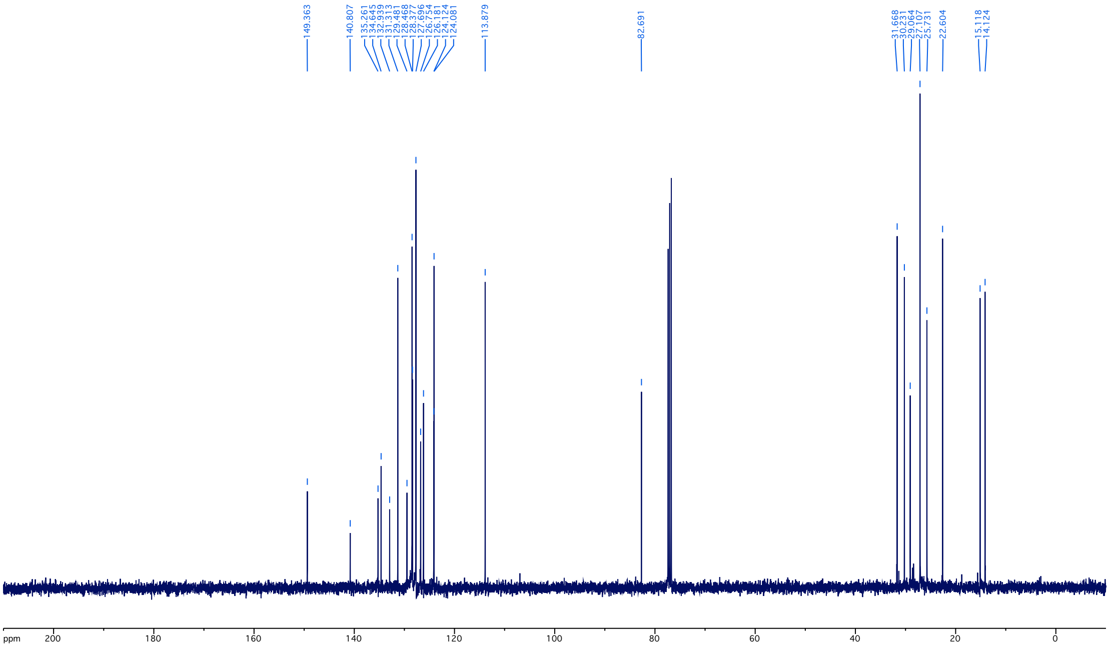

Pyrrole 4h: ^{1}H NMR (400 MHz, $CDCl_3$)

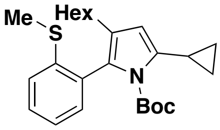

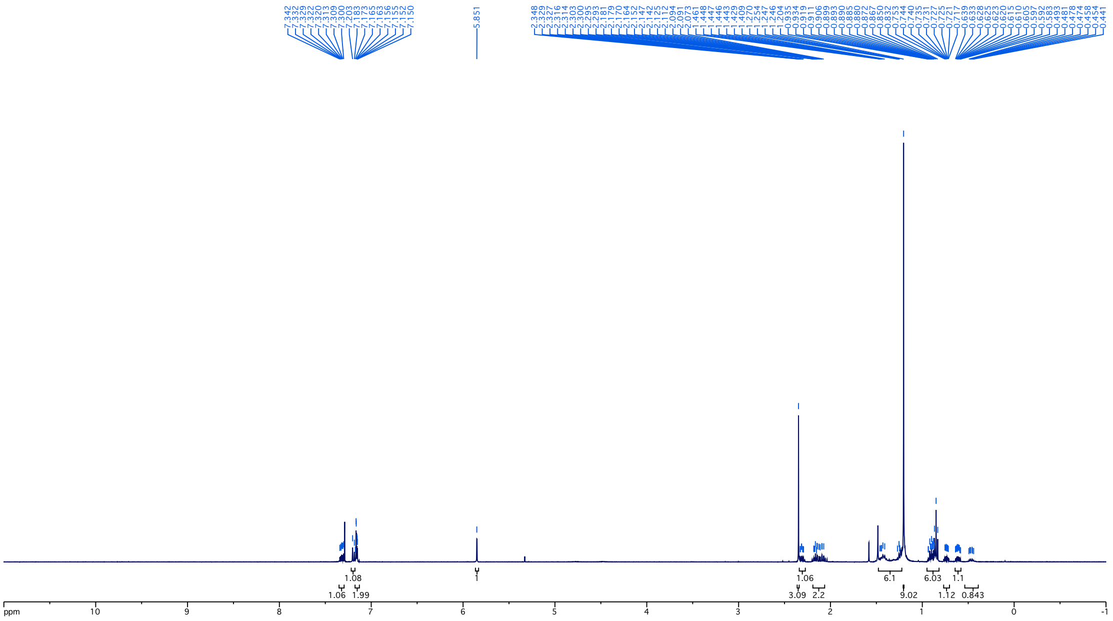

Pyrrole 4h: ^{13}C NMR (101 MHz, $CDCl_3$)

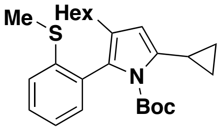

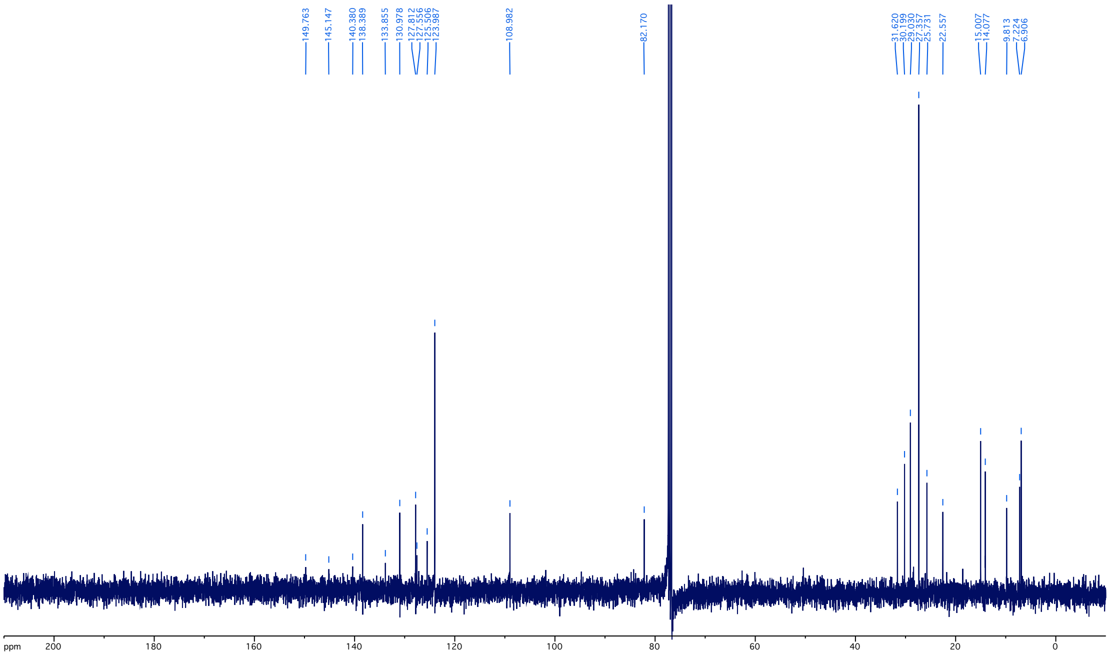

Pyrrole 4i: ^{1}H NMR (400 MHz, $CDCl_3$)

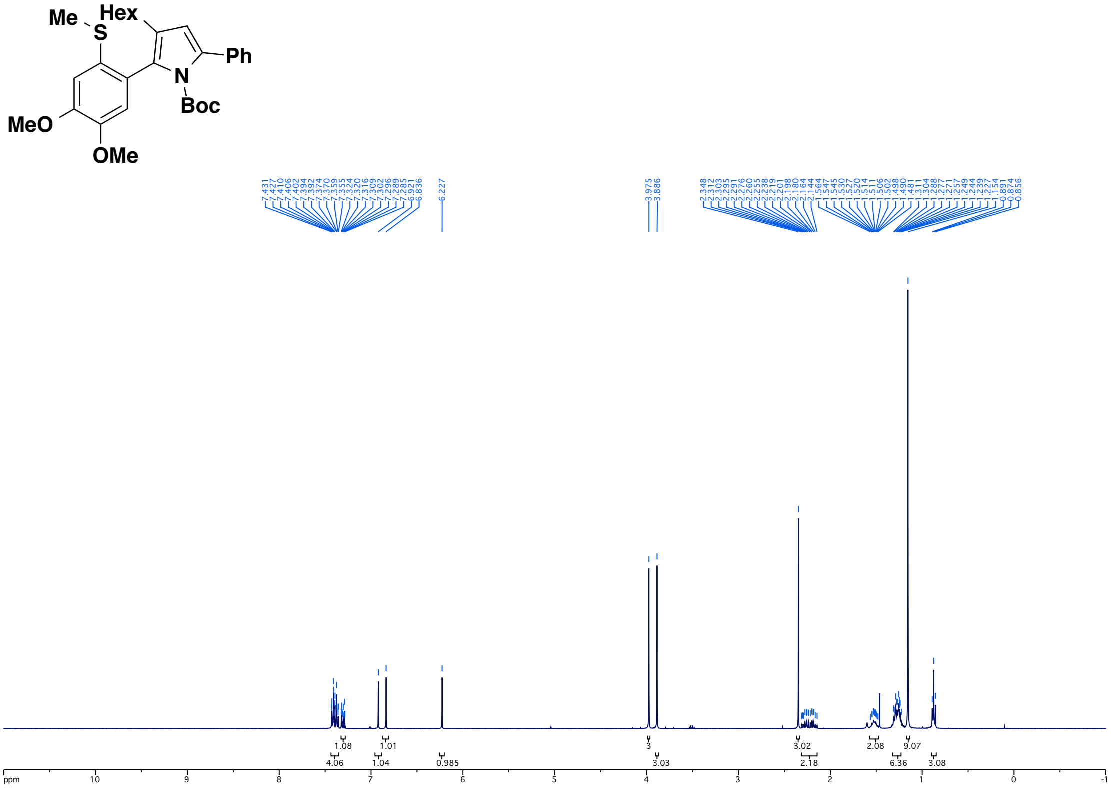

Pyrrole 4i: ^{13}C NMR (101 MHz, $CDCl_3$)

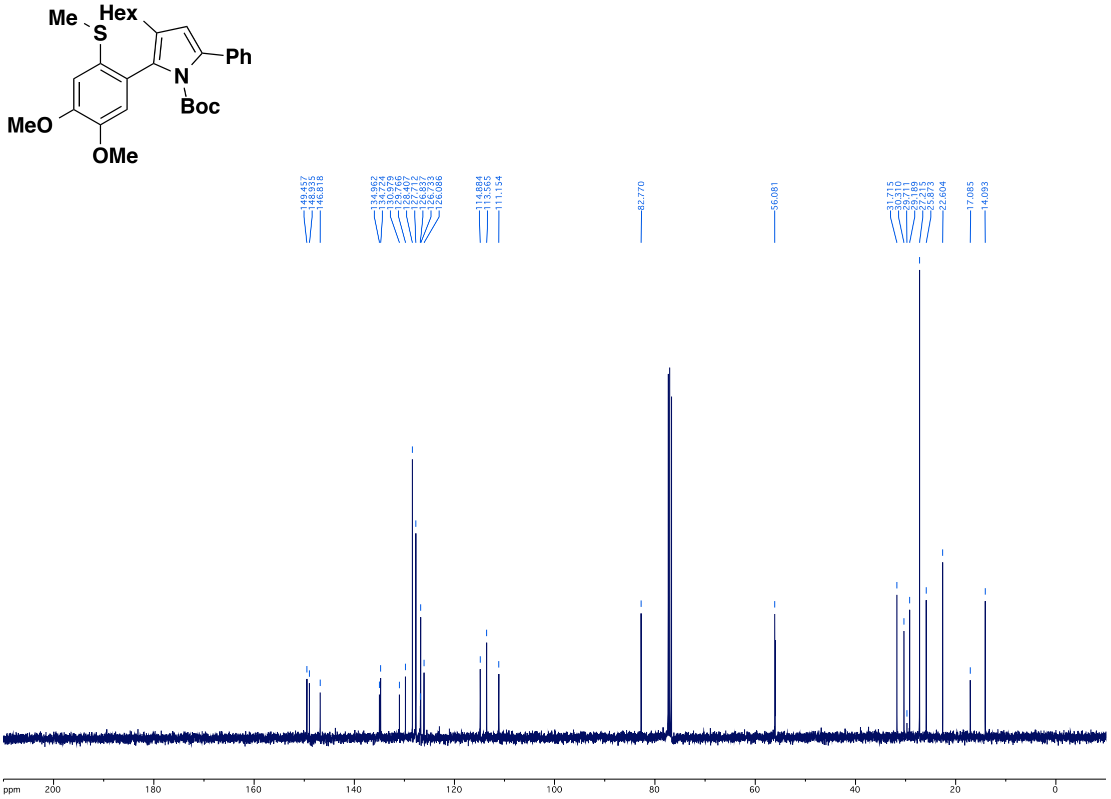

Pyrrole 4j: ^{1}H NMR (400 MHz, $CDCl_3$)

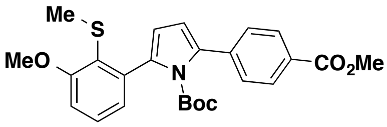

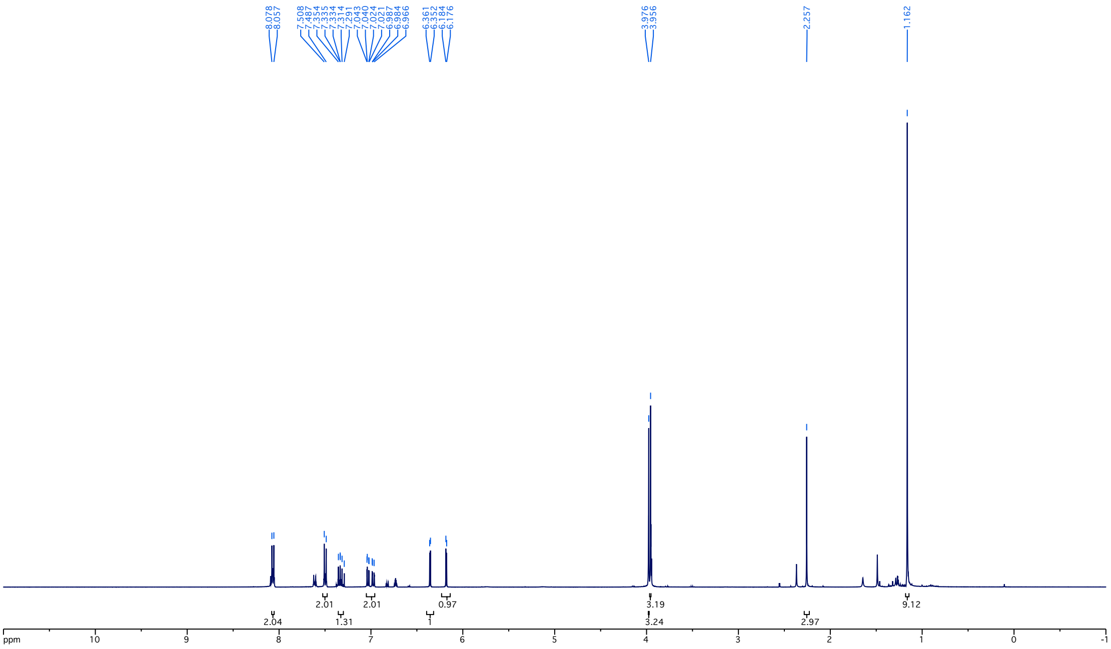

Pyrrole 4j: ^{13}C NMR (101 MHz, $CDCl_3$)

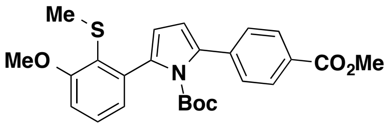

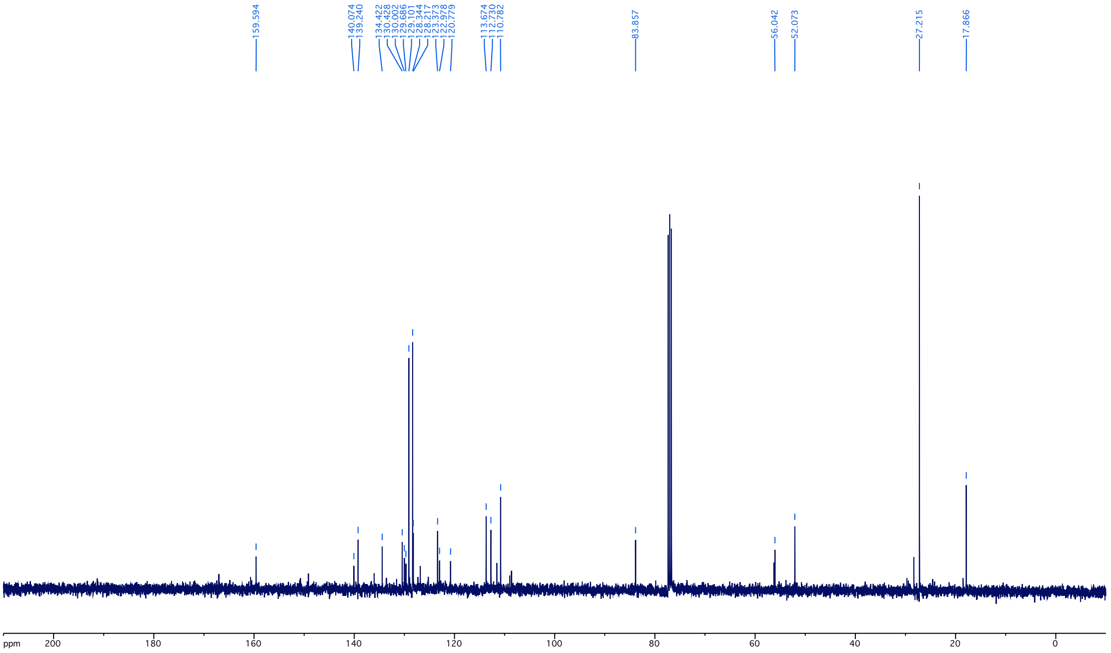

Pyrrole 4k: ^{1}H NMR (400 MHz, $CDCl_3$)

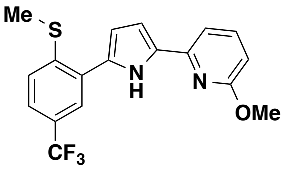

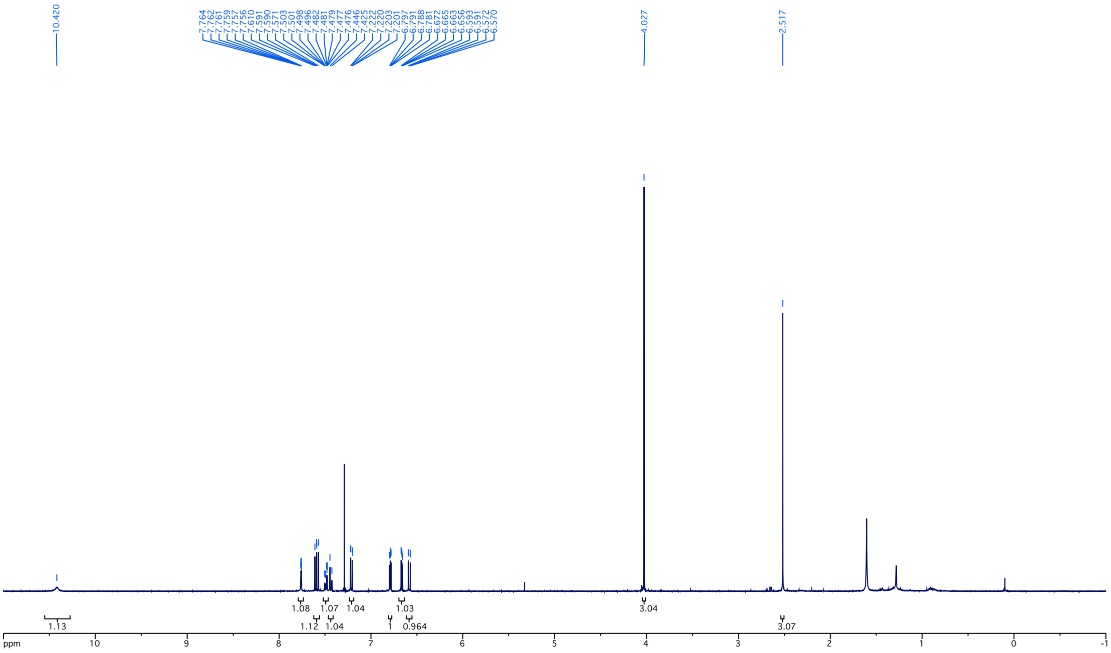

Pyrrole 4k: ^{13}C NMR (101 MHz, $CDCl_3$)

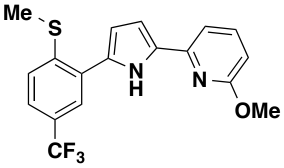

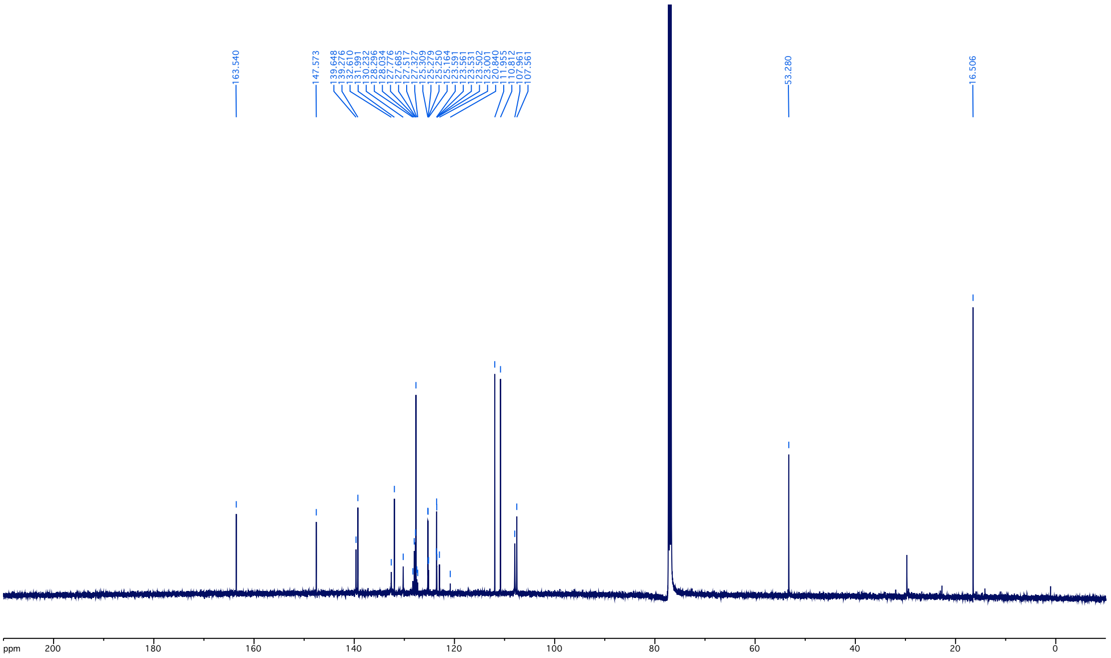

Pyrrole 4k: ^{19}F NMR (377 MHz, $CDCl_3$)

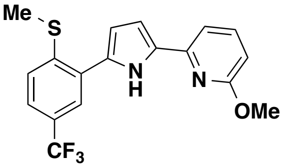

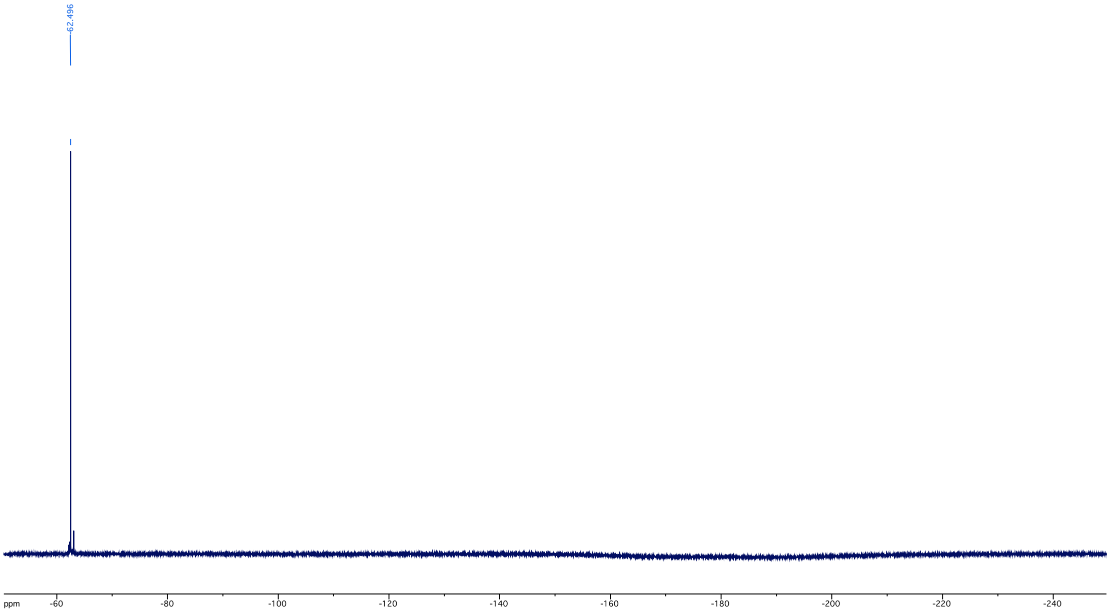

Pyrrole 4l: ^{1}H NMR (400 MHz, $CDCl_3$)

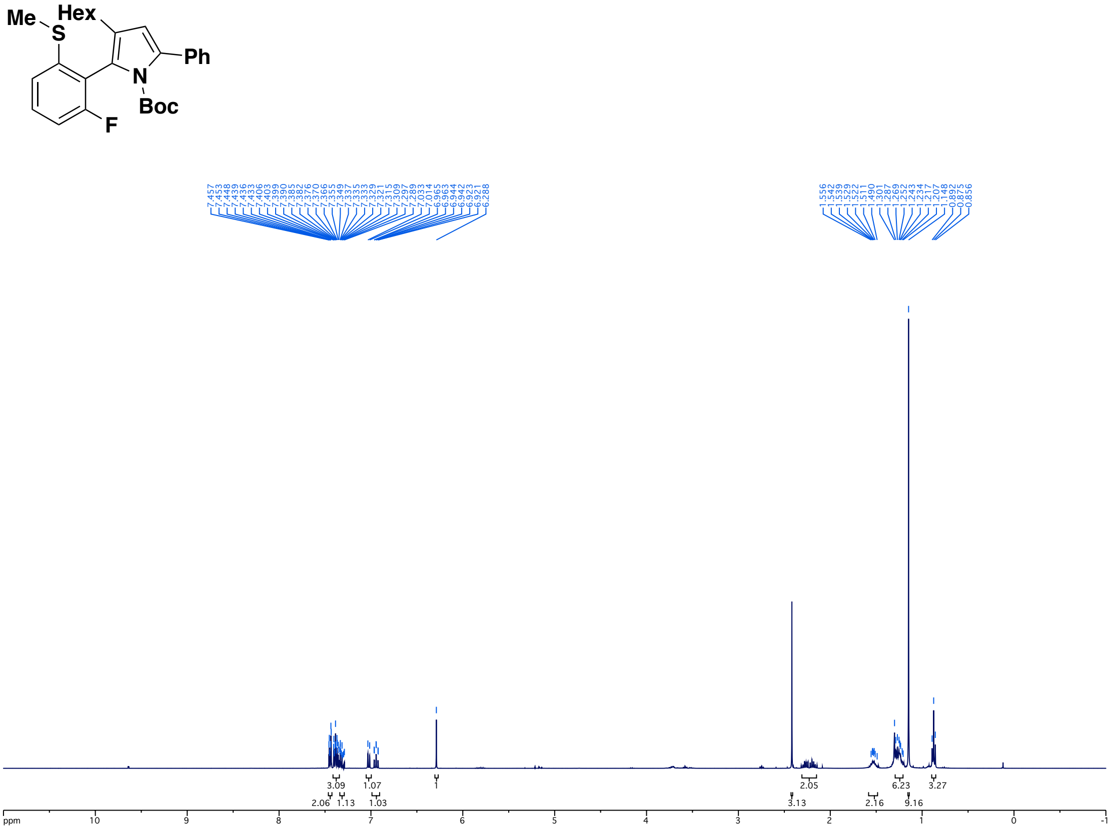

Pyrrole 4l: ^{13}C NMR (101 MHz, $CDCl_3$)

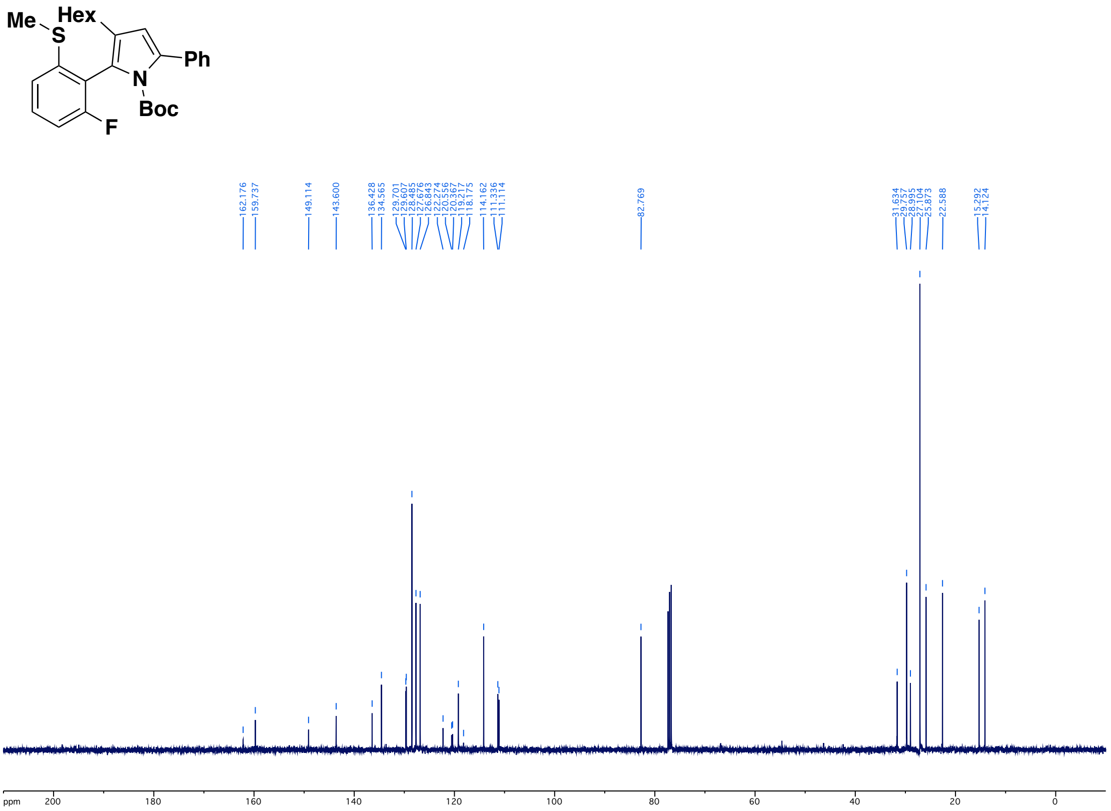

Pyrrole 4l: ^{19}F NMR (377 MHz, $CDCl_3$)

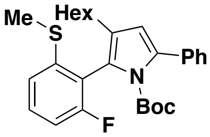

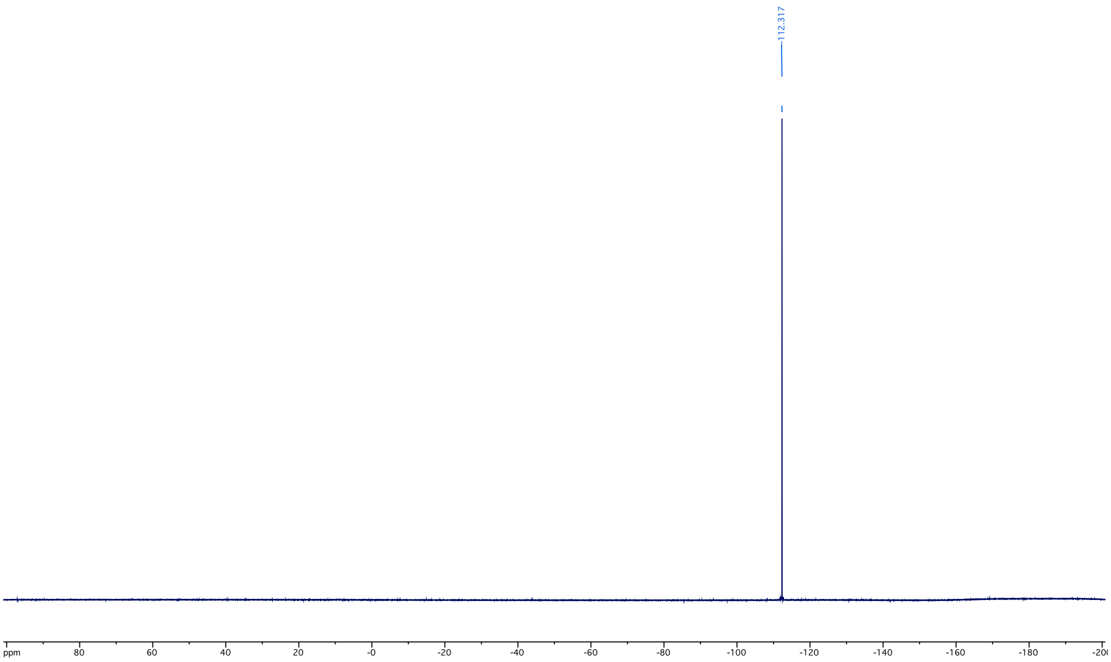

Pyrrole 4m: ^{1}H NMR (400 MHz, $CDCl_3$)

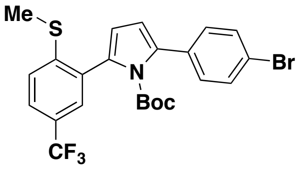

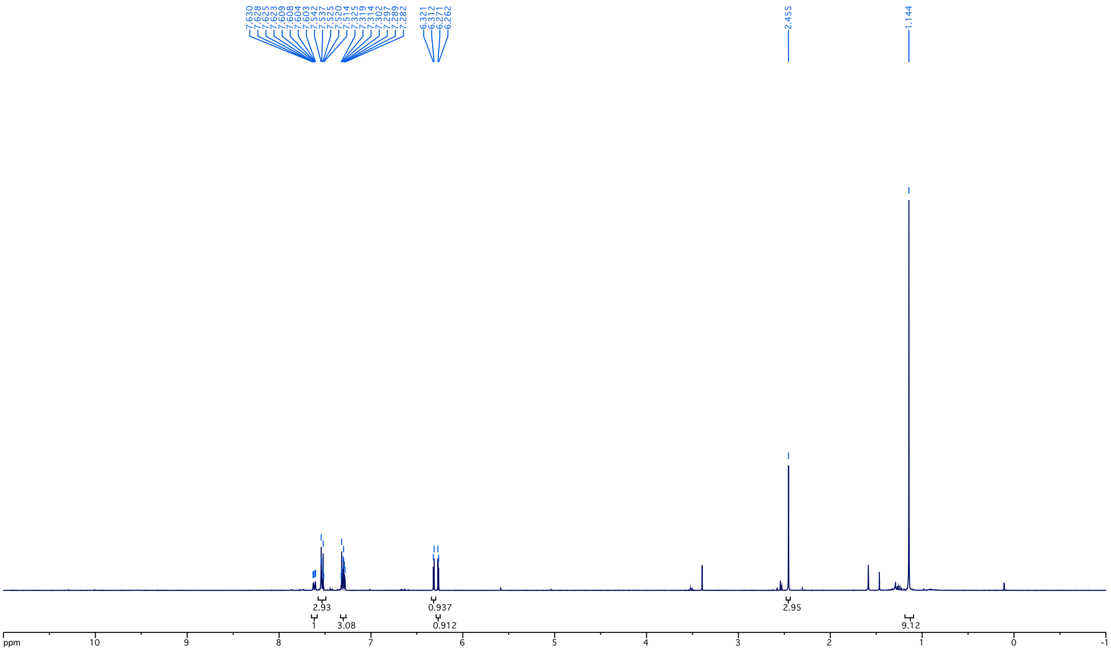

Pyrrole 4m: ^{13}C NMR (101 MHz, $CDCl_3$)

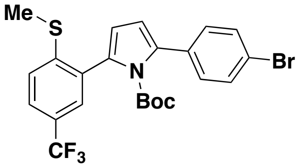

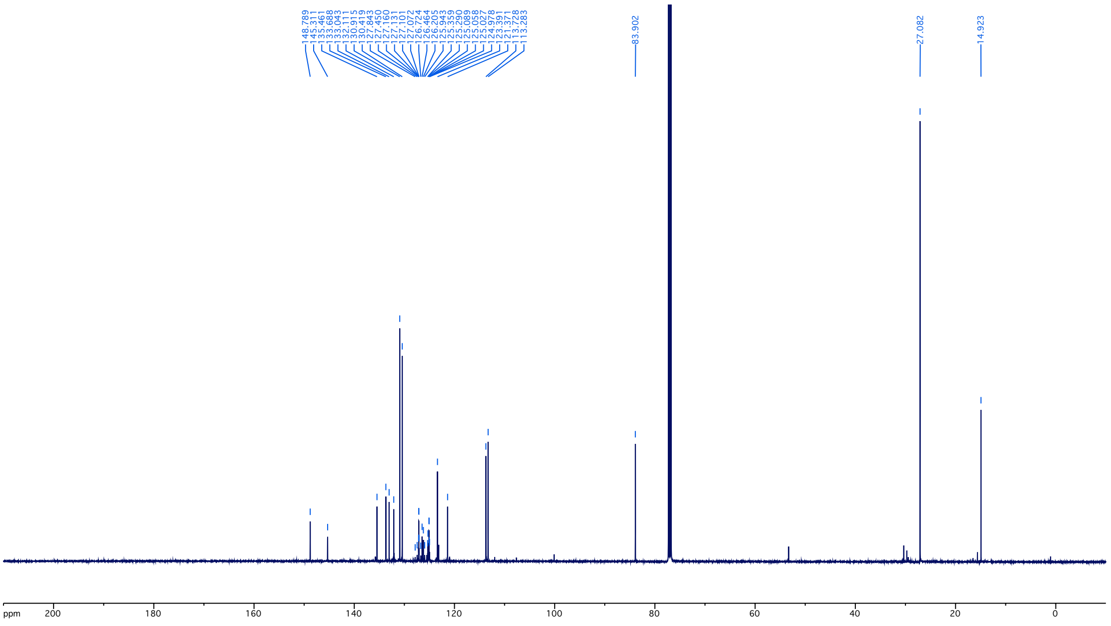

Pyrrole 4m: ^{19}F NMR (377 MHz, $CDCl_3$)

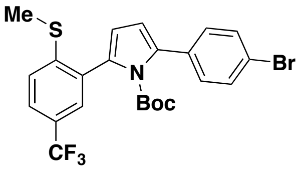

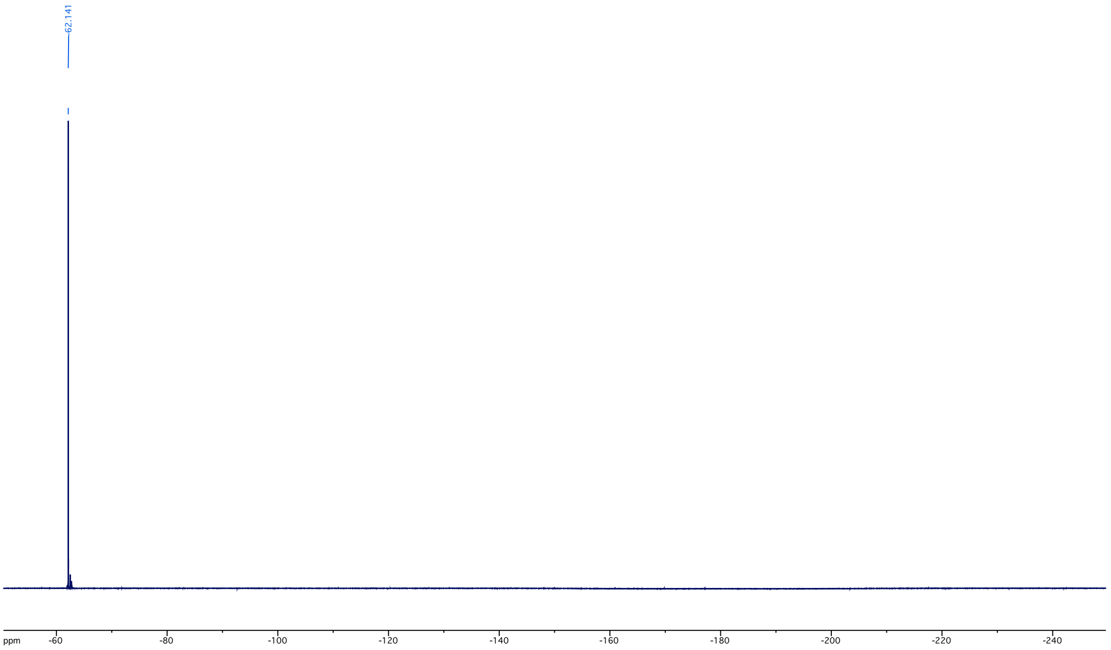

Pyrrole 4n: ^{1}H NMR (400 MHz, $CDCl_3$)

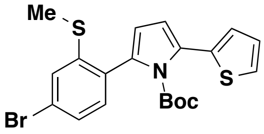

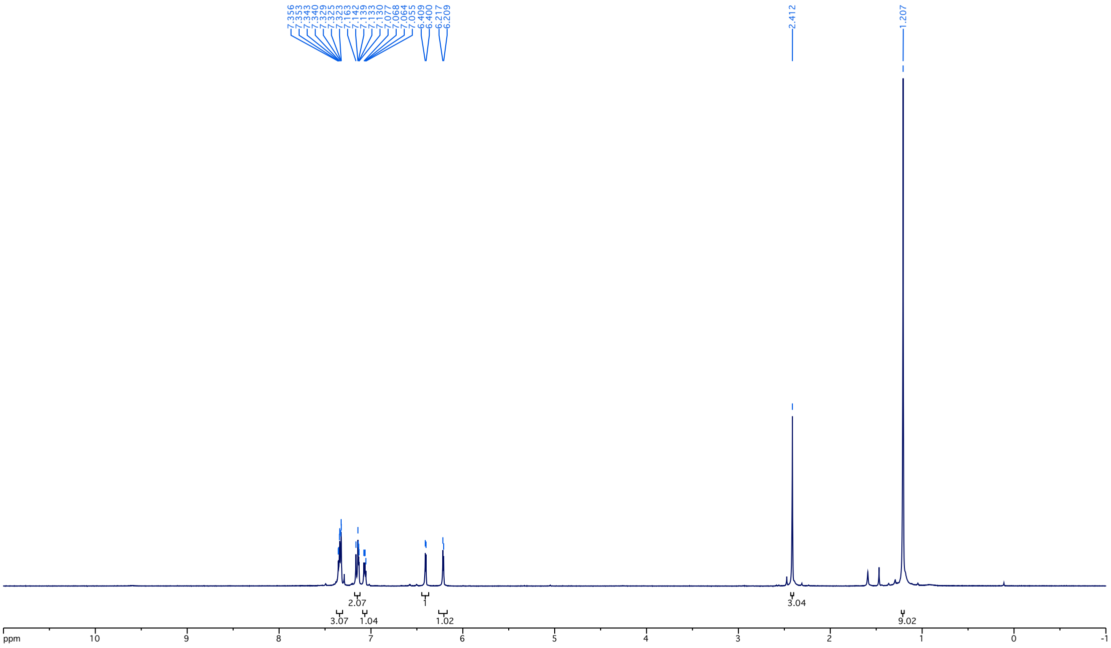

Pyrrole 4n: ^{13}C NMR (101 MHz, $CDCl_3$)

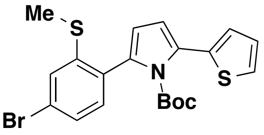

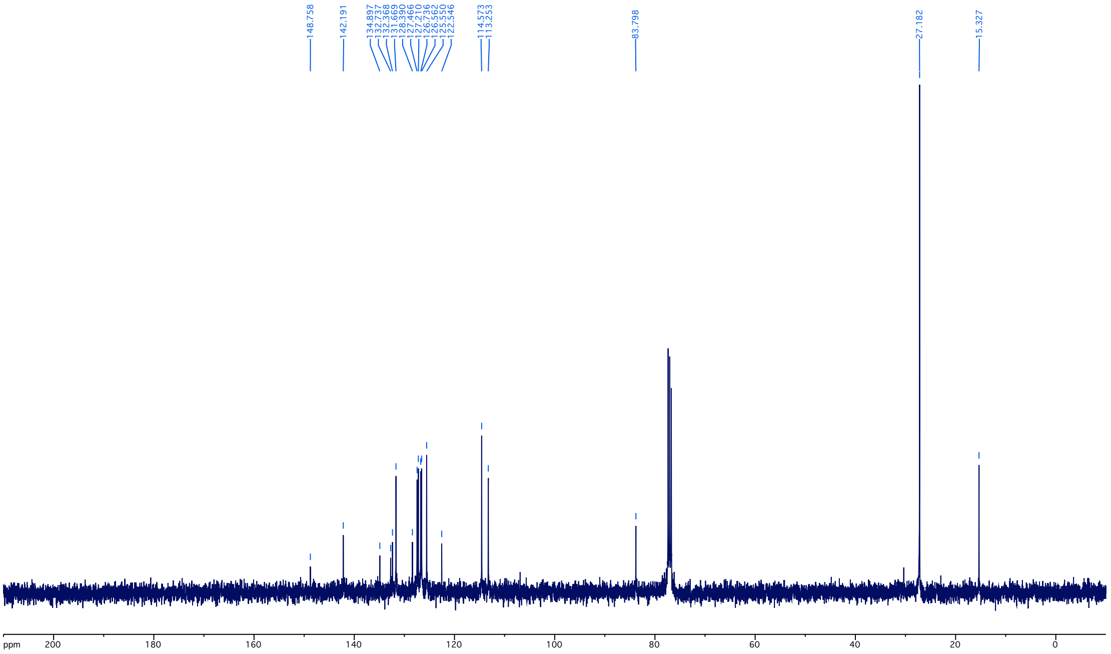

Pyrrole 4o: ^{1}H NMR (400 MHz, $CDCl_3$)

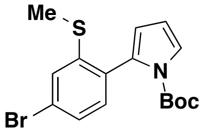

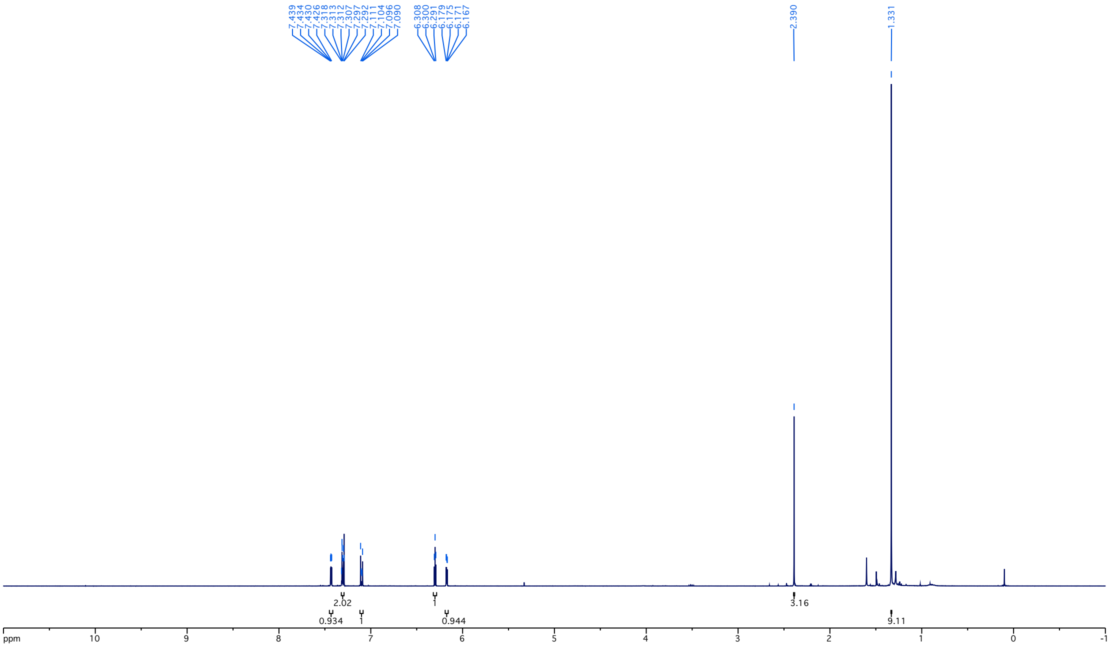

Pyrrole 4o: ^{13}C NMR (101 MHz, $CDCl_3$)

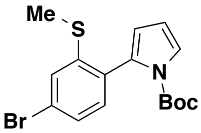

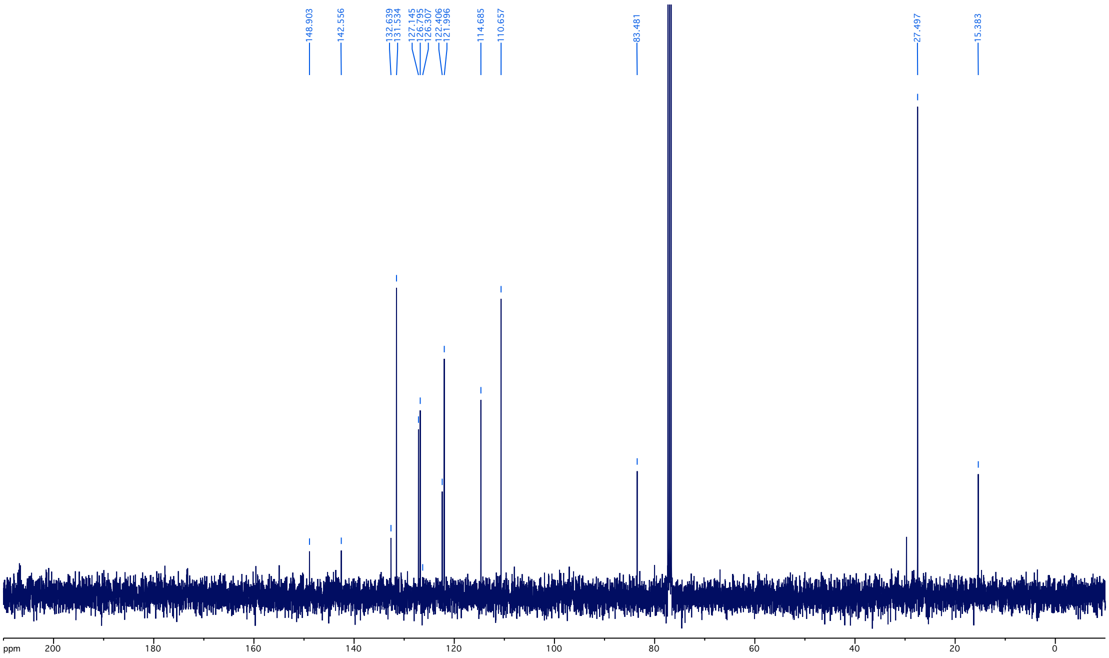

Pyrrole 4p: ^{1}H NMR (400 MHz, $CDCl_3$)

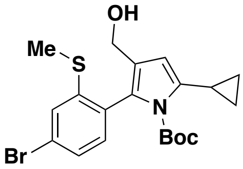

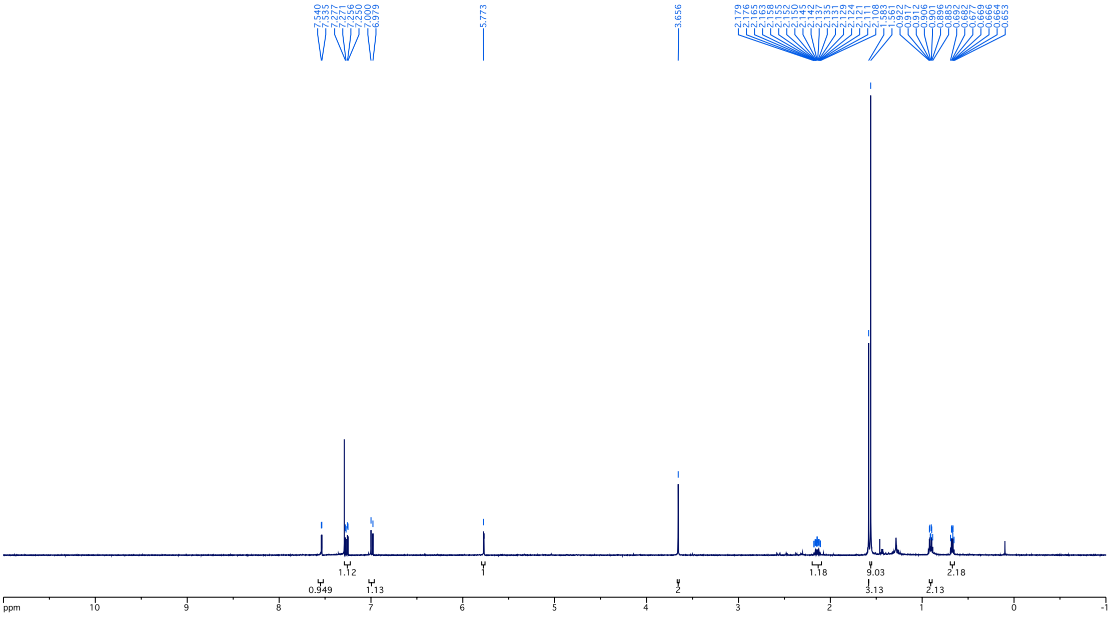

Pyrrole 4p: ^{13}C NMR (101 MHz, $CDCl_3$)

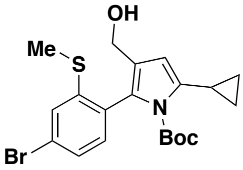

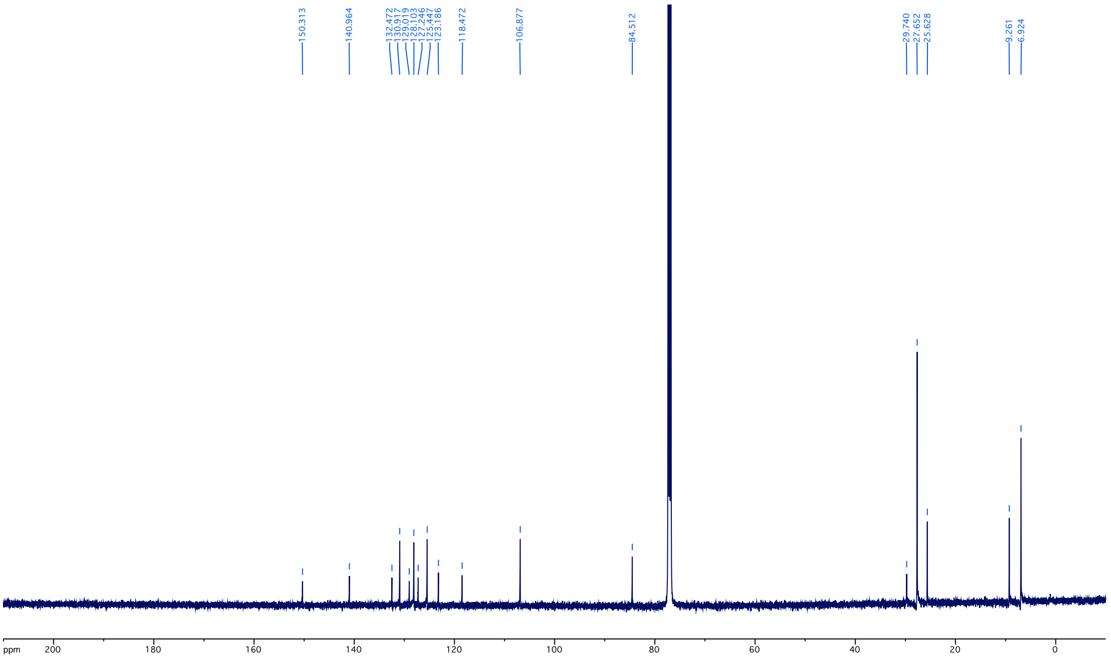

Pyrrole 4q: ^{1}H NMR (400 MHz, $CDCl_3$)

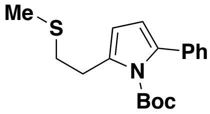

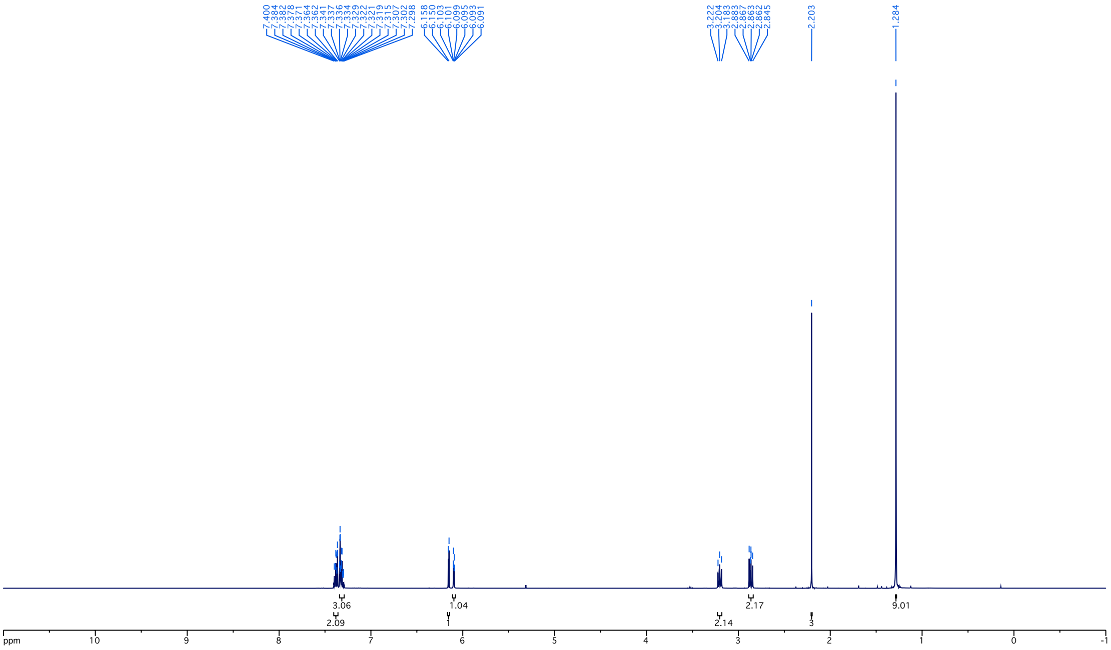

Pyrrole 4q: ^{13}C NMR (101 MHz, $CDCl_3$)

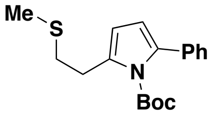

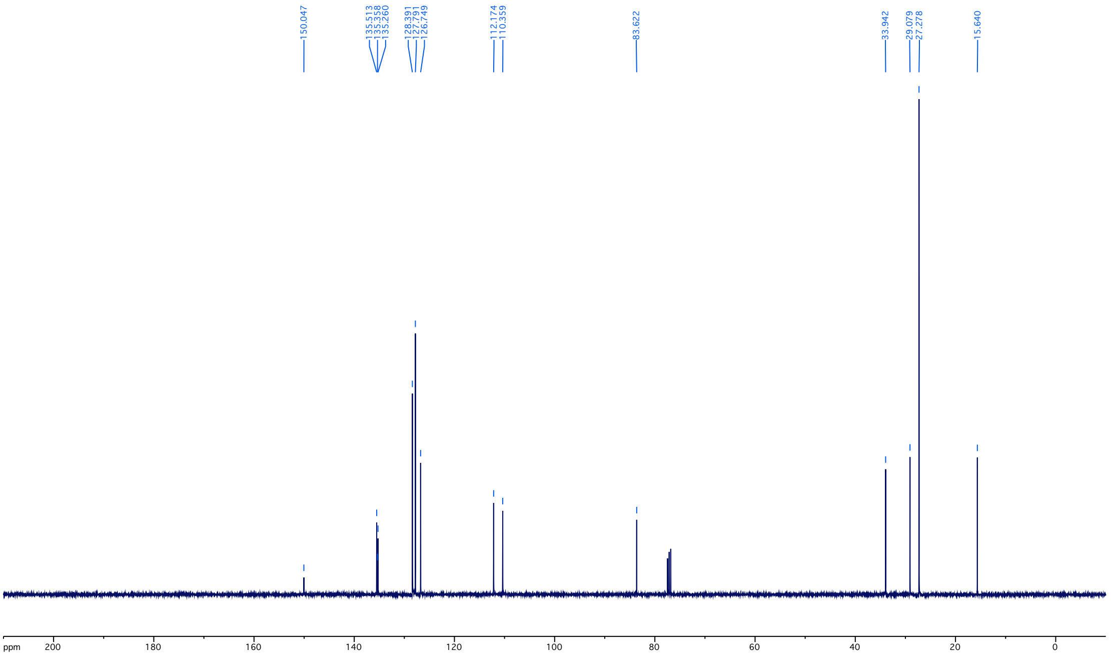

Pyrrole 4r: ^{1}H NMR (400 MHz, $CDCl_3$)

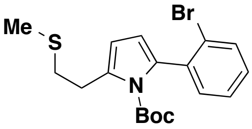

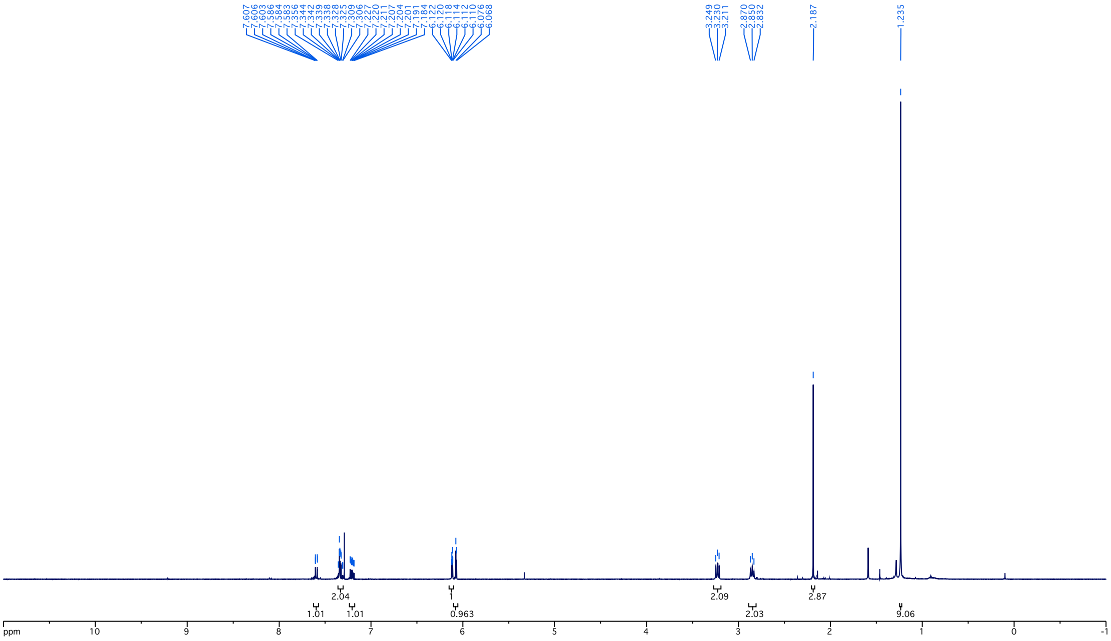

Pyrrole 4r: ^{13}C NMR (101 MHz, $CDCl_3$)

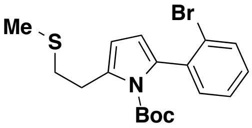

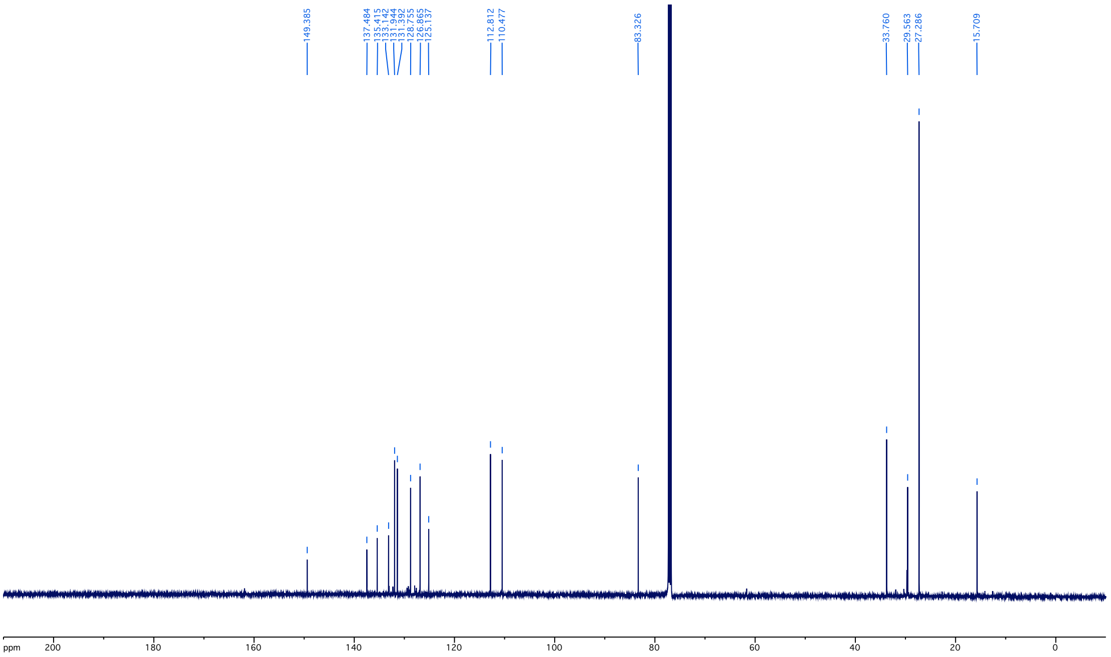

Pyrrole 4s: ^1H NMR (400 MHz, $CDCl_3$)

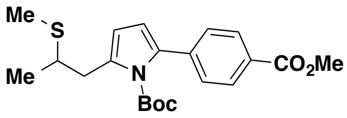

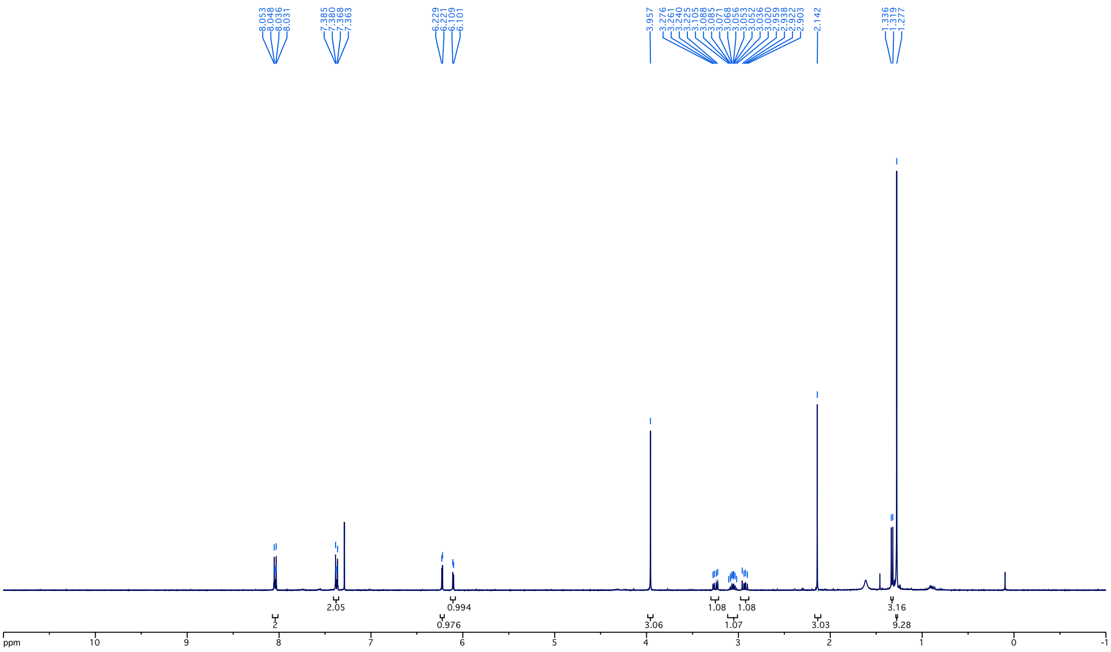

Pyrrole 4s: ^{13}C NMR (101 MHz, $CDCl_3$)

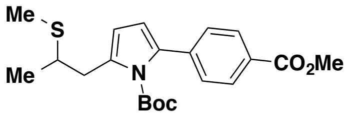

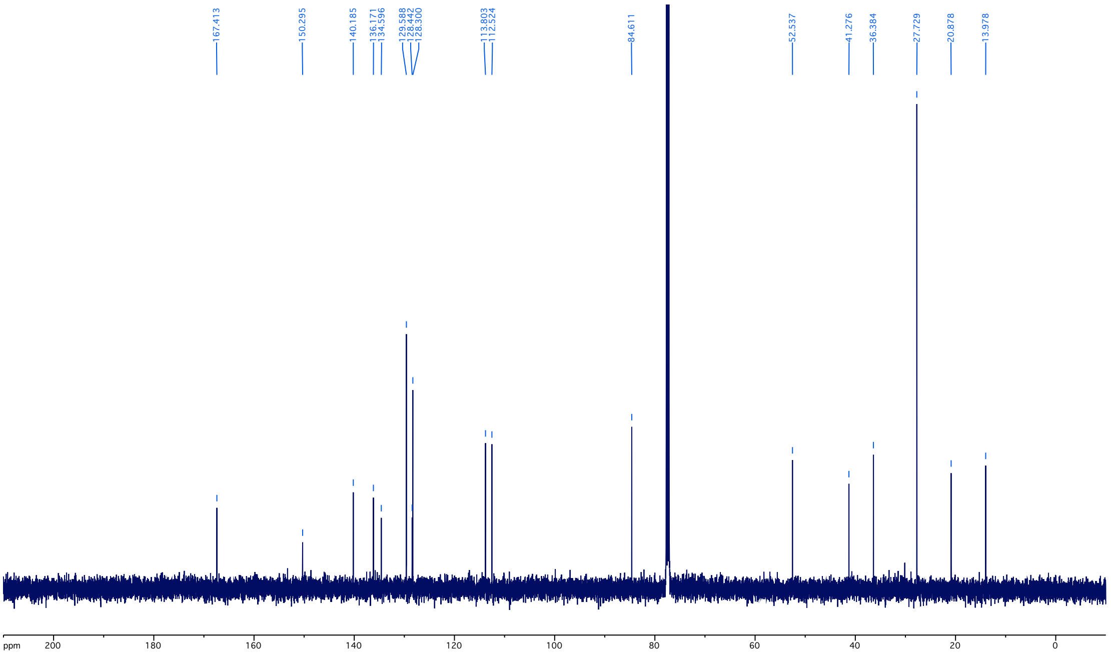

Pyrrole 4t: ^{1}H NMR (400 MHz, $CDCl_3$)

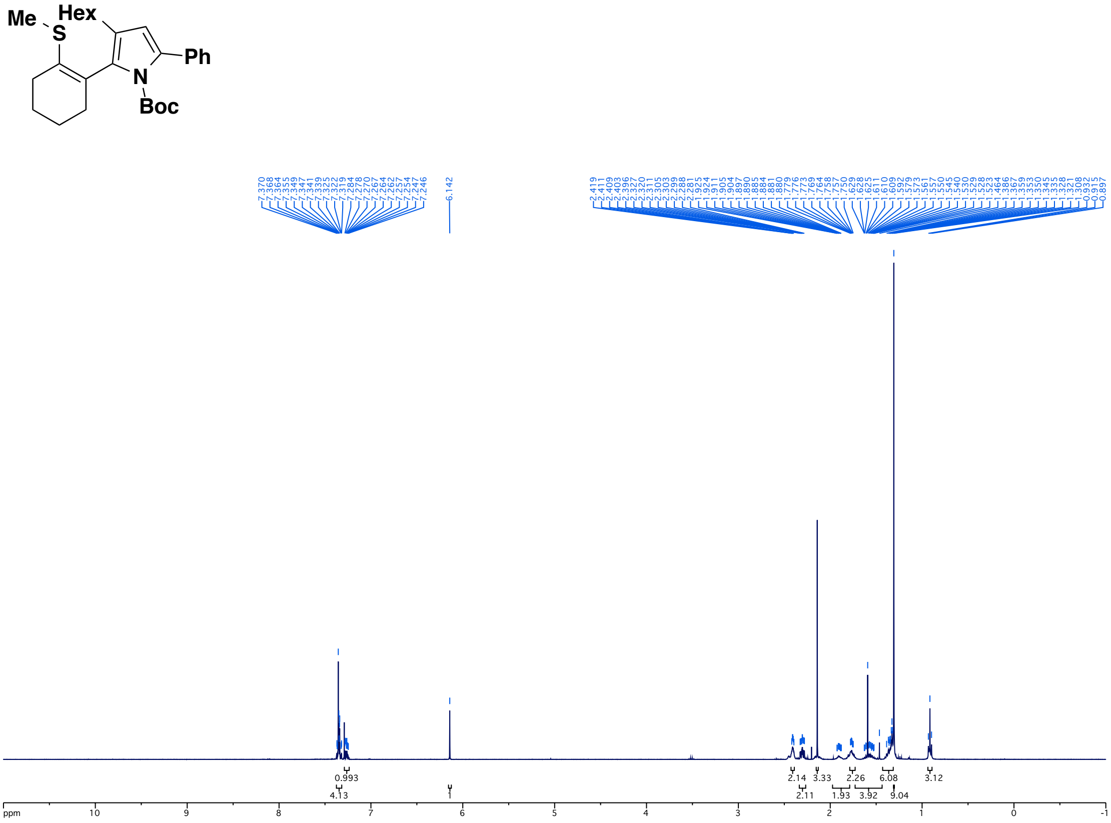

Pyrrole 4t: ^{13}C NMR (101 MHz, $CDCl_3$)

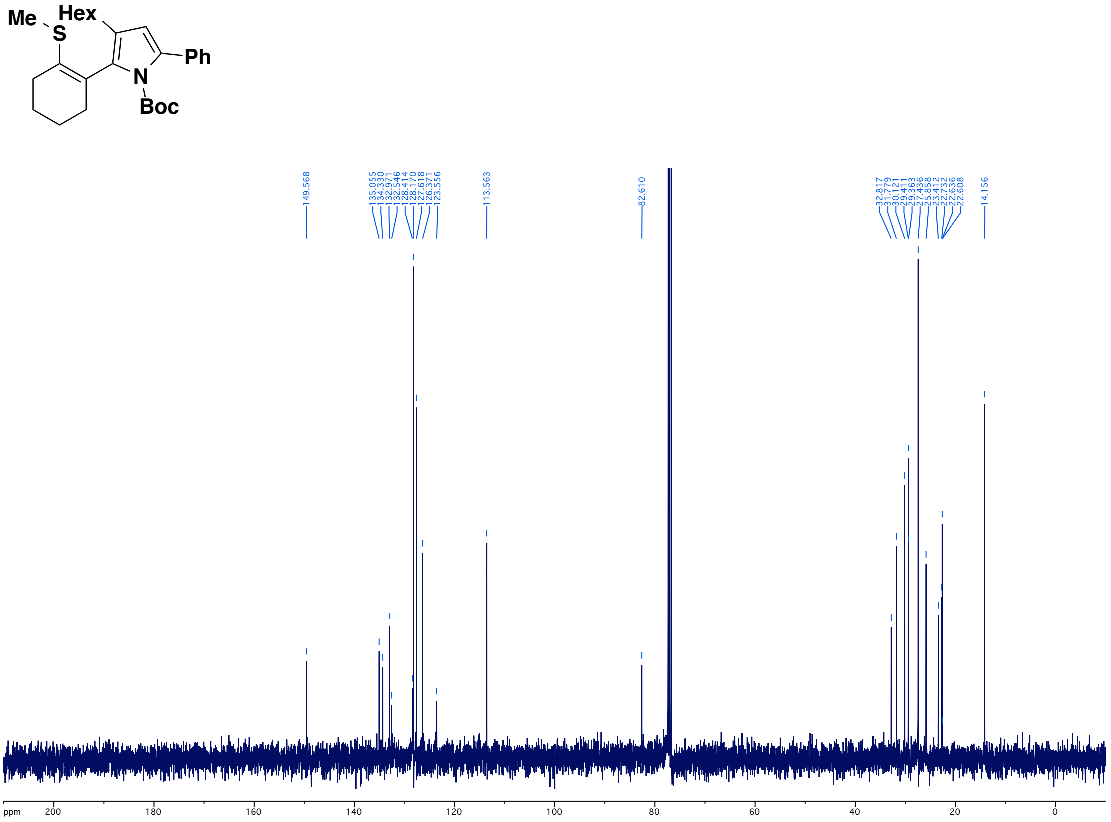

Pyrrole 4u: ^{1}H NMR (400 MHz, $CDCl_3$)

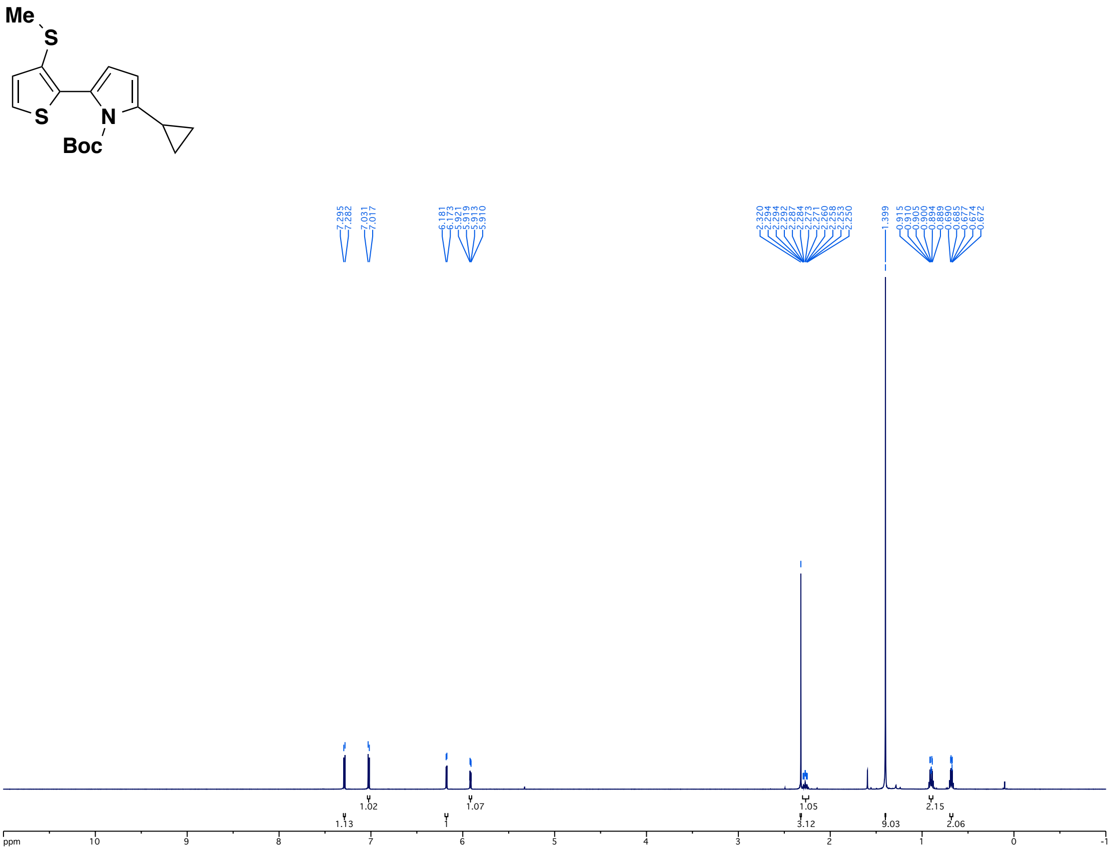

Pyrrole 4u: ^{13}C NMR (101 MHz, $CDCl_3$)

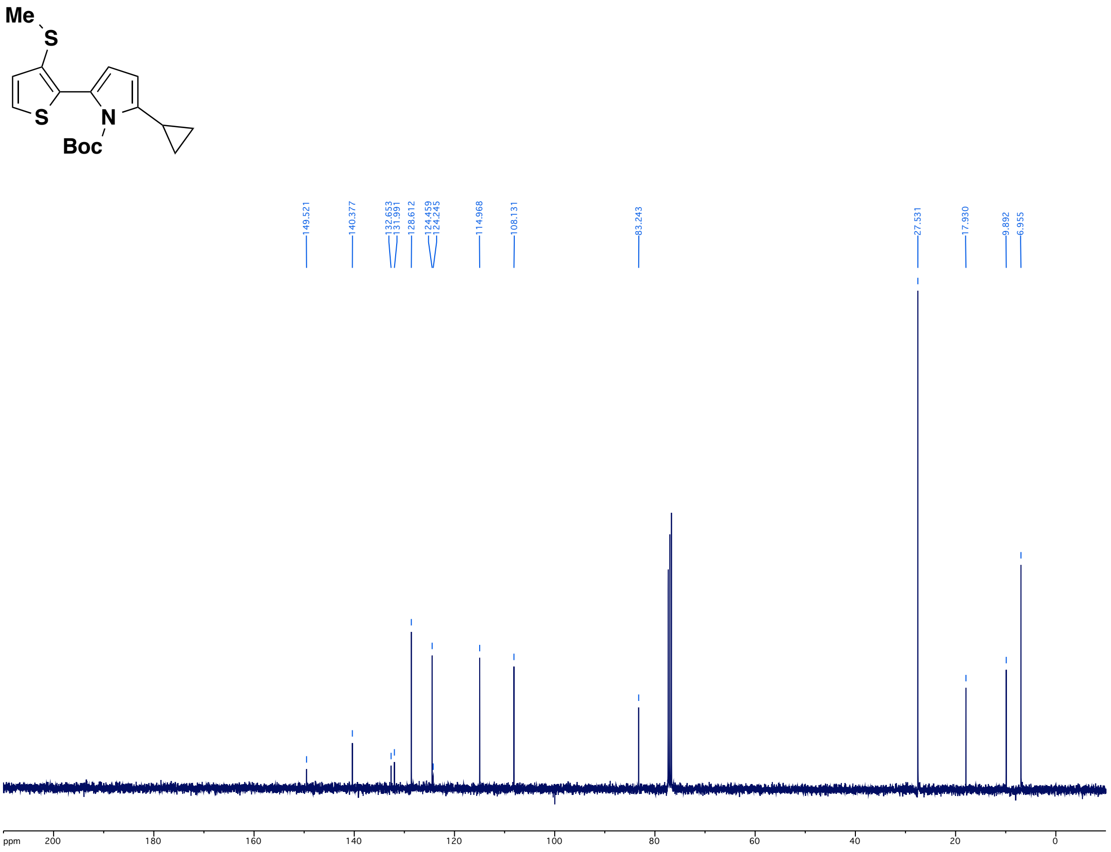

Pyrrole 4v: ^{1}H NMR (400 MHz, $CDCl_3$)

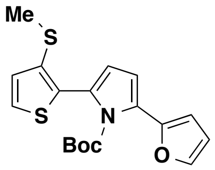

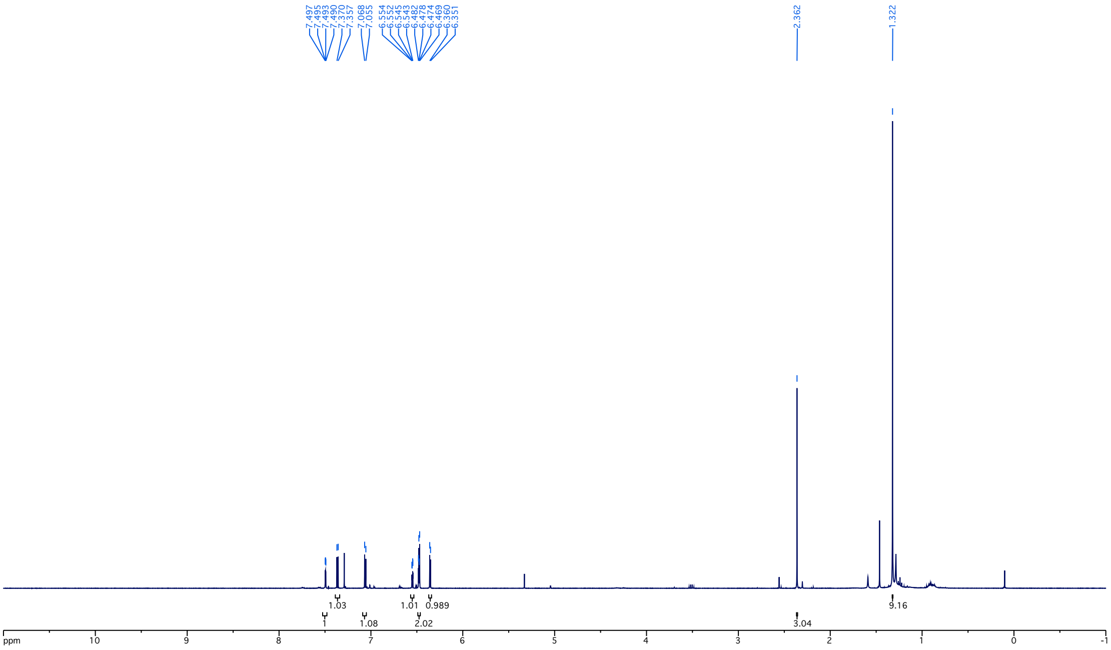

Pyrrole 4v: ^{13}C NMR (101 MHz, $CDCl_3$)

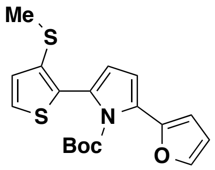

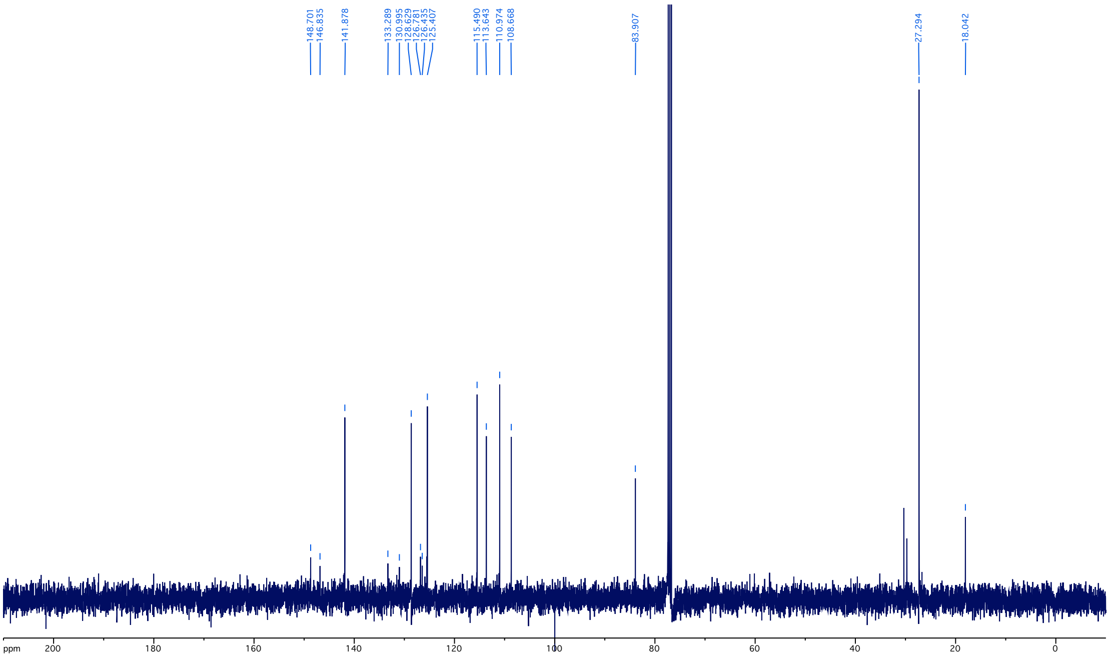

Pyrrole 4w: ^{1}H NMR (400 MHz, $CDCl_3$)

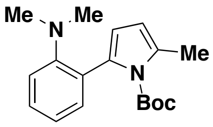

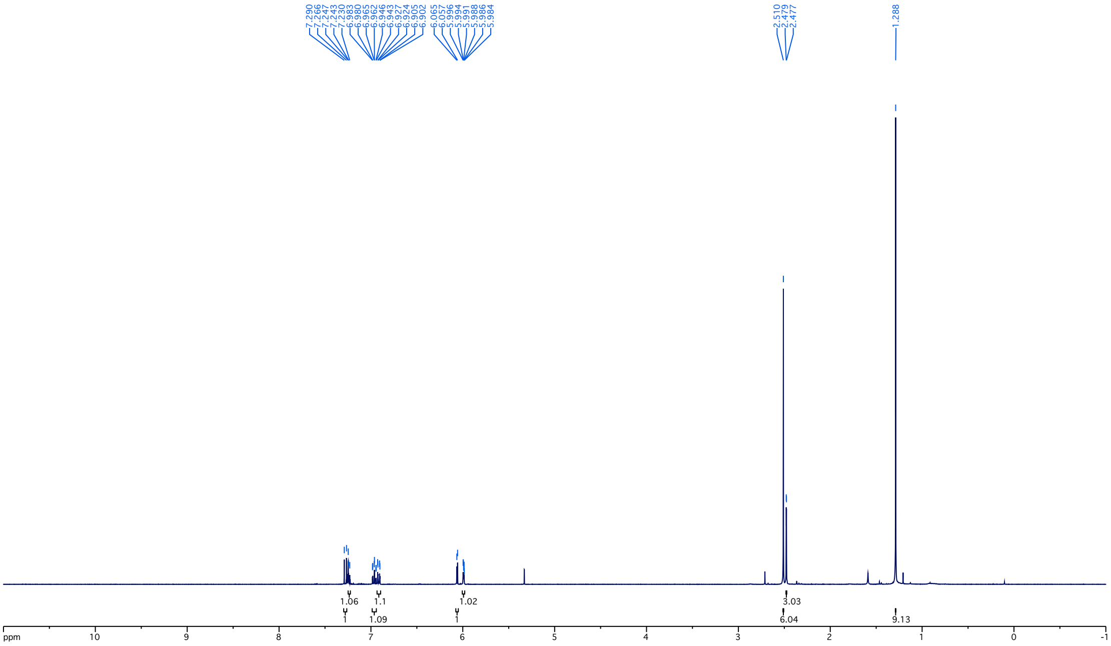

Pyrrole 4w: ^{13}C NMR (101 MHz, $CDCl_3$)

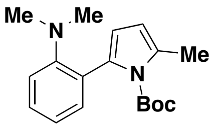

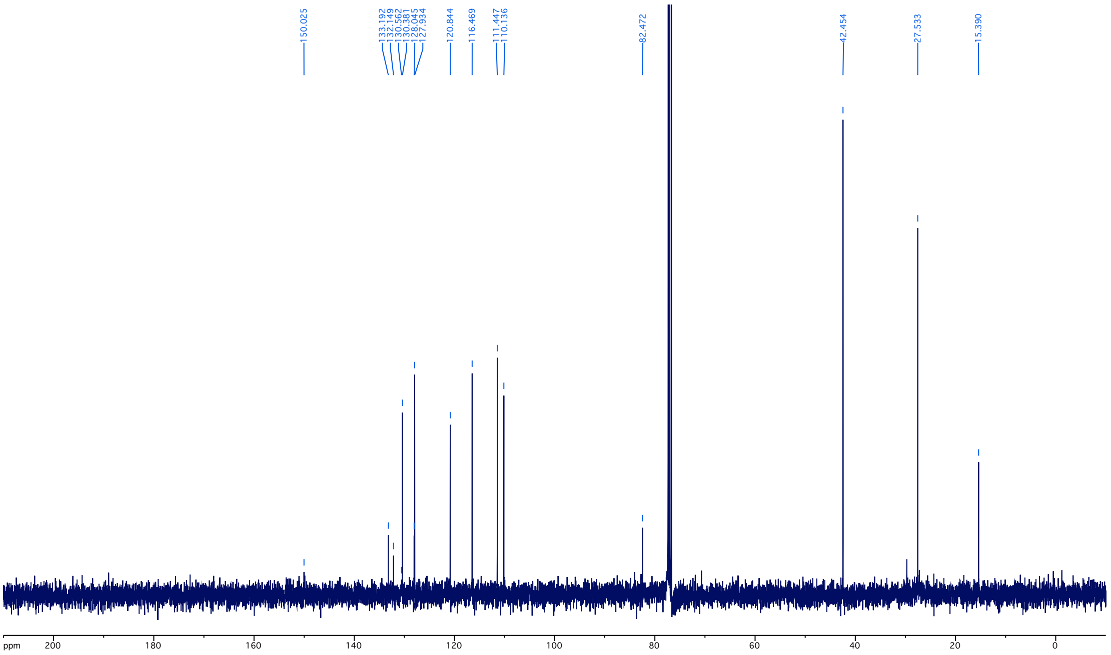

Pyrrole 4x: ^{1}H NMR (400 MHz, $CDCl_3$)

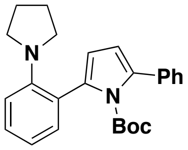

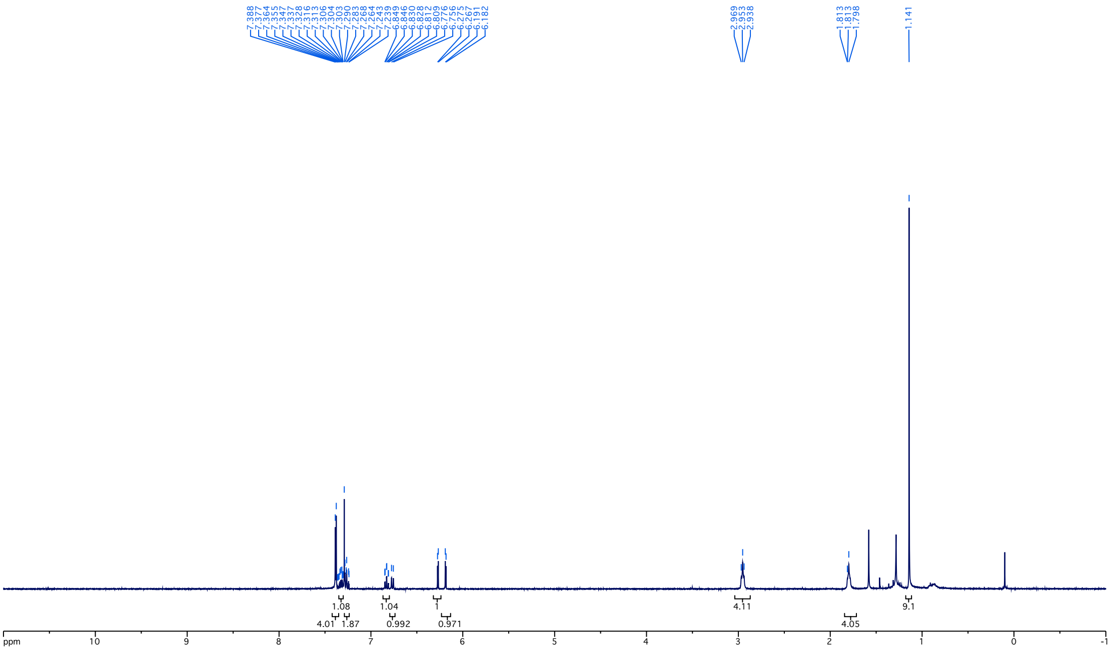

Pyrrole 4x: ^{13}C NMR (101 MHz, $CDCl_3$)

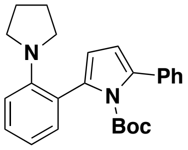

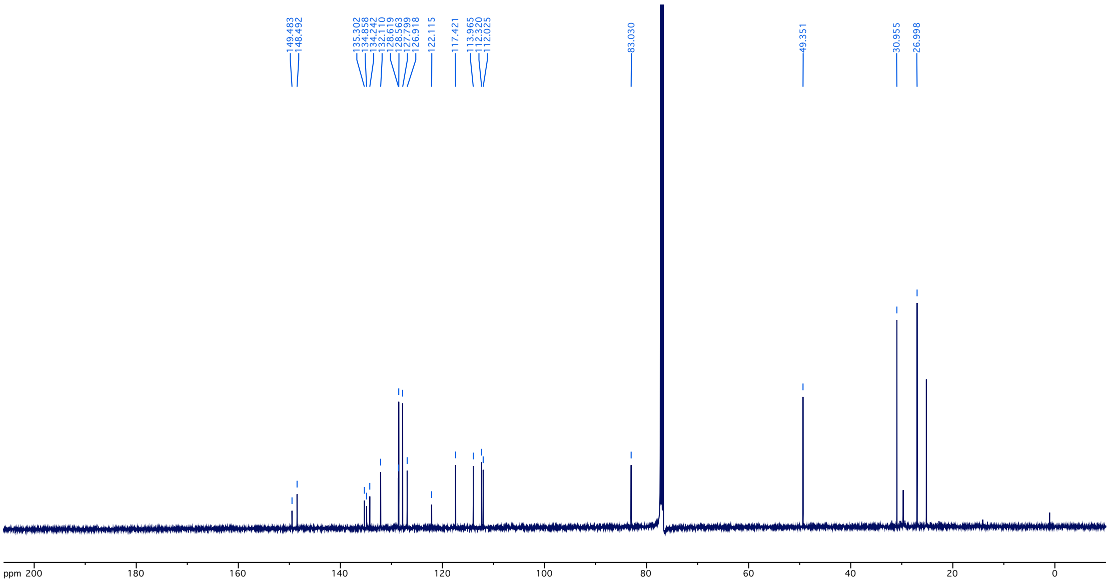

Pyrrole 5a: ¹H NMR (400 MHz, $CDCl_3$)

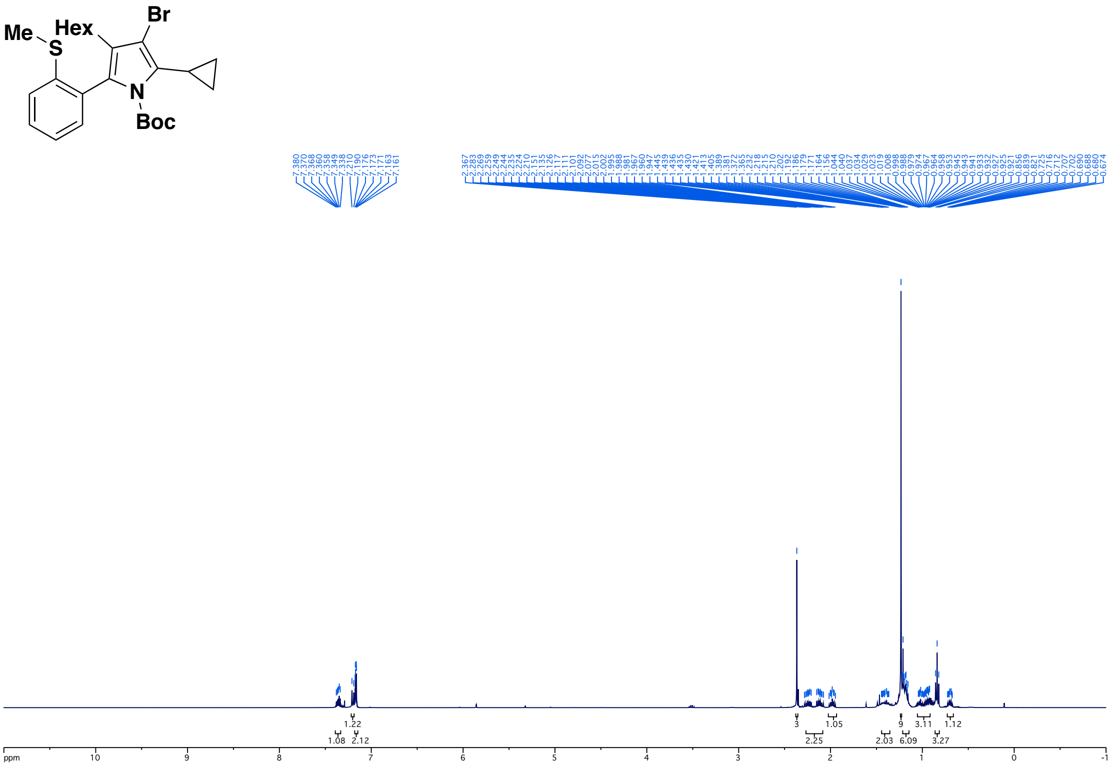

Pyrrole 5a: ¹³C NMR (101 MHz, $CDCl_3$)

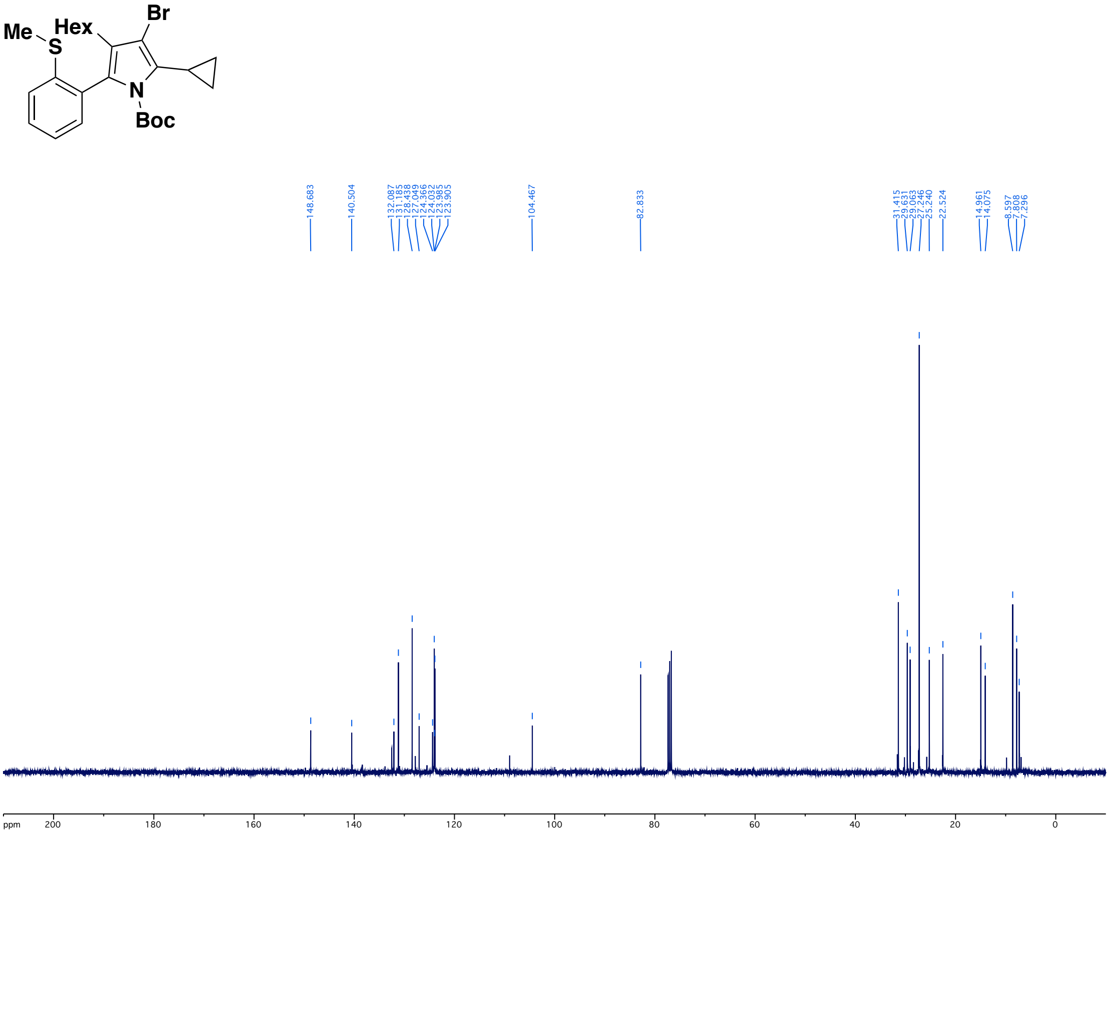

Pyrrole 5b: ^{1}H NMR (400 MHz, $CDCl_3$)

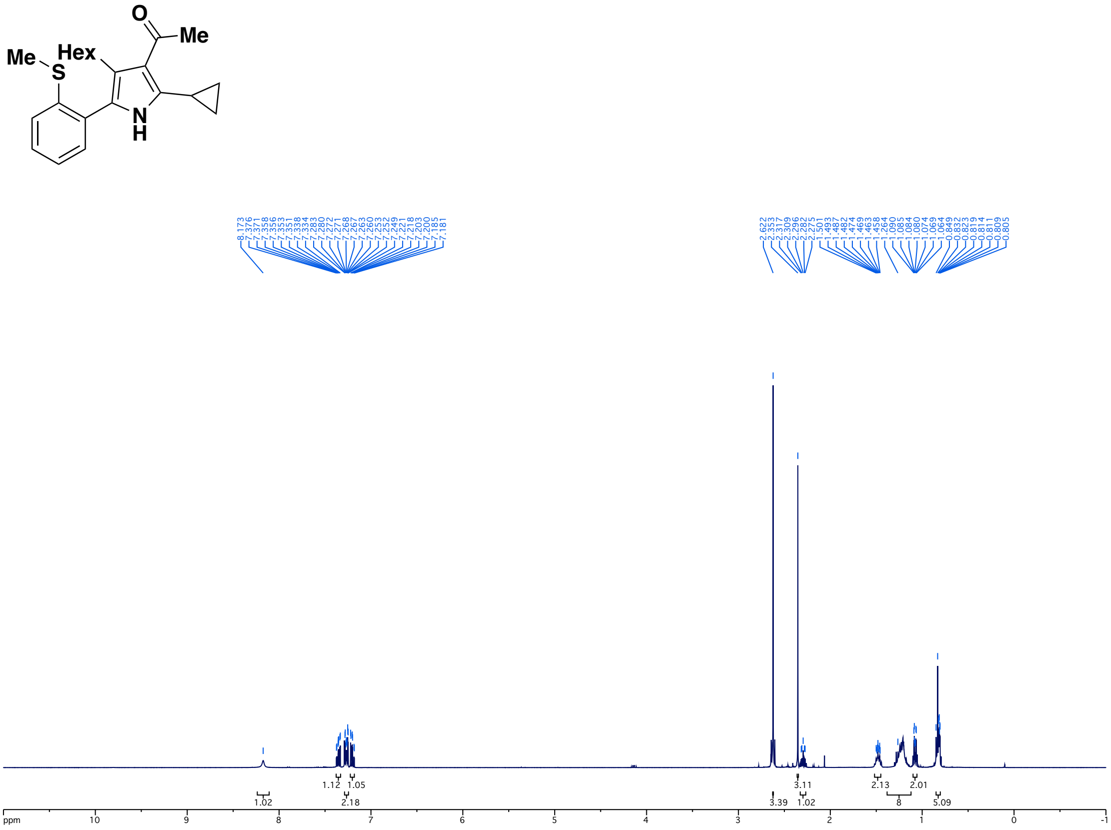

Pyrrole 5b: ^{13}C NMR (101 MHz, $CDCl_3$)

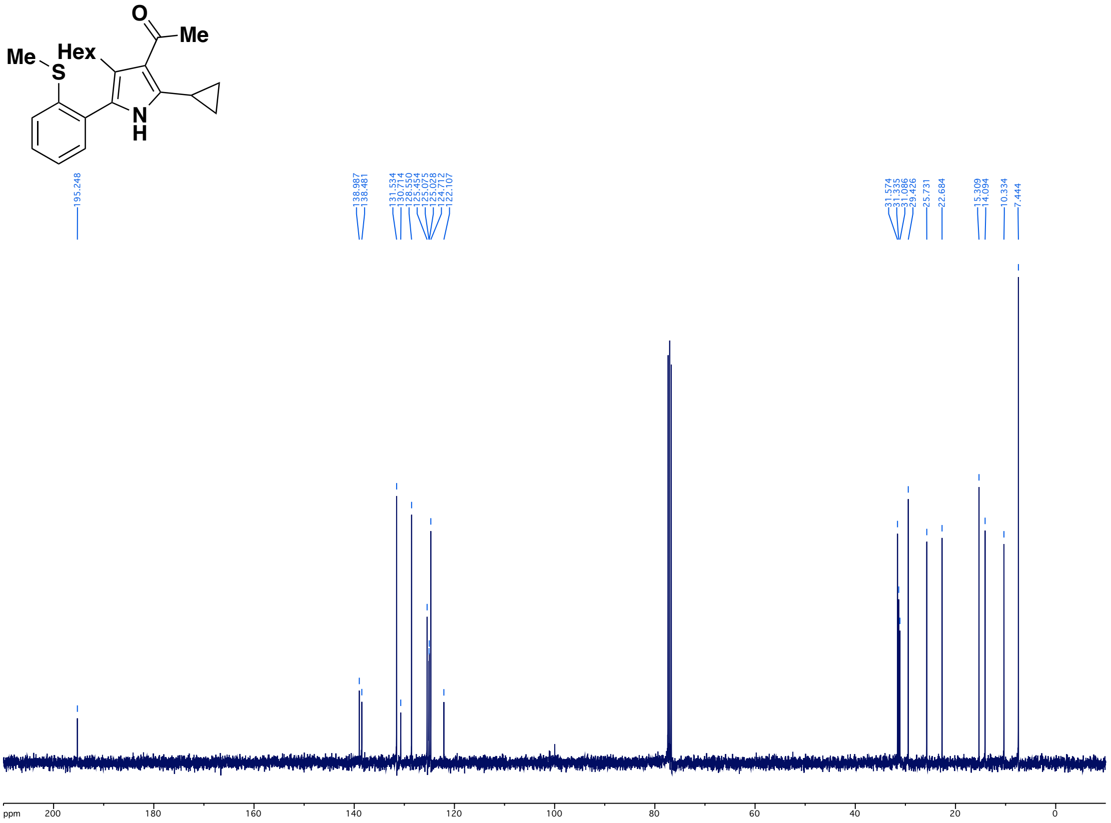

Pyrrole 5c: ^{1}H NMR (400 MHz, $CDCl_3$)

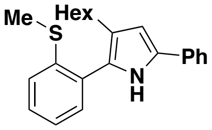

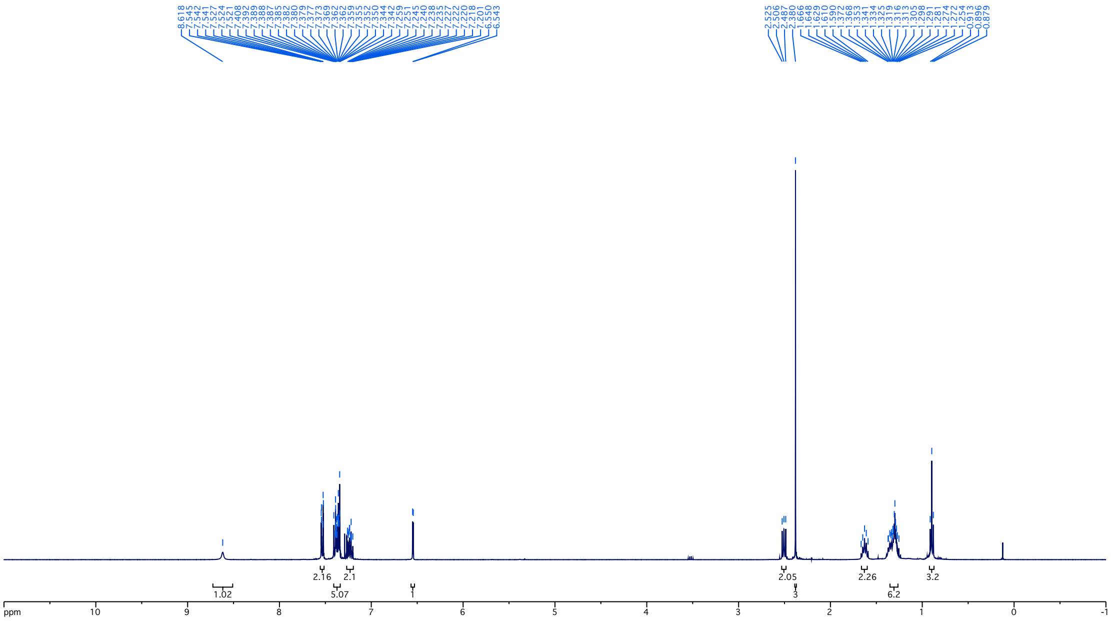

Pyrrole 5c: ^{13}C NMR (101 MHz, $CDCl_3$)

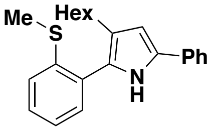

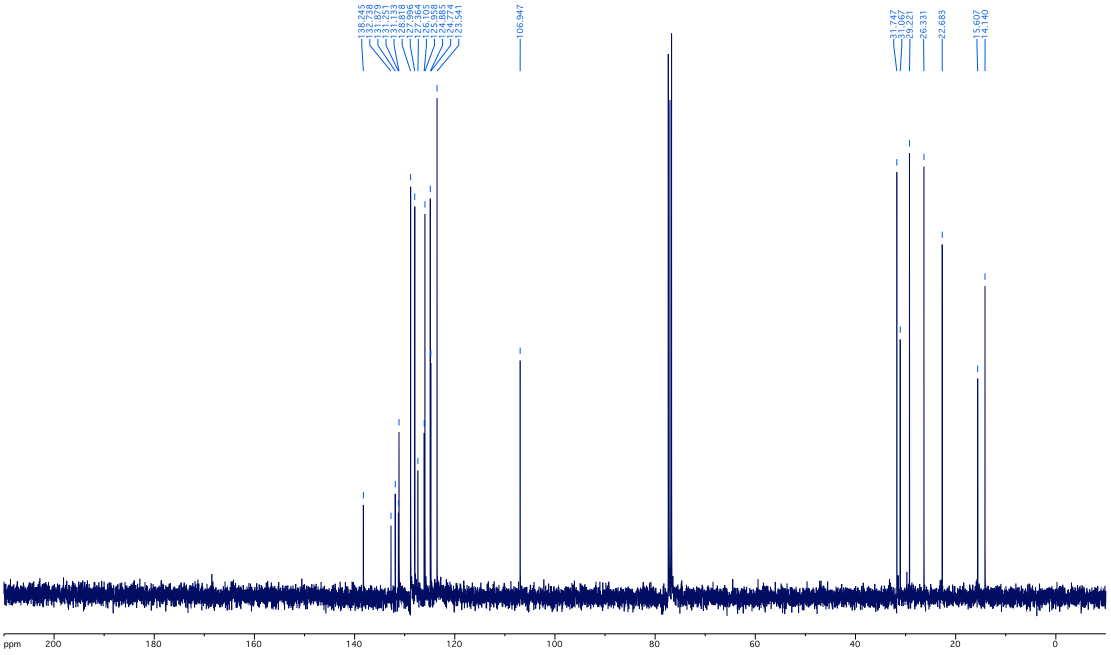

Pyrrole 6a: ^{1}H NMR (400 MHz, $CDCl_3$)

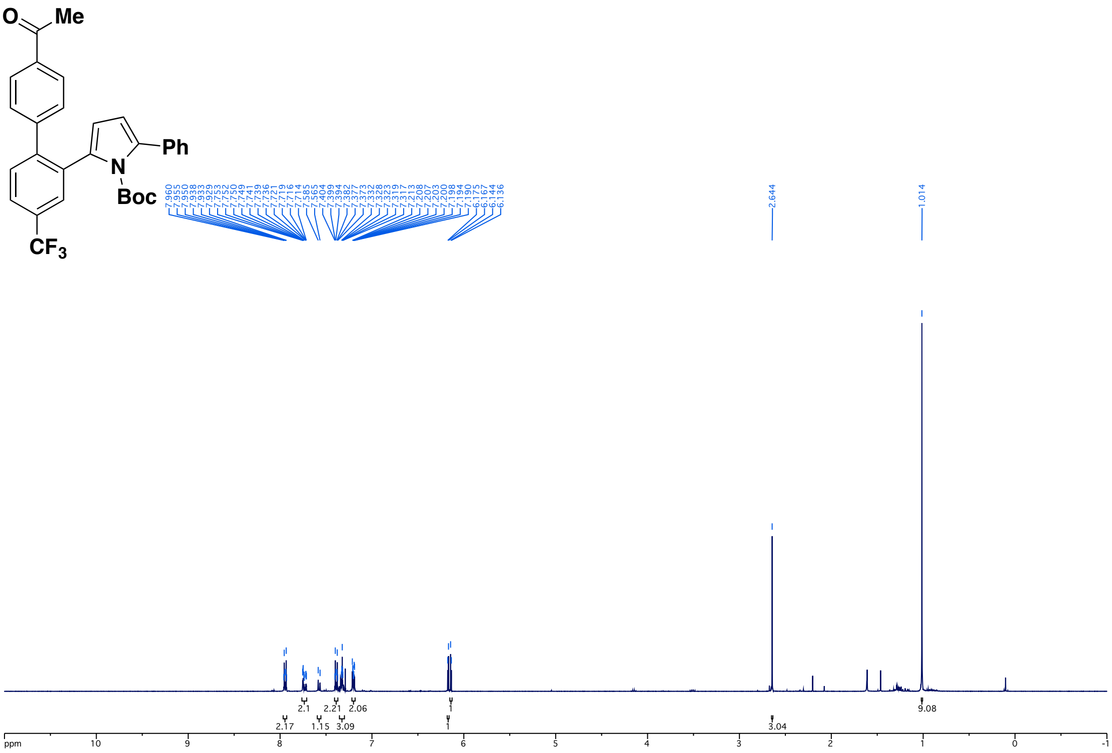

Pyrrole 6a: ^{13}C NMR (101 MHz, $CDCl_3$)

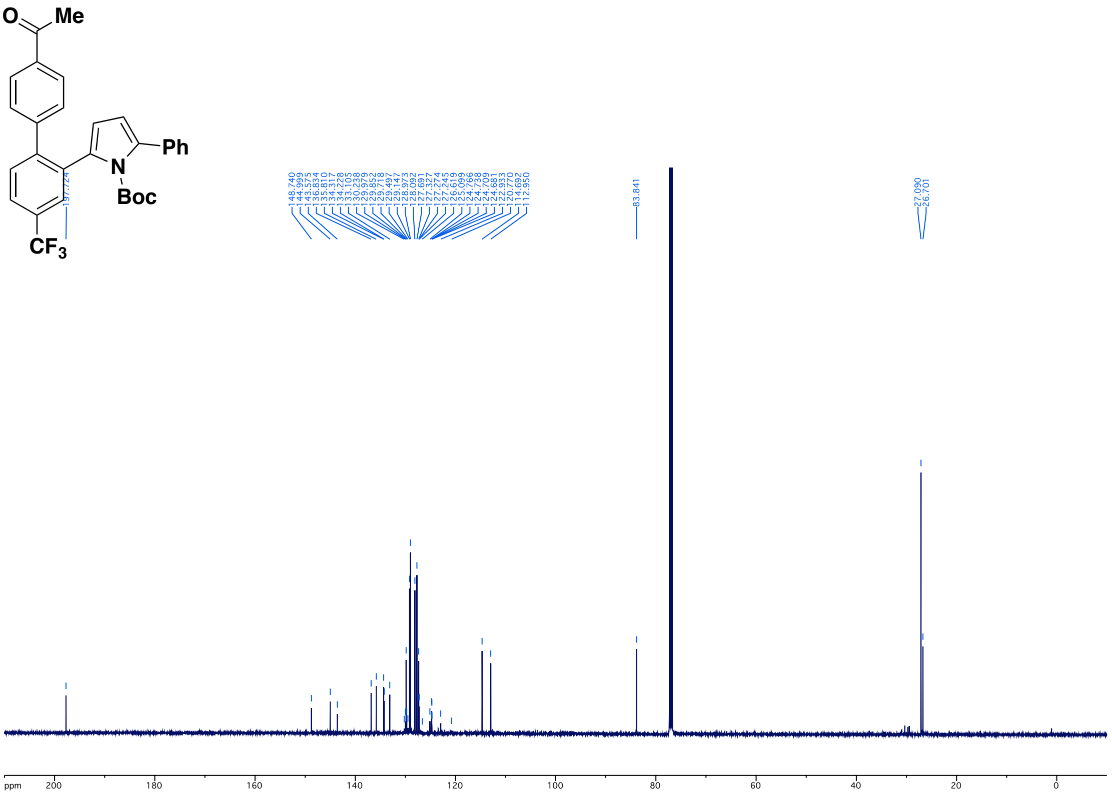

Pyrrole 6a: ^{19}F NMR (377 MHz, $CDCl_3$)

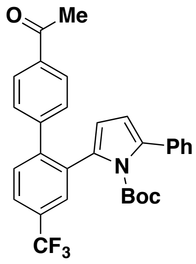

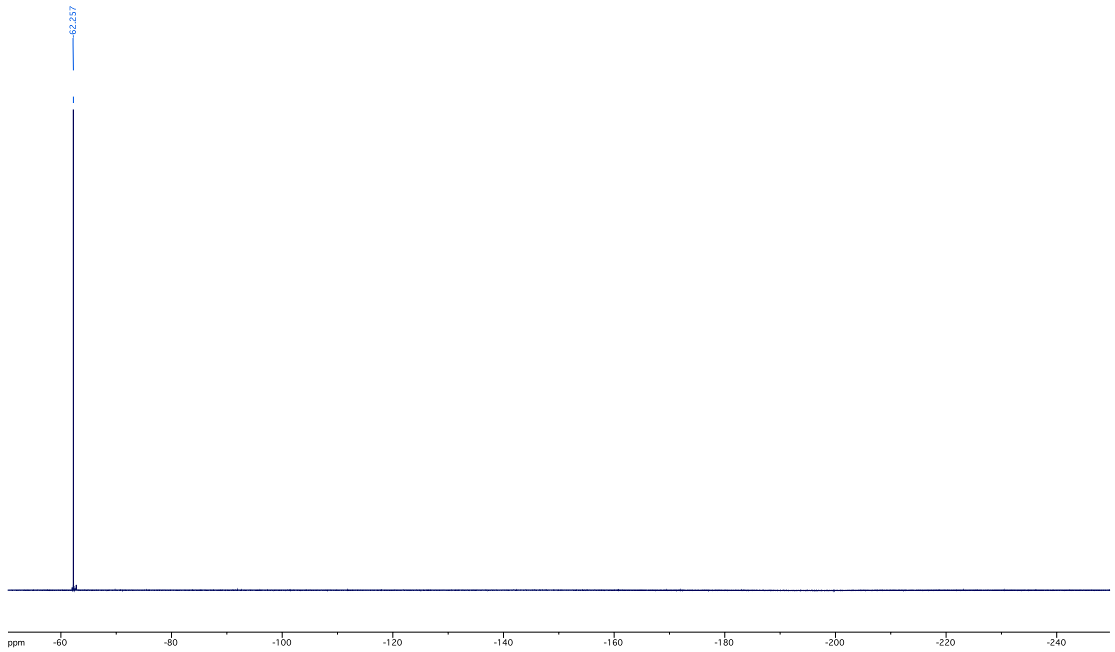

Pyrrole 6b: ^{1}H NMR (400 MHz, $CDCl_3$)

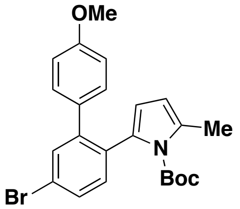

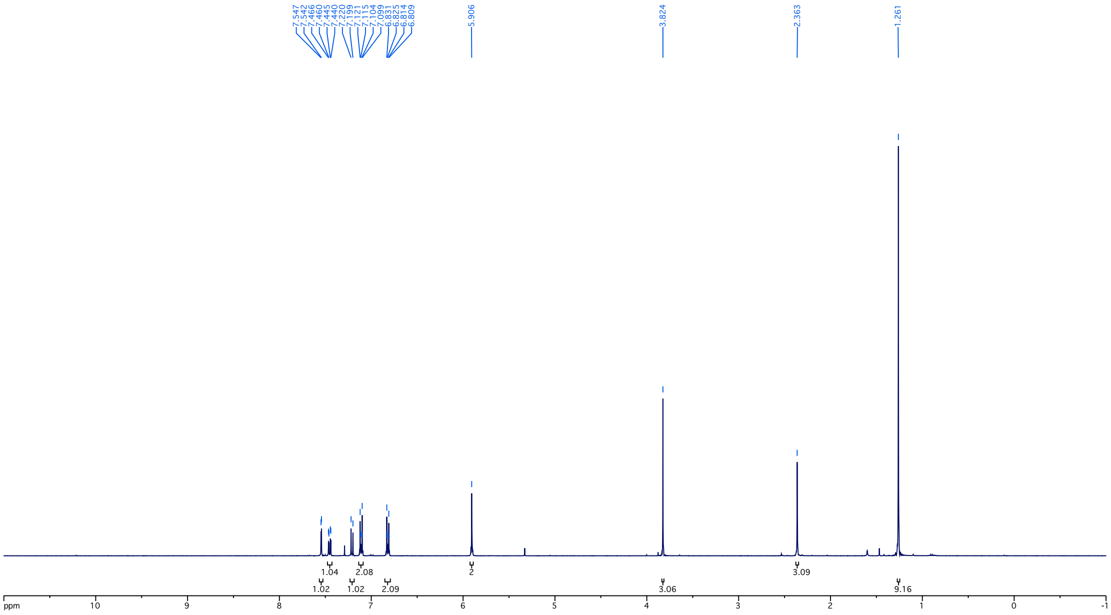

Pyrrole 6b: ^{13}C NMR (101 MHz, $CDCl_3$)

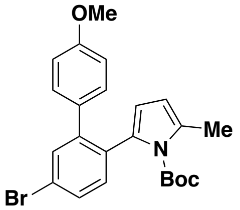

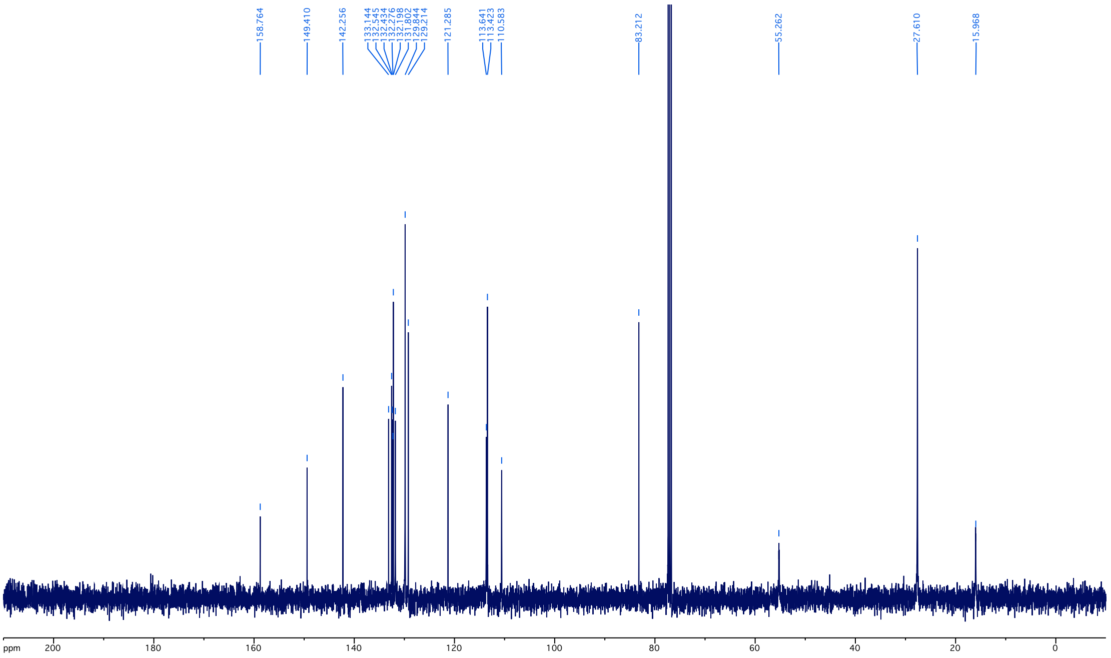

Pyrrole 6c: ^{1}H NMR (400 MHz, $CDCl_3$)

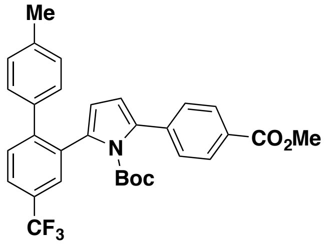

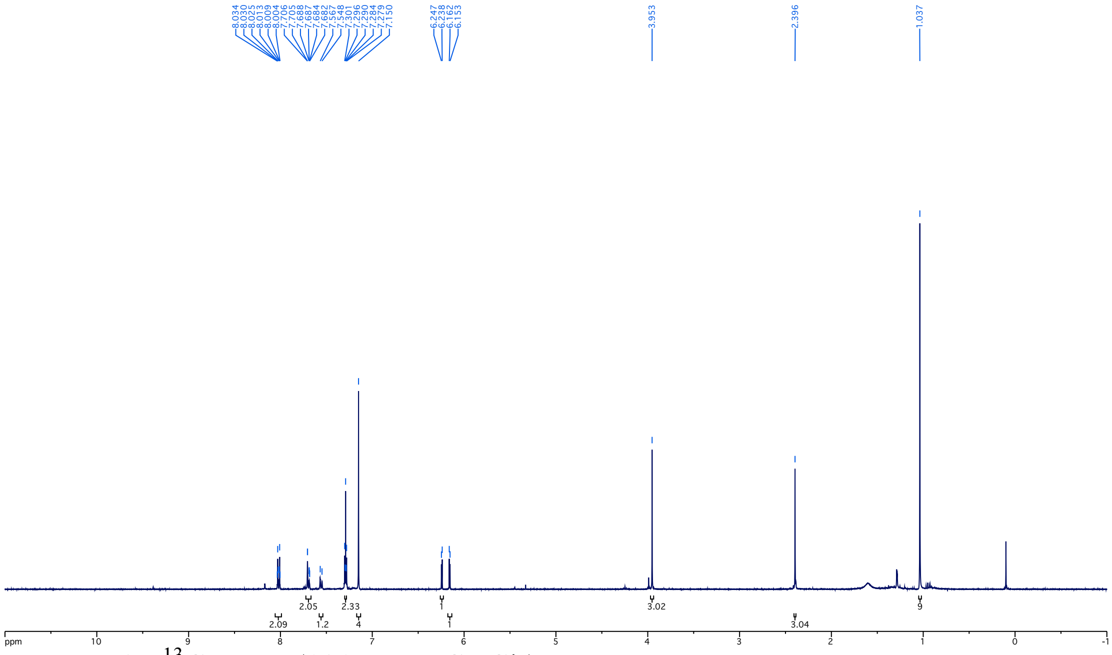

Pyrrole 6c: ^{13}C NMR (101 MHz, $CDCl_3$)

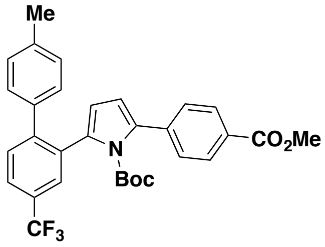

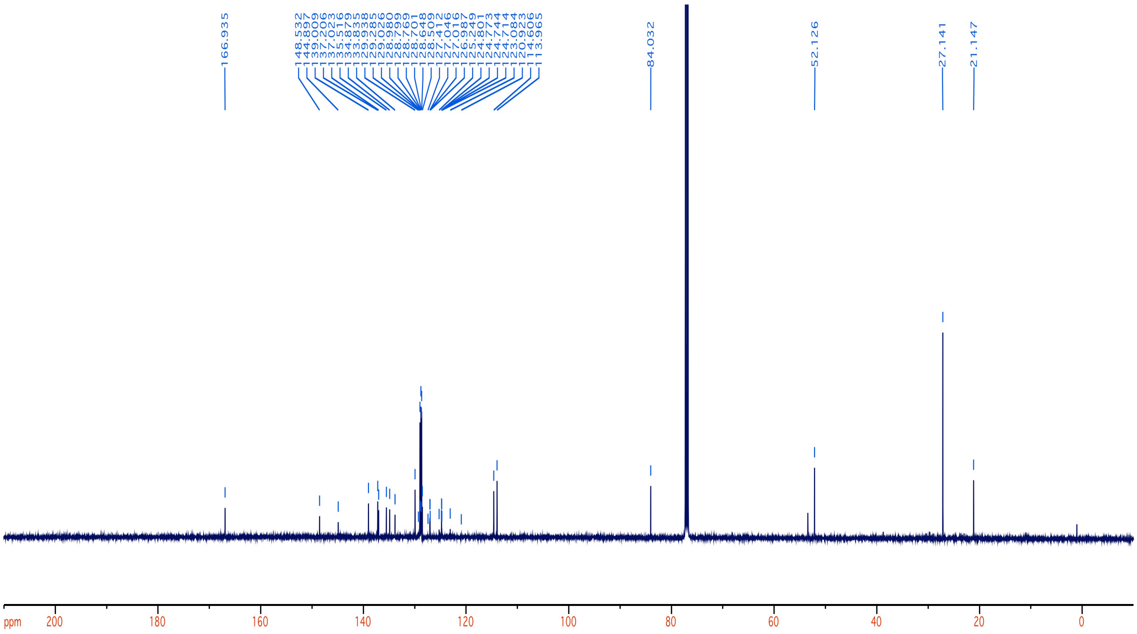

Pyrrole 6c: ^{19}F NMR (377 MHz, $CDCl_3$)

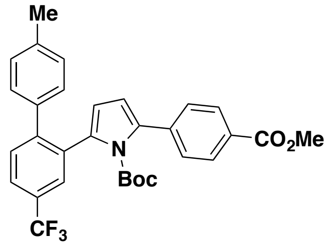

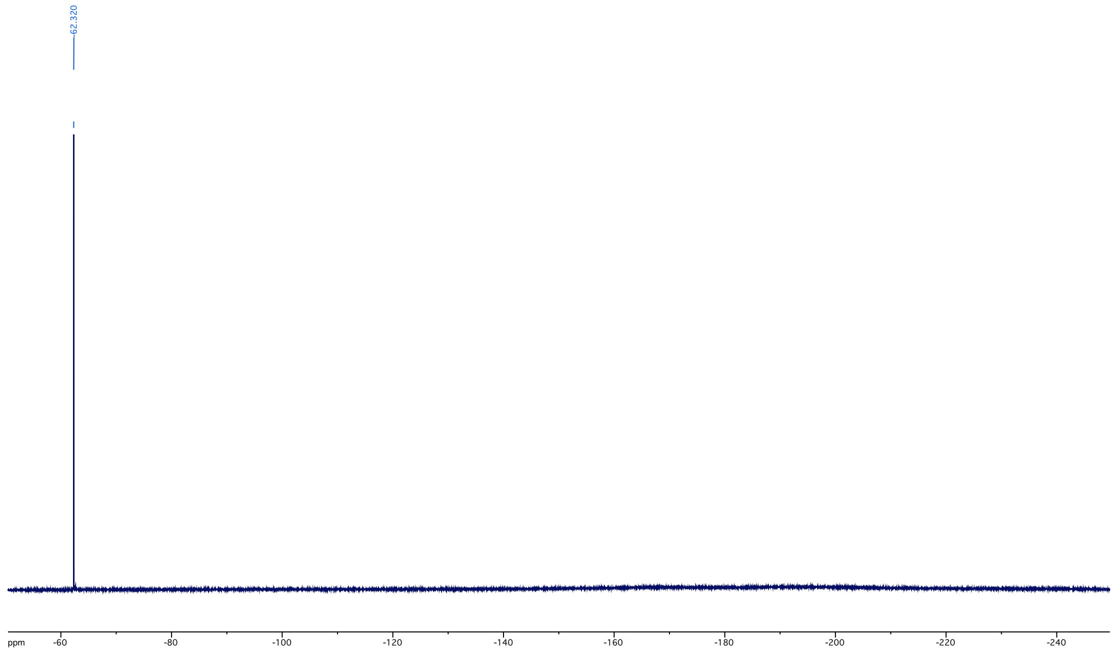

Dihydropyrrole 7a: ^{1}H NMR (400 MHz, $CDCl_3$)

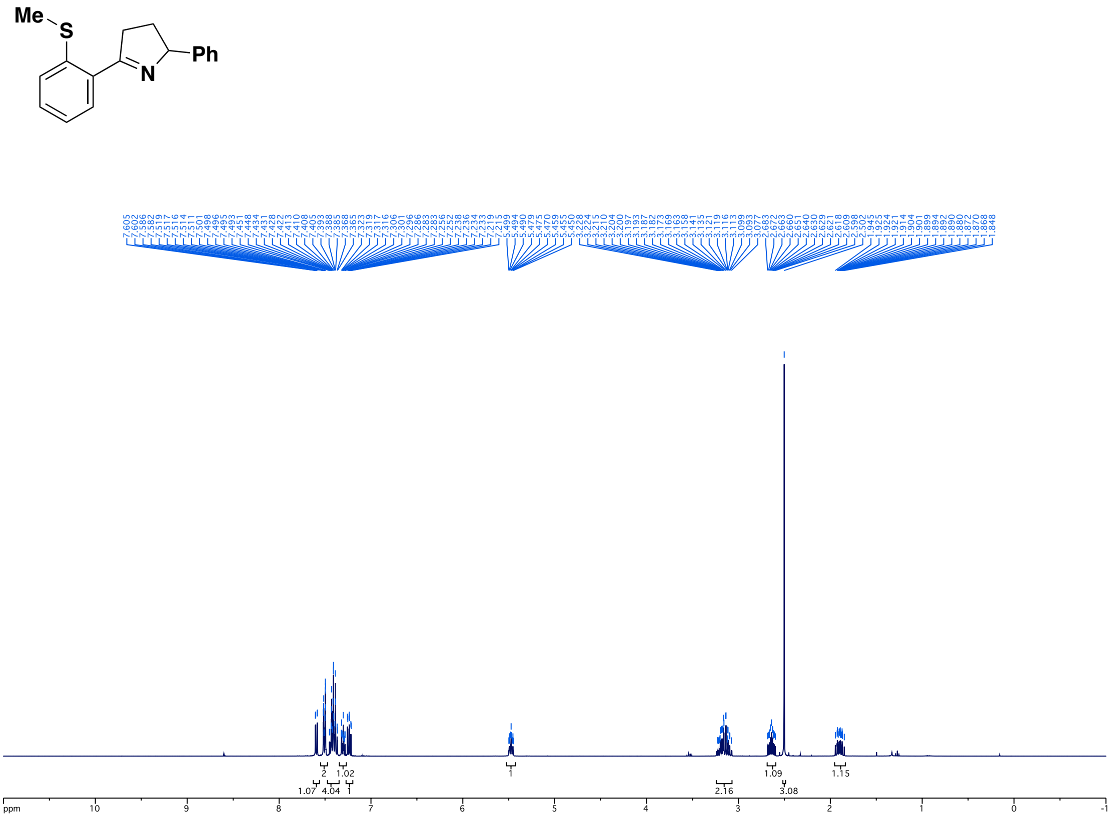

Dihydropyrrole 7a: ^{13}C NMR (101 MHz, $CDCl_3$)

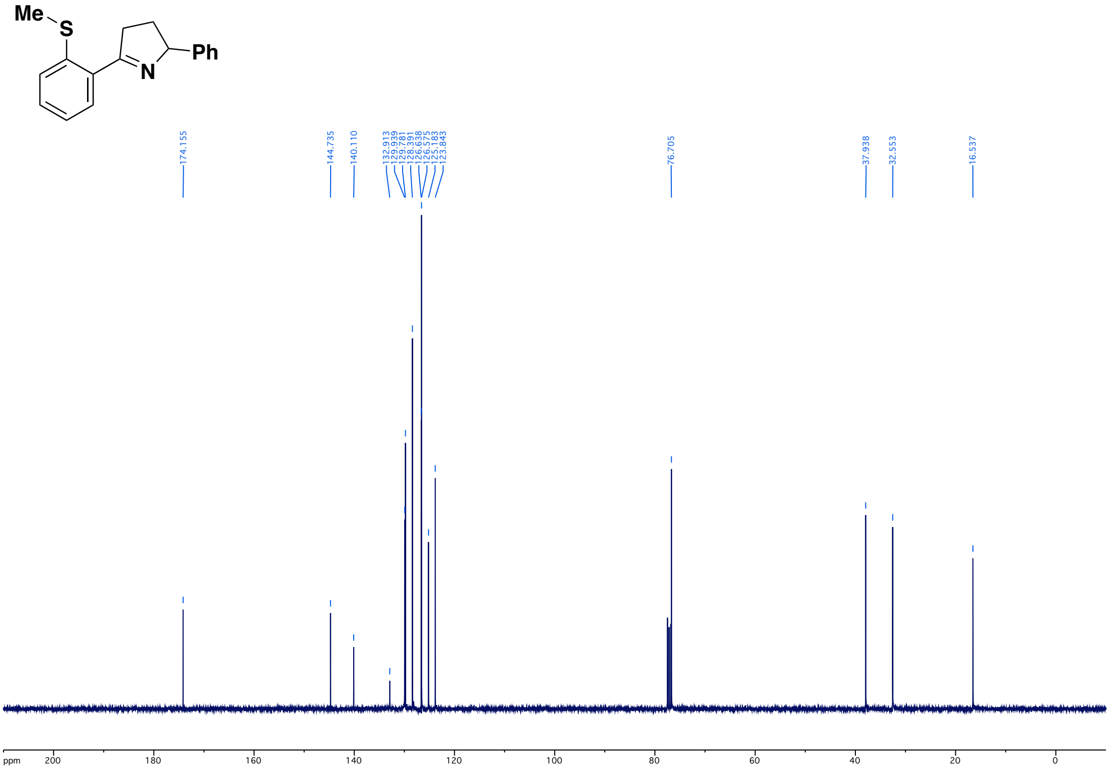

Dihydropyrrole 7b: ^{1}H NMR (400 MHz, $CDCl_3$)

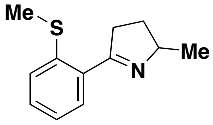

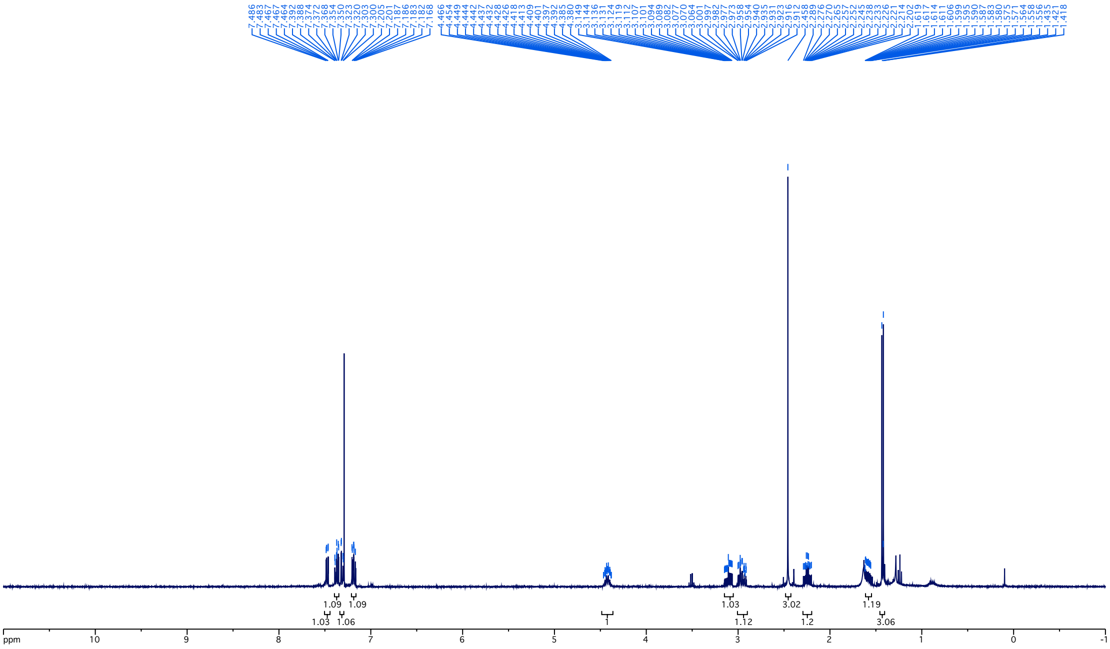

Dihydropyrrole 7b: ^{13}C NMR (101 MHz, $CDCl_3$)

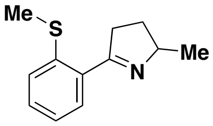

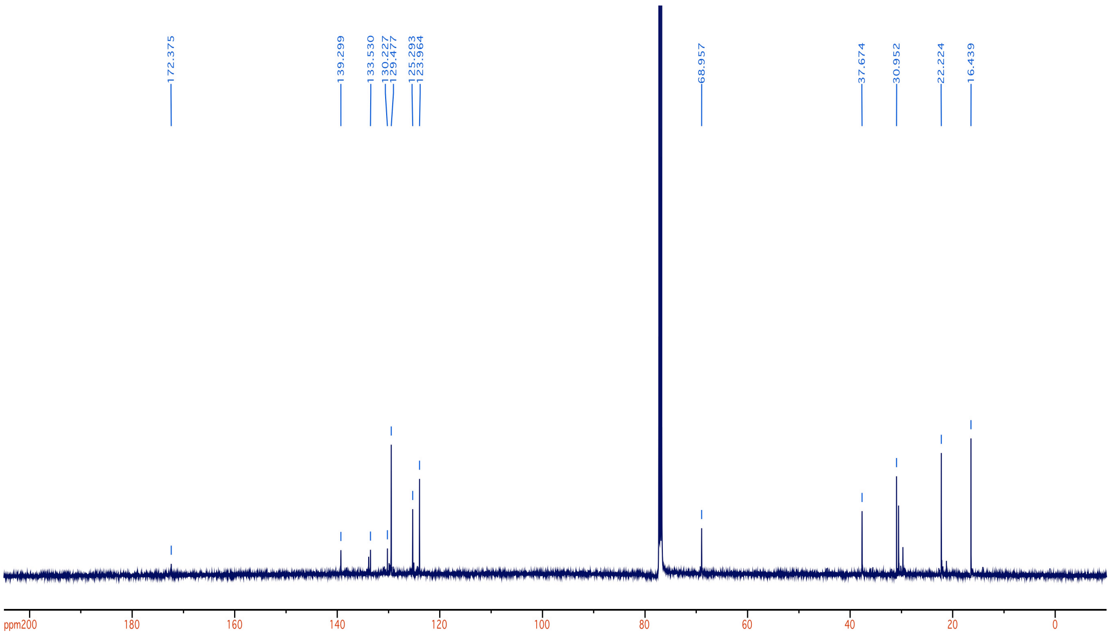

Dihydropyrrole 7c: ^{1}H NMR (400 MHz, $CDCl_3$)

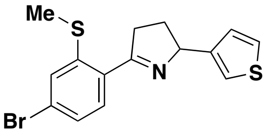

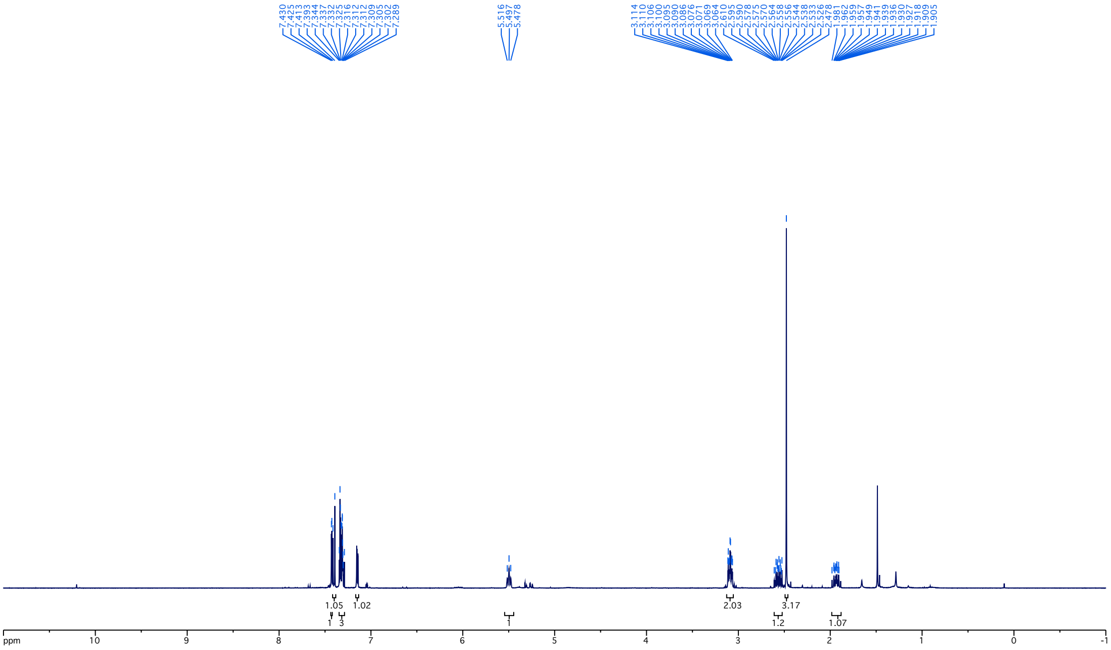

Dihydropyrrole 7c: ^{13}C NMR (101 MHz, $CDCl_3$)

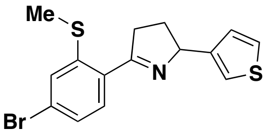

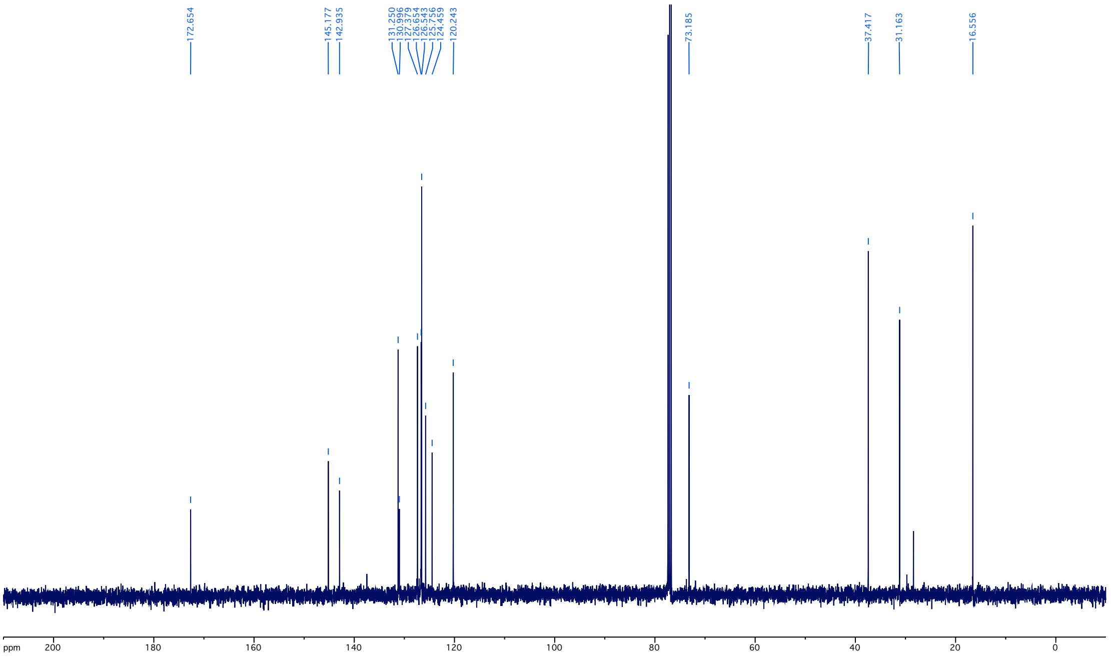

Dihydropyrrole 7d: ^{1}H NMR (400 MHz, $CDCl_3$)

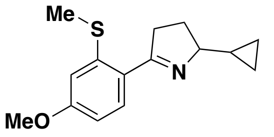

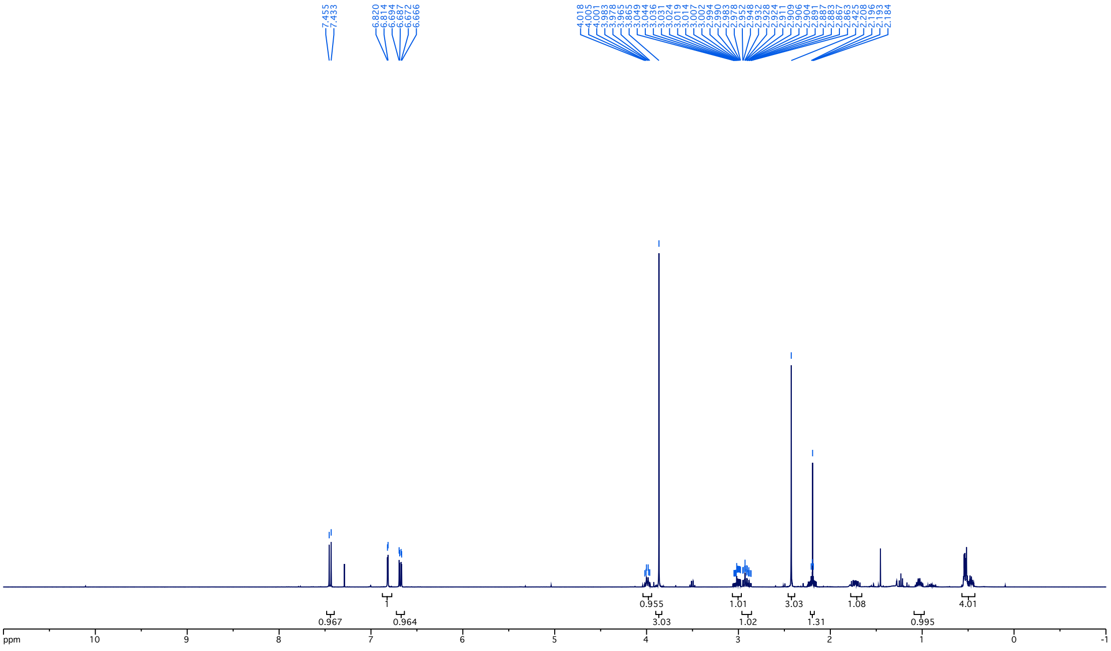

Dihydropyrrole 7d: ^{13}C NMR (101 MHz, $CDCl_3$)

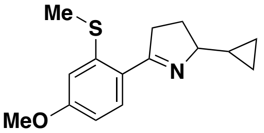

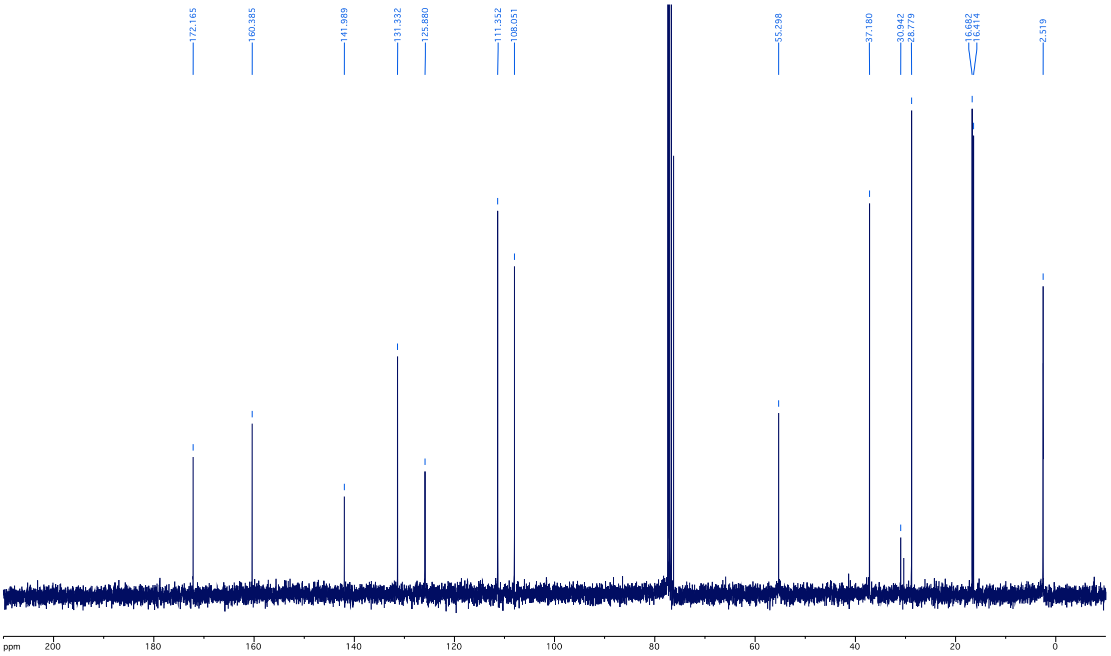

Dihydropyrrole 7e: ^{1}H NMR (400 MHz, $CDCl_3$)

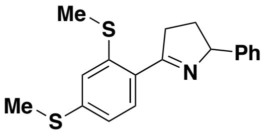

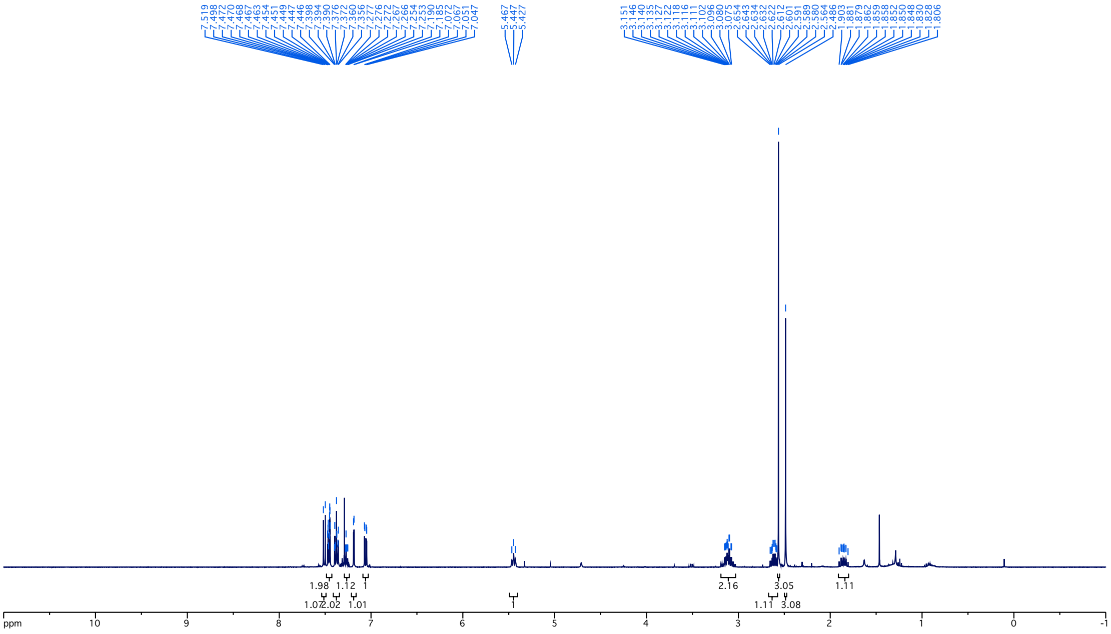

Dihydropyrrole 7e: ^{13}C NMR (101 MHz, $CDCl_3$)

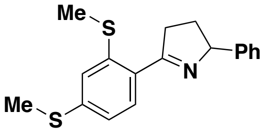

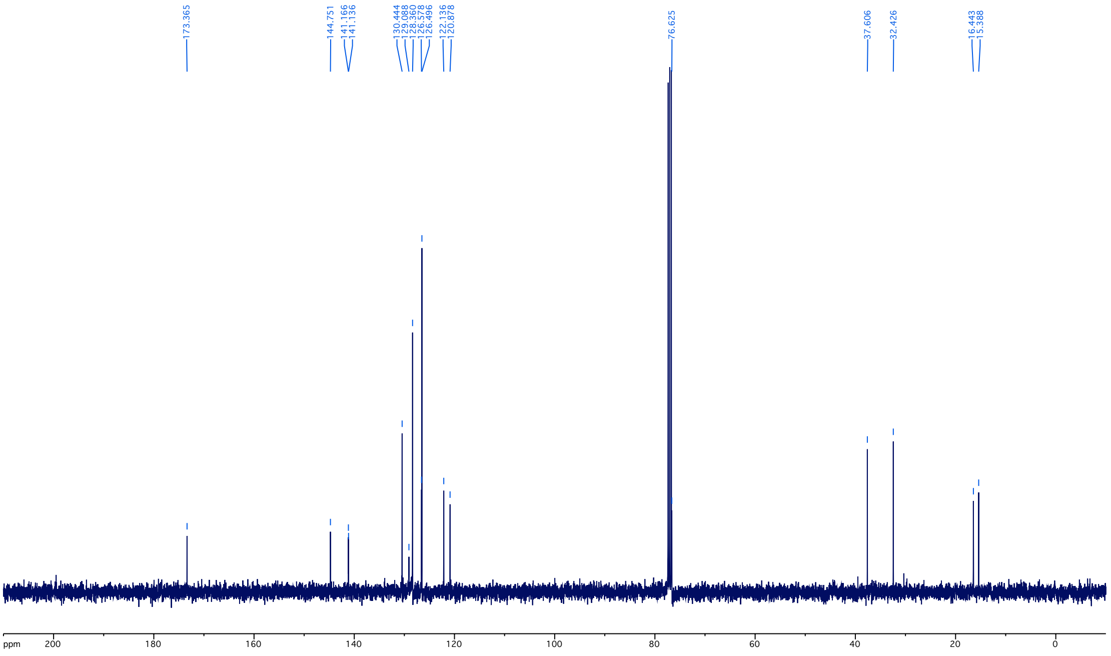

Dihydropyrrole 7f: ^{1}H NMR (400 MHz, $CDCl_3$)

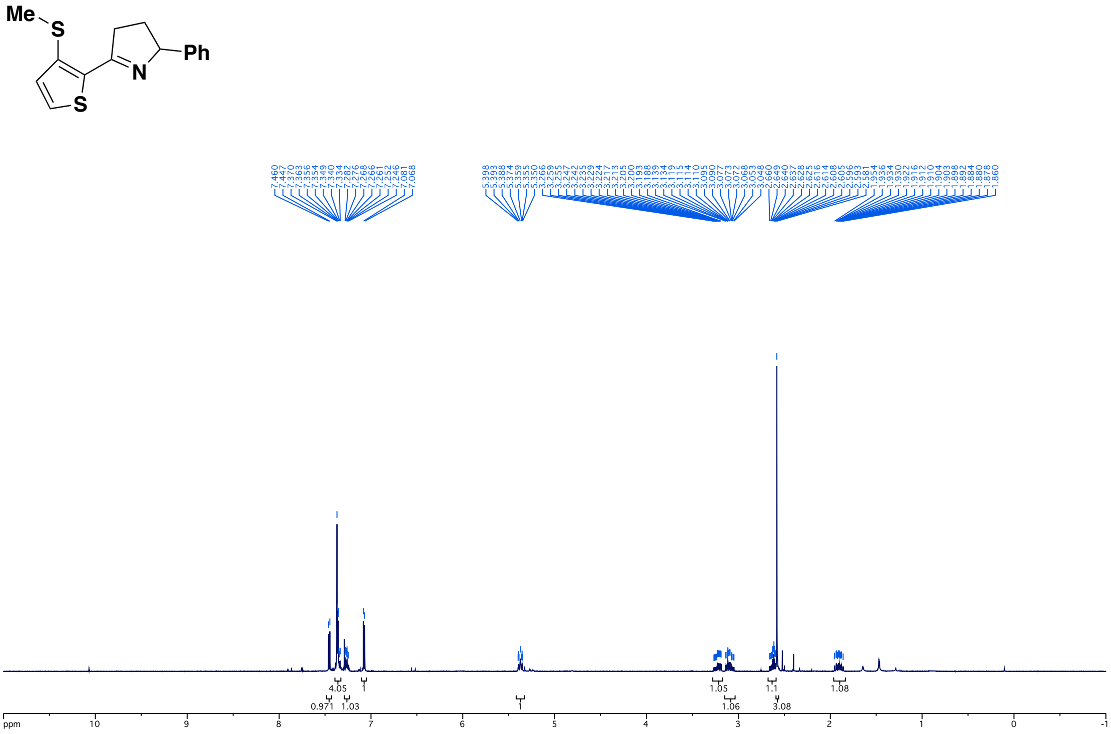

Dihydropyrrole 7f: ^{13}C NMR (101 MHz, $CDCl_3$)

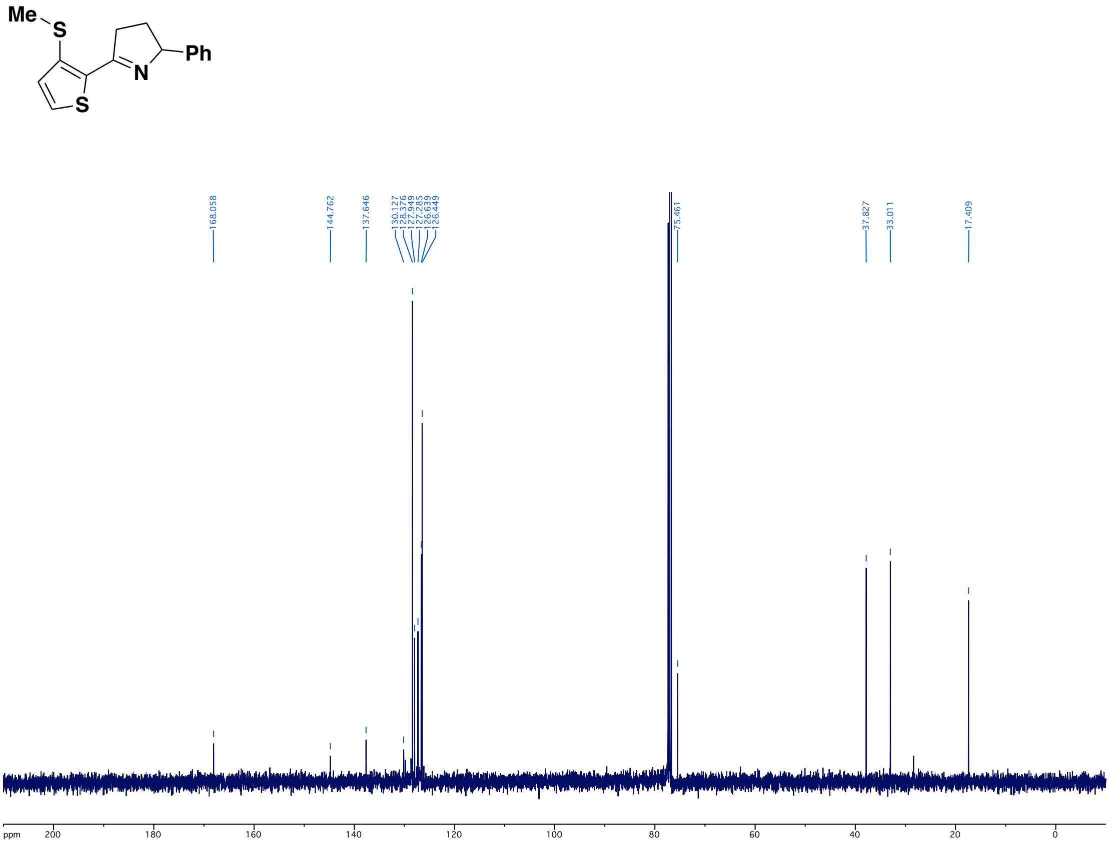

Dihydropyrrole 7g: ^{1}H NMR (400 MHz, $CDCl_3$)

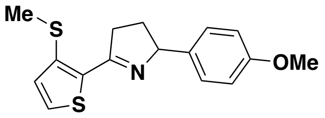

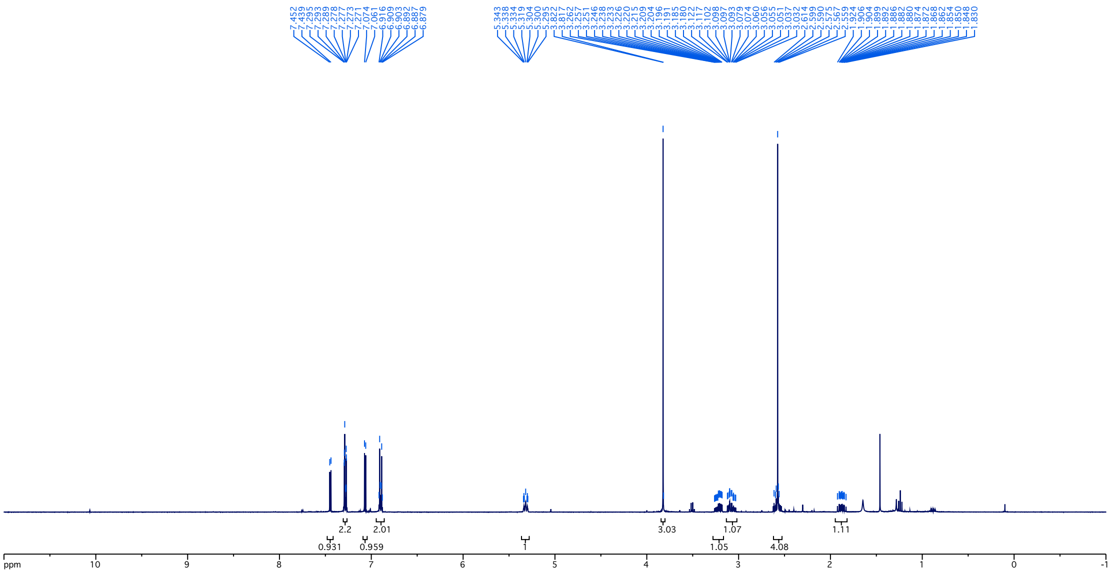

Dihydropyrrole 7g: ^{13}C NMR (101 MHz, $CDCl_3$)

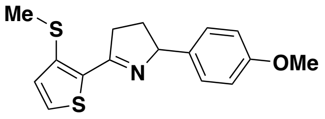

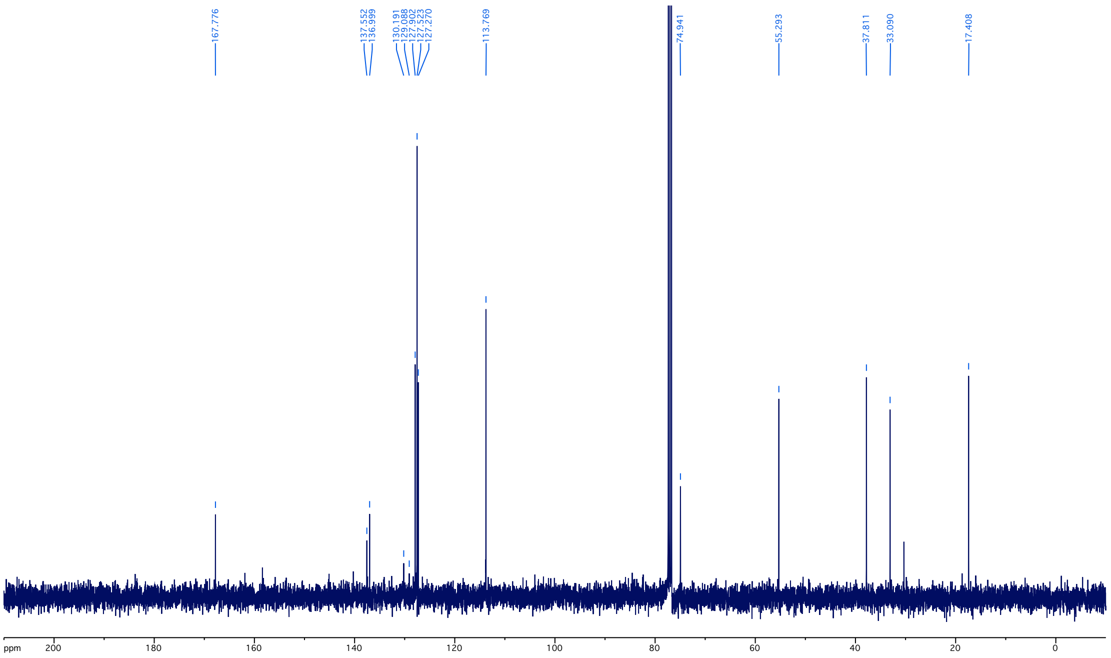

Dihydropyrrole 7h: ^{1}H NMR (400 MHz, $CDCl_3$)

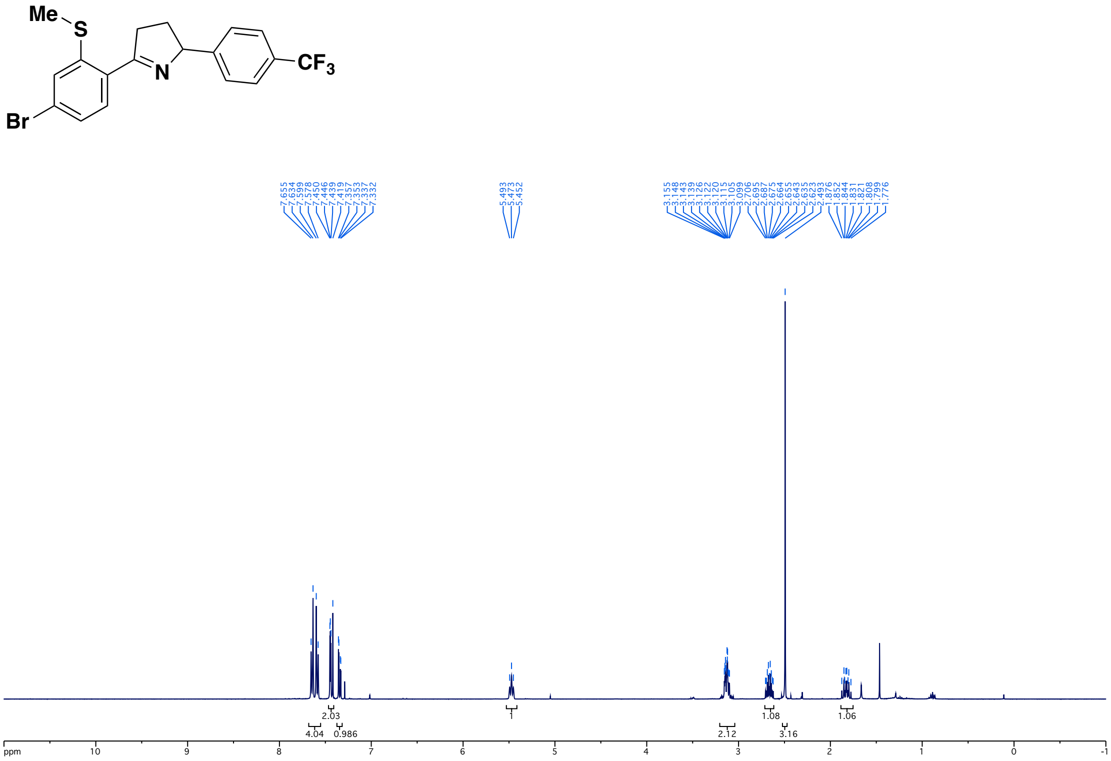

Dihydropyrrole 7h: ^{13}C NMR (101 MHz, $CDCl_3$)

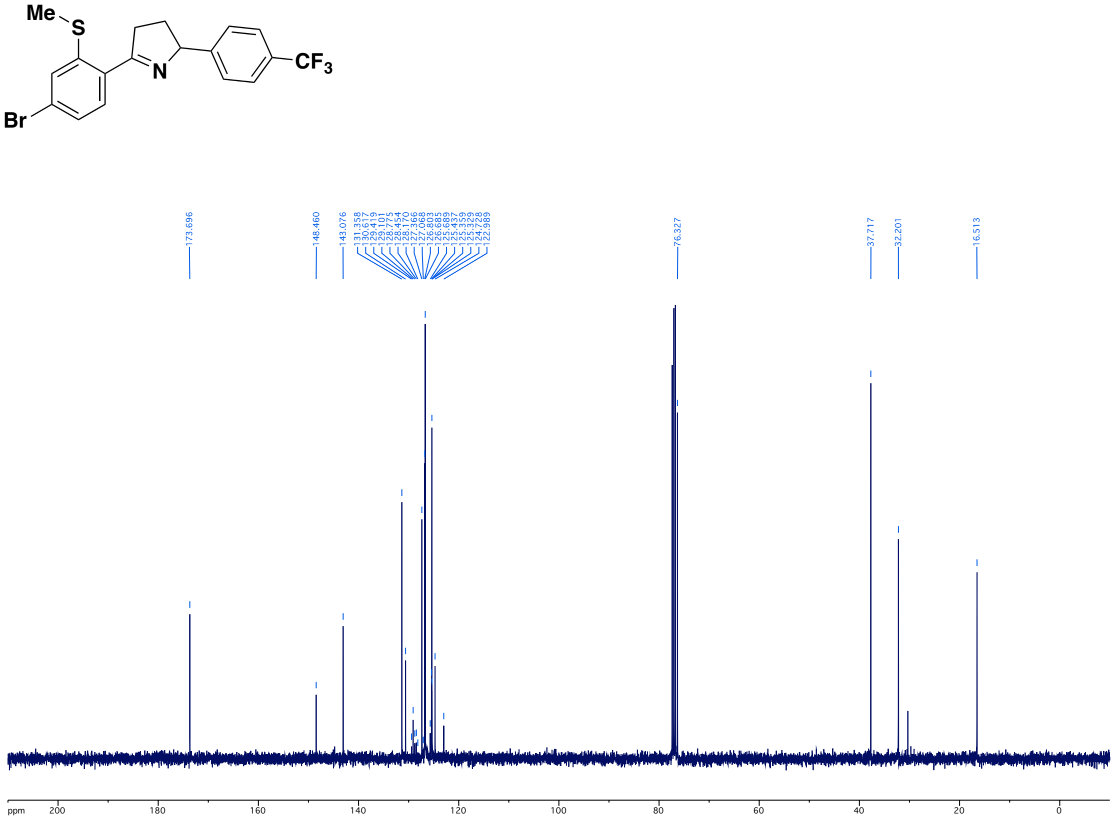

Dihydropyrrole 7h: ^{19}F NMR (377 MHz, $CDCl_3$)

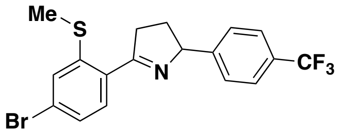

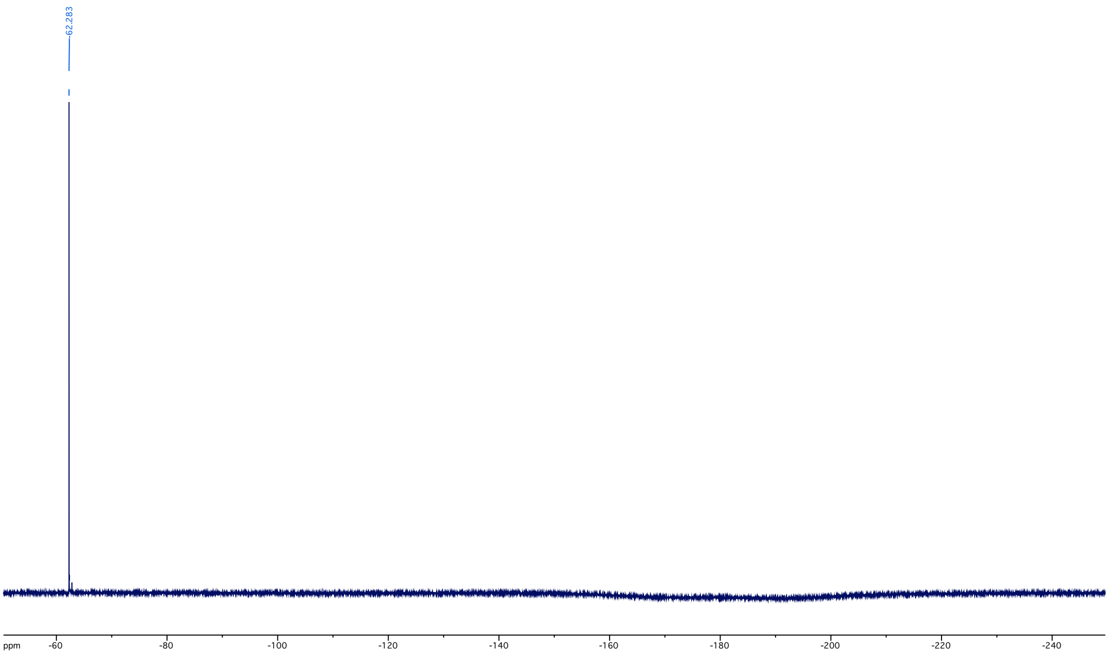

Pyrrolidine 7a: ^{1}H NMR (400 MHz, $CDCl_3$)

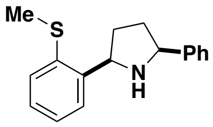

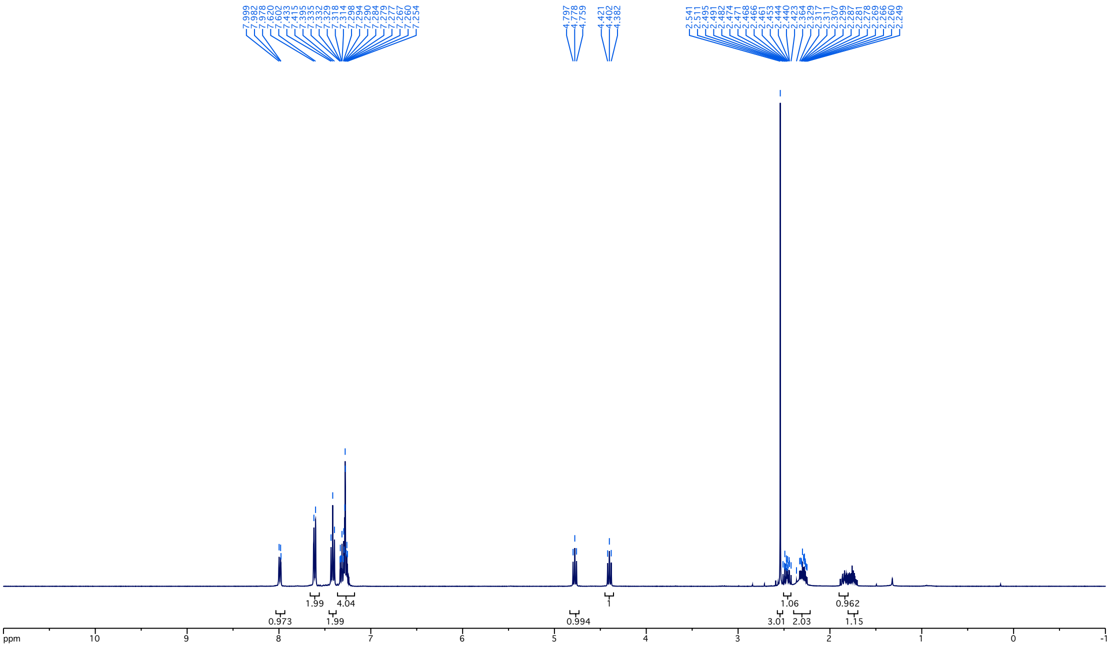

Pyrrolidine 7a: ^{13}C NMR (101 MHz, $CDCl_3$)

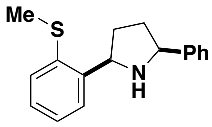

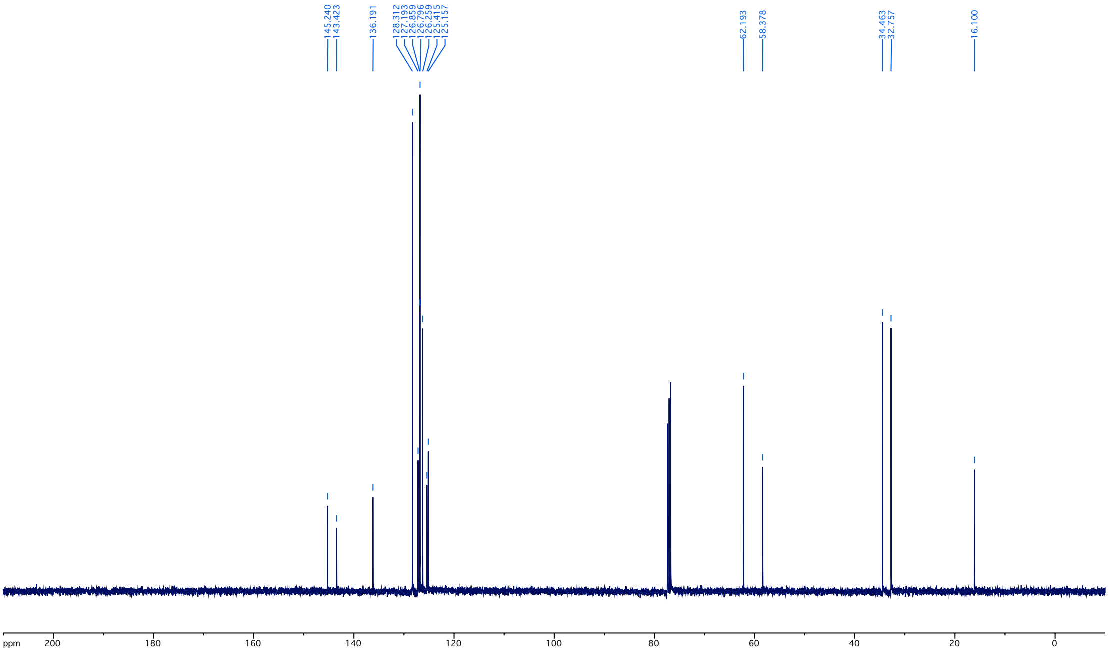

Pyrrolidine 8b: ^{1}H NMR (400 MHz, $CDCl_3$)

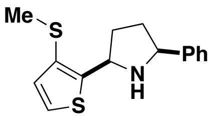

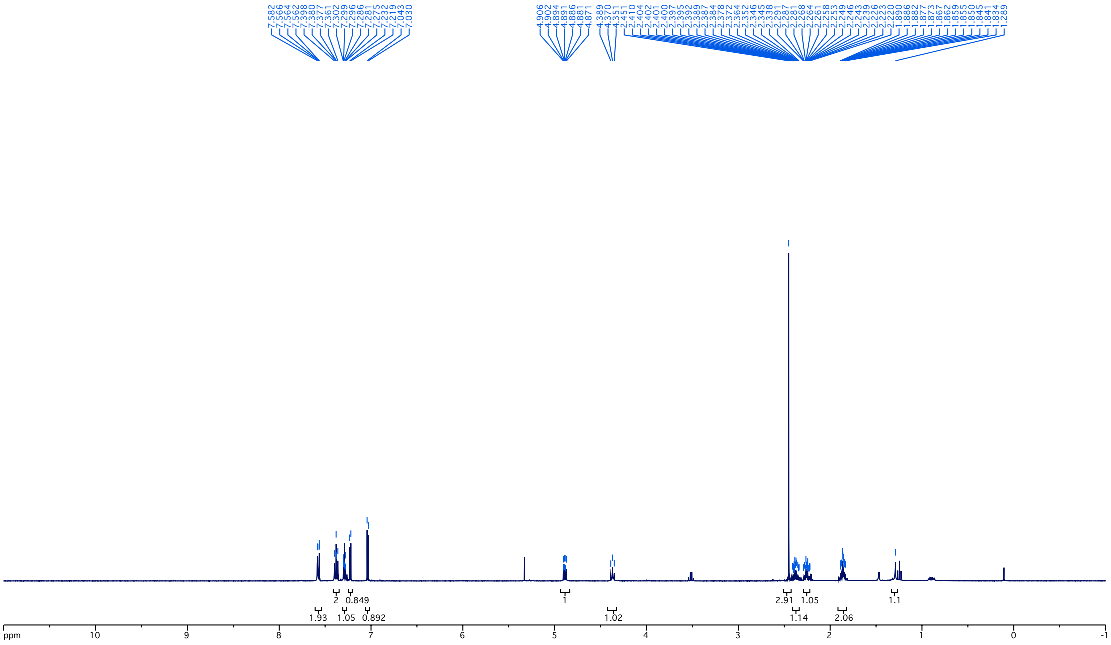

Pyrrolidine 8b: ^{13}C NMR (101 MHz, $CDCl_3$)

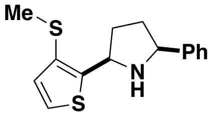

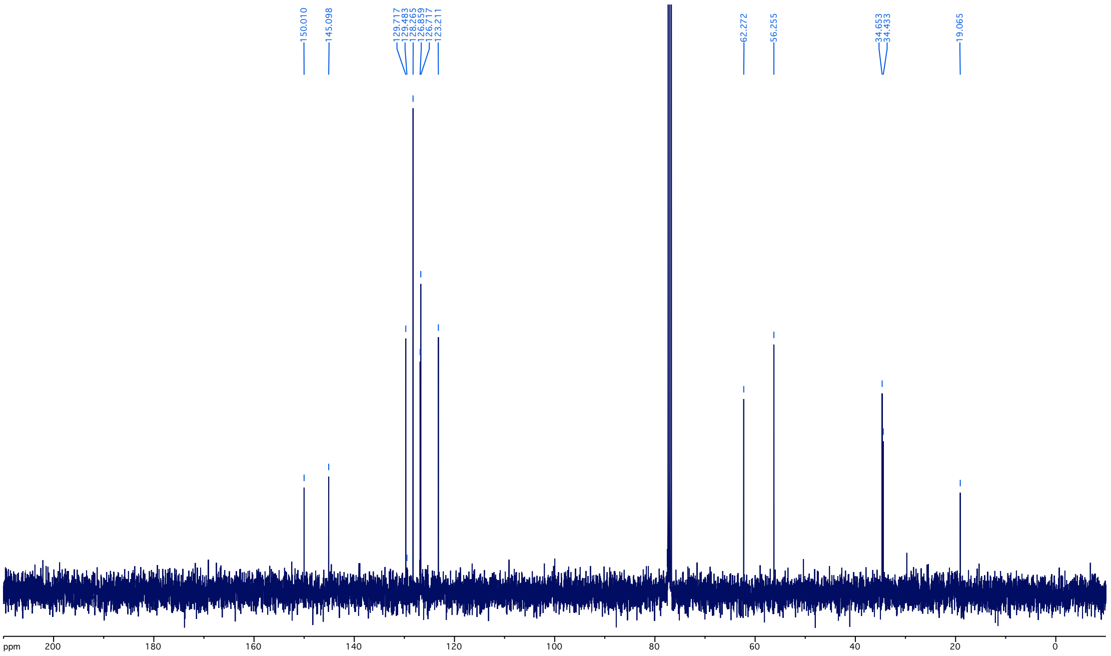

Pyrrolidine 8c: ^{1}H NMR (400 MHz, $CDCl_3$)

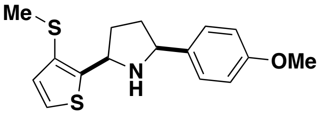

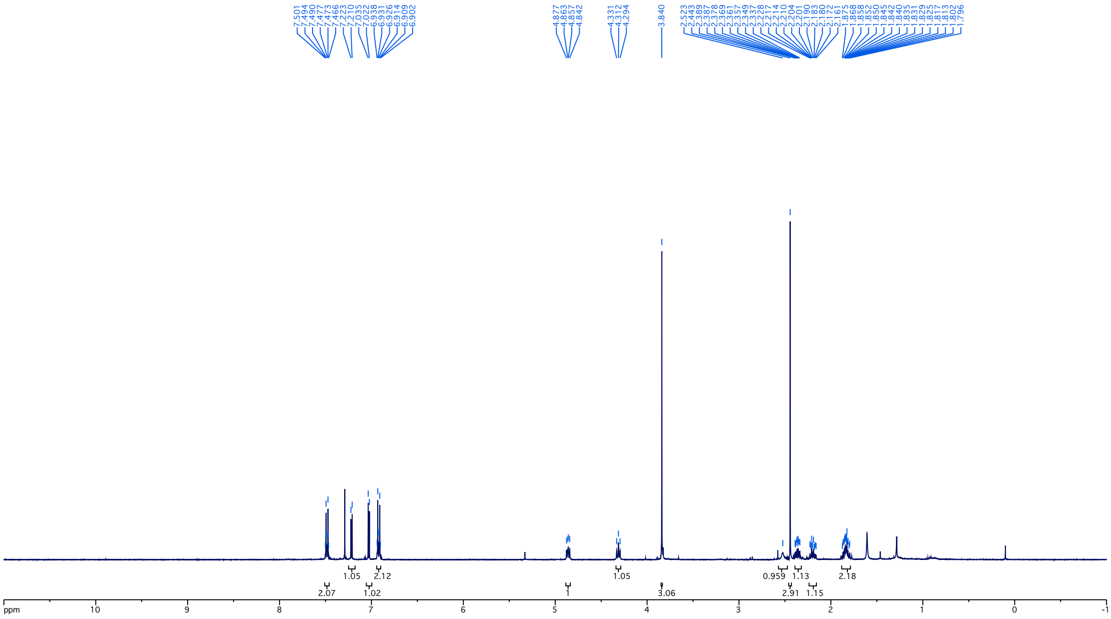

Pyrrolidine 8c: ^{13}C NMR (101 MHz, $CDCl_3$)

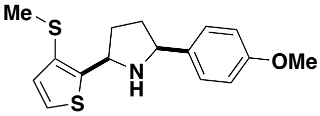

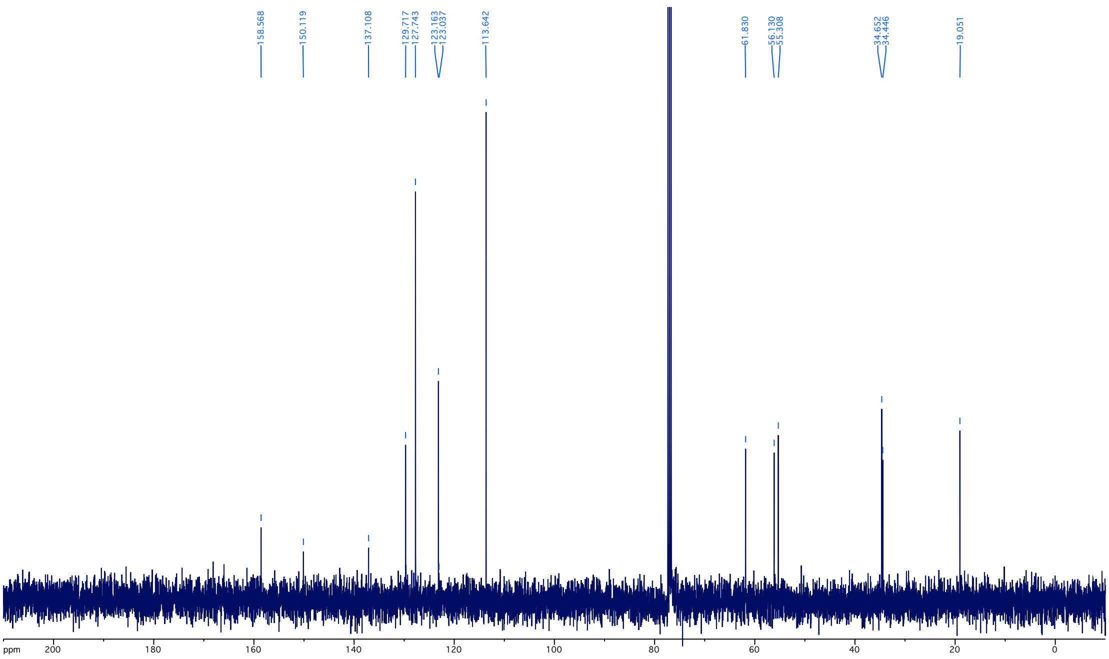

Pyrrolidine 8d: ^{1}H NMR (400 MHz, $CDCl_3$)

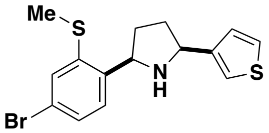

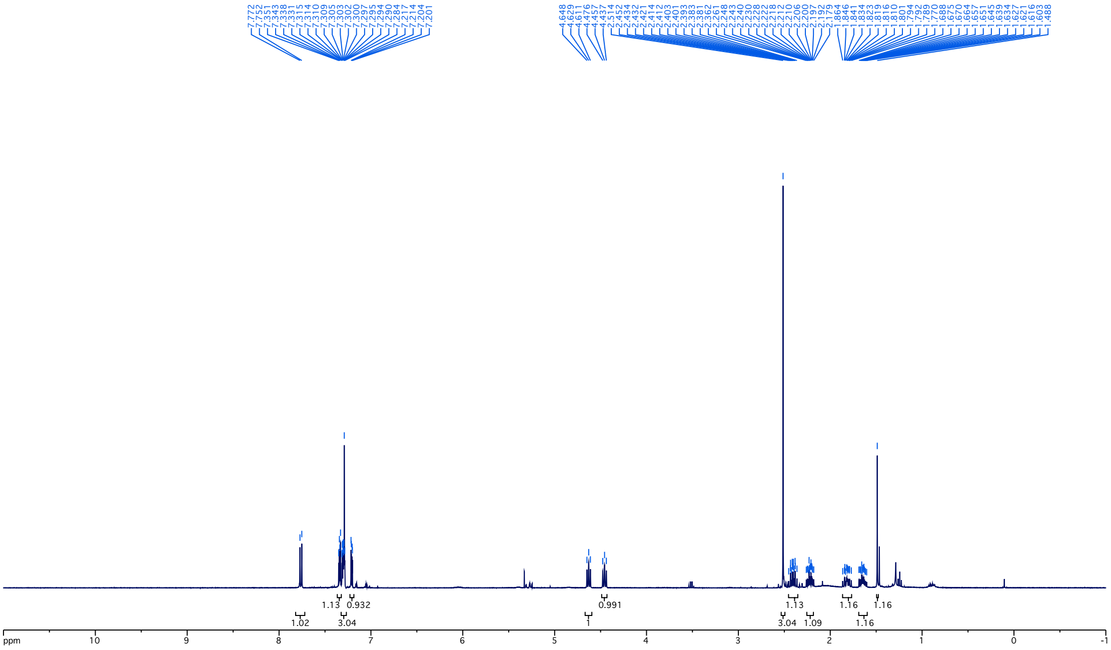

Pyrrolidine 8d: ^{13}C NMR (101 MHz, $CDCl_3$)

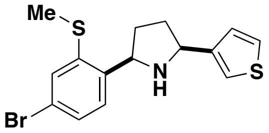

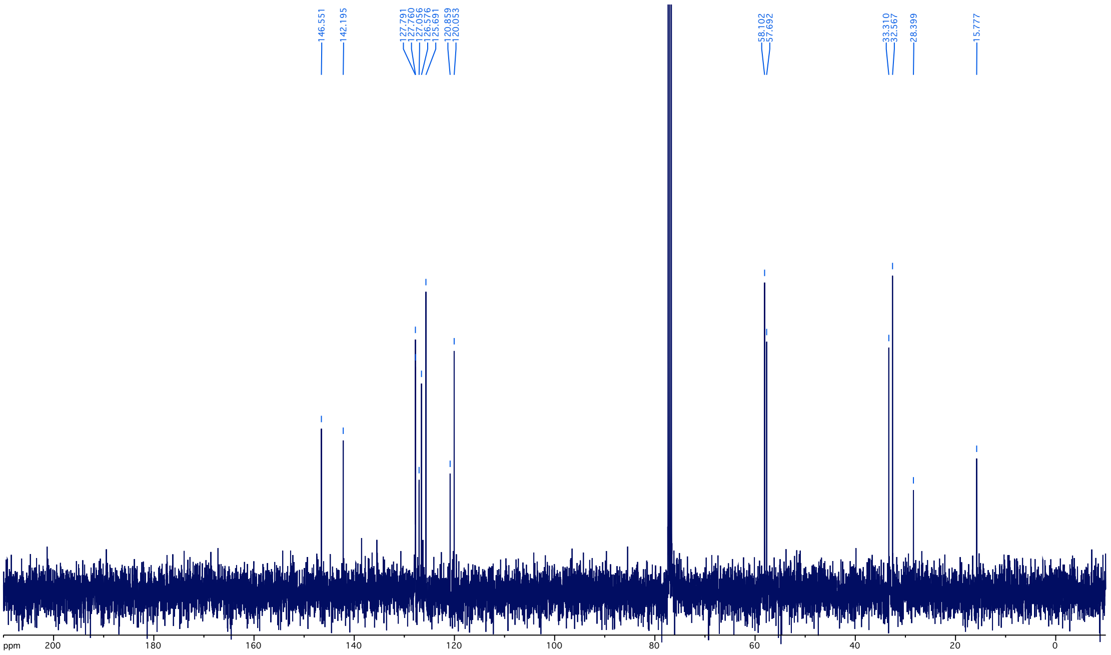

Pyrrolidine 8e: ^{1}H NMR (400 MHz, $CDCl_3$)

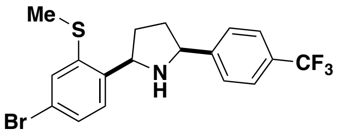

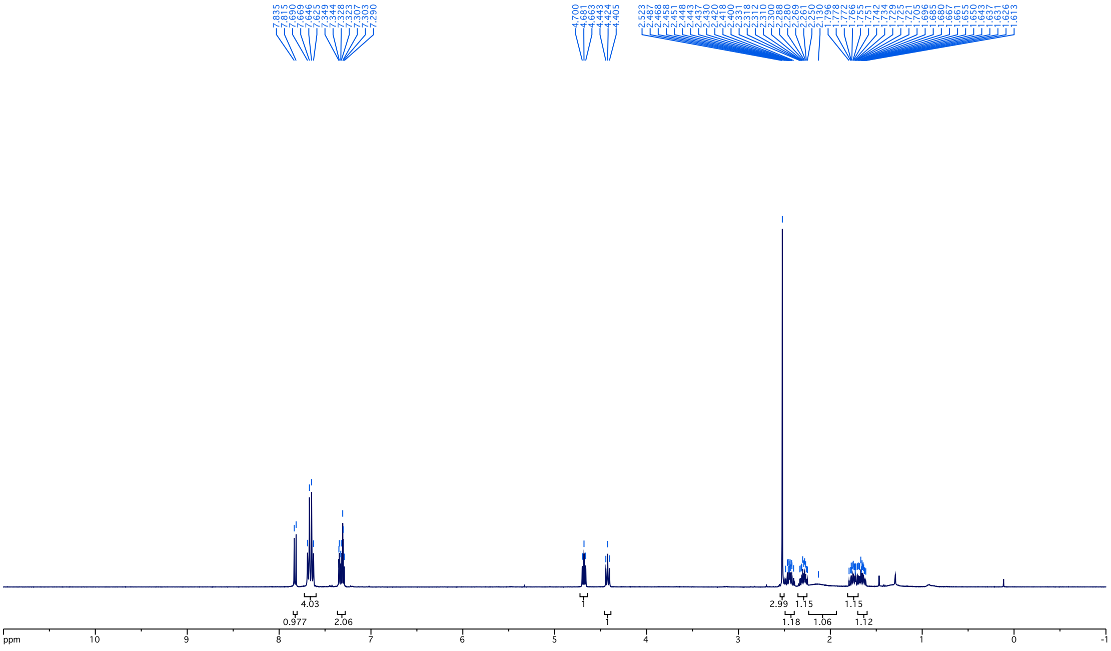

Pyrrolidine 8e: ^{13}C NMR (101 MHz, $CDCl_3$)

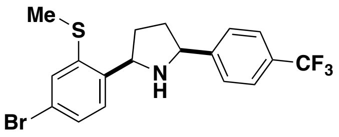

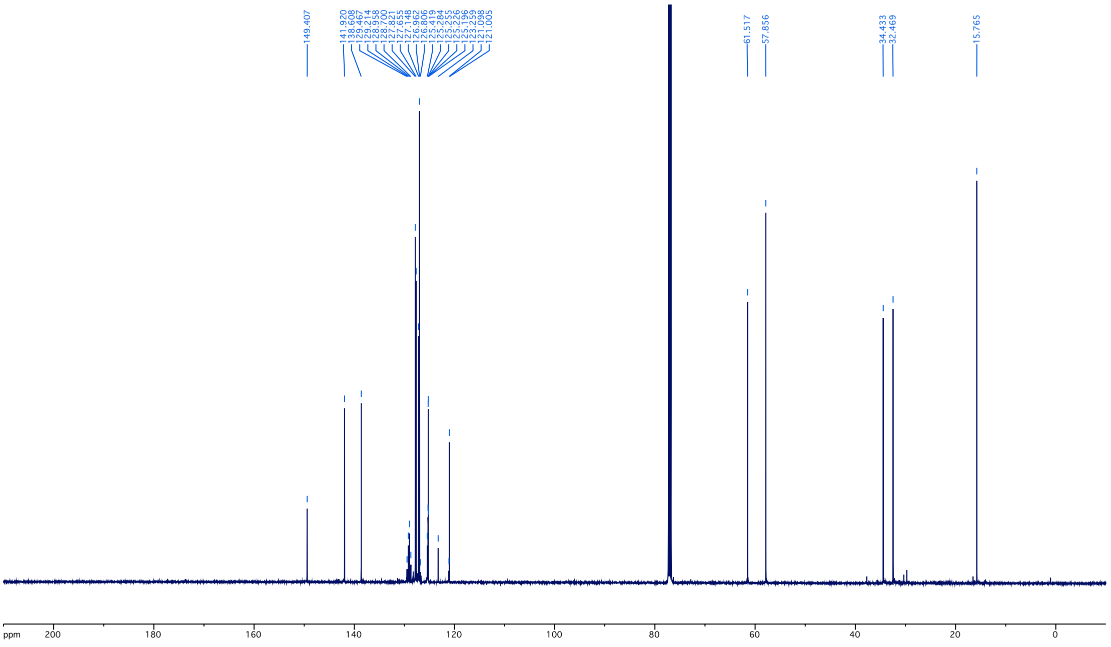

Pyrrolidine 8e: ^{19}F NMR (377 MHz, $CDCl_3$)

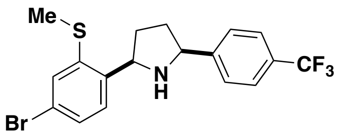

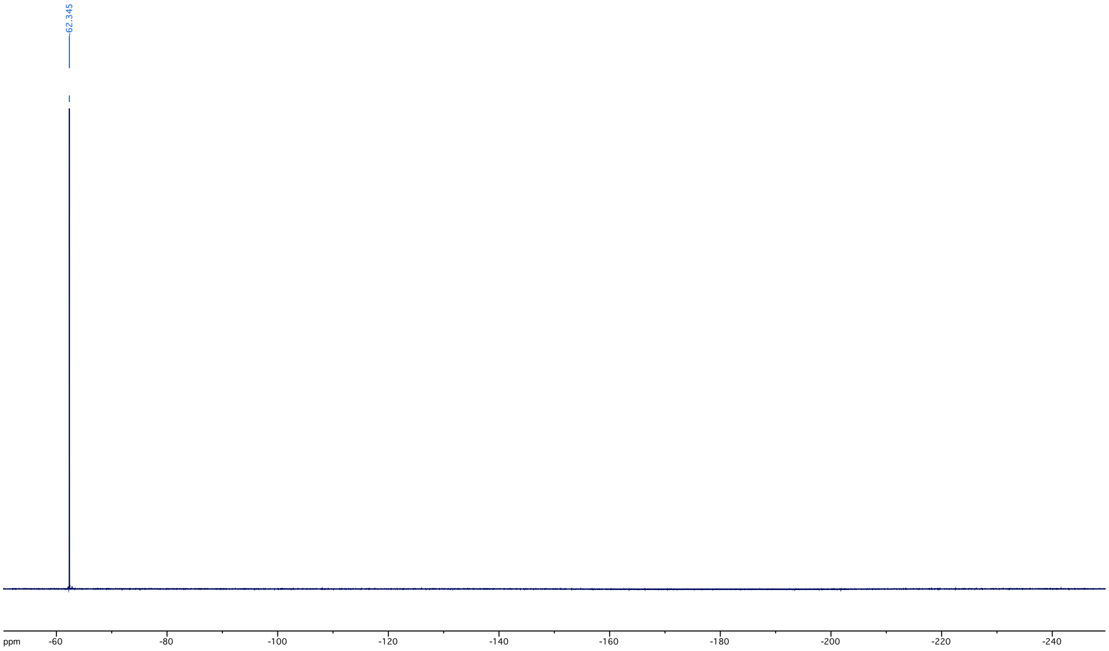

Pyrrolidine 8f: ^{1}H NMR (400 MHz, $CDCl_3$)

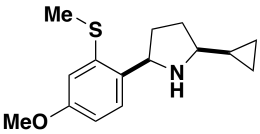

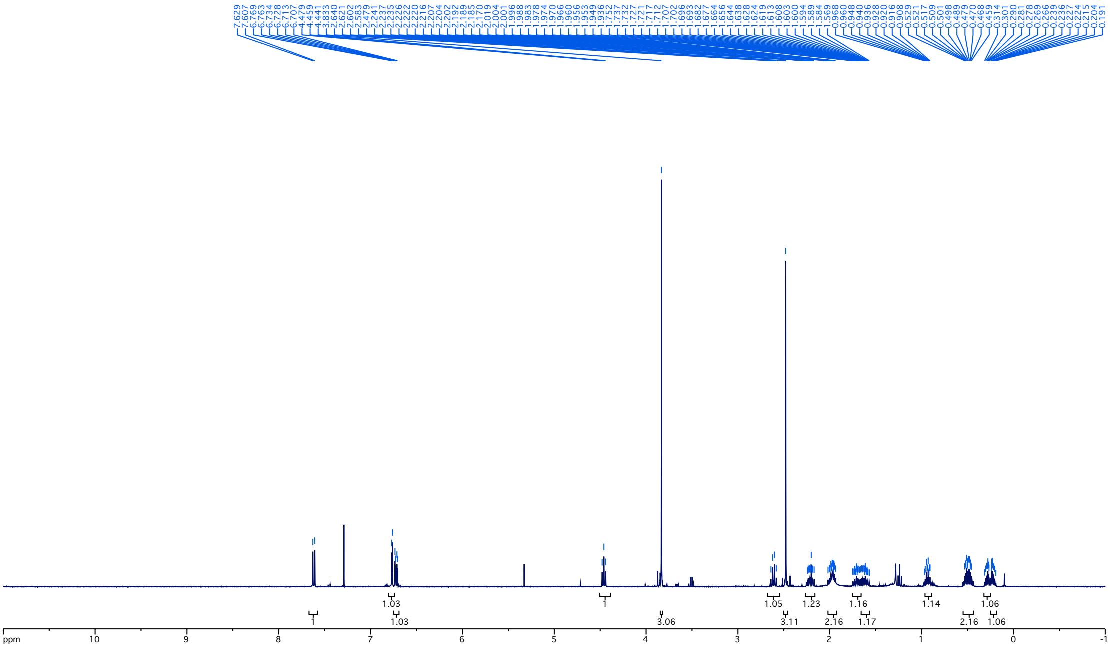

Pyrrolidine 8f: ^{13}C NMR (101 MHz, $CDCl_3$)

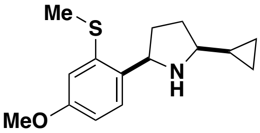

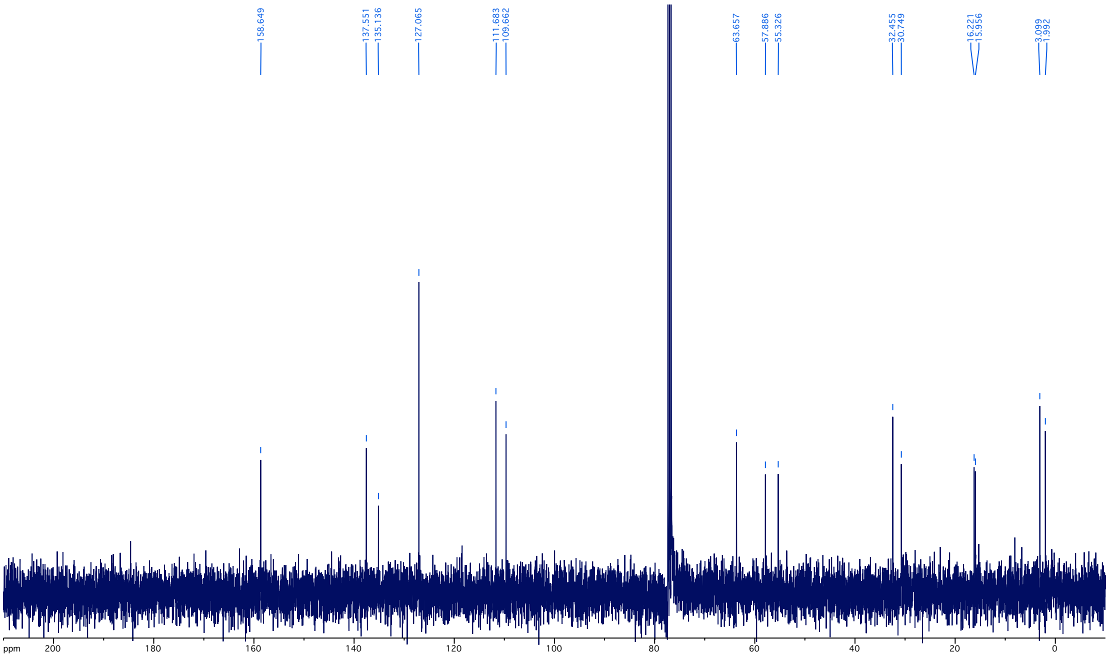

Pyrrolidine 8g: ^{1}H NMR (400 MHz, $CDCl_3$)

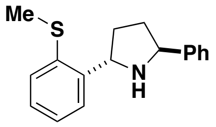

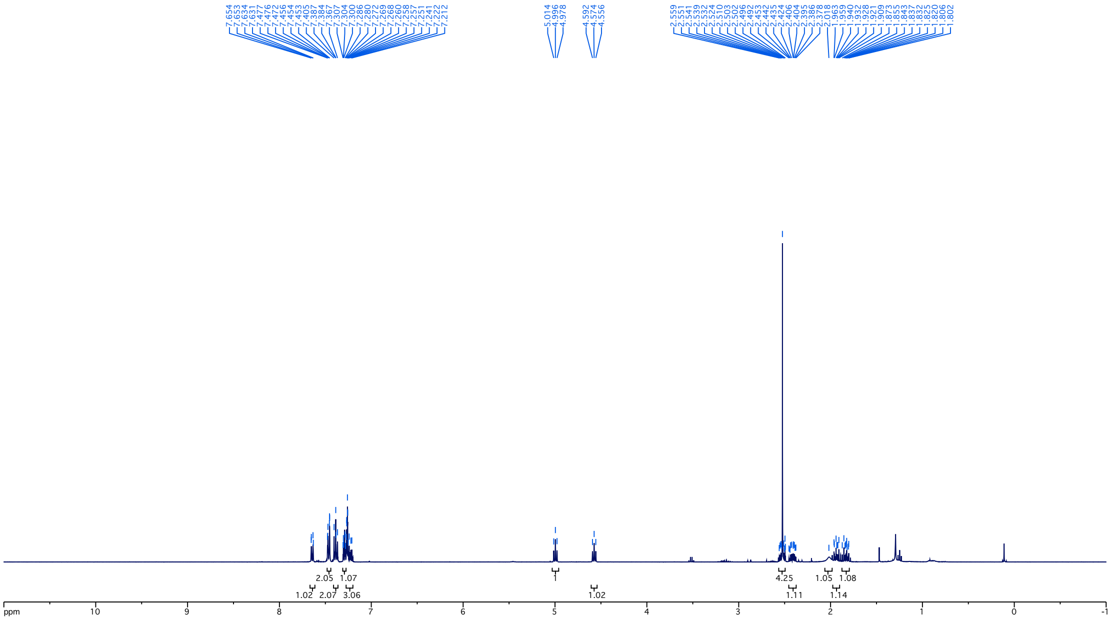

Pyrrolidine 8g: ^{13}C NMR (101 MHz, $CDCl_3$)

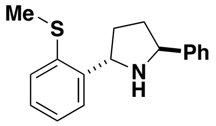

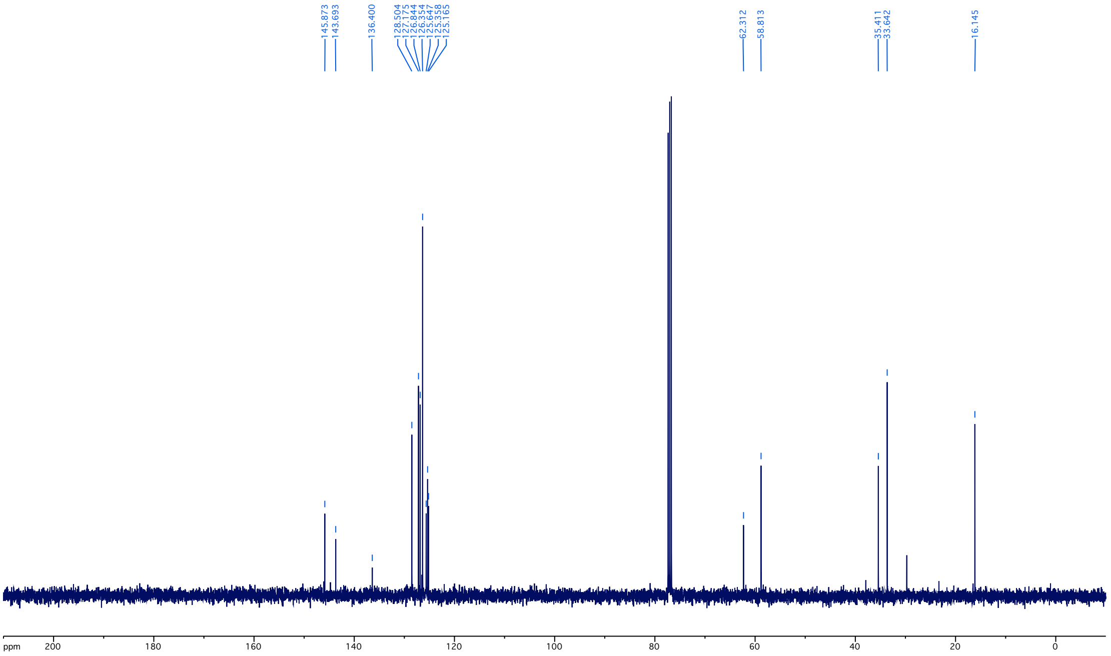

Pyrrolidine 8h: ^{1}H NMR (400 MHz, $CDCl_3$)

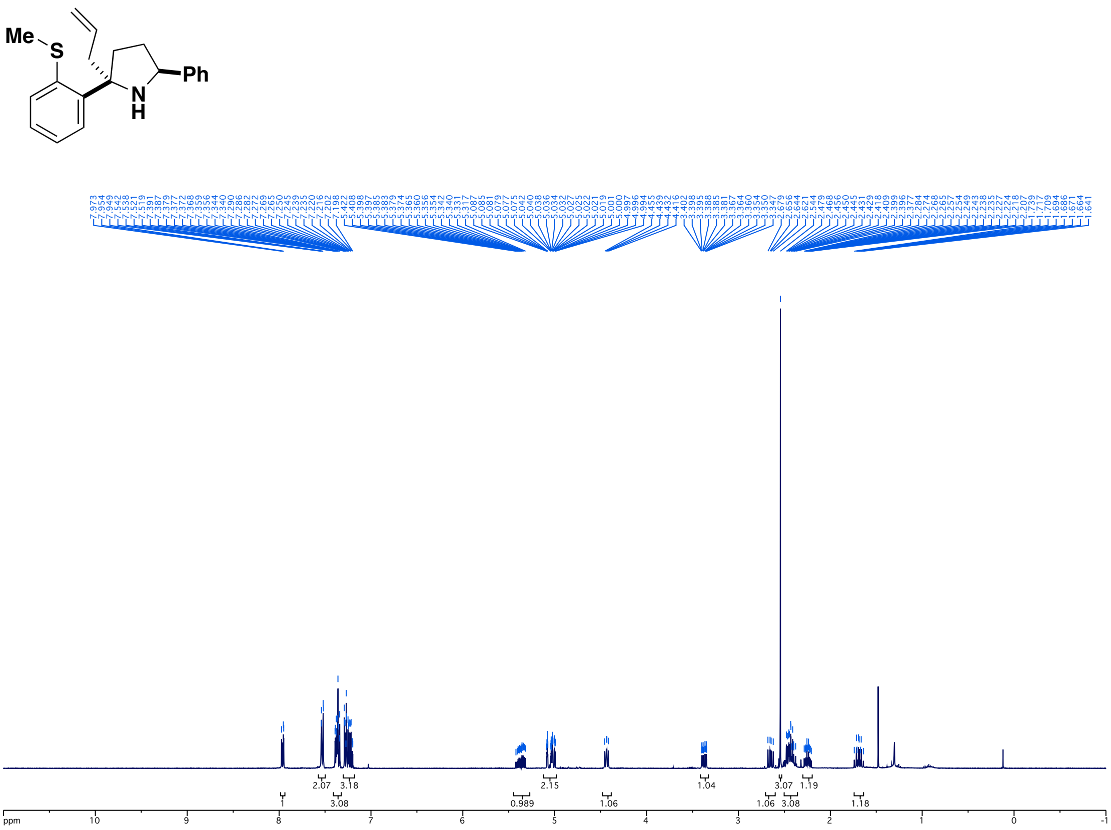

Pyrrolidine 8h: ^{13}C NMR (101 MHz, $CDCl_3$)

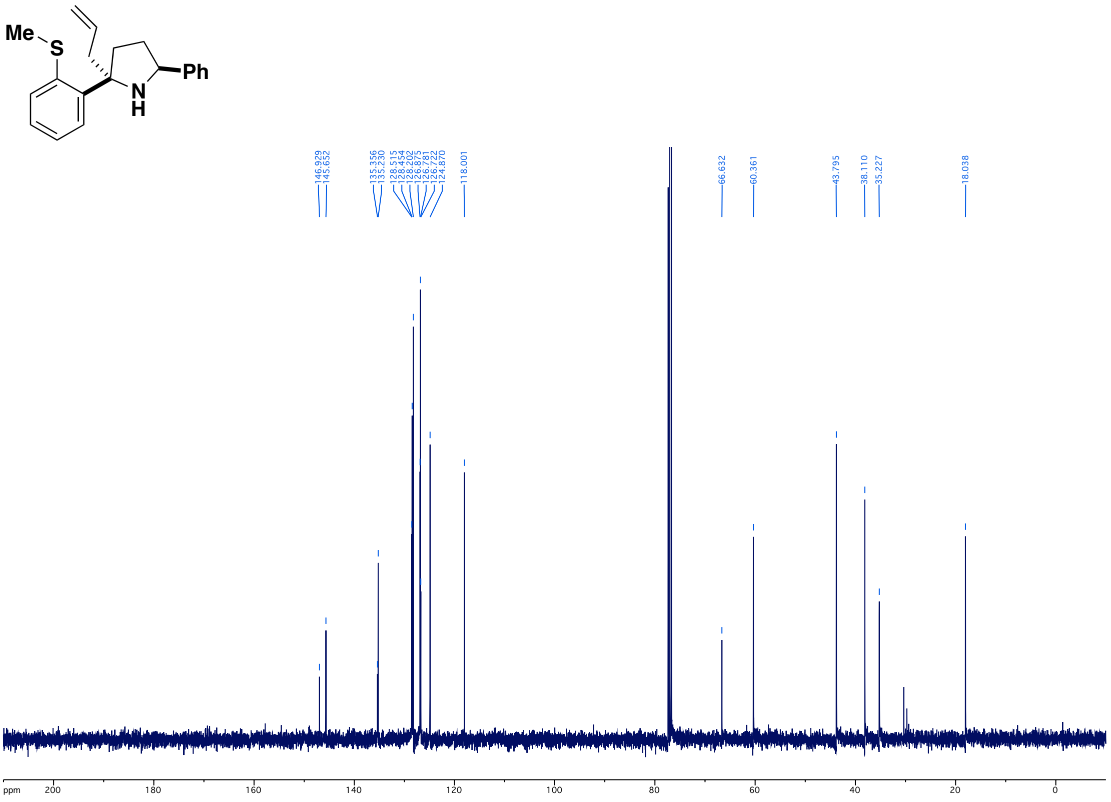

Supplement: Supplementary file 1 — Supplementary [file CHEM-22-7879-s001.pdf]
